# From Fermentation to Function: Genomic Diversity and Probiotic Potential in the Reclassified *Lactobacillus* Lineage

**DOI:** 10.34133/csbj.0004

**Published:** 2026-03-16

**Authors:** Nattarika Chaichana, Kamonnut Singkhamanan, Monwadee Wonglapsuwan, Sarunyou Chusri, Rattanaruji Pomwised, Komwit Surachat

**Affiliations:** ^1^ Department of Biomedical Sciences and Biomedical Engineering, Faculty of Medicine, Prince of Songkla University, Hat Yai, Songkhla 90110, Thailand.; ^2^ Division of Biological Science, Faculty of Science, Prince of Songkla University, Hat Yai,Songkla, Thailand.; ^3^ Division of Infectious Diseases, Department of Internal Medicine, Faculty of Medicine, Prince of Songkla University, Hatyai, Songkhla 90110, Thailand.

## Abstract

The genus *Lactobacillus* has long been recognized for its essential roles in food fermentation and as a key component of probiotic therapy. Recent advancements in taxonomic classification have led to the reorganization of many species into separate genera, opening new avenues for understanding their genetic and functional diversity. In this study, we performed an extensive genomic comparison of 3,813 high-quality genomes from 35 species previously classified under *Lactobacillus*. Our focus was on identifying functional genes, bacteriocin clusters, secondary metabolite biosynthesis gene clusters (BGCs), and carbohydrate-active enzymes (CAZymes), which are central to the beneficial properties of these bacteria. Among the species analyzed, *Lactiplantibacillus plantarum* stood out due to its adaptability in carbohydrate metabolism and its ability to perform vital probiotic potential, such as antimicrobial activity, γ-aminobutyric acid (GABA) production, and resilience to environmental stress. We also observed important species-specific variation in functional traits, with *Lacticaseibacillus rhamnosus* showing high prevalence of antimicrobial genes, while *Lactobacillus iners* displayed specialized metabolic pathways that enable it to thrive in unique ecological environments. Notably, no classical virulence factors were detected, indicating a generally favorable genomic safety profile, although experimental validation remains necessary prior to probiotic application. This study highlights the remarkable genetic and functional diversity within the reclassified *Lactobacillus* group, offering valuable insights into their roles in food fermentation, human health, and biotechnological applications. The findings provide a foundation for advancing the development of novel probiotics and functional foods, with potential benefits for human health and sustainable food production.

## Graphical Abstract

**Figure:**
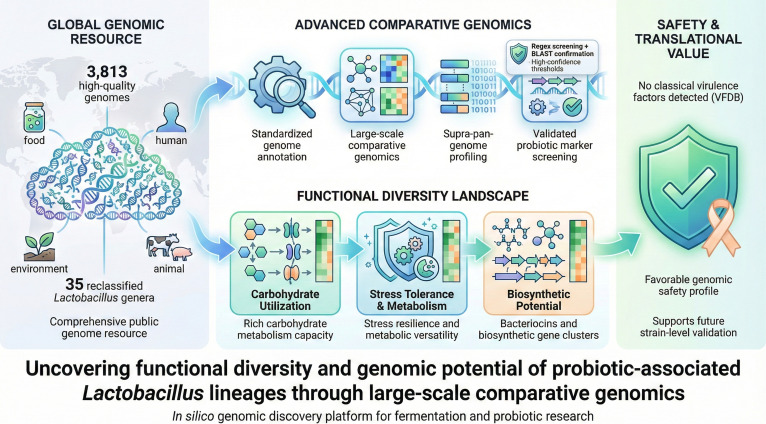

## Introduction

*Lactobacillus* is one of the most widely studied and ecologically significant genera of bacteria, primarily known for its roles in food fermentation and as probiotics. Historically, it encompassed over 250 species that are integral to the production of fermented foods like yogurt, cheese, and sauerkraut, as well as contributing to human gut health [1,2]. In 2020, a major taxonomic revision based on phylogenomic analysis led to the reclassification of many species into 25 distinct genera, including *Lactiplantibacillus*, *Lacticaseibacillus*, *Ligilactobacillus*, and *Fructilactobacillus*. This reorganization was based on more precise genetic information and allowed for a deeper understanding of their evolutionary relationships, ecological adaptations, and probiotic functionalities [3]. Despite the reclassification, the potential of these species remains largely unexplored. Each of these newly defined genera still contains species with significant probiotic properties and health benefits, yet there is a gap in understanding how the genomic traits of these species contribute to their functionality.

Previous research has shown that species like *Lactiplantibacillus plantarum*, *Lacticaseibacillus rhamnosus*, and *Lactobacillus gasseri* possess unique traits such as antimicrobial activity, biofilm formation, and the ability to degrade complex carbohydrates [4–6]. Furthermore, *Lactobacillus* species are known to produce bioactive compounds like organic acids, especially lactic acid, which exhibit antimicrobial and antifungal properties [7,8]. These metabolites not only inhibit harmful microbes but also promote human health through anti-inflammatory pathways [9] and support plant growth [10]. Furthermore, this bacterial group produces peptide-based bacteriocins, which have been shown to possess antimicrobial and antifungal properties [11]. Nutrient competition, particularly in the rapid uptake of carbon sources and micronutrients, plays a crucial role in outcompeting fungal spoilage organisms. These factors make *Lactobacillus* species not only functional probiotics but also effective competitors in complex microbial environments, contributing to their ecological success [12,13].

However, a comprehensive genomic analysis comparing these species, especially within the newly established genera [3], is essential for fully understanding their potential applications in food biotechnology, medicine, and health. In this study, we performed a comprehensive comparative genomic analysis of the former *Lactobacillus* genus (sensu lato) by examining high-quality genomes retrieved from National Center for Biotechnology Information (NCBI), including genome size, guanine-cytosine (GC) content, and assembly quality. We then analyzed the distribution of probiotic-associated marker genes, bacteriocin gene clusters, secondary metabolite biosynthetic gene clusters, and carbohydrate-active enzymes (CAZymes) to assess functional diversity. Finally, we visualized these traits using integrative plots to highlight species-specific strengths and conserved features. This research provides new insights into the genetic diversity of these species and their potential as functional probiotics in the food industry, as well as their roles in promoting human health through modulation of the gut microbiome.

## Materials and Methods

### Genome retrieval, inclusion criteria, and characterization

Genomic assemblies belonging to the former *Lactobacillus* genus, currently reclassified into multiple genera within the family *Lactobacillaceae*, were retrieved from the NCBI RefSeq and GenBank databases (accessed on 2025 September 18) using the NCBI Datasets command-line toolkit (v14.3.0). To ensure high-quality assemblies suitable for comparative genomic analysis, the following inclusion criteria were applied: (a) assemblies designated as complete genome, chromosome, scaffold, or contig; (b) exclusion of metagenome-assembled genomes (MAGs) and single-cell-derived assemblies; (c) assemblies with >200 contigs were removed unless assembly continuity was acceptable (contig N50 ≥ 50 kb); (d) quality filtering using CheckM, retaining only genomes with estimated completeness ≥95% and contamination ≤5%. Only species represented by ≥10 genomes after filtering were retained for downstream analyses to reduce bias from underrepresented taxa.

### Annotation and screening of probiotic functional markers

To identify probiotic-associated genes, all genomes were newly annotated using Prokka v1.14.6 [14] with standardized parameters (--kingdom Bacteria --gram-positive --rfam). The resulting GFF3 annotation files were parsed through a custom Python pipeline that screened the gene, product, and note fields of each coding sequence (CDS) against a curated set of regular expression patterns designed to capture established probiotic markers. The marker panel encompassed several functional categories. Genes related to bile salt hydrolase activity (*bsh*) were included [15], as well as the glutamate decarboxylase system (*gadA*, *gadB*, *gadC*), which contributes to γ-aminobutyric acid (GABA) production [16]. Adhesion and pilus formation were assessed through sortase genes (*srtA*, LPXTG-anchored proteins) [17] and sortase-dependent pili (*spaA*, *spaB*, *spaC*, pilin/fimbrial genes, or LPXTG motifs) [18]. Exopolysaccharide (EPS) biosynthesis was captured through scaffolding genes such as *eps*, *wzx/wzy*, *wzb/wzc*, *gtf*, *rfb*, *ugd*, and *gnd* [19,20]. Stress adaptation genes included molecular chaperones (*groEL*, *dnaK*, *clpC*) [21] and oxidative stress enzymes (*sodA*, *kat*, *ahpC*) [22,23]. Additional categories targeted vitamin and cofactor biosynthesis genes, including folate (*fol* operon), riboflavin (*rib* operon), and thiamine (*thi* operon) [24]. Acid and bile tolerance genes encompassed F_0_F_1_ adenosine triphosphatase (ATPase) subunits (*atpA-H*) [25] and the arginine deiminase system (*arcA-T*) [26]. Adhesion-associated surface proteins were also screened, including mucus-binding proteins (*mub*) [27], collagen and fibronectin-binding proteins (*cna*, *fbpA*) [28], and *S*-layer proteins (*slpA/B*) [29]. Antimicrobial functions were represented by bacteriocins, encompassing general descriptors [bacteriocin, lantibiotic, ribosomally synthesized and post-translationally modified peptides (RiPP)], plantaricin genes (*pln*) [30], and the reuterin system (*pdu*, *cob/cbi*) [31]. Carbohydrate utilization was evaluated through phosphotransferase systems (*pts*) [32] and sugar transporters (*lac*, *gal*, *man*, *rbs*) [33], while defense mechanisms included CRISPR-Cas loci (*cas* genes) [34]. To validate the regular expression (regex)-based probiotic marker gene (PMG) detection approach, we conducted additional analyses. Candidate PMG hits were initially identified using curated regex screening of Prokka annotations. The representative single-locus markers were sequence-confirmed. For each selected marker (*bsh*, *gadB*, *atpA*, *dnaK*, *srtA*, *clpP*, and *dltA*), candidate protein sequences were extracted from Prokka-generated protein FASTA files based on General Feature Format (GFF) coordinates and locus tags and queried using BLASTp against a curated reference database constructed from NCBI RefSeq protein sequences. Hits were considered confirmed using conservative criteria of ≥40% amino acid identity, ≥70% query coverage, and *E* value ≤1e^−5^. All regex patterns used are provided in Table S2.

### Identification of safety concern-associated genes

Genes associated with potential safety concerns were also examined. Antimicrobial resistance (AMR) genes were detected using AMRFinderPlus (NCBI) [35] on both assembled contigs and predicted proteins generated by Prokka. As an independent cross-check for acquired AMR determinants, contigs were screened with ABRicate against ResFinder, retaining hits with ≥50% coverage and ≥90% identity [36]. In addition, putative virulence factors (VFs) were screened with ABRicate against VFDB [37], applying stricter thresholds (≥70% coverage, ≥90% identity) to minimize spurious matches in lactic acid bacteria (LABs). ABRicate was run with default parameters after database initialization. To flag additional safety-relevant loci, we screened for biogenic amine (BA) decarboxylases, and associated transporters—histidine (*hdcA*), tyrosine (*tdc/tyrDC*), ornithine (*odc*), and lysine (*cadA*) decarboxylases, as well as *tyrP* [37,38], and for hemolysin and cytolysin terms (e.g., *hly* for hemolysin and *cyl* for operon aliases) [39,40].

### Prediction and comparative analysis of BGCs

Bacteriocin-encoding genes were identified using the BAGEL4 pipeline [41], which integrates hidden Markov model (HMM)-based searches with curated bacteriocin databases. All genomes were annotated de novo before screening, and predicted open reading frames (ORFs) were subjected to BAGEL4’s motif-based detection to capture both structural peptides and associated biosynthetic, immunity, and transport genes. To ensure consistency, overlapping or redundant hits were consolidated, and bacteriocins were classified into subclasses (class I-lantibiotics, class IIa-pediocin-like, class IIb-two-peptide, class IIc-circular, class IId-linear unmodified, class III-large proteins, and others) using a combination of BAGEL4 annotations and manual curation informed by the BAGEL4 reference database and published literature. Presence–absence matrices were generated to evaluate distribution patterns across species and strains, while gene counts were aggregated at both strain and species levels to assess abundance. Moreover, secondary metabolite BGCs were also identified using antiSMASH v8.0 by detecting known cluster types, ClusterFinder, and comparison against the MIBiG database to annotate putative BGCs.

### CAZyme annotation among former *Lactobacillus*

CAZyme families were identified using the standalone dbCAN3 pipeline [42] in DIAMOND-only mode against the CAZy database (last update July 2025) [43]. For each predicted proteome, we executed run_dbcan <proteins.faa> protein --tools diamond, which applies DIAMOND searches against curated CAZy entries. Although dbCAN3 supports multiple approaches (e.g., HMMER versus dbCAN HMMdb, DIAMOND versus CAZy, and HMMER versus dbCAN-sub), the analysis was restricted to the DIAMOND versus CAZy workflow. Unless otherwise specified, DIAMOND hits were filtered using dbCAN’s recommended thresholds for the DIAMOND workflow (*E* value < 1 × 10^−102^; top hit per query, *k* = 1). Thresholds for HMMER and Hotpep were not applied, as only the DIAMOND-based approach was used in this study. DIAMOND v2.1 was employed as the sequence aligner, offering BLAST-compatible protein alignments optimized for high-throughput CAZyme screening. CAZyme family definitions were derived from the dbCAN resource and the expert-curated CAZy database.

### Supra-pan-genome analysis across former *Lactobacillus*

**Fig. 1.**
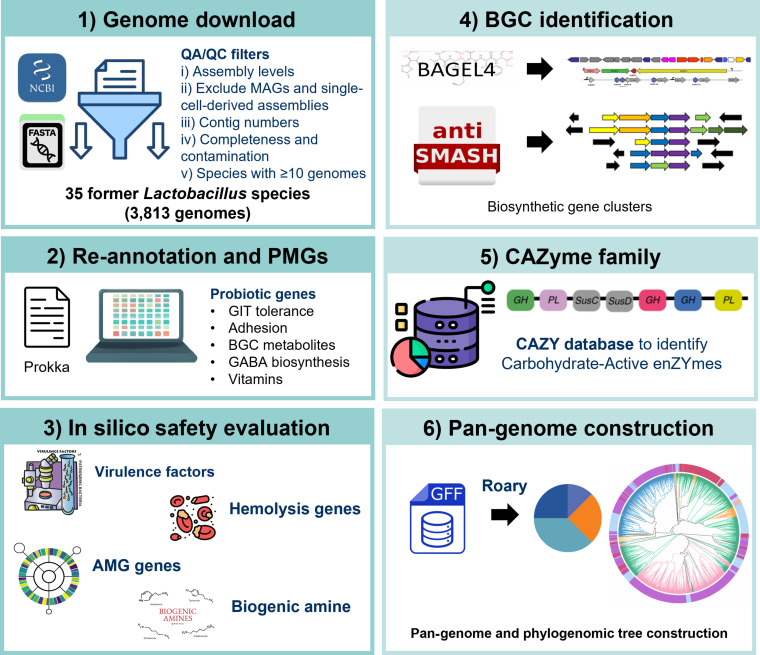
Workflow for data curation and subsequent downstream analysis, including genome download, re-annotation, safety evaluation, BGC identification, CAZyme family identification, and pan-genome construction.

The supra-pan-genome analysis of all 3,813 former *Lactobacillus* species was constructed using the Roary pipeline [44], where proteins with ≥95% amino acid sequence identity were grouped into the same orthologous family. A maximum-likelihood phylogenomic tree was generated using FastTree v2.1 [45] and subsequently annotated and visualized using the Interactive Tree of Life (iTOL) v8. This analysis spans multiple genera; the resulting gene set represents a family-level gene repertoire rather than a classical species-level pan-genome. The absence of core genes is therefore expected. The overview of this study is summarized in Fig. 1.

## Results

### Genome characterization of the former *Lactobacillus* genus

**Fig. 2.**
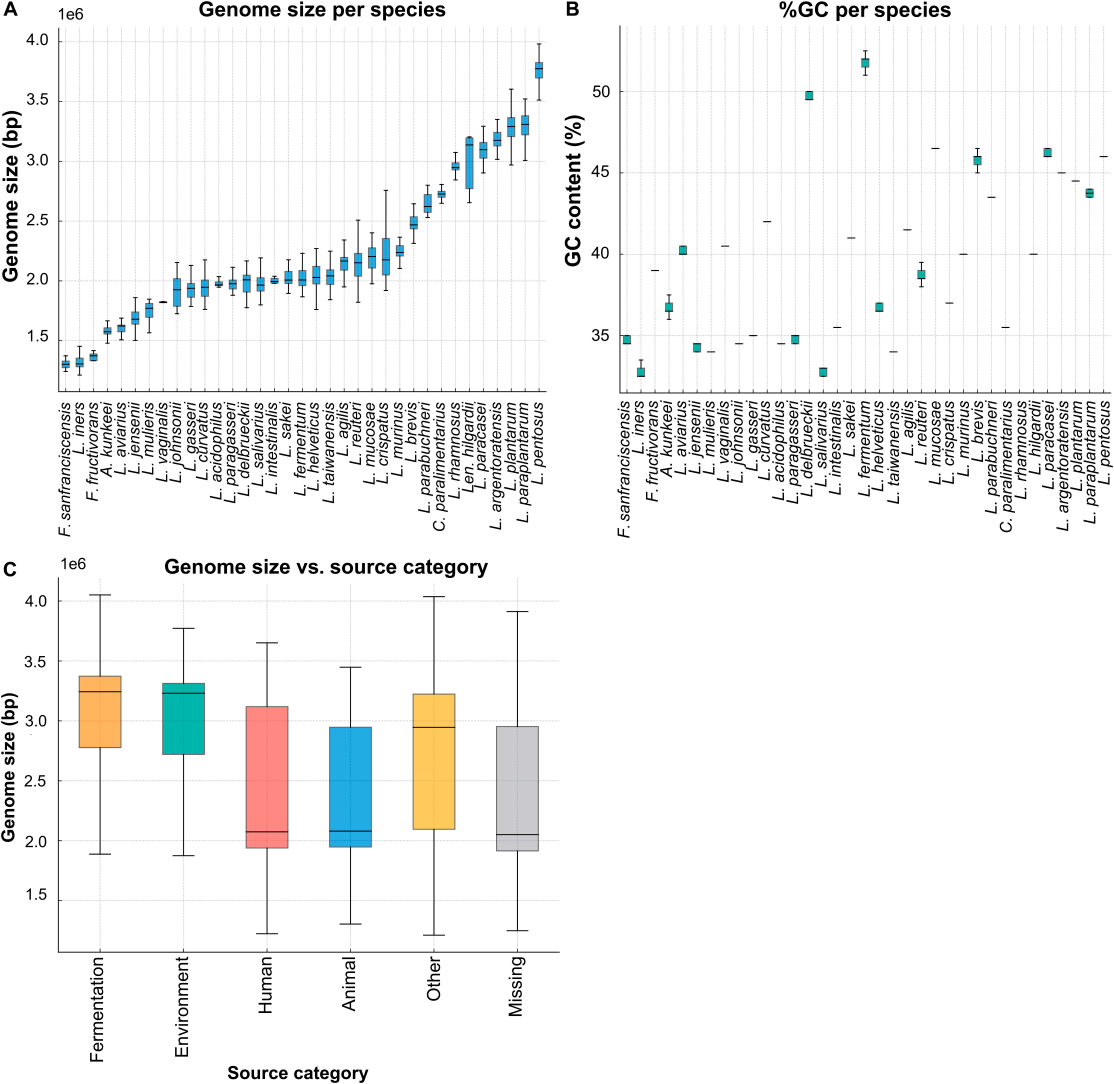
Genome characteristics and ecological origins of the former *Lactobacillus* genus. (A) Genome size distributions per species shown as boxplots with individual genomes overlaid as jittered points. Species labels are abbreviated at the genus level. (B) GC content (%) per species shown as boxplots with jittered points. (C) Genome size compared across isolation source categories, shown as boxplots with jittered points.

A total of 3,813 high-quality genomes representing 35 species of the former *Lactobacillus* genus were retained after applying strict quality control filters. Species representation was highly uneven, with some taxa represented by extensive sequencing efforts and others by only a handful of assemblies. The largest dataset was available for *L. plantarum* (*n* = 1,233 genomes), followed by *L. rhamnosus* (*n* = 331), *Limosilactobacillus reuteri* (*n* = 310), *Ligilactobacillus salivarius* (*n* = 240), and *L. gasseri* (*n* = 223). In contrast, several lineages, such as *Lentilactobacillus hilgardii* and *Lactobacillus intestinalis*, were represented by only 20 genomes each. Moreover, genome size exhibited substantial variation across the dataset, ranging from 1.21 Mb to 4.05 Mb (Fig. 2A). *L. plantarum* and *L. pentosus* carried the largest genome sizes (>3.0 Mb on average), whereas vaginal species such as *L. iners*, *Lactobacillus crispatus*, and *L. gasseri* exhibited compact genomes (1.3 to 2.0 Mb). GC content ranged from 32.5% to 53.0%, with most species clustering between 34% and 47% (Fig. 2B). *Fructilactobacillus fructivorans* showed the highest GC content (>50%), while *L. iners* consistently harbored the lowest values (approximately 32% to 33%). Assembly contiguity ranged from complete chromosomes to highly fragmented drafts (up to 811 contigs), with median contig counts generally <50 (Fig. S1A). BioSample metadata showed genomes from fermentation, environment, human, animal, other, and missing categories (Fig. S1B). Fermentation-associated isolates (*n* = 700) and human-derived genomes (*n* = 833) form major fractions, with a substantial metadata gap (*n* = 994). Comparisons of genome size by isolation source (Fig. 2C) revealed slightly larger median genomes in fermentation- or environment-derived isolates relative to host-associated lineages. The source-proportion heatmap (Fig. S1C) highlighted niche biases, such as *Fructilactobacillus sanfranciscensis* in sourdough, *Apilactobacillus kunkeei* in honeybees, and *L. rhamnosus* in human-associated samples. The genome characteristic summary was presented in Table S1.

### Comparative genomic distribution of probiotic functional categories

**Fig. 3.**
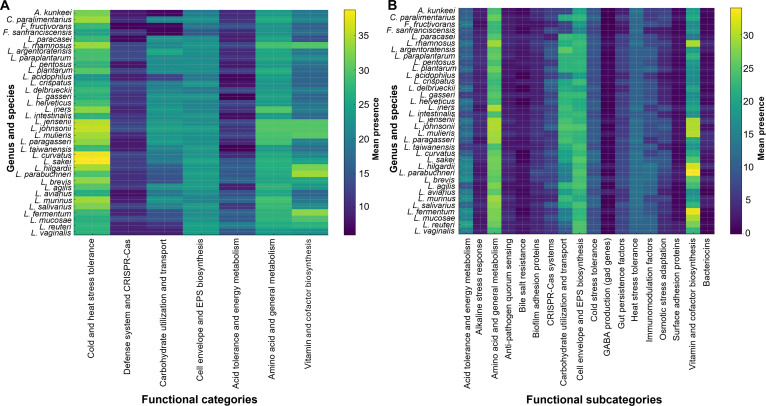
Heatmap of probiotic functional categories across former *Lactobacillus* species. (A) Collapsed heatmap showing 7 major categories: Cold and heat stress tolerance, defense systems and CRISPR-Cas, carbohydrate utilization and transport, cell envelope and EPS biosynthesis, acid tolerance and energy metabolism, amino acid and general metabolism, vitamin and cofactor biosynthesis, and bacteriocin and antimicrobial peptide production. (B) Detailed heatmap displaying subcategories, including adhesion proteins, bile salt resistance, GABA production, immunomodulation factors, and others.

**Fig. 4.**
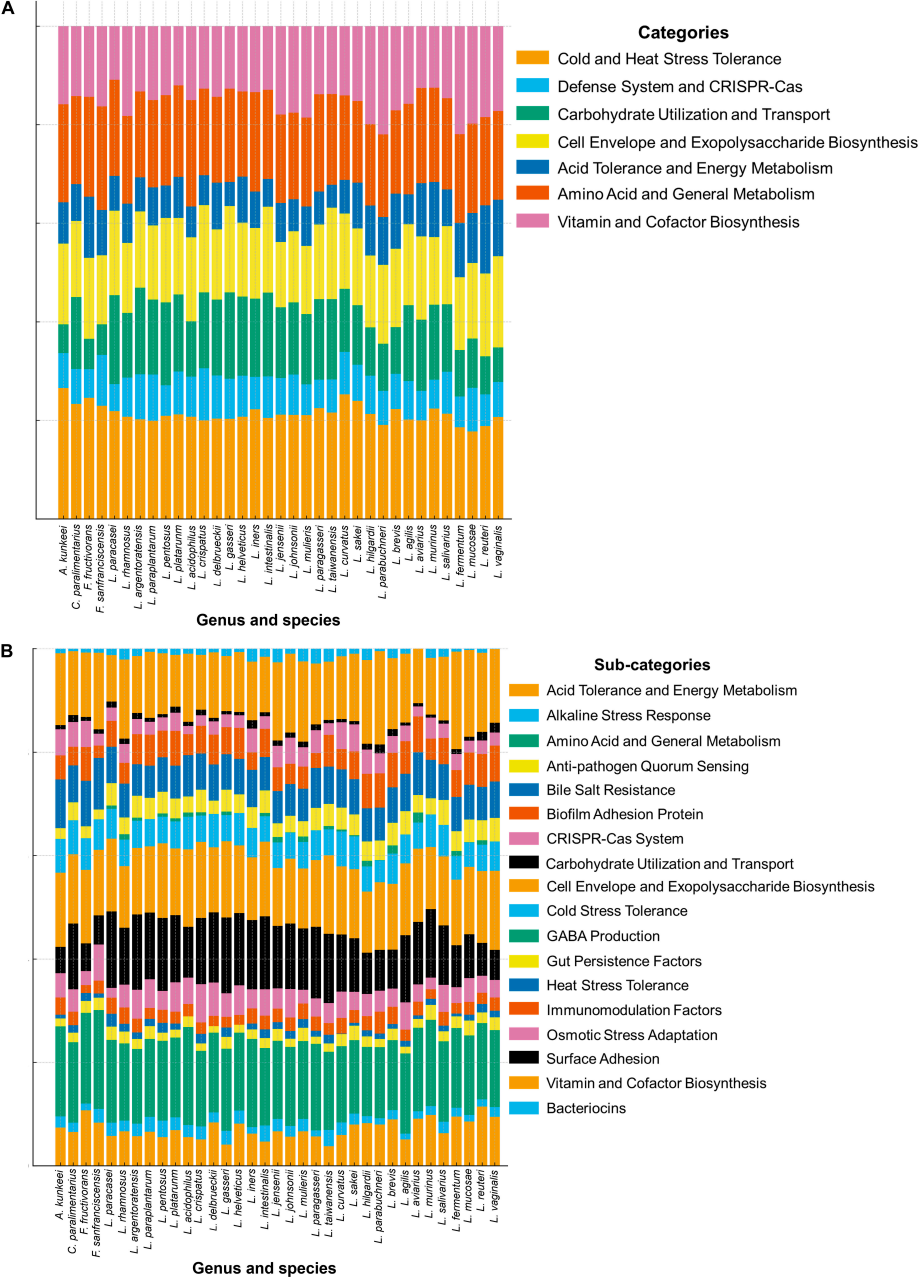
Comparative enrichment of probiotic-associated functional categories among 35 species in the former *Lactobacillus* genus. (A) Relative enrichment of 7 main functional categories. (B) Detailed subcategory distribution showing species-level variation in traits. Each bar represents normalized proportions of functional genes within each species.

The distribution of PMGs across the 35 LAB species analyzed in this study is summarized in Figs. 3 and 4 and Table 1. The collapsed and detailed heatmaps revealed both conserved functions and species-specific traits (Fig. 3). At the category level (Fig. 3A), functions related to stress tolerance (acid, heat, cold), amino acid and energy metabolism, and vitamin/cofactor biosynthesis were broadly conserved. Carbohydrate utilization and transport, amino acid and general metabolism, and cell envelope and EPS biosynthesis were enriched in taxa such as *L. crispatus*, *L. pentosus*, and *L. rhamnosus*. Vitamin and cofactor biosynthesis was mostly found in *Lentilactobacillus parabuchneri*, *Limosilactobacillus fermentum*, and *L. hilgardii.* At the subcategory level (Fig. 3B), conserved functions included amino acid and general metabolism, carbohydrate utilization and transport, and cell envelope and EPS biosynthesis, which were present across most species. *L. paracasei*, *Lactiplantibacillus paraplantarum*, *L. plantarum*, *L. pentosus*, and *L. rhamnosus* showed enrichment in amino acid metabolism and carbohydrate pathways. Fermentation-associated taxa such as *Companilactobacillus paralimentarius*, *L. crispatus*, and *L. pentosus* were enriched in carbohydrate metabolism. EPS biosynthesis and surface adhesion proteins were abundant in several species, such as *L. crispatus*, *L. pentosus*, *L. intestinalis*, and *Latilactobacillus sakei*. Vitamin and cofactor biosynthesis genes were most prevalent in fermentation-related lineages such as *L. plantarum*, *Lactiplantibacillus argentoratensis*, *L. paraplantarum*, *L. pentosus*, *L. sakei*, *L. hilgardii*, and *L. parabuchneri*. Other categories, including alkaline stress response, anti-pathogen quorum sensing, bile salt resistance, biofilm adhesion proteins, CRISPR-Cas systems, cold stress tolerance, GABA production, gut persistence factors, surface adhesion proteins, and bacteriocin-related genes, were distributed in strain-specific patterns.

**Table 1.** Functional strengths, unique traits, and potential traits of LAB species summarized from the literature and findings in this study

Species	Main strengths	Unique traits	Potential traits	Notes	References
*Apilactobacillus kunkeei*	Stress tolerance; adhesion traits	Biofilm and surface adhesion proteins	Bee probiotic; honeybee gut health; adaptation to hive environment	Ecological specialist, niche adaptation	[94]
*Lactiplantibacillus plantarum*	Broad stress tolerance; versatile carbohydrate metabolism	GABA production (*gad* operon); adhesion proteins	Functional foods with GABA enrichment; gut microbiome resilience; fermented vegetables	Well-established LAB, multifunctional probiotic	[56,57]
*Lactiplantibacillus pentosus*	Carbohydrate utilization; acid tolerance	Plant-associated fermentation genes	Olive and vegetable fermentation; potential gut probiotic	Common in fermented plant foods	[95]
*Lactiplantibacillus paraplantarum*	Stress tolerance; sugar transport systems	Close relative of *L. plantarum*	Vegetable fermentation; gut probiotic potential	Functionally similar to *L. plantarum*	[96]
*Lactiplantibacillus argentoratensis*	Acid and carbohydrate metabolism	Plant-associated genes	Cereal fermentation; starter culture applications	Less studied LAB, related to *L. plantarum* complex	[97]
*Lacticaseibacillus rhamnosus*	EPS biosynthesis; stress resilience	Bile salt resistance; host adhesion	Gut probiotics; dairy fermentation; gastrointestinal survival	Widely used commercial probiotic strain	[58]
*Lacticaseibacillus paracasei*	Carbohydrate metabolism; stress response	Dairy-associated adaptation	Cheese and yogurt fermentation; probiotic dairy supplements	Closely related to *L. rhamnosus*	[98,99]
*Latilactobacillus curvatus*	Protein and amino acid metabolism	Meat fermentation adaptation	Starter in meat fermentation (salami, sausages)	Traditional food fermentation LAB	[100]
*Latilactobacillus sakei*	Cold stress tolerance; carbohydrate metabolism	Nitrite reduction capacity	Meat preservation; cold-adapted probiotic candidate	Key starter in meat fermentation	[101]
*Lentilactobacillus hilgardii*	EPS biosynthesis; carbohydrate utilization	Wine and beverage fermentation genes	Wine and vegetable fermentation; potential starter culture	Associated with wine and sauerkraut	[102]
*Lentilactobacillus parabuchneri*	Amino acid metabolism; stress response	Histamine production genes	Cheese ripening; caution in probiotic use	Known as spoilage-associated but genomically diverse	[103]
*Levilactobacillus brevis*	Defense traits; carbohydrate metabolism	CRISPR-Cas systems; immunomodulation factors	Gut probiotic; plant-based fermentations	Adaptive LAB with flexible metabolism	[104]
*Fructilactobacillus sanfranciscensis*	Carbohydrate metabolism; acid tolerance	Sourdough-adapted enzymes	Sourdough starter; bakery fermentation	Classical sourdough species	[54,105]
*Fructilactobacillus fructivorans*	Stress tolerance; carbohydrate metabolism	Fruit-associated fermentation traits	Fruit and wine fermentation; potential niche probiotic	Common in fruit fermentations	[106]
*Companilactobacillus paralimentarius*	Carbohydrate metabolism; EPS biosynthesis	Bread-associated functions	Sourdough and cereal fermentation	Important in bakery ecosystems	[55]
*Lactobacillus acidophilus*	Acid tolerance; bile resistance	Adhesion proteins; immunomodulation	Probiotic supplements; dairy fermentation	Well-studied gut commensal	[107]
*Lactobacillus crispatus*	Vaginal colonization; lactic acid production	Adhesion and mucin-binding proteins	Vaginal probiotics; women’s health	Key species in vaginal microbiota	[59]
*Lactobacillus gasseri*	Stress tolerance; acid production	Bacteriocin genes; adhesion traits	Probiotic supplements; gut health	Frequently found in gut microbiome	[108]
*Lactobacillus johnsonii*	Acid tolerance; carbohydrate metabolism	Host-associated adhesion proteins	Gut probiotics; functional dairy	Prominent gut-associated LAB	[109]

The relative enrichment of probiotic-associated gene categories across 35 species in the former *Lactobacillus* genus is shown in Fig. 4, which was also correlated with the category distribution. Conserved functional categories such as stress tolerance, amino acid and energy metabolism, cell envelope and EPS biosynthesis, and vitamin and cofactor biosynthesis were consistently represented across nearly all taxa (Fig. 4A). Carbohydrate utilization and transport were more abundant in species such as *Lactobacillus acidophilus*, *L. crispatus*, *L. plantarum*, and *L. pentosus*, while defense systems and CRISPR-Cas and acid tolerance and energy metabolism were less enriched among all species. At the subcategory level (Fig. 4B), core functional categories, including amino acid and general metabolism, carbohydrate utilization, cell envelope and EPS biosynthesis, and vitamin and cofactor biosynthesis, were highly enriched and conserved across all species. In addition, several subcategories exhibited broad distribution with moderate enrichment, such as acid tolerance and energy metabolism, biofilm adhesion proteins, CRISPR-Cas systems, cold stress tolerance, gut persistence factors, heat stress tolerance, immunomodulation factors, and osmotic stress adaptation. In contrast, functions related to anti-pathogen quorum sensing, bile salt resistance, GABA production, surface adhesion proteins, and bacteriocin biosynthesis displayed more restricted, species- or strain-specific enrichment patterns. The validation of example genes was performed by sequence-based confirmation using BLASTp for a subset of PMGs. The result showed high concordance between regex-based screening and BLAST confirmation for most tested markers, including 100% concordance for *gadB*, *atpA*, *clpP*, and *dltA*, and >97% concordance for *bsh*, *dnaK*, and *srtA*. Therefore, BLAST-based confirmation was retained for this marker subset (Table S3). The specific marker genes and regex patterns employed are detailed in Table S2.

Functional comparison of 35 species belonging to the former *Lactobacillus* genus revealed distinct patterns of metabolic capability, ecological adaptation, and potential traits (Table 1). Core probiotic-associated functions, including stress tolerance, carbohydrate metabolism, and acid resistance, were commonly detected across nearly all taxa. Species within the *Lactiplantibacillus* group, such as *L. plantarum*, *L. pentosus*, and *L. paraplantarum*, showed broad metabolic versatility and high adaptability to diverse environments. *L. plantarum* exhibited unique GABA production genes, while *L. pentosus* and *L. argentoratensis* were characterized by plant-associated fermentation genes. Members of the *Lacticaseibacillus* group, including *L. rhamnosus* and *L. paracasei*, possessed strong EPS biosynthesis, bile salt resistance, and adhesion-related functions. Meat-associated species, such as *Latilactobacillus curvatus* and *L. sakei*, showed enhanced amino acid metabolism and cold stress tolerance. *L. hilgardii* and *L. parabuchneri* carried genes involved in wine and vegetable fermentations. Other genera exhibited niche-specific features. For example, *F. sanfranciscensis* and *C. paralimentarius* contained sourdough-associated carbohydrate metabolism genes. *A. kunkeei* showed biofilm and surface adhesion proteins associated with adaptation to honeybee habitats. *Levilactobacillus brevis* contained CRISPR-Cas defense elements and immunomodulation factors. Among traditional *Lactobacillus* members, *L. acidophilus*, *L. gasseri*, *L. crispatus*, and *L. johnsonii* displayed acid and bile tolerance, adhesion, and immune-related functional genes consistent with probiotic characteristics.

### Diversity and distribution of bacteriocins across the former *Lactobacillus* genus

**Fig. 5.**
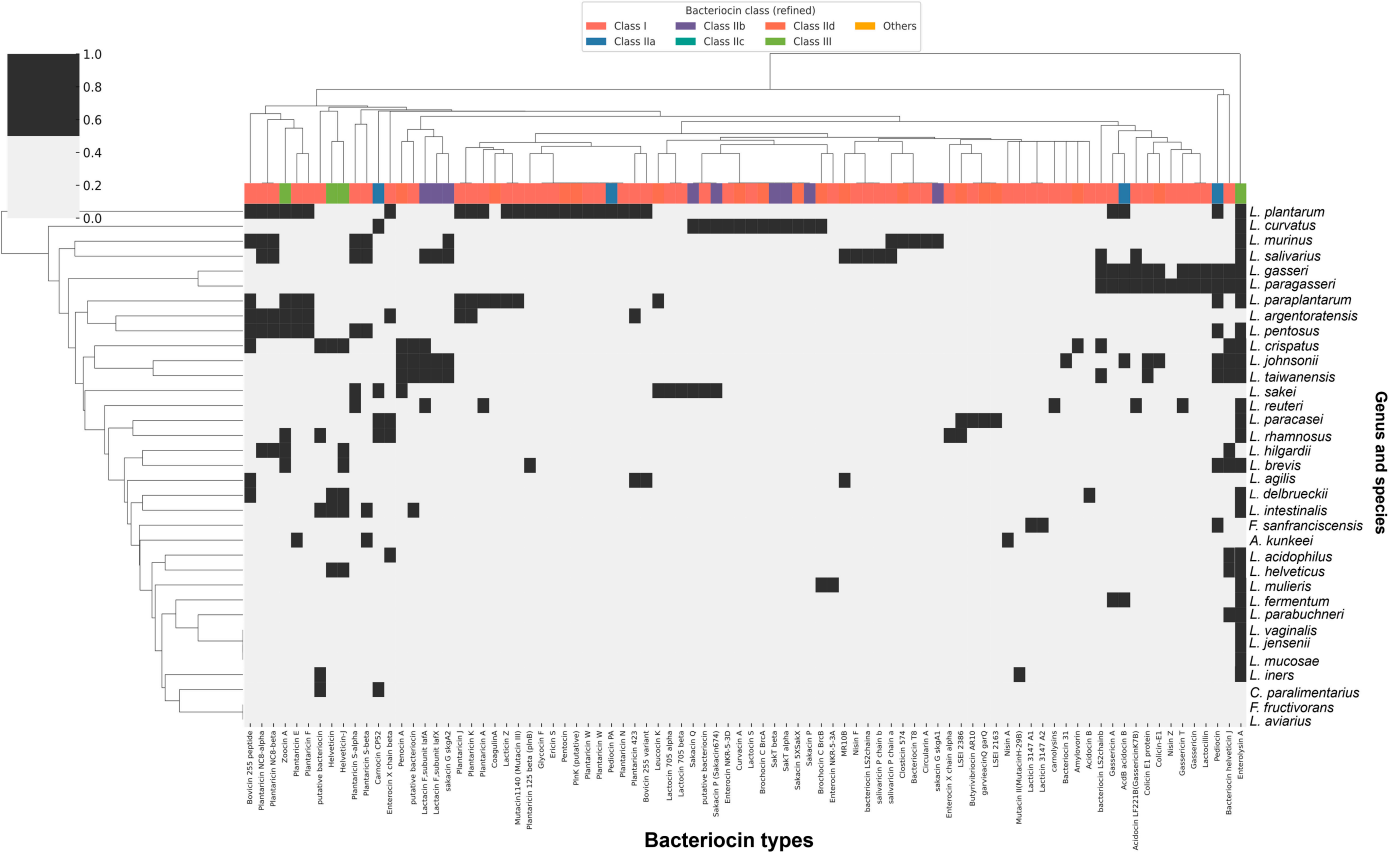
Presence or absence heatmap of bacteriocin genes among former *Lactobacillus* species. Heatmap showing the distribution of bacteriocin-encoding genes across 35 species within the former *Lactobacillus* genus. Colored cells indicate the presence of specific bacteriocin gene types, while blank cells represent absence, highlighting interspecies variation in bacteriocin diversity.

A total of 3,813 genomes representing 35 species of the former *Lactobacillus* genus were examined to identify bacteriocin biosynthetic gene clusters. The presence or absence of heatmap revealed a highly variable distribution pattern across species, with some taxa encoding multiple bacteriocin operons and others lacking detectable bacteriocin genes (Fig. 5). *L. plantarum*, *L. rhamnosus*, *L. salivarius*, *L. reuteri*, and *L. gasseri* exhibited the greatest diversity of bacteriocin-encoding genes, encompassing multiple pediocin-like and class II bacteriocin loci. In contrast, species such as *F. sanfranciscensis*, *A. kunkeei*, and *C. paralimentarius* contained only a limited number of bacteriocin genes, primarily belonging to classes IIa, IIb, or III. No bacteriocin clusters were detected in *Ligilactobacillus aviarius* and *F. fructivorans*. Within *L. plantarum*, considerable strain-level variation was observed, with several strains harboring multiple plantaricin operons, including *plnE-F*, *plnJ-K*, *plnNC8α-β*, and *plnSα-β*. The reference strain *L. plantarum* WCFS1 possessed more than 5 distinct loci. In addition, *L. rhamnosus* strains, including strain GG, encoded bacteriocin clusters such as enterocin X, carnocin CP52, and LSEI_2386. Moreover, enterolysin A was widely distributed across nearly all species, whereas other bacteriocins, such as lacticin Z, pentocin, nisin A and F, and curvacin A, were detected in a species-specific manner.

**Fig. 6.**
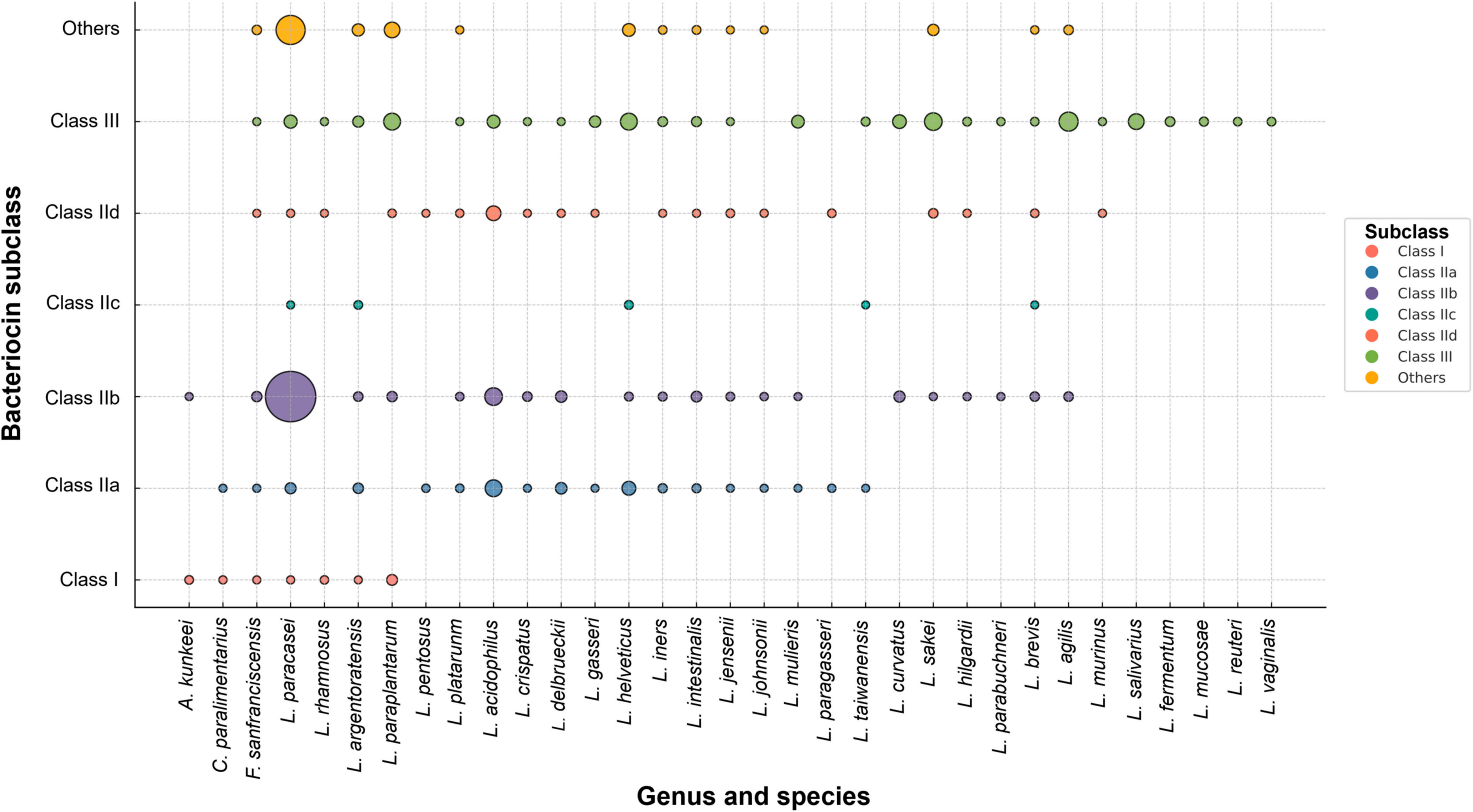
Distribution of bacteriocin subclasses among former *Lactobacillus* species. Bubble plot showing the presence and diversity of bacteriocin subclasses (classes I to III and others) across the bacteriocin presented 33 species. Bubble size represents the number of bacteriocin types identified within each subclass for each species.

The subclass-level distribution of bacteriocins across former *Lactobacillus* species identified 6 bacteriocin subclasses, including class I (lantibiotics), class IIa–IId (small, heat-stable peptides), class III (large, heat-labile proteins), and unclassified bacteriocins grouped as others (Fig. 6). *L. plantarum* contained the highest number and diversity of bacteriocin subclasses, predominantly class IIb peptides, followed by *L. paraplantarum* and *L. paragasseri*, which also carried multiple class IIa and IIb genes. *L. rhamnosus*, *L. paracasei*, and *L. salivarius* harbored several class II subclasses, whereas *L. helveticus*, *L. jensenii*, *L. parabuchneri*, *L. mucosae*, and *L. vaginalis* contained only class III bacteriocins. Class I lantibiotic bacteriocins were occasionally detected in *A. kunkeei*, *F. sanfranciscensis*, *L. plantarum*, *L. paraplantarum*, *L. iners*, *L. paragasseri*, and *L. salivarius*. Among these, *A. kunkeei* and *F. sanfranciscensis* exhibited only a limited presence of class IIa or class III bacteriocins.

**Fig. 7.**
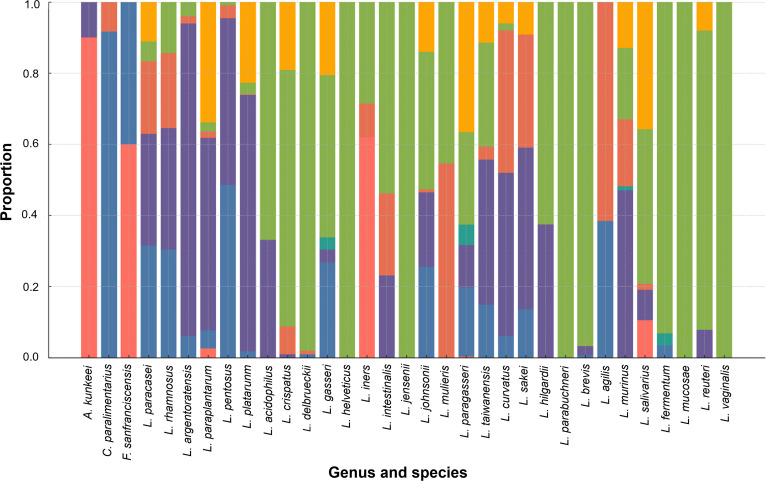
Relative class composition of bacteriocins across 35 former *Lactobacillus* species. Each stacked bar represents a species, partitioned by the proportional contribution of bacteriocin subclasses.

Moreover, the relative composition of bacteriocin subclasses was analyzed across 35 species of the former *Lactobacillus* genus (Fig. 7). The results show clear interspecies variation in the types and relative abundance of bacteriocin subclasses. Importantly, *L. plantarum*, *L. paraplantarum*, and *L. pentosus* displayed the broadest diversity, containing multiple subclasses, predominantly class IIb, followed by class IIa, class IId, and class III, indicating the presence of complex bacteriocin repertoires. Similarly, *L. rhamnosus* and *L. paracasei* carried mixed profiles, largely represented by class IIa and class IIb peptides. Species *L. reuteri*, *L. mucosae*, and *L. fermentum* were characterized by high proportions of class III bacteriocins, consistent with the dominance of large, heat-labile antimicrobial proteins in these taxa. *L. crispatus*, *L. gasseri*, *L. vaginalis*, and *L. helveticus* also showed a marked presence of class III genes, while *L. curvatus* and *L. sakei* were mainly associated with class IIa types. In addition, species such as *A. kunkeei* and *F. sanfranciscensis* showed narrow bacteriocin compositions dominated by class I, while *C. paralimentarius* showed highly in class IIa. Class II bacteriocins constituted the most prevalent subclasses across species, followed by class III, while class I and unclassified bacteriocins were relatively infrequent and species-specific.

Following the subclass composition analysis, the species-level summary of bacteriocin abundance and diversity is presented in Table 2. *L. plantarum* exhibited the highest bacteriocin abundance, with 95.1% of genomes containing bacteriocin loci and 27 unique types identified, mainly plantaricin F, plantaricin E, and plantaricin J. Species *L. rhamnosus* and *L. salivarius* showed similarly high proportions, each with 100% of genomes carrying multiple bacteriocin types, including enterocin X, carnocin CP52, salivaricin P, and enterolysin A. Moreover, *L. gasseri*, *L. reuteri*, and *L. crispatus* also demonstrated strong bacteriocin presence, with abundant helveticin J, pediocin, and enterolysin A loci. Species such as *L. helveticus*, *L. acidophilus*, *L. paragasseri*, and *L. pentosus* exhibited moderate abundance, characterized by fewer unique bacteriocin types but consistent detection across strains. In contrast, *L. curvatus*, *L. sakei*, *L. jensenii*, *L. iners*, and *A. kunkeei* displayed limited bacteriocin occurrence, primarily enterolysin A, nisin A, or unclassified peptides. *L. aviarius* and *F. fructivorans* showed no detectable bacteriocin clusters. At the strain level, *L. plantarum* WCFS1 encoded multiple pln operons, *L. rhamnosus* GG contained enterocin X, carnocin CP52, and LSEI_2386, while *L. salivarius* UCC118 possessed salivaricin P and salivaricin B. *L. gasseri* ATCC 33323 carried helveticin J and enterolysin A, and *A. kunkeei* strains harbored nisin-like lantibiotics.

**Table 2.** Species-level summary of bacteriocin abundance and diversity in Lactobacillaceae

Species	Genome numbers	% with ≥1 bacteriocin	Total abundance	Unique types	Dominant bacteriocins	Notes
*L. plantarum*	1233	95.1%	5118	27	Plantaricin F; Plantaricin E; Plantaricin J	Strong producer
*L. rhamnosus*	331	100.0%	1091	7	Enterocin X β; Carnocin CP52; LSEI 2386	Strong producer
*L. salivarius*	240	100.0%	761	16	Enterolysin A; Enterolysin A; Salivaricin P	Strong producer
*L. gasseri*	223	100.0%	725	13	Helveticin J; Pediocin; Enterolysin A	Strong producer
*L. reuteri*	310	89.0%	535	8	Enterolysin A; Enterolysin A; Acidocin LF221B	Strong producer
*L. crispatus*	112	100.0%	514	12	Enterolysin A; Helveticin J; Helveticin J	Strong producer
*L. paragasseri*	71	100.0%	350	14	Helveticin J; Pediocin; LS2 bacteriocin	Moderate
*L. acidophilus*	93	100.0%	278	3	Helveticin J; Enterolysin A; Enterocin X β	Moderate
*L. helveticus*	93	100.0%	277	4	Helveticin J; Enterolysin A; Helveticin J	Moderate
*L. pentosus*	111	97.3%	218	10	Pediocin; Plantaricin NC8 α; Plantaricin NC8 β	Moderate
*L. taiwanensis*	29	100.0%	194	11	Pediocin; Helveticin J; Lactacin F lafA	Moderate
*L. brevis*	120	87.5%	153	7	Enterolysin A; Enterolysin A; Helveticin J	Moderate
*L. johnsonii*	35	100.0%	129	13	Helveticin J; Pediocin; Lactacin F lafA	Moderate
*L. paraplantarum*	24	100.0%	118	13	Plantaricin F; Plantaricin E; Plantaricin A	Moderate
*L. delbrueckii*	60	100.0%	104	5	Enterolysin A; Helveticin J; Bovicin 255	Moderate
*L. murinus*	18	100.0%	85	12	Enterolysin A; Closticin 574; Sakacin G	Moderate
*L. paracasei*	17	100.0%	54	7	Enterocin X β; Carnocin CP52; LSEI 2386	Moderate
*L. curvatus*	67	32.8%	50	14	Putative bacteriocin; Sakacin Q; Sakacin P	Low/none
*L. argentoratensis*	33	90.9%	50	10	Enterocin X β; Plantaricin E; Plantaricin K	Low/none
*L. jensenii*	63	55.6%	47	2	Enterolysin A; Enterolysin A	Low/none
*L. intestinalis*	10	100.0%	39	6	Helveticin J; Enterolysin A; Plantaricin S β	Low/none
*L. parabuchneri*	27	100.0%	30	3	Helveticin J; Enterolysin A; Enterolysin A	Low/none
*L. fermentum*	74	33.8%	29	4	Enterolysin A; Enterolysin A; Acidocin B	Low/none
*L. agilis*	37	54.1%	26	4	Plantaricin 423; MR10B; Bovicin 255	Low/none
*L. sakei*	15	73.3%	22	9	Putative bacteriocin; Carnocin CP52; Plantaricin S α	Low/none
*L. iners*	73	24.7%	21	3	Mutacin II; Enterolysin A; Putative bacteriocin	Low/none
*A. kunkeei*	106	17.9%	20	3	Nisin A; Plantaricin S β; Plantaricin E	Low/none
*L. vaginalis*	20	90.0%	20	2	Enterolysin A; Enterolysin A	Low/none
*L. hilgardii*	10	70.0%	16	6	Helveticin J; Zoocin A; Plantaricin NC8 α	Low/none
*L. mucosae*	16	75.0%	15	2	Enterolysin A; Enterolysin A	Low/none
*C. paralimentarius*	12	91.7%	12	2	Carnocin CP52; Putative bacteriocin	Low/none
*L. mulieris*	43	20.9%	11	3	Enterolysin A; Enterocin NKR-5-3A; Brochocin C	Low/none
*F. sanfranciscensis*	51	13.7%	10	3	Pediocin; Lacticin 3147 A2; Lacticin 3147 A1	Low/none
*L. aviarius*	23	0.0%	0	0	None	Low/none
*F. fructivorans*	13	0.0%	0	0	None	Low/none

### Distribution of biosynthetic gene clusters (BGCs) in former *Lactobacillus* species

**Fig. 8.**
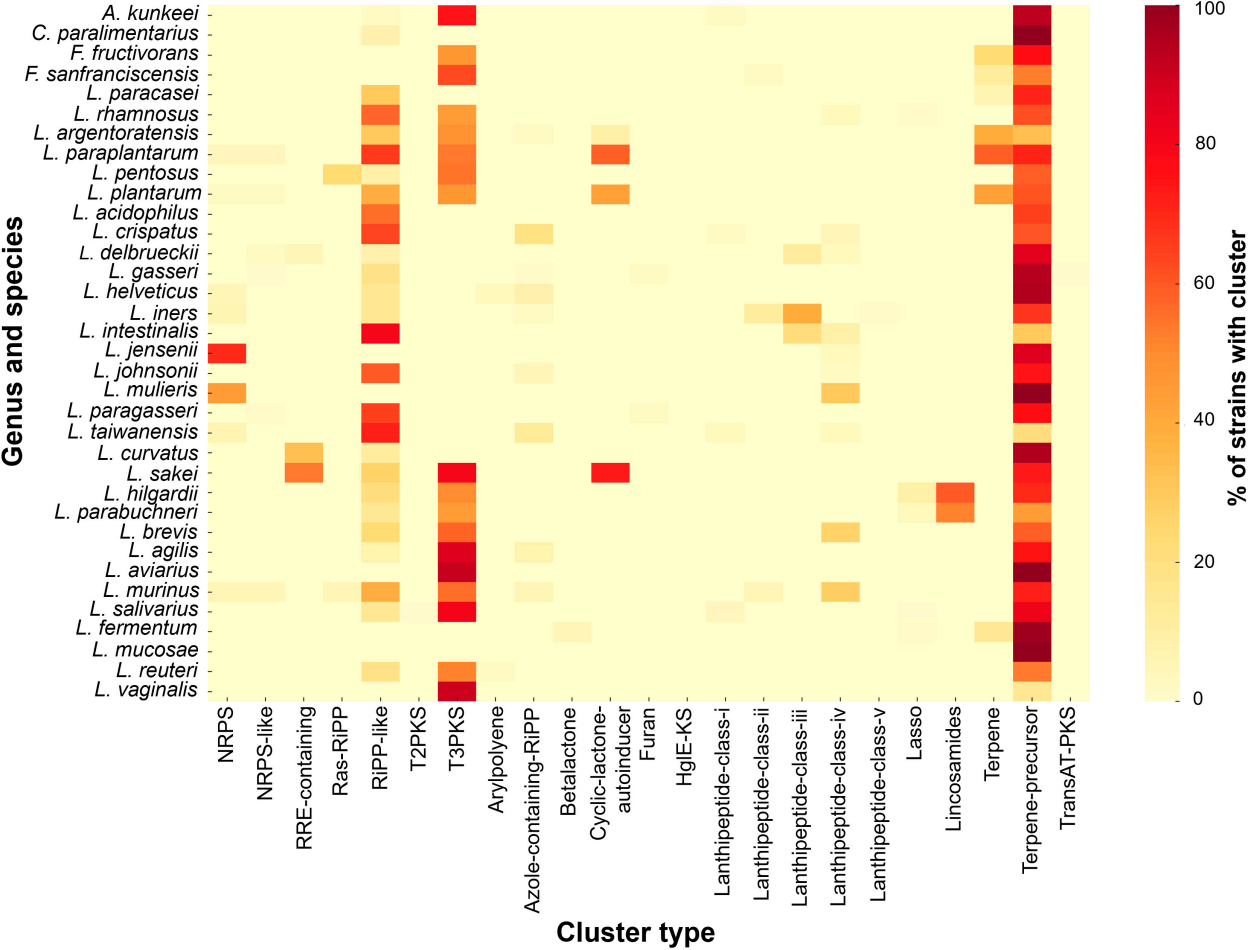
Percentage presence of all BGC types across former *Lactobacillus* species. Heatmap showing the proportion of genomes within each species that harbor a given BGC type, as predicted by antiSMASH v8.0.

Genome mining of the former *Lactobacillus* taxa identified a wide range of BGCs with uneven distribution across species (Fig. 8). Among all detected cluster types, terpene and terpene-precursor clusters were the most abundant, followed by RiPPs, RaS-RiPPs, and lanthipeptides. In contrast, nonribosomal peptide synthetases (NRPSs), polyketide synthases (PKSs), and hybrid NRPS/PKS loci were detected at low frequencies. At the genus level, *Lactiplantibacillus* exhibited the highest abundance and diversity of BGCs, with a total of 500 RiPP-like clusters, 566 terpene clusters, and 848 terpene-precursor clusters, as well as rare NRPS and hybrid loci. *Lacticaseibacillus* contained 195 RiPP-like clusters and 12 lanthipeptide class IV clusters, while *Fructilactobacillus* was dominated by terpene-related clusters, including 9 terpene and 45 terpene-precursor loci. *A. kunkeei* consistently carried terpene-precursor clusters (*n* = 98), with occasional RiPP-like and lanthipeptide class I loci. *Companilactobacillus* showed a comparatively lower number of clusters, primarily represented by terpene precursors. At the species level, *L. plantarum* was the most BGC-rich species, encoding multiple RiPP-like, RaS-RiPP, terpene, and occasional NRPS clusters. Similar repertoires were observed in *L. pentosus* and *L. paraplantarum*, reflecting their expanded genomic content. *L. rhamnosus* contained the majority of lanthipeptide class IV clusters, while *F. fructivorans* and *A. kunkeei* showed narrower profiles dominated by terpene-related clusters. In contrast, several host-adapted species, such as *C. paralimentarius*, *L. mucosae*, *L. iners*, and *L. fermentum*, exhibited a near-complete absence of BGCs.

### CAZyme repertoires across former *Lactobacillus* species

CAZyme profiling of 3,813 genomes across 35 species formerly classified under the *Lactobacillus* genus revealed substantial variation in both total abundance and category composition. A total of over 530,893 CAZymes were detected, including glycoside hydrolases (GHs; 239,681) and glycosyltransferases (GTs; 209,491) as the most prevalent categories, followed by carbohydrate-binding modules (CBMs; 67,725), carbohydrate esterases (CEs; 8,243), auxiliary activities (AAs; 5,456), and polysaccharide lyases (PLs; 243). At the species level, *L. plantarum* (248,560 CAZymes), *L. rhamnosus* (47,560), and *L. reuteri* (30,073) contained the highest numbers of CAZyme-encoding genes, whereas *L. intestinalis* (884), *F. fructivorans* (894), and *L. sakei* (1,486) possessed smaller repertoires (Table S4). The average number of CAZyme genes per genome across different species within the former *Lactobacillus* and related genera is illustrated in Fig. S2. Species such as *L. plantarum*, *L. pentosus*, and *L. argentoratensis* exhibit the highest average CAZyme counts, ranging from approximately 180 to 200 genes per genome. In contrast, species such as *L. iners*, *F. sanfranciscensis*, and *F. fructivorans* display much lower CAZyme counts, often below 50 genes per genome. Across all analyzed species, GH and GT consistently dominate, forming the largest proportion of the total CAZyme content. CBM was moderately represented, while CE was variably less distributed among species. AA and PL occur at the lowest levels, found in limited species.

**Fig. 9.**
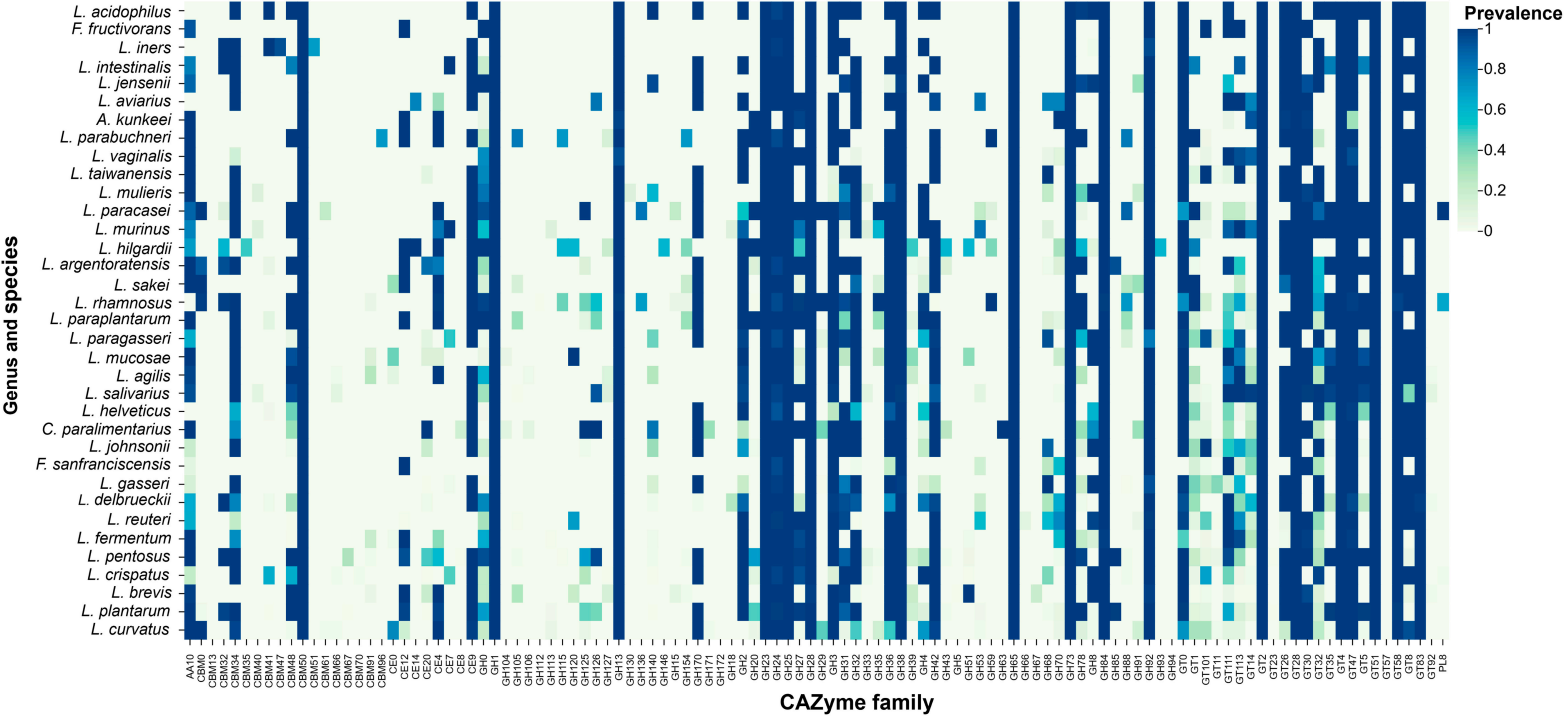
Prevalence of CAZyme families across former *Lactobacillus* species. Heatmap showing the prevalence of CAZyme families within GH, GT, CBM, PL, and AA classes annotated using dbCAN3 (DIAMOND-only mode, *E* value < 1e^−102^) against the CAZy database (last update July 2025).

Moreover, prevalence distribution across the former *Lactobacillus* species revealed substantial variability in the enzyme family (Fig. 9). A total of multiple GH, GT, and CBM families were detected, exhibiting species-specific distribution patterns. The GH families were widely represented, with several, including GH1, GH13, GH23, GH25, GH73, GH78, and GH109, present across most species. These families collectively accounted for a large proportion of the carbohydrate-degrading potential within the former *Lactobacillus* genus. GT families, particularly GT2, GT4, GT8, GT26, GT28, GT35, and GT87, were prevalent among many species, reflecting conserved carbohydrate biosynthetic capabilities. Other families, such as GT14, GT20, and GT32, occurred less frequently, displaying patchy distribution across species. Among the CBM families, CBM34, CBM50, and CBM48 were detected in several taxa, while others were sporadically distributed. Certain AA and PL families, including AA1, AA3, and PL8, were also identified, but at low prevalence. The heatmap revealed a heterogeneous distribution of CAZyme families among species, with some taxa showing broad enzyme repertoires, while others possessed a more restricted set of carbohydrate-related genes.

### Safety profiling among former *Lactobacillus* species

**Fig. 10.**
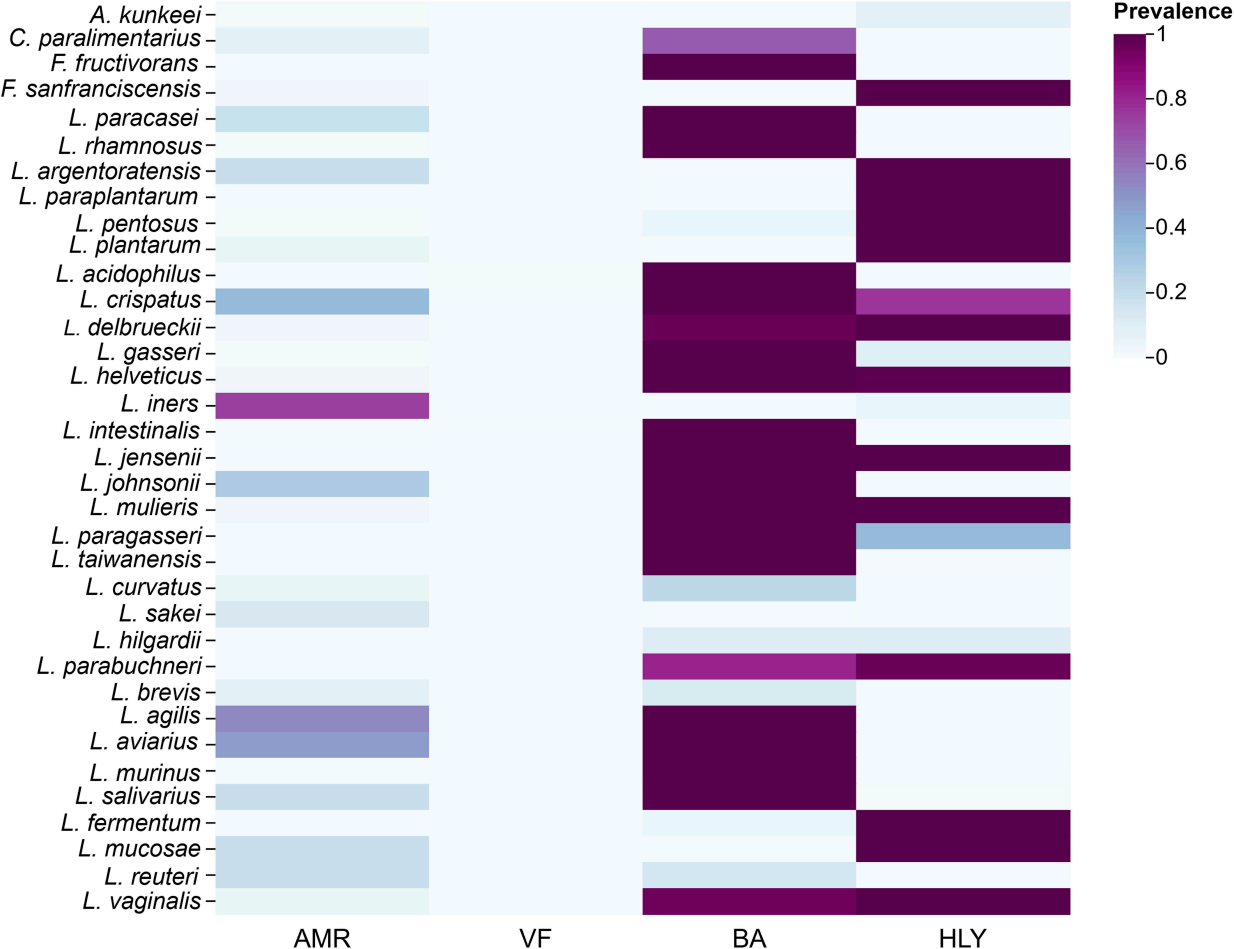
Distribution of safety-relevant genes across former *Lactobacillus* species. Heatmap illustrating the presence of ARGs, classical virulence factors, BA decarboxylases, and hemolysin or cytolysin genes across the analyzed genomes. AMR determinants were identified using ABRicate against the ResFinder database, retaining hits with ≥50% coverage and ≥90% sequence identity, while virulence factors were screened against VFDB using stricter thresholds (≥70% coverage and ≥90% identity).

Genome-wide in silico screening identified the distribution of AMR, VFs, BA, and hemolysin-associated genes among the analyzed taxa (Fig. 10). Most of the former *Lactobacillus* genomes contained a limited number of AMR genes, with *L. iners* exhibiting the highest distribution of AMR genes, followed by *L. agilis*, *L. aviaries*, and *L. crispatus*. Among the most prevalent genes were those conferring resistance to tetracyclines, such as *tet(M)*, *tet(L)*, and *tet(S)*, which were found in *L. reuteri* (14.3%) and *L. johnsonii* (21.3%). Macrolide resistance was linked to genes including *erm(B)* and *msr(D)*, with *L. iners* (21.9%) showing the highest occurrence of *msr(D)*. In addition, phenicol resistance was mediated by genes such as *cat* (0.9%) and *fexA* (1.7%), found in species *L. reuteri* (1.7%) and *L. johnsonii* (0.9%). Aminoglycoside resistance was associated with *aadD*, *str*, and *aph* genes, particularly in *L. plantarum* (2.5%) and *L. reuteri* (3.3%). β-Lactam resistance, including *blaTEM-116* (1.0%) and *blaNDM-5* (0.1%) genes, was detected in *L. plantarum* (1.2%) and *L. reuteri* (0.4%). Nitrate reductase resistance, linked to *narA* and *narB* genes, was observed in *L. curvatus* (1.5%) and *L. reuteri* (1.2%) (Table S5). Notably, classical VFs, as defined by the VFDB, were detected across the analyzed genomes; however, other safety-relevant genes, including hemolysin or cytolysin genes, and BA decarboxylases, were identified in a subset of taxa. Species, such as *F. fructivorans*, *L. paracasei*, *L. rhamnosus*, and *L. fermentum*, carried BA decarboxylase genes, which are associated with the production of BAs (e.g., histamine and tyramine), potentially posing food safety risks. In contrast, species like *F. sanfranciscensis*, *L. plantarum*, *L. iners*, and *L. sakei* exhibited a lower presence of these genes. Furthermore, some strains of *L. rhamnosus*, *L. pentosus*, and *L. plantarum* harbor hemolysin and cytolysin genes, while other species, such as *L. acidophilus* and *L. reuteri*, show fewer or no cytolysin or hemolysin genes.

### Supra-pan-genome growth and novel gene discovery

To assess genomic diversity within the former *Lactobacillus* genus, a comprehensive supra-pan-genome analysis was performed using 3,813 high-quality genomes representing 35 species of the former *Lactobacillus* (metadata in Table S6). Roary-based clustering identified an open pangenome comprising 232,087 gene families across all strains (Fig. S3A). The dataset included 2,691 shell genes (present in 15% to 95% of genomes) and 229,396 cloud genes (present in <15% of genomes). No strict core or soft-core gene families (≥95% prevalence) were detected, which is consistent with the broad taxonomic scope of the dataset and reflects extensive gene content divergence across genera. Gene frequency across these genomes illustrates a smaller number of genomes, with fewer genes being widely distributed across all strains (Fig. S3B). Moreover, the accumulation curve analysis of total gene families versus the number of genomes showed a continuously increasing trend, confirming the openness among them (Fig. S3C). Each additional genome contributed to new gene families, with diminishing yet persistent discovery rates, a pattern characteristic of metabolically versatile bacteria with wide ecological distributions. The accessory genome-based phylogenomic tree (Fig. S4) further resolved these patterns, separating former *Lactobacillus* into distinct genus- and species-level clades while integrating ecological metadata. The innermost strip represented genus-level classifications, the middle strip denoted species-level groupings, and the outer strip illustrated normalized ecological categories including human, animal, food, environment, other, and missing. This multi-layered visualization demonstrates the clear correspondence between accessory genome structure, taxonomy, and ecological origin. Strain-level analysis showed substantial variation in the number of unique genes among former *Lactobacillus* species. *L. plantarum* and *L. rhamnosus* possessed the highest numbers of unique genes per strain, whereas *F. sanfranciscensis* and *L. iners* contained comparatively fewer. The former *Lactobacillus* pangenome exhibited extensive accessory and strain-specific gene content with a minimal presence of universally conserved genes.

## Discussion

The genus *Lactobacillus* has long been recognized for its central roles in food fermentation, human health, and probiotic applications. Members of this group are essential to the fermentation of dairy products, vegetables, and other foods, where they enhance nutritional value, prevent spoilage, and inhibit the growth of pathogenic microorganisms [46,47]. In recent years, advances in genomic and phylogenetic research have led to the reclassification of many *Lactobacillus* species into distinct genera. This reorganization highlights the profound evolutionary and functional diversity within the group, calling for an in-depth exploration of the genomic characteristics and probiotic potential of these former *Lactobacillus* species [3]. This study provides a comprehensive genome-scale comparison of 3,813 genomes from 35 species formerly classified within the genus *Lactobacillus*, offering new insights into how genome architecture, ecological adaptation, functional potential, and safety-related traits vary across this reclassified group. By integrating genomic features with ecological metadata, this work advances understanding of how evolutionary pressures shape probiotic-associated traits and functional diversity at a family-wide scale. A key finding of this study is the pronounced genome size and GC content variation observed across the 3,813 genomes representing 35 former *Lactobacillus* species. Genome sizes ranged from 1.21 to 4.05 Mb, with GC content spanning 32.5% to 53.0%, consistent with earlier reports of extensive genomic heterogeneity within this group [2,48]. Species such as *L. plantarum* and *L. pentosus* exhibited large genomes, reflecting their capacity to metabolize a wide array of substrates and thrive in diverse environments. In contrast, *L. crispatus* and *L. iners* possessed markedly reduced genomes, a characteristic associated with niche specialization and genome streamlining in host-adapted environments, particularly the vaginal microbiota [49,50]. These patterns support the concept that genome architecture in former *Lactobacillus* species is strongly shaped by ecological niche and lifestyle [51–53]. Integration of ecological metadata further revealed clear relationships between genome architecture, ecology, and isolation source. Fermentation-associated species, including *F. sanfranciscensis* and *C. paralimentarius*, were almost exclusively isolated from cereal-based fermentations, consistent with their specialization as sourdough-associated taxa [54,55]. By contrast, generalists such as *L. plantarum* showed remarkable ecological versatility, spanning food, plant, and human niches, supported by large genomes and extensive metabolic repertoires [56,57]. Human-associated species showed a bimodal pattern, with reduced genomes in *L. iners* reflecting specialization, and expanded genomes in *L. plantarum* reflecting a generalist lifestyle [58,59], whereas *A. kunkeei* exemplified ecological specialization through insect symbiosis. Statistical analyses confirmed significant genome size differences across ecological categories (Kruskal–Wallis *P* < 10^−229^). Fermentation-associated isolates generally exhibited smaller genomes, consistent with adaptation to nutrient-rich and relatively stable environments. Environmental isolates harbored larger genomes, reflecting the metabolic flexibility required to cope with fluctuating conditions. Human-associated taxa displayed a bimodal pattern: Genome reduction in *L. iners* likely reflects reductive evolution driven by specialization, while genome expansion in *L. plantarum* supports a generalist strategy. Animal-associated species occupied an intermediate position, balancing host adaptation with metabolic versatility [60]. These findings demonstrate that ecological pressures are a primary driver of genome evolution in former *Lactobacillus* species and provide an essential framework for interpreting their functional capacity and probiotic relevance.

The study examined the distribution of PMGs across species and identified conserved functional categories that are essential for probiotic efficacy. These include stress tolerance (acid, heat, cold), amino acid and energy metabolism, and vitamin and cofactor biosynthesis. The consistent presence of these functions across species aligns with earlier studies showing that probiotic LAB strains must withstand the harsh conditions of the gastrointestinal tract (GIT) [61,62]. Genes associated with carbohydrate utilization and transport were broadly distributed, particularly in species such as *L. crispatus*, *L. pentosus*, and *L. rhamnosus*. This observation aligns with prior reports showing that LABs possess diverse CAZyme repertoires enabling the metabolism of a wide range of dietary carbohydrates. Through the fermentation of nondigestible fibers and complex polysaccharides, these species may contribute to host health by producing short-chain fatty acids (SCFAs), which play key roles in gut barrier integrity and immune modulation [63,64]. In addition, genes involved in EPS biosynthesis and surface adhesion, notably enriched in *L. crispatus* and *L. pentosus*, are critical for epithelial adherence, biofilm formation, and long-term persistence within the gut microbiota [65,66]. The presence of GABA production genes in *L. plantarum* is a notable finding, supporting earlier studies that have linked LAB with the production of neurotransmitters, which could contribute to gut–brain communication [67,68]. Moreover, vitamin and cofactor biosynthesis was found to be enriched in species such as *L. hilgardii* and *L. parabuchneri*, aligning with the findings of other studies that demonstrate the ability of LABs to synthesize B vitamins, which play crucial roles in human health [69]. These findings highlight that former *Lactobacillus* species contribute to host well-being through multiple complementary mechanisms, including metabolic support, stress resilience, and immune modulation. Our initial PMG identification was based on uniform Prokka re-annotation combined with curated regex-based matching, allowing scalable screening across a large genome dataset. Nevertheless, annotation-driven approaches may produce false positives when functional labels are broad or shared among paralogous proteins, and false negatives when gene nomenclature varies across annotations. To address these limitations, we performed BLAST-based sequence validation for selected representative single-locus markers and report both the regex patterns and validation results. PMGs that were not subjected to sequence-level validation should therefore be interpreted as putative indicators of functional capacity rather than conclusive evidence of biological activity. Analysis of bacteriocin-encoding genes revealed substantial species-specific variability, consistent with previous reports [70]. Species such as *L. plantarum*, *L. rhamnosus*, and *L. gasseri* exhibited the greatest diversity of bacteriocins, particularly class IIa and IIb bacteriocins, known for their ability to inhibit pathogenic microorganisms [70–72]. Notably, *L. plantarum* harbored multiple distinct bacteriocin loci, including plantaricins, which are widely exploited in fermented food systems to control microbial growth [73,74]. In contrast, *A. kunkeei* and *F. fructivorans* displayed narrower bacteriocin profiles, suggesting reduced reliance on bacteriocin-mediated competition and possible dependence on alternative ecological strategies such as metabolic specialization or biofilm formation [75]. Subclass-level analysis of bacteriocins across species demonstrated clear patterns, with *L. plantarum* and *L. paraplantarum* exhibiting the broadest diversity of bacteriocin subclasses, predominantly class IIb peptides, consistent with their robustness in fermentation environments where competitive exclusion of spoilage organisms is critical [76,77]. Conversely, species *L. helveticus* and *L. jensenii*, which predominantly harbor class III bacteriocins, are more likely to produce large, heat-labile antimicrobial proteins, often associated with dairy fermentations [78,79]. Genome mining of BGCs revealed the presence of several cluster types, including terpenes, RiPPs, and lanthipeptides, with *L. plantarum* showing the greatest diversity. This finding is consistent with previous studies identifying *L. plantarum* as a prolific producer of bioactive compounds with potential industrial and therapeutic applications [80]. The presence of terpene-related clusters in species *L. plantarum* and *L. rhamnosus* is particularly interesting, as terpenes have applications in pharmaceuticals and food preservation [81,82]. The species-specific profiles of BGCs, such as the dominance of terpene-precursor clusters in *A. kunkeei* and *F. fructivorans*, further reflect the ecological specialization of these species [83]. Therefore, while the genomic data offer valuable insights into the genetic potential of these strains, experimental validation of their antimicrobial activity and bioactive compound production through phenotypic assays is necessary to confirm their antimicrobial properties and evaluate their antagonistic effects against common pathogens in laboratory settings. The profiling of CAZymes across the 35 species revealed considerable variation in enzyme content, with *L. plantarum* possessing the highest number of CAZyme genes. This observation is consistent with its well-documented metabolic versatility, which enables utilization of a wide range of carbohydrates derived from both plant- and host-associated sources [84]. The dominance of GHs and GTs in *L. plantarum* and *L. rhamnosus* suggests that these species play crucial roles in carbohydrate degradation and biosynthesis within the human gut microbiome. These enzymes facilitate the breakdown of complex polysaccharides, dietary fibers, and prebiotic substrates, such as inulin, fructooligosaccharides (FOS), and galactooligosaccharides (GOS), into simpler sugars that can be fermented into SCFAs, essential for intestinal health [60,85]. In contrast, species such as *L. iners* and *F. fructivorans*, which exhibited lower CAZyme counts, likely possess more specialized carbohydrate metabolisms tailored to their ecological niches, such as the vaginal microbiota or marine habitats [86]. These streamlined CAZyme profiles likely reduce metabolic costs in habitats where complex carbohydrate degradation is less critical. Overall, the broad distribution of CAZymes among former *Lactobacillus* species underscores their ecological flexibility and their key roles in prebiotic utilization. Several taxa, particularly *L. plantarum*, *L. pentosus*, and *L. rhamnosus*, are well known for their capacity to metabolize prebiotics, nondigestible carbohydrates that selectively stimulate beneficial gut microbes [87]. By expressing diverse GH and GT families, these species can utilize prebiotics, such as inulin, arabinoxylan, and resistant starches, generating SCFAs (acetate, propionate, and butyrate) that contribute to gut barrier integrity, pH regulation, and immune modulation [88]. This capacity not only enhances host nutrition but also promotes other beneficial microbial cross-feeding interactions within the gut ecosystem. For example, the coculture of the *Lactobacillus* group and the genus *Bifidobacterium* can utilize oligosaccharides and promote energy and growth of other strains, reinforcing the symbiotic relationship between these probiotic taxa [89]. Therefore, the CAZyme diversity observed in this study underscores the potential of these species as effective synbiotic partners, probiotics capable of thriving on and enhancing the benefits of prebiotics. These findings emphasize their functional relevance in gut health maintenance and support their continued development in functional foods and precision probiotic applications. However, while the presence of these CAZyme genes suggests metabolic capability, phenotypic characterization through fermentation assays and growth tests would be necessary to confirm functionality. Moreover, the genome-wide in silico screening revealed key findings regarding AMR, VFs, BA production, and hemolysin-associated genes in the former *Lactobacillus* genomes. The analysis shows that most of the species contained a relatively small number of AMR genes, with *L. iners* demonstrating the highest distribution, followed by *L. agilis*, *L. aviaries*, and *L. crispatus*. Among these, tetracycline resistance, found in *L. johnsonii* and *L. reuteri*, and macrolide resistance in *L. iners* suggest potential concerns in therapeutic applications. Phenicol and aminoglycoside resistance was observed in *L. reuteri* and *L. johnsonii*, indicating the need for careful strain selection. Moreover, β-lactam resistance in *L. plantarum* and *L. reuteri* raises concerns about resistance in clinical settings. Nitrate reductase resistance in *L. curvatus* and *L. reuteri* suggests broader implications for their use in food products. These findings emphasize the importance of monitoring AMR in probiotic strains. The most commonly found antibiotic-resistance genes (ARGs) in former *Lactobacillus* genomes include tetracycline resistance genes [*tet(M)*, *tet(L)*], macrolide resistance genes [*erm(B*), msr(D)], phenicol resistance genes (*cat*, *fexA*), aminoglycoside resistance genes (*aadD*, *str*), and β-lactam resistance genes (*blaTEM-116*). These genes are primarily detected in *L. reuteri*, *L. plantarum*, *L. johnsonii*, and *L. iners*, with common isolation sources including animal gut, human gut, animal feces, and some fermented foods. These findings highlight the diverse resistance profiles of Lactobacillaceae, which are critical to consider in probiotic applications and fermented food production. Interestingly, no classical VFs were identified, suggesting that these species lack major virulence determinants despite the presence of certain resistance-associated genes. However, the detection of safety-relevant genes, including hemolysin or cytolysin genes and BA decarboxylases, highlights that genome-based safety assessment alone is insufficient and that strain-level phenotypic evaluation is necessary prior to probiotic application. In terms of BA production, several species, including *F. fructivorans*, *L. paracasei*, *L. rhamnosus*, and *L. fermentum*, contained BA decarboxylase genes, which are involved in the production of BAs like histamine and tyramine. The presence of these genes raises potential food safety concerns, as high levels of BAs can lead to food poisoning or other health issues [90]. On the other hand, species such as *F. sanfranciscensis*, *L. plantarum*, *L. iners*, and *L. sakei* exhibited a lower presence of these genes, suggesting a reduced potential for BA production and a lower associated food safety risk. The hemolysin and cytolysin genes, which are associated with cell membrane disruption and may contribute to pathogenicity [91], were found in some strains of *L. rhamnosus*, *L. pentosus*, and *L. plantarum*. However, other species like *L. acidophilus* and *L. reuteri* showed fewer or no cytolysin or hemolysin genes, indicating that these species might be less prone to causing cell damage or contributing to pathogenic behaviors. The supra-pan-genome analysis revealed an open pangenome with a vast array of accessory genes, reflecting the genetic diversity of *Lactobacillus* species. This finding is consistent with previous studies that have shown that *Lactobacillus* species exhibit extensive strain-level variability, which allows them to adapt to diverse environmental conditions [92]. The open structure, characterized by a large number of accessory genes, supports findings from previous studies, showing that *Lactobacillus* species evolve rapidly through horizontal gene transfer, gene gain, and loss. The absence of a strict core genome highlights the functional flexibility, enabling adaptation of the species to various environments, including the human gut, fermented foods, and environmental niches [93]. Nevertheless, species-level pan-genome analyses are more appropriate for identifying conserved core genes. The present study instead focuses on higher-level gene repertoire diversity across the reclassified *Lactobacillus* group. The presence of genes for carbohydrate utilization, stress tolerance, and defense mechanisms in the accessory genome emphasizes the importance of these traits for survival in diverse ecological conditions. Species *L. plantarum* and *L. rhamnosus* exhibit high genetic diversity, enabling them to thrive in both food-based and host-associated environments, while species like *L. iners* have more specialized genetic profiles suited for specific niches. The accessory genome plays a critical role in niche adaptation by encoding genes related to carbohydrate metabolism, biofilm formation, and AMR. This supports the idea that *Lactobacillus* species are highly adaptable, allowing them to occupy a wide range of ecological niches. The diversity in gene content further enhances their ability to interact with the microbiome, metabolize prebiotics, and contribute to gut health [48]. Moreover, this variability in genetic content is consistent with findings from studies on other probiotic genera, where gene exchange and adaptation to host environments are fundamental to their survival and functional roles. The current genomic functional analysis provides valuable insights. We acknowledge that further in-depth exploration of species-specific functional variations, biochemical pathways, and strain-level differences is necessary to fully understand the genetic diversity, probiotic potential, and ecological adaptations of these species. However, it is important to note that this study is primarily based on genomic comparative analysis, and experimental confirmatory research is lacking. While genomic data provide valuable insights into the genetic potential of these strains, phenotypic validation through laboratory-based assays is necessary to confirm the actual functionality of the predicted traits, such as antimicrobial activity and probiotic potential. Future studies should focus on experimental validation to support these findings and further investigate the functional roles of these strains in real-world applications.

## Data Availability

Data will be made available on request.
