# Supplementary material for: From Fermentation to Function: Genomic Diversity and Probiotic Potential in the Reclassified Lactobacillus Lineage
Source: Comput Struct Biotechnol J. 2026 Mar 16;35(1):0004. doi: 10.34133/csbj.0004 (PMC13067958; doi:10.34133/csbj.0004)
Supplement: Supplementary 1 — Figs. S1 to S4 Tables S1 to S6 [file csbj.0004.f0004.zip › Supplementary_Legend_RV1.docx]

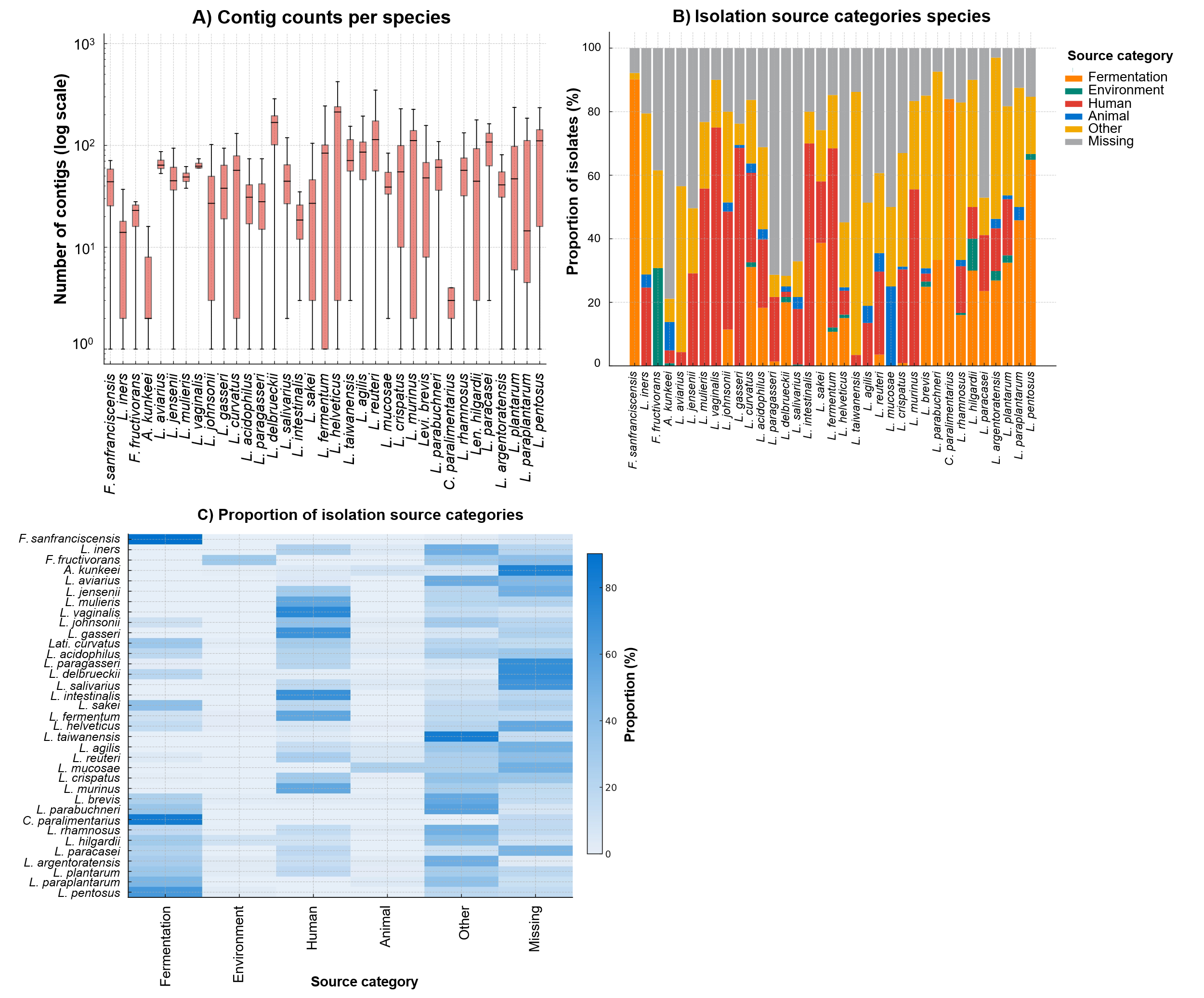


**Figure S1.** **Genome characteristics and isolation source categories of the former *Lactobacillus* genus.** (A) Assembly contiguity per species, measured by the number of contigs on a log scale. (B) Distribution of isolation source categories presented as stacked bar plots by species. (C) Heatmap of species and isolation source categories showing the proportional distribution of genomes in each ecological group.


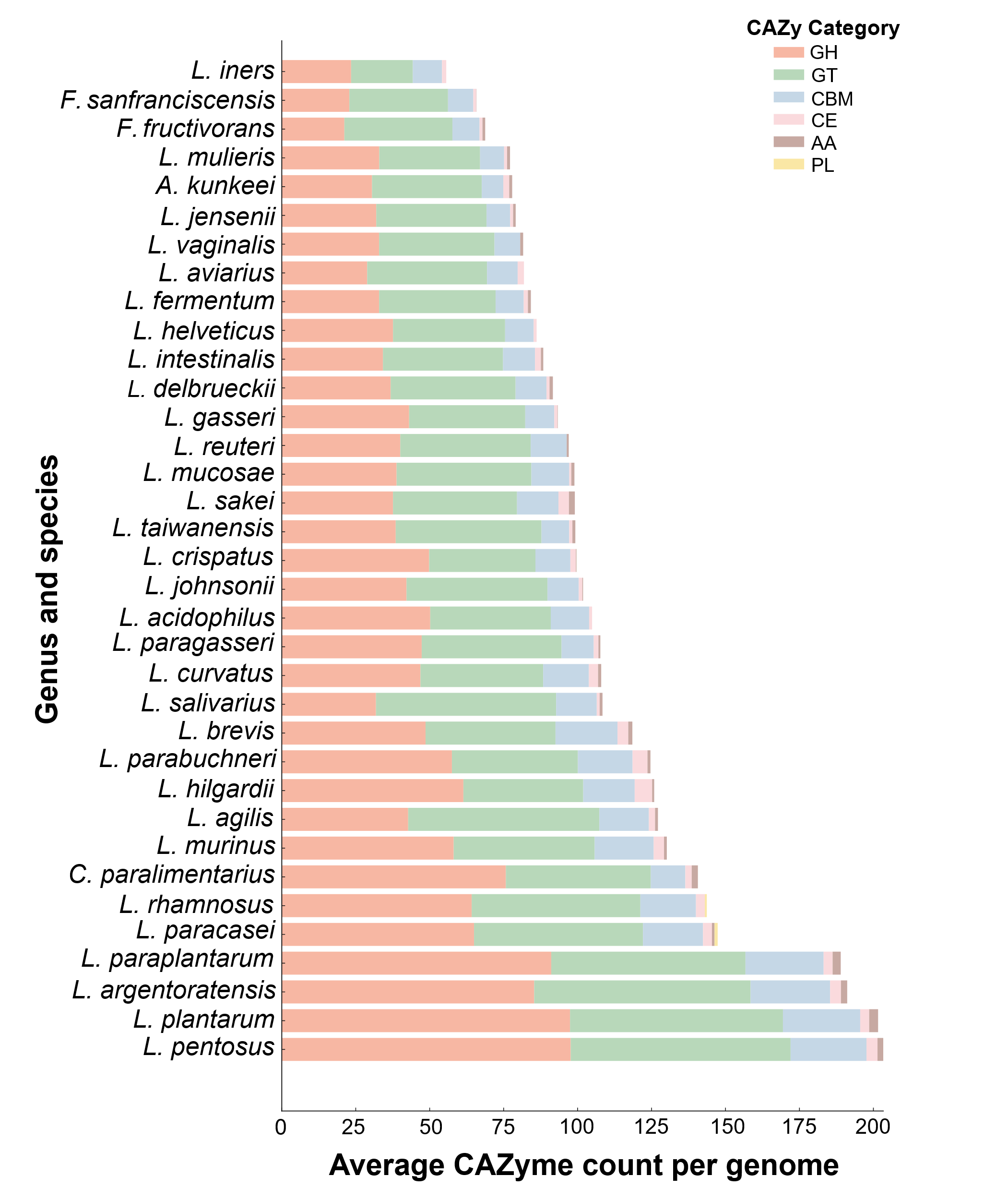


**Figure S2**. **The average CAZyme counts per genome for each species, grouped by CAZy category (GH, GT, CBM, CE, AA, PL).**


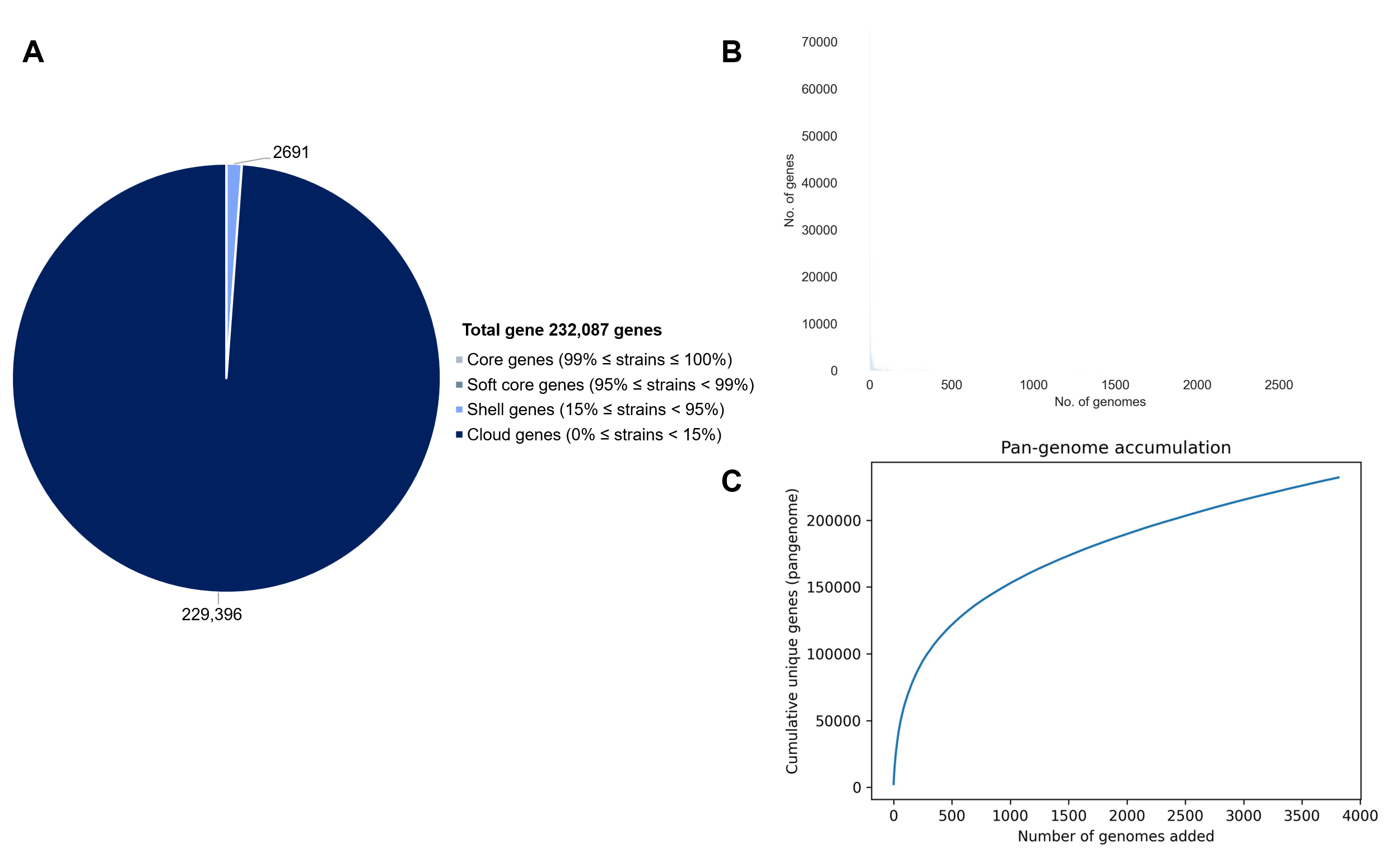


**Figure S3.** **Pan-genome analysis of 3,813 former *Lactobacillus* genomes.** (A) Distribution of core, soft core, shell, and cloud genes, with 232,087 total genes identified. (B) Gene frequency across genomes, showing the concentration of genes in fewer genomes. (C) Pangenome accumulation curve, illustrating the open pangenome structure with continuous gene addition as more genomes are included.


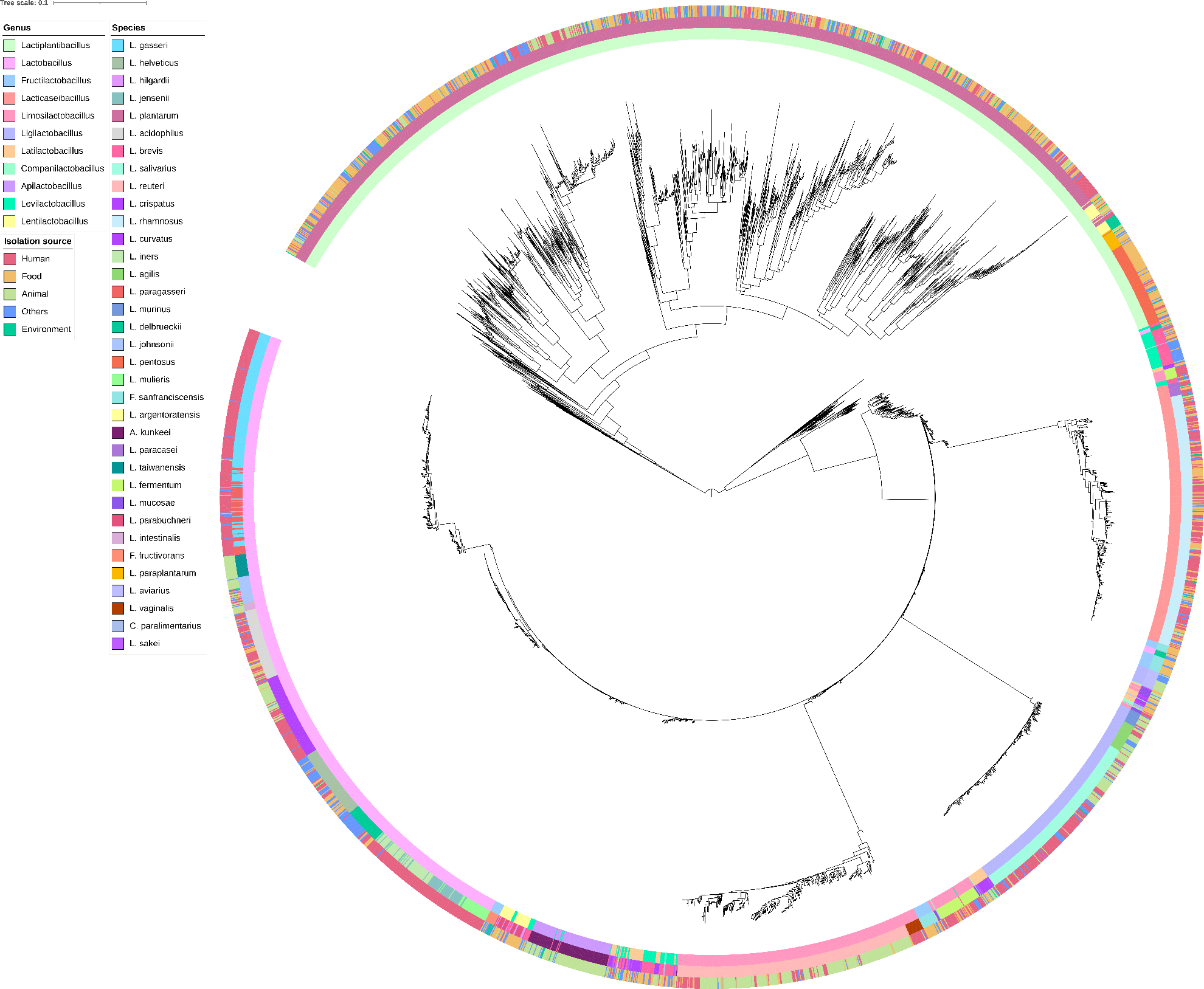


**Figure S4. Phylogenomic tree of former *Lactobacillus* with genus, species, and isolation source annotations.** The maximum-likelihood accessory genome tree was constructed from the binary gene presence or absence matrix and visualized in iTOL with three metadata layers. The innermost strip denotes genus assignment, highlighting major taxonomic clades within former *Lactobacillus*. The second strip represents species-level classification. The outer strip indicates the normalized isolation source, which was categorized by the sample type of isolation or host of each strain.

**Table S1** Genome characteristics summary for 35 species previously classified under the *Lactobacillus* species.

| Species | Genome number | Mean of genome size (bp) | Maximum genome size (bp) | Minimum genome size (bp) | Mean of GC content (%) | Mean of contig number | Mean of completeness (%) | Mean of contamination (%) |
| --- | --- | --- | --- | --- | --- | --- | --- | --- |
| *A. kunkeei* | 246.00 | 1576120.52 | 1665937 | 1406916 | 36.58 | 10.83 | 97.56 | 1.36 |
| *C. paralimentarius* | 25.00 | 2731815.56 | 2870988 | 2649162 | 35.42 | 12.64 | 98.90 | 2.28 |
| *F. fructivorans* | 26.00 | 1365753.85 | 1416533 | 1332231 | 39.00 | 22.62 | 97.01 | 0.40 |
| *F. sanfranciscensis* | 102.00 | 1303700.82 | 1373332 | 1242512 | 34.65 | 39.27 | 98.73 | 0.60 |
| *L. paracasei* | 34.00 | 3095351.71 | 3293134 | 2902983 | 46.21 | 117.24 | 95.92 | 2.86 |
| *L. rhamnosus* | 900.00 | 2959500.98 | 3436367 | 2815512 | 46.56 | 57.62 | 98.52 | 0.66 |
| *L. argentoratensis* | 67.00 | 3175916.52 | 3350338 | 3016664 | 45.04 | 49.64 | 99.21 | 1.39 |
| *L. paraplantarum* | 48.00 | 3298551.29 | 3521459 | 3007437 | 43.75 | 55.13 | 99.25 | 1.76 |
| *L. pentosus* | 222.00 | 3767678.40 | 4050922 | 3512307 | 46.03 | 95.94 | 99.29 | 2.08 |
| *L. plantarum* | 2473.00 | 3286541.72 | 3874264 | 2611772 | 44.46 | 68.95 | 99.10 | 1.49 |
| *L. acidophilus* | 186.00 | 1973714.65 | 2046837 | 1947187 | 34.53 | 33.14 | 96.37 | 0.32 |
| *L. crispatus* | 224.00 | 2230695.02 | 2912805 | 1919726 | 36.95 | 95.67 | 96.47 | 2.15 |
| *L. delbrueckii* | 120.00 | 1979107.02 | 2167229 | 1775601 | 49.67 | 171.18 | 97.76 | 0.79 |
| *L. gasseri* | 446.00 | 1928773.93 | 2251884 | 1786325 | 34.95 | 41.29 | 98.01 | 0.96 |
| *L. helveticus* | 186.00 | 2023990.20 | 2270176 | 1749160 | 36.67 | 175.26 | 97.33 | 0.87 |
| *L. iners* | 146.00 | 1318987.89 | 1507452 | 1211893 | 32.81 | 16.78 | 98.54 | 0.82 |
| *L. intestinalis* | 20.00 | 2011478.00 | 2129238 | 1979369 | 35.55 | 18.00 | 98.69 | 0.16 |
| *L. jensenii* | 127.00 | 1688995.83 | 1975951 | 1471255 | 34.35 | 65.47 | 96.52 | 1.37 |
| *L. johnsonii* | 70.00 | 1925674.54 | 2153934 | 1725518 | 34.59 | 32.06 | 96.17 | 1.42 |
| *L. mulieris* | 86.00 | 1747231.47 | 1847214 | 1565593 | 34.09 | 52.26 | 98.31 | 0.21 |
| *L. paragasseri* | 143.00 | 1977767.76 | 2133921 | 1879678 | 34.81 | 36.63 | 98.84 | 0.23 |
| *L. taiwanensis* | 58.00 | 2048845.59 | 2248719 | 1842579 | 34.02 | 80.83 | 99.02 | 0.18 |
| *L. curvatus* | 135.00 | 1944432.88 | 2175649 | 1761713 | 41.88 | 48.52 | 98.37 | 0.22 |
| *L. sakei* | 31.00 | 2014934.77 | 2176523 | 1824746 | 41.00 | 31.00 | 98.21 | 0.56 |
| *L. hilgardii* | 20.00 | 3036417.10 | 3204128 | 2654177 | 39.90 | 61.50 | 99.25 | 0.48 |
| *L. parabuchneri* | 54.00 | 2654004.11 | 2800230 | 2530379 | 43.44 | 62.00 | 98.67 | 0.75 |
| *L. brevis* | 241.00 | 2486694.10 | 2884677 | 2242019 | 45.86 | 51.73 | 98.52 | 2.59 |
| *L. agilis* | 74.00 | 2140464.86 | 2342156 | 1949635 | 41.55 | 76.19 | 98.17 | 0.92 |
| *L. aviarius* | 46.00 | 1607608.96 | 1688696 | 1506933 | 40.15 | 64.61 | 99.06 | 0.71 |
| *L. murinus* | 36.00 | 2272367.50 | 2722946 | 2042132 | 40.00 | 83.28 | 98.63 | 0.56 |
| *L. salivarius* | 480.00 | 1982950.93 | 2338231 | 1799085 | 32.80 | 50.88 | 96.61 | 1.81 |
| *L. fermentum* | 149.00 | 2021143.15 | 2230631 | 1867005 | 51.90 | 72.72 | 96.16 | 3.25 |
| *L. mucosae* | 32.00 | 2184442.75 | 2402030 | 1975815 | 46.47 | 48.00 | 98.92 | 0.44 |
| *L. reuteri* | 620.00 | 2141398.84 | 2695904 | 1821307 | 38.63 | 126.69 | 97.53 | 1.64 |
| *L. vaginalis* | 40.00 | 1821239.20 | 1936702 | 1657171 | 40.45 | 63.75 | 99.01 | 0.67 |

**Table S2** Probiotic Marker Gene (PMG) identification using regex-based keyword matching Prokka annotations. The table lists marker genes, their functions, and the corresponding regex patterns used to identify PMGs in 35 species.

| Category | Marker | Gene function | Regex pattern |
| --- | --- | --- | --- |
| Gut persistence | *mub* | Mucus-binding protein (adhesion/colonization) | \bmub\b |
|  | *mapA* | Mucus adhesion-promoting protein | \bmapA\b |
|  | *srtA* | Sortase A (surface protein anchoring) | \bsrtA\b |
|  | *srtA* | Sortase A (surface protein anchoring) | \bsortase\s*A\b |
|  | LPXTG_protein | LPXTG/LPQTG motif (anchored surface proteins) | \bLPXTG\b |
|  | LPXTG_protein | LPXTG/LPQTG motif (anchored surface proteins) | \bLPQTG\b |
|  | *spa* | Sortase-dependent pili/pilin proteins | \bspaA\b |
|  | *spa* | Sortase-dependent pili/pilin proteins | \bspaB\b |
|  | *spa* | Sortase-dependent pili/pilin proteins | \bspaC\b |
|  | *spa* | Sortase-dependent pili/pilin proteins | \bspaD\b |
|  | *spa* | Sortase-dependent pili/pilin proteins | \bpilin\b |
|  | *spa* | Sortase-dependent pili/pilin proteins | \bpilus\b |
|  | fibronectin_binding | Fibronectin-binding adhesins | \bfnbA\b |
|  | fibronectin_binding | Fibronectin-binding adhesins | \bfnbB\b |
|  | fibronectin_binding | Fibronectin-binding adhesins | \bfbpA\b |
|  | collagen_binding | Collagen-binding adhesin | \bcna\b |
|  | *slpA* | S-layer protein (surface interaction) | \bslpA\b |
|  | *wxl* | WxL-domain cell wall-binding proteins | \bWxL\b |
|  | *lysM* | LysM peptidoglycan-binding proteins | \bLysM\b |
|  | *lysM* | LysM peptidoglycan-binding proteins | \blysin\s+motif\b |
|  | *wzb* | Tyrosine phosphatase (EPS/capsule regulation) | \bwzb\b |
|  | *wzc* | Tyrosine kinase (EPS/capsule regulation) | \bwzc\b |
|  | *wzx* | Polysaccharide flippase (EPS/capsule export) | \bwzx\b |
|  | *wzy* | Polysaccharide polymerase (EPS/capsule assembly) | \bwzy\b |
|  | *gtf* | Glucosyltransferase (EPS synthesis) | \bgtf\b |
|  | *gtf* | Glucosyltransferase (EPS synthesis) | \bglucosyltransferase\b |
|  | *ugd* | UDP-glucose dehydrogenase (EPS precursors) | \bugd\b |
|  | *galU* | UTP-glucose-1-phosphate uridylyltransferase (EPS precursors) | \bgalU\b |
|  | *dltA* | Teichoic acid D-alanylation (surface charge; persistence) | \bdltA\b |
|  | *dltB* | Teichoic acid D-alanylation | \bdltB\b |
|  | *dltC* | Teichoic acid D-alanylation | \bdltC\b |
|  | *dltD* | Teichoic acid D-alanylation | \bdltD\b |
|  | adhesin_generic | Adhesin annotation keyword (generic) | \badhesin\b |
| Bile salt resistance | *bsh* | Bile salt hydrolase (deconjugation of bile salts) | \bbsh\b |
|  | *bsh* | Bile salt hydrolase (deconjugation of bile salts) | \bbile\s+salt\s+hydrolase\b |
|  | *bsh* | Bile salt hydrolase (deconjugation of bile salts) | \bbile\s+acid\s+hydrolase\b |
|  | *bsh* | Bile salt hydrolase (deconjugation of bile salts) | \bcholoylglycine\s+hydrolase\b |
|  | *bsh* | Bile salt hydrolase (deconjugation of bile salts) | \bcholate?\s+hydrolase\b |
|  | *bmrR* | MerR-family regulator (bmrRAB bile/multidrug module) | \bbmrR\b |
|  | *bmrA* | ABC transporter component (bile/multidrug efflux) | \bbmrA\b |
|  | *bmrB* | ABC transporter component (bile/multidrug efflux) | \bbmrB\b |
|  | *bilE* | Bile exclusion system (BilE; bile tolerance; Listeria-like) | \bbilE(?:A\|B)?\b |
|  | *mdrT* | Multidrug/bile efflux transporter (MdrT; Listeria-like) | \bmdrT\b |
|  | *mdrM* | Multidrug/bile efflux transporter (MdrM; Listeria-like) | \bmdrM\b |
|  | *lmrA* | Multidrug ABC transporter (bile tolerance-associated in LAB) | \blmrA\b |
|  | *lmrP* | Multidrug MFS transporter (bile tolerance-associated in LAB) | \blmrP\b |
|  | *lmrC* | Multidrug transporter component (LmrCD system) | \blmrC\b |
|  | *lmrD* | Multidrug transporter component (LmrCD system) | \blmrD\b |
|  | *lmrCD* | Multidrug transporter system label (LmrCD) | \blmrCD\b |
|  | *cfa* | Cyclopropane fatty acid synthase (membrane adaptation; bile tolerance) | \bcfa\b |
|  | *cfa* | Cyclopropane fatty acid synthase (membrane adaptation; bile tolerance) | \bcyclopropane\s+fatty\s+acid\s+synthase\b |
|  | *cfa* | Cyclopropane fatty acid synthase (membrane adaptation; bile tolerance) | \bcyclopropane\s+fatty\s+acyl\s+synthase\b |
|  | bile_MFS_efflux | Bile-associated MFS transporter (keyword-based) | \bbile\b.*\b(MFS\|major\s+facilitator)\b.*\b  (transporter\|efflux\|permease)\b |
| Adhesion and biofilm formation | *mub* | Mucus-binding protein (MucBP/MUB; adhesion) | \bmub\b |
|  | *mapA* | Mucus adhesion-promoting protein | \bmapA\b |
|  | *srtA* | Sortase A (LPXTG anchoring) | \bsrtA\b |
|  | *srtA* | Sortase A (LPXTG anchoring) | \bsortase\s*A\b |
|  | *srtB* | Sortase B (surface protein anchoring) | \bsrtB\b |
|  | *srtB* | Sortase B (surface protein anchoring) | \bsortase\s*B\b |
|  | *srtC* | Sortase C (pili assembly) | \bsrtC\b |
|  | *srtC* | Sortase C (pili assembly) | \bsortase\s*C\b |
|  | *srtF* | Sortase F (pili/surface anchoring) | \bsrtF\b |
|  | *srtF* | Sortase F (pili/surface anchoring) | \bsortase\s*F\b |
|  | LPXTG_protein | LPXTG/LPQTG motif (sortase-anchored surface proteins) | \bLPXTG\b |
|  | LPXTG_protein | LPXTG/LPQTG motif (sortase-anchored surface proteins) | \bLPQTG\b |
|  | *spa* | Sortase-dependent pili/pilin proteins (adhesion) | \bspaA\b |
|  | *spa* | Sortase-dependent pili/pilin proteins (adhesion) | \bspaB\b |
|  | *spa* | Sortase-dependent pili/pilin proteins (adhesion) | \bspaC\b |
|  | *spa* | Sortase-dependent pili/pilin proteins (adhesion) | \bspaD\b |
|  | *spa* | Sortase-dependent pili/pilin proteins (adhesion) | \bpilin\b |
|  | *spa* | Sortase-dependent pili/pilin proteins (adhesion) | \bpilus\b |
|  | fimbriae | Fimbrial proteins (adhesion) | \bfimA\b |
|  | fimbriae | Fimbrial proteins (adhesion) | \bfimB\b |
|  | fimbriae | Fimbrial proteins (adhesion) | \bfimH\b |
|  | fimbriae | Fimbrial proteins (adhesion) | \bfimbr(?:ia\|iae\|ial)\b |
|  | fibronectin_binding | Fibronectin-binding adhesins (host ECM binding) | \bfnbA\b |
|  | fibronectin_binding | Fibronectin-binding adhesins (host ECM binding) | \bfnbB\b |
|  | fibronectin_binding | Fibronectin-binding adhesins (host ECM binding) | \bfbpA\b |
|  | collagen_binding | Collagen-binding adhesin (Cna/ECM binding) | \bcna\b |
|  | *slpA* | S-layer protein (surface layer; adhesion) | \bslpA\b |
|  | *wxl* | WxL-domain cell wall-binding proteins (Gram+ surface proteins) | \bWxL\b |
|  | *lysM* | LysM peptidoglycan-binding domain proteins | \bLysM\b |
|  | *lysM* | LysM peptidoglycan-binding domain proteins | \blysin\s+motif\b |
|  | *agg* | Aggregation substance (cell-cell adhesion; biofilm) | \bagg\b |
|  | *agg* | Aggregation substance (cell-cell adhesion; biofilm) | \baggregation\s+substance\b |
|  | *icaA* | PNAG/PIA biofilm polysaccharide biosynthesis (IcaA) | \bicaA\b |
|  | *icaB* | PNAG/PIA biofilm polysaccharide modification (IcaB) | \bicaB\b |
|  | *icaC* | PNAG/PIA biofilm polysaccharide export (IcaC) | \bicaC\b |
|  | *icaD* | PNAG/PIA biofilm polysaccharide biosynthesis (IcaD) | \bicaD\b |
|  | *pgaA* | PNAG polysaccharide export (PgaA) | \bpgaA\b |
|  | *pgaB* | PNAG polysaccharide deacetylase (PgaB) | \bpgaB\b |
|  | *pgaC* | PNAG polysaccharide synthase (PgaC) | \bpgaC\b |
|  | *pgaD* | PNAG polysaccharide synthesis accessory (PgaD) | \bpgaD\b |
|  | *tasA* | Biofilm matrix protein (TasA; Bacillus-like) | \btasA\b |
|  | *tapA* | Biofilm matrix assembly protein (TapA; Bacillus-like) | \btapA\b |
|  | *bslA* | Biofilm hydrophobin-like protein (BslA; Bacillus-like) | \bbslA\b |
|  | *wzb* | Tyrosine phosphatase (EPS/capsule regulation) | \bwzb\b |
|  | *wzc* | Tyrosine kinase (EPS/capsule regulation) | \bwzc\b |
|  | *wzx* | Polysaccharide flippase (EPS/capsule export) | \bwzx\b |
|  | *wzy* | Polysaccharide polymerase (EPS/capsule assembly) | \bwzy\b |
|  | *ugd* | UDP-glucose dehydrogenase (EPS precursors) | \bugd\b |
|  | *galU* | UTP-glucose-1-phosphate uridylyltransferase (EPS precursors) | \bgalU\b |
|  | *gtf* | Glucosyltransferase (exopolysaccharide synthesis) | \bgtf\b |
|  | *gtf* | Glucosyltransferase (exopolysaccharide synthesis) | \bglucosyltransferase\b |
|  | *ftf* | Fructosyltransferase (exopolysaccharide synthesis) | \bftf\b |
|  | *ftf* | Fructosyltransferase (exopolysaccharide synthesis) | \bfructosyltransferase\b |
|  | dextransucrase | Dextransucrase (dextran biofilm polymer synthesis) | \bdextran\s*sucrose\s*glucosyltransferase\b |
|  | dextransucrase | Dextransucrase (dextran biofilm polymer synthesis) | \bdextransucrase\b |
|  | levansucrase | Levansucrase/SacB (levan synthesis) | \blevansucrase\b |
|  | levansucrase | Levansucrase/SacB (levan synthesis) | \bsacB\b |
|  | adhesin_generic | Adhesin annotation keyword (generic) | \badhesin\b |
|  | biofilm_generic | Biofilm-related annotation keyword (generic) | \bbiofilm\b.*\b(protein\|matrix\|associated\|formation\|  regulator\|exopolysaccharide)\b |
| Osmotic stress | *proV* | ProU ABC transporter ATP-binding/permease component (osmoprotectant uptake) | \bproV\b |
|  | *proW* | ProU ABC transporter permease component (osmoprotectant uptake) | \bproW\b |
|  | *proX* | ProU ABC transporter substrate-binding protein (osmoprotectant uptake) | \bproX\b |
|  | *opuA* | OpuA ABC transporter (compatible solute uptake) | \bopuA\b |
|  | *opuA* | OpuA ABC transporter (compatible solute uptake) | \bopuAA\b |
|  | *opuA* | OpuA ABC transporter (compatible solute uptake) | \bopuAB\b |
|  | *opuA* | OpuA ABC transporter (compatible solute uptake) | \bopuAC\b |
|  | *opuB* | OpuB ABC transporter (compatible solute uptake) | \bopuB\b |
|  | *opuB* | OpuB ABC transporter (compatible solute uptake) | \bopuBA\b |
|  | *opuB* | OpuB ABC transporter (compatible solute uptake) | \bopuBB\b |
|  | *opuB* | OpuB ABC transporter (compatible solute uptake) | \bopuBC\b |
|  | *opuC* | OpuC ABC transporter (compatible solute uptake) | \bopuC\b |
|  | *opuC* | OpuC ABC transporter (compatible solute uptake) | \bopuCA\b |
|  | *opuC* | OpuC ABC transporter (compatible solute uptake) | \bopuCB\b |
|  | *opuC* | OpuC ABC transporter (compatible solute uptake) | \bopuCC\b |
|  | *opuD* | OpuD BCCT transporter (glycine betaine uptake) | \bopuD\b |
|  | *gbuA* | GbuABC transporter component (glycine betaine uptake) | \bgbuA\b |
|  | *gbuB* | GbuABC transporter component (glycine betaine uptake) | \bgbuB\b |
|  | *gbuC* | GbuABC transporter component (glycine betaine uptake) | \bgbuC\b |
|  | *betT* | Choline transporter (glycine betaine precursor uptake) | \bbetT\b |
|  | *betT* | Choline transporter (glycine betaine precursor uptake) | \bcholine\s+transporter\b |
|  | *betL* | Betaine transporter (BCCT family) | \bbetL\b |
|  | *busA* | Glycine betaine/carnitine/choline ABC transporter (BusA) | \bbusA\b |
|  | *busB* | Glycine betaine/carnitine/choline ABC transporter (BusB) | \bbusB\b |
|  | *busC* | Glycine betaine/carnitine/choline ABC transporter (BusC) | \bbusC\b |
|  | *proP* | Osmoregulatory MFS transporter (proline/betaine uptake) | \bproP\b |
|  | *putP* | Na+/proline symporter (proline uptake) | \bputP\b |
|  | *putP* | Na+/proline symporter (proline uptake) | \bproline\s+permease\b |
|  | *putP* | Na+/proline symporter (proline uptake) | \bNa\+/?proline\s+symporter\b |
|  | *ectT* | Ectoine transporter (uptake) | \bectT\b |
|  | *aqpZ* | Aquaporin Z (water channel) | \baqpZ\b |
|  | *ectA* | Ectoine biosynthesis enzyme (EctA) | \bectA\b |
|  | *ectB* | Ectoine biosynthesis enzyme (EctB) | \bectB\b |
|  | *ectC* | Ectoine biosynthesis enzyme (EctC) | \bectC\b |
|  | *ectD* | Ectoine hydroxylase (5-hydroxyectoine synthesis) | \bectD\b |
|  | *betA* | Choline dehydrogenase (glycine betaine synthesis) | \bbetA\b |
|  | *betA* | Choline dehydrogenase (glycine betaine synthesis) | \bcholine\s+dehydrogenase\b |
|  | *betB* | Betaine aldehyde dehydrogenase (glycine betaine synthesis) | \bbetB\b |
|  | *betB* | Betaine aldehyde dehydrogenase (glycine betaine synthesis) | \bbetaine\s+aldehyde\s+dehydrogenase\b |
|  | *gbsA* | Betaine aldehyde dehydrogenase (Bacillus-type; glycine betaine synthesis) | \bgbsA\b |
|  | *gbsB* | Choline dehydrogenase (Bacillus-type; glycine betaine synthesis) | \bgbsB\b |
|  | *otsA* | Trehalose-6-phosphate synthase (trehalose biosynthesis) | \botsA\b |
|  | *otsB* | Trehalose-6-phosphate phosphatase (trehalose biosynthesis) | \botsB\b |
|  | *treS* | Trehalose synthase (trehalose metabolism) | \btreS\b |
|  | *treS* | Trehalose synthase (trehalose metabolism) | \btrehalose\s*synthase\b |
|  | *treY* | Maltooligosyl trehalose synthase (TreY; trehalose pathway) | \btreY\b |
|  | *treZ* | Maltooligosyl trehalose trehalohydrolase (TreZ; trehalose pathway) | \btreZ\b |
|  | *treB* | Trehalose PTS transporter EIIBC component (uptake) | \btreB\b |
|  | *treB* | Trehalose PTS transporter EIIBC component (uptake) | \btrehalose\s*PTS\b |
|  | *treC* | Trehalose-6-phosphate hydrolase (TreC; trehalose utilization) | \btreC\b |
|  | *mscL* | Mechanosensitive channel of large conductance (hypo-osmotic shock) | \bmscL\b |
|  | *mscS* | Mechanosensitive channel of small conductance (hypo-osmotic shock) | \bmscS\b |
|  | *mscK* | Mechanosensitive channel (K+) (hypo-osmotic shock) | \bmscK\b |
| Alkaline stress response | *nhaA* | Na+/H+ antiporter (pH and sodium homeostasis) | \bnhaA\b |
|  | *nhaA* | Na+/H+ antiporter (pH and sodium homeostasis) | \bNa\+/?H\+\s*antiporter\b |
|  | *nhaB* | Na+/H+ antiporter | \bnhaB\b |
|  |  | Na+/H+ antiporter | \bnhaC\b |
|  | *nhaP* | Na+/H+ antiporter | \bnhaP\b |
|  | *ybaL* | ChaA-like antiporter (Na+/Ca2+/H+ antiporter family) | \bybaL\b |
|  | *nhaR* | NhaA regulator (transcriptional regulator) | \bnhaR\b |
|  | *nhaR* | NhaA regulator (transcriptional regulator) | \bNhaA\s*regulator\b |
|  | *mrpA* | Mrp/Mnh Na+/H+ antiporter subunit A (CPA3 complex) | \bmrpA\b |
|  | *mrpA* | Mrp/Mnh Na+/H+ antiporter subunit A (CPA3 complex) | \bmnhA\b |
|  | *mrpB* | Mrp/Mnh antiporter subunit B | \bmrpB\b |
|  | *mrpB* | Mrp/Mnh antiporter subunit B | \bmnhB\b |
|  | *mrpC* | Mrp/Mnh antiporter subunit C | \bmrpC\b |
|  | *mrpC* | Mrp/Mnh antiporter subunit C | \bmnhC\b |
|  | *mrpD* | Mrp/Mnh antiporter subunit D | \bmrpD\b |
|  | *mrpD* | Mrp/Mnh antiporter subunit D | \bmnhD\b |
|  | *mrpE* | Mrp/Mnh antiporter subunit E | \bmrpE\b |
|  | *mrpE* | Mrp/Mnh antiporter subunit E | \bmnhE\b |
|  | *mrpF* | Mrp/Mnh antiporter subunit F | \bmrpF\b |
|  | *mrpF* | Mrp/Mnh antiporter subunit F | \bmnhF\b |
|  | *mrpG* | Mrp/Mnh antiporter subunit G | \bmrpG\b |
|  | *mrpG* | Mrp/Mnh antiporter subunit G | \bmnhG\b |
|  | *trkH* | Trk potassium uptake system membrane protein | \btrkH\b |
|  | *trkA* | Trk potassium uptake system NAD-binding subunit | \btrkA\b |
|  | *trkG* | Trk potassium uptake system membrane protein (alternative) | \btrkG\b |
|  | *kup* | K+ uptake permease (Kup/TrkD) | \bkup\b |
|  | *kup* | K+ uptake permease (Kup/TrkD) | \btrkD\b |
|  | *kdpA* | High-affinity K+ transporter (Kdp system; permease) | \bkdpA\b |
|  | *kdpB* | High-affinity K+ transporter ATPase (Kdp system) | \bkdpB\b |
|  | *kdpC* | High-affinity K+ transporter component (Kdp system) | \bkdpC\b |
|  | *kdpD* | Sensor kinase (Kdp two-component system) | \bkdpD\b |
|  | *kdpD* | Sensor kinase (Kdp two-component system) | \bsensor\s*kinase\b.*\bkdp\b |
|  | *kdpE* | Response regulator (Kdp two-component system) | \bkdpE\b |
|  | *kdpE* | Response regulator (Kdp two-component system) | \bresponse\s*regulator\b.*\bkdp\b |
|  | *kefB* | K+ efflux system (pH homeostasis) | \bkefB\b |
|  | *kefC* | K+ efflux system (pH homeostasis) | \bkefC\b |
|  | *clcA* | Cl-/H+ antiporter (acid/base homeostasis) | \bclcA\b |
|  | *nqrA* | Na+-translocating NADH:quinone oxidoreductase subunit A | \bnqrA\b |
|  | *nqrB* | Na+-translocating NADH:quinone oxidoreductase subunit B | \bnqrB\b |
|  | *nqrC* | Na+-translocating NADH:quinone oxidoreductase subunit C | \bnqrC\b |
|  | *nqrD* | Na+-translocating NADH:quinone oxidoreductase subunit D | \bnqrD\b |
|  | *nqrE* | Na+-translocating NADH:quinone oxidoreductase subunit E | \bnqrE\b |
|  | *nqrF* | Na+-translocating NADH:quinone oxidoreductase subunit F | \bnqrF\b |
|  | *chaA* | Ca2+/H+ (or Na+/H+) antiporter (pH homeostasis) | \bchaA\b |
|  | *chaA* | Ca2+/H+ (or Na+/H+) antiporter (pH homeostasis) | \bCa2\+/?H\+\s*antiporter\b |
|  | *chaB* | ChaB antiporter-associated protein | \bchaB\b |
| Cold stress response | *cspA* | Cold shock protein (CspA family; RNA chaperone) | \bcspA\b |
|  | *cspB* | Cold shock protein (Csp family) | \bcspB\b |
|  | *cspC* | Cold shock protein (Csp family) | \bcspC\b |
|  | *cspD* | Cold shock protein (Csp family) | \bcspD\b |
|  | *cspE* | Cold shock protein (Csp family) | \bcspE\b |
|  | *cspG* | Cold shock protein (Csp family) | \bcspG\b |
|  | *cspI* | Cold shock protein (Csp family) | \bcspI\b |
|  | *csdA* | DEAD-box RNA helicase (cold adaptation) | \bdeaD\b |
|  | *csdA* | DEAD-box RNA helicase (cold adaptation) | \bcsdA\b |
|  | *rhlE* | RNA helicase (cold adaptation) | \brhlE\b |
|  | *rhlB* | RNA helicase (cold adaptation) | \brhlB\b |
|  | *rbfA* |  | \brbfA\b |
|  | *yfiA_raiA* | Ribosome-associated inhibitor (stationary/cold response) | \byfiA\b |
|  | *yfiA_raiA* | Ribosome-associated inhibitor (stationary/cold response) | \braiA\b |
|  | *yfiA_raiA* | Ribosome-associated inhibitor (stationary/cold response) | \bpsrA\b |
|  | *pnp* | Polyribonucleotide nucleotidyltransferase (PNPase; RNA processing) | \bpnp\b |
|  | *pnp* | Polyribonucleotide nucleotidyltransferase (PNPase; RNA processing) | \bpolyribonucleotide\s+nucleotidyltransferase\b |
|  | *pnp* | Polyribonucleotide nucleotidyltransferase (PNPase; RNA processing) | \bPNPase\b |
|  | *rnr* | RNase R (exoribonuclease; RNA processing) | \brnr\b |
|  | *rnr* | RNase R (exoribonuclease; RNA processing) | \brnase\s*r\b |
|  | *rnr* | RNase R (exoribonuclease; RNA processing) | \bribonuclease\s*r\b |
|  | *desA* | Fatty acid desaturase (membrane fluidity) | \bdesA\b |
|  | *desB* | Fatty acid desaturase | \bdesB\b |
|  | *desK* | Membrane fluidity sensor histidine kinase (DesK) | \bdesK\b |
|  | *desK* | Membrane fluidity sensor histidine kinase (DesK) | \bhistidine\s+kinase\b.*des |
|  | *desK* | Membrane fluidity sensor histidine kinase (DesK) | \bmembrane\s+fluidity\s+sensor\b |
|  | *desR* | Response regulator for desaturase genes (DesR) | \bdesR\b |
|  | *desR* | Response regulator for desaturase genes (DesR) | \bresponse\s+regulator\b.*des |
|  | *opuA* | Compatible solute transporter (cold/osmoprotection) | \bopuA\b |
|  | *opuC* | Compatible solute transporter (cold/osmoprotection) | \bopuC\b |
|  | *proP* | Osmoregulatory transporter (cold/osmoprotection) | \bproP\b |
|  | *betL* | Betaine transporter (cold/osmoprotection) | \bbetL\b |
|  | *cshA* | Cold shock helicase (CshA) | \bcshA\b |
|  | *cshB* | Cold shock helicase (CshB) | \bcshB\b |
|  | *csrA* | Global RNA-binding regulator (carbon storage regulator; stress response) | \bcsrA\b |
|  | *csrA* | Global RNA-binding regulator (carbon storage regulator; stress response) | \brsmA\b |
| Heat stress tolerance | *dnaK* | Chaperone Hsp70 (protein folding; heat stress) | \bdnaK\b |
|  | *dnaK* | Chaperone Hsp70 (protein folding; heat stress) | \bhsp70\b |
|  | *dnaJ* | Co-chaperone DnaJ (J-domain; heat stress) | \bdnaJ\b |
|  | *dnaJ* | Co-chaperone DnaJ (J-domain; heat stress) | \bj-?domain\b |
|  | *grpE* | Nucleotide exchange factor for DnaK (heat stress) | \bgrpE\b |
|  | *groEL* | Chaperonin GroEL/Hsp60 (protein folding) | \bgroEL\b |
|  | *groEL* | Chaperonin GroEL/Hsp60 (protein folding) | \bhsp60\b |
|  | *groEL* | Chaperonin GroEL/Hsp60 (protein folding) | \bcpn60\b |
|  | *groES* | Co-chaperonin GroES/Hsp10 | \bgroES\b |
|  | *groES* | Co-chaperonin GroES/Hsp10 | \bhsp10\b |
|  | *groES* | Co-chaperonin GroES/Hsp10 | \bcpn10\b |
|  | *tig* | Trigger factor (ribosome-associated chaperone) | \btig\b |
|  | *tig* | Trigger factor (ribosome-associated chaperone) | \btrigger\s*factor\b |
|  | *htpG* | Hsp90 family chaperone (HtpG) | \bhtpG\b |
|  | *htpG* | Hsp90 family chaperone (HtpG) | \bhsp90\b |
|  | *hslO* | Hsp33 family chaperone (oxidative/heat stress) | \bhslO\b |
|  | *hslO* | Hsp33 family chaperone (oxidative/heat stress) | \bhsp33\b |
|  | *clpP* | Clp protease proteolytic subunit (protein quality control) | \bclpP\b |
|  | *clpX* | Clp protease ATPase subunit (protein quality control) | \bclpX\b |
|  | *clpC* | Clp ATPase chaperone (protein quality control) | \bclpC\b |
|  | *clpE* | Clp ATPase chaperone (protein quality control) | \bclpE\b |
|  | *ftsH* | Membrane-anchored AAA+ protease (heat stress) | \bftsH\b |
|  | *lon* | Lon protease (protein quality control) | \blon\b |
|  | *lon* | Lon protease (protein quality control) | \blon\s+protease\b |
|  | *hrcA* | Heat shock repressor (HrcA) | \bhrcA\b |
|  | *ctsR* | Stress response regulator (CtsR; class III heat shock) | \bctsR\b |
|  | *hsp20* | Small heat shock protein (Hsp20/alpha-crystallin family) | \bhsp20\b |
|  | *sigB* | Alternative sigma factor (general stress response) | \bsigB\b |
|  | *sigB* | Alternative sigma factor (general stress response) | \bsigma\s*B\b |
|  | *sigB* | Alternative sigma factor (general stress response) | \brpoF\b |
|  | *hspR* | Heat shock regulator (HspR) | \bhspR\b |
|  | *clpL* | Clp ATPase (LAB stress-associated chaperone) | \bclpL\b |
|  | *clpB* | ClpB disaggregase (heat stress) | \bclpB\b |
| Immunomodulation | *ltaS* | Lipoteichoic acid synthase (cell envelope; TLR2-active LTA) | \bltaS\b |
|  | *ltaS* | Lipoteichoic acid synthase (cell envelope; TLR2-active LTA) | \blipoteichoic\s+acid\s+synthetase\b |
|  | *tagO* | Teichoic acid biosynthesis initiator (WTA/LTA pathway) | \btagO\b |
|  | *tagO* | Teichoic acid biosynthesis initiator (WTA/LTA pathway) | \bteichoic\s+acid\b.*\binitiat(?:or\|ing)\b |
|  | *tagB* | Teichoic acid biosynthesis protein (WTA) | \btagB\b |
|  | *tagF* | Teichoic acid polymerase (WTA) | \btagF\b |
|  | *tarO* | Teichoic acid biosynthesis initiator (alternative naming) | \btarO\b |
|  | *ltaP* | Lipoteichoic acid primase/polymerase (Firmicutes; LTA pathway) | \bltaP\b |
|  | *lytR* | LytR-CpsA-Psr family regulator (cell envelope/teichoic acid attachment) | \blytR\b |
|  | *dltA* | Teichoic acid D-alanylation (DltA; surface charge modulation) | \bdltA\b |
|  | *dltB* | Teichoic acid D-alanylation (DltB; membrane component) | \bdltB\b |
|  | *dltC* | Teichoic acid D-alanylation (DltC; carrier protein) | \bdltC\b |
|  | *dltD* | Teichoic acid D-alanylation (DltD; accessory protein) | \bdltD\b |
|  | *slpA* | S-layer protein (surface layer; host interaction) | \bslpA\b |
|  | *mub* | Mucus-binding protein (MucBP/MUB; adhesion) | \bmub\b |
|  | *mapA* | Mucus adhesion-promoting protein (colonization factor) | \bmapA\b |
|  | *srtA* | Sortase A (LPXTG-anchored surface protein assembly) | \bsrtA\b |
|  | *srtA* | Sortase A (LPXTG-anchored surface protein assembly) | \bsortase\s*A\b |
|  | LPXTG_protein | LPXTG/LPQTG motif (sortase-anchored surface proteins) | \bLPXTG\b |
|  | LPXTG_protein | LPXTG/LPQTG motif (sortase-anchored surface proteins) | \bLPQTG\b |
|  | *spa* | Sortase-dependent pili/pilin proteins (adhesion/biofilm) | \bspaA\b |
|  | *spa* | Sortase-dependent pili/pilin proteins (adhesion/biofilm) | \bspaB\b |
|  | *spa* | Sortase-dependent pili/pilin proteins (adhesion/biofilm) | \bspaC\b |
|  | *spa* | Sortase-dependent pili/pilin proteins (adhesion/biofilm) | \bspaD\b |
|  | *spa* | Sortase-dependent pili/pilin proteins (adhesion/biofilm) | \bpilin\b |
|  | *spa* | Sortase-dependent pili/pilin proteins (adhesion/biofilm) | \bpilus\b |
|  | *wzb* | Tyrosine phosphatase (EPS/capsule regulation) | \bwzb\b |
|  | *wzc* | Tyrosine kinase (EPS/capsule regulation) | \bwzc\b |
|  | *wzx* | Polysaccharide flippase (EPS/O-antigen/capsule export) | \bwzx\b |
|  | *wzy* | Polysaccharide polymerase (EPS/O-antigen/capsule assembly) | \bwzy\b |
|  | *ugd* | UDP-glucose dehydrogenase (EPS precursor biosynthesis) | \bugd\b |
|  | *galU* | UTP-glucose-1-phosphate uridylyltransferase (EPS precursor biosynthesis) | \bgalU\b |
|  | *fliC* | Flagellin (TLR5 ligand; motility/immune signaling) | \bfliC\b |
|  | *fliC* | Flagellin (TLR5 ligand; motility/immune signaling) | \bflagellin\b |
|  | *nlpC_P60* | NlpC/P60 endopeptidase (peptidoglycan remodeling; p40/p75-like) | \bNlpC/?P60\b |
|  | *CHAP* | CHAP-domain peptidoglycan hydrolase (cell wall remodeling) | \bCHAP\b\s*(?:domain\|endopeptidase)? |
|  | M23_peptidase | M23 metallopeptidase/lysostaphin-like (cell wall cleavage) | \bM23\b\s*(?:peptidase\|endopeptidase)? |
|  | *msp1_p75* | p75-like secreted/muralytic protein (LGG-like immunomodulation) | \bmsp1\b |
|  | *msp1_p75* | p75-like secreted/muralytic protein (LGG-like immunomodulation) | \bp75\b |
|  | *msp2_p40* | p40-like secreted protein (LGG-like immunomodulation) | \bmsp2\b |
|  | *msp2_p40* | p40-like secreted protein (LGG-like immunomodulation) | \bp40\b |
|  | *bsh* | Bile salt hydrolase (bile detoxification; gut adaptation) | \bbsh\b |
|  | *bsh* | Bile salt hydrolase (bile detoxification; gut adaptation) | \bbile\s+(salt\|acid)\s+hydrolase\b |
|  | *bsh* | Bile salt hydrolase (bile detoxification; gut adaptation) | \bcholoylglycine\s+hydrolase\b |
|  | salivaricin | Salivaricin bacteriocin genes | \bsalA\b |
|  | salivaricin | Salivaricin bacteriocin genes | \bsalivaricin\b |
|  | bacteriocin_generic | Bacteriocin/lantibiotic (antimicrobial peptide; generic) | \bbacteriocin\b |
|  | bacteriocin_generic | Bacteriocin/lantibiotic (antimicrobial peptide; generic) | \blantibiotic\b |
|  | *arcA* | Arginine deiminase (ADI pathway; acid stress/immune modulation context) | \barcA\b |
|  | *arcA* | Arginine deiminase (ADI pathway; acid stress/immune modulation context) | \barginine\s+deiminase\b |
|  | *arcB* | Ornithine carbamoyltransferase (ADI pathway) | \barcB\b |
|  | *arcB* | Ornithine carbamoyltransferase (ADI pathway) | \bornithine\s+carbamoyltransferase\b |
|  | *arcC* | Carbamate kinase (ADI pathway) | \barcC\b |
|  | *arcC* | Carbamate kinase (ADI pathway) | \bcarbamate\s+kinase\b |
|  | *arcD* | Arginine/ornithine antiporter (ADI pathway) | \barcD\b |
|  | *arcD* | Arginine/ornithine antiporter (ADI pathway) | \barginine/ornithine\s+antiporter\b |

**Table S3** Validation of PMG using sequence-based confirmation.

| Marker | Regex candidates | BLAST-confirmed | Concordance (%) |
| --- | --- | --- | --- |
| *bsh* | 3,278 | 3,205 | 97.9 |
| *gadB* | 1,815 | 1,815 | 100.0 |
| *atpA* | 3,812 | 3,812 | 100.0 |
| *srtA* | 4,144 | 4,092 | 98.7 |
| *dltA* | 3,448 | 3,448 | 100.0 |
| *clpP* | 1,876 | 1,876 | 100.0 |
| *dnaK* | 3,817 | 3,815 | 99.9 |

**Table S4** CAZyme profiling across 35 former *Lactobacillus* species.

| Species | Genome number | CAZyme family | | | | | | Total enzyme |
| --- | --- | --- | --- | --- | --- | --- | --- | --- |
|  |  | GH | GT | PL | CE | AA | CBM |  |
| *A. kunkeei* | 106 | 3,234 | 3,933 | 0 | 212 | 106 | 771 | 8,256 |
| *C. paralimentarius* | 12 | 909 | 588 | 0 | 26 | 25 | 140 | 1,688 |
| *F. fructivorans* | 13 | 275 | 475 | 0 | 13 | 12 | 119 | 894 |
| *F. sanfranciscensis* | 51 | 1,165 | 1,701 | 0 | 51 | 4 | 436 | 3,357 |
| *L. paracasei* | 17 | 1,105 | 971 | 18 | 51 | 15 | 345 | 2,505 |
| *L. rhamnosus* | 331 | 21,249 | 18,877 | 223 | 992 | 0 | 6,219 | 47,560 |
| *L. argentoratensis* | 33 | 2,816 | 2,414 | 0 | 121 | 70 | 887 | 6,308 |
| *L. paraplantarum* | 24 | 2,186 | 1,576 | 0 | 73 | 66 | 634 | 4,535 |
| *L. pentosus* | 111 | 10,841 | 8,252 | 0 | 414 | 216 | 2,847 | 22,570 |
| *L. plantarum* | 1,233 | 120,147 | 88,753 | 0 | 3,748 | 3,718 | 32,194 | 248,560 |
| *L. acidophilus* | 93 | 4,667 | 3,790 | 0 | 93 | 0 | 1,206 | 9,756 |
| *L. crispatus* | 112 | 5,580 | 4,023 | 2 | 208 | 25 | 1,323 | 11,161 |
| *L. delbrueckii* | 60 | 2,208 | 2,534 | 0 | 62 | 68 | 627 | 5,499 |
| *L. gasseri* | 223 | 9,601 | 8,753 | 0 | 231 | 33 | 2,190 | 20,808 |
| *L. helveticus* | 93 | 3,497 | 3,524 | 0 | 93 | 0 | 898 | 8,012 |
| *L. iners* | 73 | 1,711 | 1,524 | 0 | 104 | 0 | 720 | 4,059 |
| *L. intestinalis* | 10 | 342 | 406 | 0 | 20 | 8 | 108 | 884 |
| *L. jensenii* | 63 | 2,012 | 2,349 | 0 | 63 | 55 | 503 | 4,982 |
| *L. johnsonii* | 35 | 1,476 | 1,668 | 0 | 42 | 9 | 370 | 3,565 |
| *L. mulieris* | 43 | 1,417 | 1,466 | 0 | 43 | 43 | 349 | 3,318 |
| *L. paragasseri* | 71 | 3,358 | 3,357 | 0 | 113 | 46 | 772 | 7,646 |
| *L. taiwanensis* | 29 | 1,117 | 1,429 | 0 | 31 | 29 | 272 | 2,878 |
| *L. curvatus* | 67 | 3,141 | 2,780 | 0 | 208 | 71 | 1035 | 7,235 |
| *L. sakei* | 15 | 564 | 629 | 0 | 53 | 30 | 210 | 1,486 |
| *L. hilgardii* | 10 | 614 | 405 | 0 | 59 | 7 | 174 | 1,259 |
| *L. parabuchneri* | 27 | 1,553 | 1,150 | 0 | 136 | 27 | 499 | 3,365 |
| *L. brevis* | 120 | 5,832 | 5,273 | 0 | 436 | 167 | 2,514 | 14,222 |
| *L. agilis* | 37 | 1,581 | 2,391 | 0 | 77 | 36 | 620 | 4,705 |
| *L. aviarius* | 23 | 664 | 932 | 0 | 49 | 0 | 238 | 1,883 |
| *L. murinus* | 18 | 1,046 | 858 | 0 | 63 | 17 | 359 | 2,343 |
| *L. salivarius* | 240 | 7,640 | 14,621 | 0 | 240 | 228 | 3,292 | 26,021 |
| *L. fermentum* | 74 | 2,435 | 2,915 | 0 | 104 | 74 | 703 | 6,231 |
| *L. mucosae* | 16 | 621 | 729 | 0 | 11 | 17 | 205 | 1,583 |
| *L. reuteri* | 310 | 12,419 | 13,665 | 0 | 3 | 214 | 3,772 | 30,073 |
| *L. vaginalis* | 20 | 658 | 780 | 0 | 0 | 20 | 174 | 1,632 |

**Table S5** Antimicrobial-resistance genes detected in 3,813 strains of the *Lactobacillus* genus.

| Drug-resistance class | Gene | Function of the mechanism | Species |
| --- | --- | --- | --- |
| Tetracyclines | *tet(M)* | Ribosomal protection protein conferring tetracycline resistance | *C. paralimentarius* (8.3%)*, L. paracasei* (17.6%)*, L. rhamnosus* (0.3%), *L. argentoratensis* (6.1%)*, L. pentosus* (0.9%)*, L. plantarum* (0.6%), *L. gasseri* (0.4%), *L. iners* (74.0%), *L. mulieris* (2.3%), *L. curvatus* (3.0%), *L. sakei* (6.7%), *L. brevis* (1.7%), *L. agilis* (43.2%), *L. salivarius* (12.9%), *L. reuteri* (1.0%) |
|  | *tet(L)* | Tetracycline efflux pump | *L. agilis* (24.3%)*, L. salivarius* (10.4%), *L. reuteri* (0.6%) |
|  | *tet(W)* | Ribosomal protection protein conferring tetracycline resistance | *L. crispatus* (37.5%)*, L. delbrueckii* (3.3%), *L. johnsonii* (14.3%), *L. aviarius* (43.5%), *L. mucosae* (18.8%), *L. reuteri* (15.5%) |
|  | *tet(O)* | Ribosomal protection protein conferring tetracycline resistance | *L. aviarius* (4.3%) |
|  | *tet(O/W/32/O/W/O)* | Mosaic ribosomal protection protein | *L. johnsonii* (11.4%) |
|  | *tet(S)* | Ribosomal protection protein conferring tetracycline resistance | *L. argentoratensis* (3.0%) |
| Macrolides-Lincosamides-Streptogramin B (MLS-B) | *erm(A)* | 23S rRNA methyltransferase; macrolide–lincosamide resistance | *L. crispatus* (0.9%)*, L. salivarius* (1.7%) |
|  | *erm(B)* | 23S rRNA methyltransferase; macrolide–lincosamide resistance | *A. kunkeei* (0.9%)*, C. paralimentarius* (8.3%)*, L. argentoratensis* (3.0%)*, L. crispatus* (4.5%)*, L. iners* (21.9%), *L. johnsonii* (20.0%), *L. agilis* (2.7%), *L. salivarius* (2.9%), *L. reuteri* (3.5%), *L. vaginalis* (5.0%) |
|  | *erm(C)* |  | *L. vaginalis* (1.7%) |
|  | *erm(T)* |  | *L. crispatus* (8.9%), *L. johnsonii* (2.9%), *L. salivarius* (0.4%), *L. reuteri* (0.3%) |
| Macrolides | *mef(A)* | Macrolide efflux pump | *L. iners* (21.9%) |
|  | *msr(D)* | ABC-F ribosomal protection protein; macrolide resistance | *L. iners* (21.9%) |
| Phenicols | *cat* | Chloramphenicol acetyltransferase | *L. argentoratensis* (0.3%)*, L. crispatus* (0.9%)*, L. salivarius* (2.5%) |
|  | *cat(pC194)* | Chloramphenicol acetyltransferase | *A. kunkeei* (0.9%)*, L. plantarum* (2.2%), *L. salivarius* (2.4%), *L. reuteri* (0.3%) |
|  | *fexA* | Phenicol efflux transporter | *L. johnsonii* (2.9%)*, L. salivarius* (0.4%)*, L. reuteri* (1.0%) |
|  | *fexB* | Phenicol efflux transporter | *L. plantarum* (0.1%)*, L. salivarius* (1.7%), *L. reuteri* (0.3%), *L. vaginalis* (5.0%) |
| Diaminopyrimidines | *dfrE* | Trimethoprim-resistant dihydrofolate reductase | *L. argentoratensis* (3.0%), *L. plantarum* (0.1%) |
|  | *dfrG* | Trimethoprim-resistant dihydrofolate reductase | *L. plantarum* (0.2%)*, L. reuteri* (0.3%) |
| Aminoglycosides | *str* | Streptomycin resistance protein | *C. paralimentarius* (8.3%)*, L. argentoratensis* (9.1%), *L. brevis* (4.2%) |
|  | *aadD* | Aminoglycoside adenyltransferase | *L. salivarius* (0.4%)*, L. reuteri* (0.3%) |
|  | *ant(6)-Ia* | Aminoglycoside nucleotidyltransferase | *L. argentoratensis* (3.0%)*, L. plantarum* (0.2%), *L. salivarius* (1.7%)*, L. reuteri* (0.3%) |
|  | *ant(9)-Ia* | Aminoglycoside nucleotidyltransferase | *L. salivarius* (1.3%) |
|  | *aph(3′)-Ia* | Aminoglycoside phosphotransferase | *F. sanfranciscensis* (2.0%)*, L. rhamnosus* (0.3%), *L. plantarum* (0.1%) |
|  | *aac(6′)-aph(2′′)* | Bifunctional aminoglycoside-modifying enzyme | *L. plantarum* (0.1%)*, L. salivarius* (1.7%), *L. reuteri* (1.3%), L. helveticus (1.1%), |
| Lincosamides | *lnu(A)* | Lincosamide nucleotidyltransferase | *L. argentoratensis* (3.0%), *L. plantarum* (0.1%), *L. crispatus* (8.0%), *L. johnsonii* (2.9%), *L. curvatus* (1.5%), *L. sakei* (6.7%), *L. aviarius* (8.7%), *L. salivarius* (4.6%), *L. reuteri* (7.4%) |
|  | *lnu(C)* | Lincosamide nucleotidyltransferase | *L. crispatus* (6.3%)*, L. johnsonii* (2.9%), *L. agilis* (32.4%), *L. salivarius* (2.9%), *L. mucosae* (18.8%) |
| Streptogramin | *vat(A)* | Streptogramin A acetyltransferase | *L. plantarum* (0.9%)*, L. brevis* (2.5%) |
|  | *vat(E)* | Streptogramin A acetyltransferase | *L. argentoratensis* (9.1%), *L. plantarum* (0.9%), *L. crispatus* (13.4%), *L. brevis* (5.8%),  *L. agilis* (21.6%), *L. salivarius* (3.3%), *L. reuteri* (1.9%) |
|  | *vgb(A)* | Streptogramin B lyase | *L. plantarum* (0.9%), *L. brevis* (2.5%) |
| β-lactams | *blaTEM-116* | Class A β-lactamase | *L. rhamnosus* (0.6%)*, L. plantarum* (0.1%), *L. helveticus* (2.2%) |
|  | *blaTEM-171* | Class A β-lactamase | *L. salivarius* (0.4%) |
| Carbapenems | *blaNDM-5* | Carbapenemase | *L. plantarum* (0.1%) |
| Phenicol, oxazolidinone, tetracycline | *poxtA* | Modification of lipid A | *L. plantarum* (0.1%), *L. salivarius* (1.7%), *L. reuteri* (0.3%) |
| Nitrate reductase | *narA,* | Nitrate reductase subunits | *L. curvatus* (1.5%) |
|  | *narB* | Nitrate reductase subunits | *L. curvatus* (1.5%) |

**Table S6** Metadata summary of genomes included in this study.

| Accession | Species | Strain | Isolation source |
| --- | --- | --- | --- |
| GCF_000741865.1 | *Apilactobacillus kunkeei* | MP2 | Animal |
| GCF_000830375.1 | *Apilactobacillus kunkeei* | AR114 | Animal |
| GCF_001281165.1 | *Apilactobacillus kunkeei* | Fhon2 | Animal |
| GCF_001281205.1 | *Apilactobacillus kunkeei* | LAko | Animal |
| GCF_001281215.1 | *Apilactobacillus kunkeei* | LAla | Animal |
| GCF_001281225.1 | *Apilactobacillus kunkeei* | LAan | Animal |
| GCF_001281285.1 | *Apilactobacillus kunkeei* | LAni | Animal |
| GCF_001308185.1 | *Apilactobacillus kunkeei* | LMbe | Animal |
| GCF_001308195.1 | *Apilactobacillus kunkeei* | LMbo | Others |
| GCF_001308205.1 | *Apilactobacillus kunkeei* | LAdo | Animal |
| GCF_001308215.1 | *Apilactobacillus kunkeei* | LAnu | Animal |
| GCF_001314945.1 | *Apilactobacillus kunkeei* | MP2 | Animal |
| GCF_001421115.1 | *Apilactobacillus kunkeei* | LAce | Animal |
| GCF_001421135.1 | *Apilactobacillus kunkeei* | LAfl | Animal |
| GCF_001949975.2 | *Apilactobacillus kunkeei* | FF30-6 | Others |
| GCF_005930915.1 | *Apilactobacillus kunkeei* | UASWS1867-NN5 | Animal |
| GCF_005930925.1 | *Apilactobacillus kunkeei* | UASWS1869-NN19 | Animal |
| GCF_005930935.1 | *Apilactobacillus kunkeei* | UASWS1870-NN20 | Animal |
| GCF_005930975.1 | *Apilactobacillus kunkeei* | UASWS1868-NN17 | Animal |
| GCF_006493415.1 | *Apilactobacillus kunkeei* | KA_01 | Animal |
| GCF_006493615.1 | *Apilactobacillus kunkeei* | 3L | Animal |
| GCF_009910775.1 | *Apilactobacillus kunkeei* | O29 | Others |
| GCF_014323645.1 | *Apilactobacillus kunkeei* | Dan39 | Animal |
| GCF_019575995.1 | *Apilactobacillus kunkeei* | DSMZ 12361 | Others |
| GCF_019576055.1 | *Apilactobacillus kunkeei* | 7K4AA | Enviroment |
| GCF_019718775.1 | *Apilactobacillus kunkeei* | 7K11C | Enviroment |
| GCF_023218665.1 | *Apilactobacillus kunkeei* | R13 | Animal |
| GCF_023218695.1 | *Apilactobacillus kunkeei* | R5 | Animal |
| GCF_023218705.1 | *Apilactobacillus kunkeei* | 26 | Animal |
| GCF_023218755.1 | *Apilactobacillus kunkeei* | 9 | Animal |
| GCF_023218775.1 | *Apilactobacillus kunkeei* | 10 | Animal |
| GCF_023218815.1 | *Apilactobacillus kunkeei* | 3 | Animal |
| GCF_026229345.1 | *Apilactobacillus kunkeei* | LB24 | Animal |
| GCF_026428215.1 | *Apilactobacillus kunkeei* | IBH001 | Animal |
| GCF_030388685.1 | *Apilactobacillus kunkeei* | S1 | Enviroment |
| GCF_035919605.1 | *Apilactobacillus kunkeei* | GYUN-333 | Animal |
| GCF_040364485.1 | *Apilactobacillus kunkeei* | FMO35 | Animal |
| GCF_040366445.1 | *Apilactobacillus kunkeei* | NRIC 0776 | Animal |
| GCF_047551945.1 | *Apilactobacillus kunkeei* | CA1 | Animal |
| GCF_052412395.1 | *Apilactobacillus kunkeei* | NFICC2128 | Animal |
| GCF_946887465.1 | *Apilactobacillus kunkeei* | G0405 | Animal |
| GCF_946887475.1 | *Apilactobacillus kunkeei* | G0103 | Animal |
| GCF_946887485.1 | *Apilactobacillus kunkeei* | G0802 | Animal |
| GCF_946887495.1 | *Apilactobacillus kunkeei* | G0804 | Animal |
| GCF_946887505.1 | *Apilactobacillus kunkeei* | G0101 | Animal |
| GCF_946887515.1 | *Apilactobacillus kunkeei* | G0406 | Animal |
| GCF_946887545.1 | *Apilactobacillus kunkeei* | H4B2-04J | Animal |
| GCF_946887555.1 | *Apilactobacillus kunkeei* | A2101 | Animal |
| GCF_946887565.1 | *Apilactobacillus kunkeei* | H3B1-01J | Animal |
| GCF_946887585.1 | *Apilactobacillus kunkeei* | H3B1-02A | Animal |
| GCF_946887595.1 | *Apilactobacillus kunkeei* | G0803 | Animal |
| GCF_946887605.1 | *Apilactobacillus kunkeei* | G0402 | Animal |
| GCF_946887615.1 | *Apilactobacillus kunkeei* | H1B1-04J | Animal |
| GCF_946887625.1 | *Apilactobacillus kunkeei* | A2002 | Animal |
| GCF_946887635.1 | *Apilactobacillus kunkeei* | G0403 | Animal |
| GCF_946887645.1 | *Apilactobacillus kunkeei* | A2003 | Animal |
| GCF_946887655.1 | *Apilactobacillus kunkeei* | A1805 | Animal |
| GCF_946887665.1 | *Apilactobacillus kunkeei* | H3B1-03M | Animal |
| GCF_946887685.1 | *Apilactobacillus kunkeei* | G0404 | Animal |
| GCF_946887695.1 | *Apilactobacillus kunkeei* | H3B2-09X | Animal |
| GCF_946887705.1 | *Apilactobacillus kunkeei* | H4B4-02J | Animal |
| GCF_946887715.1 | *Apilactobacillus kunkeei* | G0702 | Animal |
| GCF_946887725.1 | *Apilactobacillus kunkeei* | H3B1-10M | Animal |
| GCF_946887735.1 | *Apilactobacillus kunkeei* | H3B2-07X | Animal |
| GCF_946887745.1 | *Apilactobacillus kunkeei* | H3B1-11A | Animal |
| GCF_946887765.1 | *Apilactobacillus kunkeei* | H3B1-01A | Animal |
| GCF_946887775.1 | *Apilactobacillus kunkeei* | H3B1-09M | Animal |
| GCF_946887795.1 | *Apilactobacillus kunkeei* | G0801 | Animal |
| GCF_946887825.1 | *Apilactobacillus kunkeei* | H4B2-11M | Animal |
| GCF_946887835.1 | *Apilactobacillus kunkeei* | H1B3-02M | Animal |
| GCF_946887845.1 | *Apilactobacillus kunkeei* | H3B2-04J | Animal |
| GCF_946887855.1 | *Apilactobacillus kunkeei* | H3B2-05J | Animal |
| GCF_946887865.1 | *Apilactobacillus kunkeei* | H3B2-08X | Animal |
| GCF_946887895.1 | *Apilactobacillus kunkeei* | A1401 | Animal |
| GCF_946887935.1 | *Apilactobacillus kunkeei* | H4B2-02J | Animal |
| GCF_946887955.1 | *Apilactobacillus kunkeei* | G0601 | Animal |
| GCF_946888005.1 | *Apilactobacillus kunkeei* | H3B2-03J | Animal |
| GCF_946888015.1 | *Apilactobacillus kunkeei* | H3B1-04X | Animal |
| GCF_946888065.1 | *Apilactobacillus kunkeei* | G0401 | Animal |
| GCF_946888095.1 | *Apilactobacillus kunkeei* | G0414 | Animal |
| GCF_946888115.1 | *Apilactobacillus kunkeei* | H4B2-05J | Animal |
| GCF_946888145.1 | *Apilactobacillus kunkeei* | G0417 | Animal |
| GCF_946888155.1 | *Apilactobacillus kunkeei* | G0415 | Animal |
| GCF_946888165.1 | *Apilactobacillus kunkeei* | A1001 | Animal |
| GCF_946888185.1 | *Apilactobacillus kunkeei* | H3B1-04J | Animal |
| GCF_946888215.1 | *Apilactobacillus kunkeei* | G0407 | Animal |
| GCF_946888225.1 | *Apilactobacillus kunkeei* | Fhon2 | Animal |
| GCF_946888245.1 | *Apilactobacillus kunkeei* | A2103 | Animal |
| GCF_946888255.1 | *Apilactobacillus kunkeei* | H4B5-02X | Animal |
| GCF_946888265.1 | *Apilactobacillus kunkeei* | H4B5-08X | Animal |
| GCF_946888275.1 | *Apilactobacillus kunkeei* | A1202 | Animal |
| GCF_946888285.1 | *Apilactobacillus kunkeei* | H4B5-01J | Animal |
| GCF_946888295.1 | *Apilactobacillus kunkeei* | H4B5-05J | Animal |
| GCF_946888305.1 | *Apilactobacillus kunkeei* | H3B2-02X | Animal |
| GCF_946888325.1 | *Apilactobacillus kunkeei* | A1003 | Animal |
| GCF_946888345.1 | *Apilactobacillus kunkeei* | G0602 | Animal |
| GCF_946888355.1 | *Apilactobacillus kunkeei* | H3B1-07A | Animal |
| GCF_946888375.1 | *Apilactobacillus kunkeei* | H1B1-05A | Animal |
| GCF_946888405.1 | *Apilactobacillus kunkeei* | G0408 | Animal |
| GCF_946888415.1 | *Apilactobacillus kunkeei* | H4B4-12M | Animal |
| GCF_946888425.1 | *Apilactobacillus kunkeei* | H4B5-07J | Animal |
| GCF_946888455.1 | *Apilactobacillus kunkeei* | H4B5-04J | Animal |
| GCF_946888505.1 | *Apilactobacillus kunkeei* | H3B1-03J | Animal |
| GCF_946888535.1 | *Apilactobacillus kunkeei* | H3B1-11M | Animal |
| GCF_946888545.1 | *Apilactobacillus kunkeei* | G0102 | Animal |
| GCF_946888655.1 | *Apilactobacillus kunkeei* | H4B5-03X | Animal |
| GCF_007991815.1 | *Companilactobacillus paralimentarius* | NBRC 106466 | Others |
| GCF_007991895.1 | *Companilactobacillus paralimentarius* | NBRC 107149 | Others |
| GCF_031323805.1 | *Companilactobacillus paralimentarius* | FUA3121 | Food |
| GCF_963227115.1 | *Companilactobacillus paralimentarius* | R19081 | Food |
| GCF_963227125.1 | *Companilactobacillus paralimentarius* | R19092 | Food |
| GCF_963227135.1 | *Companilactobacillus paralimentarius* | R19079 | Food |
| GCF_963227255.1 | *Companilactobacillus paralimentarius* | IMDO 1MG105 | Food |
| GCF_963227545.1 | *Companilactobacillus paralimentarius* | IMDO 1MG81 | Food |
| GCF_963227675.1 | *Companilactobacillus paralimentarius* | IMDO 1MG86 | Food |
| GCF_963227695.1 | *Companilactobacillus paralimentarius* | IMDO EBRM1 | Food |
| GCF_963506725.1 | *Companilactobacillus paralimentarius* | IMDO BBRM18 | Food |
| GCF_963506805.1 | *Companilactobacillus paralimentarius* | R18618 | Food |
| GCF_000185465.1 | *Fructilactobacillus fructivorans* | KCTC 3543 | Animal |
| GCF_000814735.1 | *Fructilactobacillus fructivorans* | DmCS_002 | Others |
| GCF_001434095.1 | *Fructilactobacillus fructivorans* | DSM 20203 | Others |
| GCF_001436935.1 | *Fructilactobacillus fructivorans* | DSM 20350 | Food |
| GCF_001436985.1 | *Fructilactobacillus fructivorans* | DSM 20571 | Others |
| GCF_001437175.1 | *Fructilactobacillus fructivorans* | ATCC 27394 | Food |
| GCF_003368395.1 | *Fructilactobacillus fructivorans* | KCTC 3543 | Others |
| GCF_009496955.1 | *Fructilactobacillus fructivorans* | LF543 | Others |
| GCF_025129355.1 | *Fructilactobacillus fructivorans* | 196 | Environment |
| GCF_025185805.1 | *Fructilactobacillus fructivorans* | 176 | Environment |
| GCF_025185845.1 | *Fructilactobacillus fructivorans* | 175 | Environment |
| GCF_025185855.1 | *Fructilactobacillus fructivorans* | 187 | Environment |
| GCF_965135945.1 | *Fructilactobacillus fructivorans* | CIP103141 | Others |
| GCF_002907145.1 | *Fructilactobacillus sanfranciscensis* | TMW 1.2137 | Food |
| GCF_002907155.1 | *Fructilactobacillus sanfranciscensis* | TMW 1.2138 | Food |
| GCF_002907185.1 | *Fructilactobacillus sanfranciscensis* | TMW 1.2139 | Food |
| GCF_002907205.1 | *Fructilactobacillus sanfranciscensis* | TMW 1.2140 | Food |
| GCF_002907225.1 | *Fructilactobacillus sanfranciscensis* | TMW 1.2142 | Food |
| GCF_002907245.1 | *Fructilactobacillus sanfranciscensis* | TMW 1.2141 | Food |
| GCF_002907255.1 | *Fructilactobacillus sanfranciscensis* | TMW 1.726 | Food |
| GCF_002907285.1 | *Fructilactobacillus sanfranciscensis* | TMW 1.1597 | Food |
| GCF_002907305.1 | *Fructilactobacillus sanfranciscensis* | TMW 1.54 | Food |
| GCF_002907325.1 | *Fructilactobacillus sanfranciscensis* | TMW 1.1150 | Food |
| GCF_002907345.1 | *Fructilactobacillus sanfranciscensis* | TMW 1.392 | Food |
| GCF_003369815.1 | *Fructilactobacillus sanfranciscensis* | JCM 5668 | Food |
| GCF_006334415.1 | *Fructilactobacillus sanfranciscensis* | Ah4 | Food |
| GCF_006334425.1 | *Fructilactobacillus sanfranciscensis* | Gs9 | Food |
| GCF_006334465.1 | *Fructilactobacillus sanfranciscensis* | Sd1-3 | Food |
| GCF_006334475.1 | *Fructilactobacillus sanfranciscensis* | Gs2 | Food |
| GCF_009496975.1 | *Fructilactobacillus sanfranciscensis* | LS451 | Food |
| GCF_009749295.1 | *Fructilactobacillus sanfranciscensis* | Ls-1001 | Food |
| GCF_010377125.1 | *Fructilactobacillus sanfranciscensis* | TMW11730 | Food |
| GCF_010377135.1 | *Fructilactobacillus sanfranciscensis* | TMW11470 | Food |
| GCF_010377175.1 | *Fructilactobacillus sanfranciscensis* | TMW11304 | Food |
| GCF_010377205.1 | *Fructilactobacillus sanfranciscensis* | TMW11154 | Food |
| GCF_010377225.1 | *Fructilactobacillus sanfranciscensis* | TMW11152 | Food |
| GCF_010377235.1 | *Fructilactobacillus sanfranciscensis* | TMW12134 | Food |
| GCF_010377255.1 | *Fructilactobacillus sanfranciscensis* | TMW1907 | Food |
| GCF_010377275.1 | *Fructilactobacillus sanfranciscensis* | TMW1640 | Food |
| GCF_022343425.1 | *Fructilactobacillus sanfranciscensis* | TMW1936 | Food |
| GCF_022343445.1 | *Fructilactobacillus sanfranciscensis* | TMW1897 | Food |
| GCF_029023685.1 | *Fructilactobacillus sanfranciscensis* | SPC-SNU-70-4 | Others |
| GCF_030415415.1 | *Fructilactobacillus sanfranciscensis* | TMW 1.2323 | Food |
| GCF_039535745.1 | *Fructilactobacillus sanfranciscensis* | JCM 5668 | Food |
| GCF_039645225.1 | *Fructilactobacillus sanfranciscensis* | Fs_1014 | Food |
| GCF_039645235.1 | *Fructilactobacillus sanfranciscensis* | Fs_1011 | Food |
| GCF_039645285.1 | *Fructilactobacillus sanfranciscensis* | Fs_1012 | Food |
| GCF_039645305.1 | *Fructilactobacillus sanfranciscensis* | Fs_1013 | Food |
| GCF_039645325.1 | *Fructilactobacillus sanfranciscensis* | Fs_1009 | Food |
| GCF_039645345.1 | *Fructilactobacillus sanfranciscensis* | Fs_1006 | Food |
| GCF_039645375.1 | *Fructilactobacillus sanfranciscensis* | Fs_1007 | Food |
| GCF_039645395.1 | *Fructilactobacillus sanfranciscensis* | Fs_1005 | Food |
| GCF_039645415.1 | *Fructilactobacillus sanfranciscensis* | Fs_1008 | Food |
| GCF_039645435.1 | *Fructilactobacillus sanfranciscensis* | Fs_1004 | Food |
| GCF_039645455.1 | *Fructilactobacillus sanfranciscensis* | Fs_1003 | Food |
| GCF_039645475.1 | *Fructilactobacillus sanfranciscensis* | Fs_1002 | Food |
| GCF_039645495.1 | *Fructilactobacillus sanfranciscensis* | Fs_1001 | Food |
| GCF_044049425.1 | *Fructilactobacillus sanfranciscensis* | FUA 30089 | Others |
| GCF_044050215.1 | *Fructilactobacillus sanfranciscensis* | FUA 30088 | Others |
| GCF_044050225.1 | *Fructilactobacillus sanfranciscensis* | FUA 30087 | Others |
| GCF_044050235.1 | *Fructilactobacillus sanfranciscensis* | FUA 3417 | Others |
| GCF_963227785.1 | *Fructilactobacillus sanfranciscensis* | IMDO 150101 | Food |
| GCF_963506705.1 | *Fructilactobacillus sanfranciscensis* | ACADC 3378 | Food |
| GCF_963506835.1 | *Fructilactobacillus sanfranciscensis* | R19202 | Food |
| GCF_001636215.1 | *Lacticaseibacillus paracasei* | BM-LC14617 | Food |
| GCF_001858275.1 | *Lacticaseibacillus paracasei* | L9D | Human |
| GCF_003712785.1 | *Lacticaseibacillus paracasei* | FAM6161 | Food |
| GCF_013867595.1 | *Lacticaseibacillus paracasei* | s-16 | Others |
| GCF_018257175.1 | *Lacticaseibacillus paracasei* | Dm-2019-60 | Animal |
| GCF_025190665.1 | *Lacticaseibacillus paracasei* | CIRM-BIA 655 | Environment |
| GCF_027666625.1 | *Lacticaseibacillus paracasei* | AM74-01pH10A | Human |
| GCF_027666665.1 | *Lacticaseibacillus paracasei* | AM75-01pH5A | Human |
| GCF_027680865.1 | *Lacticaseibacillus paracasei* | AF67-02pH5A | Human |
| GCF_027685825.1 | *Lacticaseibacillus paracasei* | AF11-6H | Human |
| GCF_027685985.1 | *Lacticaseibacillus paracasei* | AF11-16H | Human |
| GCF_027693445.1 | *Lacticaseibacillus paracasei* | OF26-11pH5A | Human |
| GCF_028878315.1 | *Lacticaseibacillus paracasei* | DSM 4905 | Human |
| GCF_030939115.1 | *Lacticaseibacillus paracasei* | AV 2-1 | Human |
| GCF_039615415.1 | *Lacticaseibacillus paracasei* | TBM 27 | Food |
| GCF_049191405.1 | *Lacticaseibacillus paracasei* | IATA083 | Human |
| GCF_051123965.1 | *Lacticaseibacillus paracasei* | M8 | Food |
| GCF_000743075.1 | *Lacticaseibacillus rhamnosus* | 24 | Human |
| GCF_000801045.1 | *Lacticaseibacillus rhamnosus* | 116 | Human |
| GCF_000814485.1 | *Lacticaseibacillus rhamnosus* | 308 | Human |
| GCF_000932035.1 | *Lacticaseibacillus rhamnosus* | CLS17 | Human |
| GCF_001005625.1 | *Lacticaseibacillus rhamnosus* | CNCM-I-3698 | Animal |
| GCF_001044405.1 | *Lacticaseibacillus rhamnosus* | 40f | Human |
| GCF_001044415.1 | *Lacticaseibacillus rhamnosus* | 313 | Human |
| GCF_001062885.1 | *Lacticaseibacillus rhamnosus* | 186_LRHA | Human |
| GCF_001062955.1 | *Lacticaseibacillus rhamnosus* | 214_LRHA | Human |
| GCF_001063655.1 | *Lacticaseibacillus rhamnosus* | 526_LRHA | Human |
| GCF_001064515.1 | *Lacticaseibacillus rhamnosus* | 319_LRHA | Human |
| GCF_001064785.1 | *Lacticaseibacillus rhamnosus* | 390_LRHA | Human |
| GCF_001065365.1 | *Lacticaseibacillus rhamnosus* | 541_LRHA | Human |
| GCF_001067215.1 | *Lacticaseibacillus rhamnosus* | 769_LRHA | Human |
| GCF_001067335.1 | *Lacticaseibacillus rhamnosus* | 784_LRHA | Human |
| GCF_001068015.1 | *Lacticaseibacillus rhamnosus* | 979_LRHA | Human |
| GCF_001068215.1 | *Lacticaseibacillus rhamnosus* | 944_LRHA | Human |
| GCF_001368735.1 | *Lacticaseibacillus rhamnosus* | BPL15 | Others |
| GCF_001590655.1 | *Lacticaseibacillus rhamnosus* | ASCC 290 | Food |
| GCF_001645615.1 | *Lacticaseibacillus rhamnosus* | R19-3 | Human |
| GCF_001656535.1 | *Lacticaseibacillus rhamnosus* | Lrh8 | Human |
| GCF_001656545.1 | *Lacticaseibacillus rhamnosus* | Lrh32 | Human |
| GCF_001656575.1 | *Lacticaseibacillus rhamnosus* | Lrh31 | Human |
| GCF_001656635.1 | *Lacticaseibacillus rhamnosus* | Lrh23 | Human |
| GCF_001656655.1 | *Lacticaseibacillus rhamnosus* | Lrh22 | Human |
| GCF_001656675.1 | *Lacticaseibacillus rhamnosus* | Lrh20 | Human |
| GCF_001656685.1 | *Lacticaseibacillus rhamnosus* | Lrh19 | Human |
| GCF_001656735.1 | *Lacticaseibacillus rhamnosus* | Lrh11 | Human |
| GCF_001656765.1 | *Lacticaseibacillus rhamnosus* | Lrh34 | Food |
| GCF_001656785.1 | *Lacticaseibacillus rhamnosus* | Lrh9 | Human |
| GCF_001656815.1 | *Lacticaseibacillus rhamnosus* | Lrh7 | Human |
| GCF_001656895.1 | *Lacticaseibacillus rhamnosus* | Lrh30 | Human |
| GCF_001656925.1 | *Lacticaseibacillus rhamnosus* | Lrh28 | Human |
| GCF_001656945.1 | *Lacticaseibacillus rhamnosus* | Lrh27 | Human |
| GCF_001656995.1 | *Lacticaseibacillus rhamnosus* | Lrh21 | Human |
| GCF_001657055.1 | *Lacticaseibacillus rhamnosus* | Lrh18 | Human |
| GCF_001657075.1 | *Lacticaseibacillus rhamnosus* | Lrh17 | Human |
| GCF_001657115.1 | *Lacticaseibacillus rhamnosus* | Lrh14 | Human |
| GCF_001657135.1 | *Lacticaseibacillus rhamnosus* | Lrh13 | Human |
| GCF_001657165.1 | *Lacticaseibacillus rhamnosus* | Lrh10 | Food |
| GCF_001657205.1 | *Lacticaseibacillus rhamnosus* | Lrh42 | Others |
| GCF_001756565.1 | *Lacticaseibacillus rhamnosus* | HCT70 | Food |
| GCF_001831215.1 | *Lacticaseibacillus rhamnosus* | ASCC 3018 | Others |
| GCF_001831225.1 | *Lacticaseibacillus rhamnosus* | ASCC 3016 | Others |
| GCF_001831235.1 | *Lacticaseibacillus rhamnosus* | ASCC 3029 | Others |
| GCF_001831275.1 | *Lacticaseibacillus rhamnosus* | ASCC 1521 | Others |
| GCF_001981725.1 | *Lacticaseibacillus rhamnosus* | RI-004 | Others |
| GCF_001988935.1 | *Lacticaseibacillus rhamnosus* | BFE5264 | Food |
| GCF_001991035.1 | *Lacticaseibacillus rhamnosus* | L156.4 | Animal |
| GCF_002025085.1 | *Lacticaseibacillus rhamnosus* | WQ2 | Food |
| GCF_002027355.1 | *Lacticaseibacillus rhamnosus* | R709 | Food |
| GCF_002076955.1 | *Lacticaseibacillus rhamnosus* | Pen | Human |
| GCF_002103155.1 | *Lacticaseibacillus rhamnosus* | Lrh38 | Human |
| GCF_002103185.1 | *Lacticaseibacillus rhamnosus* | Lrh47 | Human |
| GCF_002103215.1 | *Lacticaseibacillus rhamnosus* | Lrh46 | Human |
| GCF_002158925.1 | *Lacticaseibacillus rhamnosus* | 4B15 | Human |
| GCF_002238035.1 | *Lacticaseibacillus rhamnosus* | IBL027 | Human |
| GCF_002286235.1 | *Lacticaseibacillus rhamnosus* | LR5 | Human |
| GCF_002406705.1 | *Lacticaseibacillus rhamnosus* | B1 | Food |
| GCF_002406715.1 | *Lacticaseibacillus rhamnosus* | P5 | Food |
| GCF_002406745.1 | *Lacticaseibacillus rhamnosus* | P4 | Food |
| GCF_002406795.1 | *Lacticaseibacillus rhamnosus* | P1 | Food |
| GCF_002762445.1 | *Lacticaseibacillus rhamnosus* | GR-1 | Food |
| GCF_002848015.1 | *Lacticaseibacillus rhamnosus* | UMB0004 | Human |
| GCF_002849515.1 | *Lacticaseibacillus rhamnosus* | NRRL B-442 | Human |
| GCF_002960215.1 | *Lacticaseibacillus rhamnosus* | SCT-10-10-60 | Human |
| GCF_003046115.1 | *Lacticaseibacillus rhamnosus* | LR2 | Food |
| GCF_003052925.1 | *Lacticaseibacillus rhamnosus* | DS18_11 | Food |
| GCF_003052945.1 | *Lacticaseibacillus rhamnosus* | DS13_11 | Food |
| GCF_003052965.1 | *Lacticaseibacillus rhamnosus* | DS4_11 | Food |
| GCF_003052985.1 | *Lacticaseibacillus rhamnosus* | DS3_11 | Food |
| GCF_003061565.1 | *Lacticaseibacillus rhamnosus* | DS17_11 | Food |
| GCF_003061605.1 | *Lacticaseibacillus rhamnosus* | DS22_11 | Food |
| GCF_003061625.1 | *Lacticaseibacillus rhamnosus* | DS9_11 | Food |
| GCF_003061645.1 | *Lacticaseibacillus rhamnosus* | DS15_11 | Food |
| GCF_003061665.1 | *Lacticaseibacillus rhamnosus* | DS12_11 | Food |
| GCF_003061705.1 | *Lacticaseibacillus rhamnosus* | DS14_11 | Food |
| GCF_003129615.1 | *Lacticaseibacillus rhamnosus* | TMC3115 | Food |
| GCF_003129645.1 | *Lacticaseibacillus rhamnosus* | LR863 | Food |
| GCF_003433395.1 | *Lacticaseibacillus rhamnosus* | JCM1553 | Others |
| GCF_003573615.1 | *Lacticaseibacillus rhamnosus* | ARJD | Food |
| GCF_004010975.1 | *Lacticaseibacillus rhamnosus* | LR-B1 | Human |
| GCF_004122925.1 | *Lacticaseibacillus rhamnosus* | 5-?.?. | Food |
| GCF_004125395.1 | *Lacticaseibacillus rhamnosus* | LR-B2 | Human |
| GCF_004125455.1 | *Lacticaseibacillus rhamnosus* | LR-CVC | Human |
| GCF_004125465.1 | *Lacticaseibacillus rhamnosus* | LR-GG-MoProbi | Human |
| GCF_004125475.1 | *Lacticaseibacillus rhamnosus* | LR-S | Human |
| GCF_004167055.1 | *Lacticaseibacillus rhamnosus* | co_0103 | Human |
| GCF_004798455.1 | *Lacticaseibacillus rhamnosus* | UBLR-58 | Food |
| GCF_005864245.1 | *Lacticaseibacillus rhamnosus* | FAM 20558 | Food |
| GCF_006151905.1 | *Lacticaseibacillus rhamnosus* | 1.032 | Food |
| GCF_008727835.1 | *Lacticaseibacillus rhamnosus* | hsryfm 1301 | Human |
| GCF_008831425.1 | *Lacticaseibacillus rhamnosus* | BIO6870 | Human |
| GCF_009429065.1 | *Lacticaseibacillus rhamnosus* | IDCC 3201 | Human |
| GCF_009679255.1 | *Lacticaseibacillus rhamnosus* | BIOML-A10 | Human |
| GCF_009679265.1 | *Lacticaseibacillus rhamnosus* | BIOML-A9 | Human |
| GCF_009679295.1 | *Lacticaseibacillus rhamnosus* | BIOML-A8 | Human |
| GCF_009679335.1 | *Lacticaseibacillus rhamnosus* | BIOML-A4 | Human |
| GCF_009679345.1 | *Lacticaseibacillus rhamnosus* | BIOML-A5 | Human |
| GCF_009679355.1 | *Lacticaseibacillus rhamnosus* | BIOML-A6 | Human |
| GCF_009679395.1 | *Lacticaseibacillus rhamnosus* | BIOML-A3 | Human |
| GCF_009679405.1 | *Lacticaseibacillus rhamnosus* | BIOML-A2 | Human |
| GCF_009720565.1 | *Lacticaseibacillus rhamnosus* | BIO5326 | Human |
| GCF_009742715.1 | *Lacticaseibacillus rhamnosus* | LR_AVK | Food |
| GCF_009805825.1 | *Lacticaseibacillus rhamnosus* | Lrh40 | Human |
| GCF_013167115.1 | *Lacticaseibacillus rhamnosus* | LV108 | Human |
| GCF_013377685.1 | *Lacticaseibacillus rhamnosus* | QC | Animal |
| GCF_013425565.1 | *Lacticaseibacillus rhamnosus* | AMC0707 | Human |
| GCF_013425605.1 | *Lacticaseibacillus rhamnosus* | AMC0720 | Human |
| GCF_013425645.1 | *Lacticaseibacillus rhamnosus* | AMC0716 | Human |
| GCF_013425665.1 | *Lacticaseibacillus rhamnosus* | AMC0722 | Human |
| GCF_013425725.1 | *Lacticaseibacillus rhamnosus* | AMC0712 | Human |
| GCF_013425735.1 | *Lacticaseibacillus rhamnosus* | AMC0721 | Human |
| GCF_014155845.1 | *Lacticaseibacillus rhamnosus* | DPC 7102 | Food |
| GCF_014212185.1 | *Lacticaseibacillus rhamnosus* | CBC-LR1 | Food |
| GCF_014639015.1 | *Lacticaseibacillus rhamnosus* | NCB 441 | Food |
| GCF_015160815.1 | *Lacticaseibacillus rhamnosus* | RSI3 | Food |
| GCF_015377485.1 | *Lacticaseibacillus rhamnosus* | TK-F8B | Food |
| GCF_015548835.1 | *Lacticaseibacillus rhamnosus* | 1001283B150210_160208_A4 | Human |
| GCF_015549085.1 | *Lacticaseibacillus rhamnosus* | 1001311H_170123_H11 | Human |
| GCF_015557825.1 | *Lacticaseibacillus rhamnosus* | 1001216B_150713_B1 | Human |
| GCF_015558325.1 | *Lacticaseibacillus rhamnosus* | 1001287B_170213_A1 | Human |
| GCF_015668455.1 | *Lacticaseibacillus rhamnosus* | 1001270B_150601_F12 | Human |
| GCF_016599675.1 | *Lacticaseibacillus rhamnosus* | B6 | Food |
| GCF_016653515.1 | *Lacticaseibacillus rhamnosus* | KF7 | Food |
| GCF_017795605.1 | *Lacticaseibacillus rhamnosus* | LDTM7511 | Human |
| GCF_018141205.1 | *Lacticaseibacillus rhamnosus* | CE1 | Food |
| GCF_018228745.1 | *Lacticaseibacillus rhamnosus* | X253 | Food |
| GCF_018286375.1 | *Lacticaseibacillus rhamnosus* | AS | Food |
| GCF_018449495.1 | *Lacticaseibacillus rhamnosus* | Lr-G14 | Food |
| GCF_018458775.1 | *Lacticaseibacillus rhamnosus* | RAB2019A | Human |
| GCF_018966895.1 | *Lacticaseibacillus rhamnosus* | F | Human |
| GCF_019967935.1 | *Lacticaseibacillus rhamnosus* | CAU 1365 | Food |
| GCF_019990845.1 | *Lacticaseibacillus rhamnosus* | cek-R1 | Environment |
| GCF_020826335.1 | *Lacticaseibacillus rhamnosus* | PMC203 | Human |
| GCF_020844065.2 | *Lacticaseibacillus rhamnosus* | IMI 507023 | Environment |
| GCF_022220485.1 | *Lacticaseibacillus rhamnosus* | VHProbi Y39 | Human |
| GCF_022509255.1 | *Lacticaseibacillus rhamnosus* | QAULRN2 | Food |
| GCF_022802715.1 | *Lacticaseibacillus rhamnosus* | 3160-SD0198 | Human |
| GCF_022802735.1 | *Lacticaseibacillus rhamnosus* | 3189-SD0197 | Human |
| GCF_022802755.1 | *Lacticaseibacillus rhamnosus* | 3195-SD0196 | Human |
| GCF_023913535.1 | *Lacticaseibacillus rhamnosus* | P118 | Food |
| GCF_024053515.2 | *Lacticaseibacillus rhamnosus* | DM065 | Human |
| GCF_024158105.2 | *Lacticaseibacillus rhamnosus* | DM054 | Human |
| GCF_024397415.1 | *Lacticaseibacillus rhamnosus* | HN067 | Human |
| GCF_024442295.1 | *Lacticaseibacillus rhamnosus* | CLK 101 | Food |
| GCF_024610975.1 | *Lacticaseibacillus rhamnosus* | VHProbi M14 | Human |
| GCF_024665595.1 | *Lacticaseibacillus rhamnosus* | GR-1 | Human |
| GCF_024718295.1 | *Lacticaseibacillus rhamnosus* | O8-1 | Human |
| GCF_025189265.1 | *Lacticaseibacillus rhamnosus* | CIRM-BIA 1107 | Environment |
| GCF_025189285.1 | *Lacticaseibacillus rhamnosus* | CIRM-BIA 909 | Others |
| GCF_025189315.1 | *Lacticaseibacillus rhamnosus* | CIRM-BIA 607 | Others |
| GCF_025189345.1 | *Lacticaseibacillus rhamnosus* | CIRM-BIA 2120 | Others |
| GCF_025189365.1 | *Lacticaseibacillus rhamnosus* | CIRM-BIA 930 | Food |
| GCF_025189385.1 | *Lacticaseibacillus rhamnosus* | CIRM-BIA 1112 | Food |
| GCF_025189405.1 | *Lacticaseibacillus rhamnosus* | CIRM-BIA 774 | Food |
| GCF_025189415.1 | *Lacticaseibacillus rhamnosus* | CIRM-BIA 780 | Others |
| GCF_025189425.1 | *Lacticaseibacillus rhamnosus* | CIRM-BIA 2116 | Others |
| GCF_025189465.1 | *Lacticaseibacillus rhamnosus* | CIRM-BIA 913 | Others |
| GCF_025189475.1 | *Lacticaseibacillus rhamnosus* | CIRM-BIA 2131 | Food |
| GCF_025189505.1 | *Lacticaseibacillus rhamnosus* | CIRM-BIA 2132 | Food |
| GCF_025189525.1 | *Lacticaseibacillus rhamnosus* | CIRM-BIA 2133 | Food |
| GCF_025189545.1 | *Lacticaseibacillus rhamnosus* | CIRM-BIA 910 | Food |
| GCF_025189565.1 | *Lacticaseibacillus rhamnosus* | CIRM-BIA 2134 | Food |
| GCF_025189585.1 | *Lacticaseibacillus rhamnosus* | CIRM-BIA 1436 | Food |
| GCF_025189605.1 | *Lacticaseibacillus rhamnosus* | CIRM-BIA 1952 | Others |
| GCF_025195045.1 | *Lacticaseibacillus rhamnosus* | L12 | Human |
| GCF_025212175.1 | *Lacticaseibacillus rhamnosus* | CIRM-BIA 777 | Food |
| GCF_025631035.1 | *Lacticaseibacillus rhamnosus* | PB7 | Food |
| GCF_026427555.1 | *Lacticaseibacillus rhamnosus* | VHProbi F20 | Human |
| GCF_026966975.1 | *Lacticaseibacillus rhamnosus* | Lrh_yogurt_D6 | Food |
| GCF_026966995.1 | *Lacticaseibacillus rhamnosus* | Lrh_yogurt_D4 | Food |
| GCF_026967015.1 | *Lacticaseibacillus rhamnosus* | Lrh_yogurt_D5 | Food |
| GCF_026967025.1 | *Lacticaseibacillus rhamnosus* | Lrh_yogurt_D3 | Food |
| GCF_026967035.1 | *Lacticaseibacillus rhamnosus* | Lrh_yogurt_D2 | Food |
| GCF_026967075.1 | *Lacticaseibacillus rhamnosus* | Lrh_yogurt_D1 | Food |
| GCF_026967105.1 | *Lacticaseibacillus rhamnosus* | Lrh_yogurt_C2 | Food |
| GCF_026967125.1 | *Lacticaseibacillus rhamnosus* | Lrh_yogurt_C4 | Food |
| GCF_026967135.1 | *Lacticaseibacillus rhamnosus* | Lrh_yogurt_C6 | Food |
| GCF_026967315.1 | *Lacticaseibacillus rhamnosus* | Lrh_yogurt_A5 | Food |
| GCF_026967335.1 | *Lacticaseibacillus rhamnosus* | Lrh_yogurt_A6 | Food |
| GCF_026967345.1 | *Lacticaseibacillus rhamnosus* | Lrh_yogurt_A2 | Food |
| GCF_026967355.1 | *Lacticaseibacillus rhamnosus* | Lrh_yogurt_A4 | Food |
| GCF_026967395.1 | *Lacticaseibacillus rhamnosus* | Lrh_yogurt_A1 | Food |
| GCF_026967405.1 | *Lacticaseibacillus rhamnosus* | Lrh_blood001 | Human |
| GCF_027692365.1 | *Lacticaseibacillus rhamnosus* | OF44-15pH10T | Human |
| GCF_027692625.1 | *Lacticaseibacillus rhamnosus* | OF38-7pH5A | Human |
| GCF_027857655.1 | *Lacticaseibacillus rhamnosus* | lbd85-1 | Food |
| GCF_027857665.1 | *Lacticaseibacillus rhamnosus* | lbd330 | Food |
| GCF_027857695.1 | *Lacticaseibacillus rhamnosus* | lbr152 | Food |
| GCF_027857715.1 | *Lacticaseibacillus rhamnosus* | lbr105 | Food |
| GCF_027857735.1 | *Lacticaseibacillus rhamnosus* | lbr108 | Food |
| GCF_027857765.1 | *Lacticaseibacillus rhamnosus* | lbr154 | Food |
| GCF_027857795.1 | *Lacticaseibacillus rhamnosus* | lbr136 | Food |
| GCF_028320325.1 | *Lacticaseibacillus rhamnosus* | F12 | Human |
| GCF_028321125.1 | *Lacticaseibacillus rhamnosus* | F11 | Human |
| GCF_028321165.1 | *Lacticaseibacillus rhamnosus* | D6 | Human |
| GCF_028322035.1 | *Lacticaseibacillus rhamnosus* | D1 | Human |
| GCF_028322725.1 | *Lacticaseibacillus rhamnosus* | F3 | Human |
| GCF_028322785.1 | *Lacticaseibacillus rhamnosus* | F6 | Human |
| GCF_028322835.1 | *Lacticaseibacillus rhamnosus* | F11 | Human |
| GCF_028322895.1 | *Lacticaseibacillus rhamnosus* | D3 | Human |
| GCF_028323345.1 | *Lacticaseibacillus rhamnosus* | G3 | Human |
| GCF_028878115.1 | *Lacticaseibacillus rhamnosus* | N202 | Human |
| GCF_028878125.1 | *Lacticaseibacillus rhamnosus* | Mo2 | Human |
| GCF_028878145.1 | *Lacticaseibacillus rhamnosus* | N1110 | Human |
| GCF_028878245.1 | *Lacticaseibacillus rhamnosus* | O14 | Food |
| GCF_028878345.1 | *Lacticaseibacillus rhamnosus* | UD193 | Food |
| GCF_029010255.1 | *Lacticaseibacillus rhamnosus* | VSI33 | Human |
| GCF_029011275.1 | *Lacticaseibacillus rhamnosus* | VSI43 | Human |
| GCF_029269765.1 | *Lacticaseibacillus rhamnosus* | ERKN4 | Food |
| GCF_029543065.1 | *Lacticaseibacillus rhamnosus* | A5 | Others |
| GCF_029874435.1 | *Lacticaseibacillus rhamnosus* | FME96 | Food |
| GCF_029961125.1 | *Lacticaseibacillus rhamnosus* | LB C25 | Food |
| GCF_030034775.1 | *Lacticaseibacillus rhamnosus* | k32 | Human |
| GCF_030217405.1 | *Lacticaseibacillus rhamnosus* | UMB1344 | Human |
| GCF_030217425.1 | *Lacticaseibacillus rhamnosus* | UMB1351A | Human |
| GCF_030217985.1 | *Lacticaseibacillus rhamnosus* | UMB1066 | Human |
| GCF_030224725.1 | *Lacticaseibacillus rhamnosus* | UMB1351B | Human |
| GCF_030227945.1 | *Lacticaseibacillus rhamnosus* | UMB8035B | Human |
| GCF_030233995.1 | *Lacticaseibacillus rhamnosus* | UMB8035A | Human |
| GCF_030237645.1 | *Lacticaseibacillus rhamnosus* | C44 | Food |
| GCF_030237905.1 | *Lacticaseibacillus rhamnosus* | C33 | Food |
| GCF_030286545.1 | *Lacticaseibacillus rhamnosus* | ISO20 | Food |
| GCF_030361185.1 | *Lacticaseibacillus rhamnosus* | TOM.283 | Food |
| GCF_030480385.1 | *Lacticaseibacillus rhamnosus* | MCC 0256 | Others |
| GCF_030490305.1 | *Lacticaseibacillus rhamnosus* | DM163 | Human |
| GCF_030876785.1 | *Lacticaseibacillus rhamnosus* | L4 | Human |
| GCF_030929905.1 | *Lacticaseibacillus rhamnosus* | CRL 2244 | Environment |
| GCF_030939245.1 | *Lacticaseibacillus rhamnosus* | NA 1-8 | Human |
| GCF_031202655.1 | *Lacticaseibacillus rhamnosus* | Fmb14 | Food |
| GCF_031593775.1 | *Lacticaseibacillus rhamnosus* | VHProbi M12 | Food |
| GCF_031594435.1 | *Lacticaseibacillus rhamnosus* | VHProbi V6156 | Food |
| GCF_031626835.1 | *Lacticaseibacillus rhamnosus* | FUA3185 | Food |
| GCF_032248855.1 | *Lacticaseibacillus rhamnosus* | DS0508 | Human |
| GCF_032465875.1 | *Lacticaseibacillus rhamnosus* | LMG 23551 | Human |
| GCF_032465895.1 | *Lacticaseibacillus rhamnosus* | LMG 23327 | Human |
| GCF_032465915.1 | *Lacticaseibacillus rhamnosus* | LMG 23277 | Human |
| GCF_032465935.1 | *Lacticaseibacillus rhamnosus* | LMG 19717 | Human |
| GCF_032465955.1 | *Lacticaseibacillus rhamnosus* | LMG 19716 | Human |
| GCF_032465975.1 | *Lacticaseibacillus rhamnosus* | LMG 23550 | Human |
| GCF_032465995.1 | *Lacticaseibacillus rhamnosus* | LMG 10768 | Human |
| GCF_032841215.1 | *Lacticaseibacillus rhamnosus* | MRS-10 | Food |
| GCF_033802705.1 | *Lacticaseibacillus rhamnosus* | SN21-1 | Animal |
| GCF_033977045.1 | *Lacticaseibacillus rhamnosus* | LR6 | Human |
| GCF_035334605.1 | *Lacticaseibacillus rhamnosus* | S262-4W | Human |
| GCF_035594055.1 | *Lacticaseibacillus rhamnosus* | SBS-CC-2110 | Food |
| GCF_035928125.1 | *Lacticaseibacillus rhamnosus* | VHProbi V77 | Food |
| GCF_036454445.1 | *Lacticaseibacillus rhamnosus* | UO.H3002 | Human |
| GCF_036583945.1 | *Lacticaseibacillus rhamnosus* | CP-1 | Human |
| GCF_036595825.1 | *Lacticaseibacillus rhamnosus* | SD11 | Human |
| GCF_036595865.1 | *Lacticaseibacillus rhamnosus* | SD4 | Human |
| GCF_037118725.1 | *Lacticaseibacillus rhamnosus* | VTCC 12819 | Animal |
| GCF_037966675.1 | *Lacticaseibacillus rhamnosus* | ATA-LTC-Lrs010902 | Human |
| GCF_038039235.1 | *Lacticaseibacillus rhamnosus* | ICIS25 | Food |
| GCF_038086925.1 | *Lacticaseibacillus rhamnosus* | LR110 | Human |
| GCF_039615375.1 | *Lacticaseibacillus rhamnosus* | NTM 1004 | Food |
| GCF_039615465.1 | *Lacticaseibacillus rhamnosus* | SM 4.3 | Animal |
| GCF_039654095.1 | *Lacticaseibacillus rhamnosus* | S22825 | Food |
| GCF_039733345.1 | *Lacticaseibacillus rhamnosus* | C12 | Human |
| GCF_039751055.1 | *Lacticaseibacillus rhamnosus* | B7 | Human |
| GCF_039838405.1 | *Lacticaseibacillus rhamnosus* | B5 | Human |
| GCF_039838445.1 | *Lacticaseibacillus rhamnosus* | G9 | Human |
| GCF_039857935.1 | *Lacticaseibacillus rhamnosus* | TCI366 | Human |
| GCF_040235135.2 | *Lacticaseibacillus rhamnosus* | BIM B-189 | Others |
| GCF_040446565.1 | *Lacticaseibacillus rhamnosus* | MY-1 | Environment |
| GCF_040705095.1 | *Lacticaseibacillus rhamnosus* | PM4 | Human |
| GCF_040785145.1 | *Lacticaseibacillus rhamnosus* | gbc_e | Others |
| GCF_040785165.1 | *Lacticaseibacillus rhamnosus* | gbc_B | Others |
| GCF_040801795.1 | *Lacticaseibacillus rhamnosus* | MG4644 | Human |
| GCF_040822755.1 | *Lacticaseibacillus rhamnosus* | ATA-LRS1902 | Human |
| GCF_040883415.1 | *Lacticaseibacillus rhamnosus* | AM-LR-51 | Others |
| GCF_040914465.1 | *Lacticaseibacillus rhamnosus* | A7 | Human |
| GCF_040924875.1 | *Lacticaseibacillus rhamnosus* | H10 | Human |
| GCF_040924925.1 | *Lacticaseibacillus rhamnosus* | G4 | Human |
| GCF_040924945.1 | *Lacticaseibacillus rhamnosus* | A4 | Human |
| GCF_040924965.1 | *Lacticaseibacillus rhamnosus* | F3 | Human |
| GCF_040924975.1 | *Lacticaseibacillus rhamnosus* | F11 | Human |
| GCF_040924985.1 | *Lacticaseibacillus rhamnosus* | G9 | Human |
| GCF_040924995.1 | *Lacticaseibacillus rhamnosus* | G2 | Human |
| GCF_040925045.1 | *Lacticaseibacillus rhamnosus* | C2 | Human |
| GCF_040925065.1 | *Lacticaseibacillus rhamnosus* | A2 | Human |
| GCF_041167785.1 | *Lacticaseibacillus rhamnosus* | YGRT01 | Food |
| GCF_041228125.1 | *Lacticaseibacillus rhamnosus* | NS2301G1 | Food |
| GCF_041379775.1 | *Lacticaseibacillus rhamnosus* | YGRT11 | Food |
| GCF_041679765.1 | *Lacticaseibacillus rhamnosus* | GG_ID | Food |
| GCF_043589385.1 | *Lacticaseibacillus rhamnosus* | WHH1155 | Food |
| GCF_044283575.1 | *Lacticaseibacillus rhamnosus* | OF44 | Human |
| GCF_044339895.1 | *Lacticaseibacillus rhamnosus* | M2b | Human |
| GCF_045061055.1 | *Lacticaseibacillus rhamnosus* | MRD080 | Human |
| GCF_045256955.1 | *Lacticaseibacillus rhamnosus* | 484 | Human |
| GCF_046244375.1 | *Lacticaseibacillus rhamnosus* | SA74 | Human |
| GCF_047501695.1 | *Lacticaseibacillus rhamnosus* | MW019593 | Animal |
| GCF_047778005.1 | *Lacticaseibacillus rhamnosus* | MRD-YKTA | Human |
| GCF_048015625.1 | *Lacticaseibacillus rhamnosus* | B3421 | Environment |
| GCF_048629105.1 | *Lacticaseibacillus rhamnosus* | BBAH2 | Animal |
| GCF_048629435.1 | *Lacticaseibacillus rhamnosus* | TB3 | Animal |
| GCF_048629445.1 | *Lacticaseibacillus rhamnosus* | TS4 | Animal |
| GCF_048629795.1 | *Lacticaseibacillus rhamnosus* | HI1 | Animal |
| GCF_048629835.1 | *Lacticaseibacillus rhamnosus* | BBAH7 | Animal |
| GCF_049204955.1 | *Lacticaseibacillus rhamnosus* | IATA117 | Human |
| GCF_049205035.1 | *Lacticaseibacillus rhamnosus* | IATA115 | Human |
| GCF_050286485.1 | *Lacticaseibacillus rhamnosus* | JL | Food |
| GCF_050367955.1 | *Lacticaseibacillus rhamnosus* | SL42 | Human |
| GCF_050706795.1 | *Lacticaseibacillus rhamnosus* | VHProbi O07 | Human |
| GCF_051064015.1 | *Lacticaseibacillus rhamnosus* | 373F | Human |
| GCF_051064055.1 | *Lacticaseibacillus rhamnosus* | 370F | Human |
| GCF_051064375.1 | *Lacticaseibacillus rhamnosus* | 339F | Human |
| GCF_051123815.1 | *Lacticaseibacillus rhamnosus* | C1 | Food |
| GCF_051131585.1 | *Lacticaseibacillus rhamnosus* | UO.H3007 | Human |
| GCF_051132775.1 | *Lacticaseibacillus rhamnosus* | ICIS-1127 | Human |
| GCF_051200805.1 | *Lacticaseibacillus rhamnosus* | OBM8 | Food |
| GCF_051252675.1 | *Lacticaseibacillus rhamnosus* | LR02 | Food |
| GCF_051252695.1 | *Lacticaseibacillus rhamnosus* | LR01 | Food |
| GCF_051549825.1 | *Lacticaseibacillus rhamnosus* | MG20180718 | Food |
| GCF_051908385.1 | *Lacticaseibacillus rhamnosus* | KACC 92513P | Human |
| GCF_052567965.1 | *Lacticaseibacillus rhamnosus* | HL-JO04 | Human |
| GCF_900070175.1 | *Lacticaseibacillus rhamnosus* | BPL5 | Human |
| GCF_900636875.1 | *Lacticaseibacillus rhamnosus* | NCTC13710 | Others |
| GCF_900636965.1 | *Lacticaseibacillus rhamnosus* | NCTC13764 | Others |
| GCF_901830405.1 | *Lacticaseibacillus rhamnosus* | AMBR1 | Human |
| GCF_901971785.1 | *Lacticaseibacillus rhamnosus* | INIA P344 | Human |
| GCF_901971795.1 | *Lacticaseibacillus rhamnosus* | INIA P540 | Human |
| GCF_902166035.1 | *Lacticaseibacillus rhamnosus* | 4928STDY7387919 | Human |
| GCF_902381635.1 | *Lacticaseibacillus rhamnosus* | MGYG-HGUT-01293 | Human |
| GCF_902652215.1 | *Lacticaseibacillus rhamnosus* | LFYP97 | Others |
| GCF_925281845.1 | *Lacticaseibacillus rhamnosus* | IM1429 | Others |
| GCF_925281885.1 | *Lacticaseibacillus rhamnosus* | IM1389 | Others |
| GCF_925285305.1 | *Lacticaseibacillus rhamnosus* | IM561 | Others |
| GCF_925297845.1 | *Lacticaseibacillus rhamnosus* | IM1459 | Others |
| GCF_925301965.1 | *Lacticaseibacillus rhamnosus* | IM1338 | Others |
| GCF_003641165.1 | *Lactiplantibacillus argentoratensis* | DSM 16365 | Food |
| GCF_017963485.1 | *Lactiplantibacillus argentoratensis* | XSBN-2 | Food |
| GCF_018551525.1 | *Lactiplantibacillus argentoratensis* | LQC 2422 | Food |
| GCF_018551565.1 | *Lactiplantibacillus argentoratensis* | LQC 2520 | Food |
| GCF_018551595.1 | *Lactiplantibacillus argentoratensis* | LQC 2441 | Food |
| GCF_018552295.1 | *Lactiplantibacillus argentoratensis* | LQC 2320 | Food |
| GCF_018552305.1 | *Lactiplantibacillus argentoratensis* | LQC 2516 | Food |
| GCF_018917085.1 | *Lactiplantibacillus argentoratensis* | N104 | Animal |
| GCF_020295655.1 | *Lactiplantibacillus argentoratensis* | BSR19 | Animal |
| GCF_020552455.1 | *Lactiplantibacillus argentoratensis* | C7-83 | Food |
| GCF_025212135.1 | *Lactiplantibacillus argentoratensis* | CIRM-BIA 2108 | Food |
| GCF_030247085.1 | *Lactiplantibacillus argentoratensis* | ATP111 | Animal |
| GCF_037290055.1 | *Lactiplantibacillus argentoratensis* | D1 | Environment |
| GCF_046736475.1 | *Lactiplantibacillus argentoratensis* | CVASU4 | Animal |
| GCF_048589005.1 | *Lactiplantibacillus argentoratensis* | NIAB_CM10C01 | Animal |
| GCF_050445345.1 | *Lactiplantibacillus argentoratensis* | FU4 | Others |
| GCF_050847345.1 | *Lactiplantibacillus argentoratensis* | 12-27B | Food |
| GCF_051154035.1 | *Lactiplantibacillus argentoratensis* | BE1101-6-22 | Environment |
| GCF_051154075.1 | *Lactiplantibacillus argentoratensis* | BE1101-6-19 | Environment |
| GCF_051154095.1 | *Lactiplantibacillus argentoratensis* | BE1101-6-16 | Environment |
| GCF_051154225.1 | *Lactiplantibacillus argentoratensis* | BE1101-5-26 | Environment |
| GCF_051154335.1 | *Lactiplantibacillus argentoratensis* | BE1101-5-16 | Environment |
| GCF_051154525.1 | *Lactiplantibacillus argentoratensis* | BE1010-6-1 | Environment |
| GCF_051154545.1 | *Lactiplantibacillus argentoratensis* | BE1010-5-8 | Environment |
| GCF_051154605.1 | *Lactiplantibacillus argentoratensis* | BE1010-5-38 | Environment |
| GCF_051154645.1 | *Lactiplantibacillus argentoratensis* | BE1010-5-35 | Environment |
| GCF_051154705.1 | *Lactiplantibacillus argentoratensis* | BE1010-5-34 | Environment |
| GCF_051156695.1 | *Lactiplantibacillus argentoratensis* | BE091028-5-34 | Environment |
| GCF_051157395.1 | *Lactiplantibacillus argentoratensis* | BE090929-6-32 | Environment |
| GCF_051158255.1 | *Lactiplantibacillus argentoratensis* | BE090929-5-33 | Environment |
| GCF_051390895.1 | *Lactiplantibacillus argentoratensis* | DHuNHHMY9L1 | Food |
| GCF_051391295.1 | *Lactiplantibacillus argentoratensis* | FHNMY22M4 | Human |
| GCF_051391515.1 | *Lactiplantibacillus argentoratensis* | QHLJZD13L6 | Food |
| GCF_000469115.1 | *Lactiplantibacillus paraplantarum* | AY01 | Food |
| GCF_000758145.1 | *Lactiplantibacillus paraplantarum* | L-ZS9 | Food |
| GCF_001443645.1 | *Lactiplantibacillus paraplantarum* | L-ZS9 | Food |
| GCF_002897175.1 | *Lactiplantibacillus paraplantarum* | D2-1 | Others |
| GCF_003045685.1 | *Lactiplantibacillus paraplantarum* | AS-7 | Food |
| GCF_003346405.1 | *Lactiplantibacillus paraplantarum* | KMB_599 | Animal |
| GCF_003609655.1 | *Lactiplantibacillus paraplantarum* | Lp109 | Animal |
| GCF_003641145.1 | *Lactiplantibacillus paraplantarum* | DSM 10667 | Food |
| GCF_007991915.1 | *Lactiplantibacillus paraplantarum* | NBRC 107151 | Others |
| GCF_013113675.1 | *Lactiplantibacillus paraplantarum* | CK401 | Food |
| GCF_021869545.1 | *Lactiplantibacillus paraplantarum* | KCCM 11826P | Food |
| GCF_025212025.1 | *Lactiplantibacillus paraplantarum* | CIRM-BIA 1870 | Environment |
| GCF_025515415.1 | *Lactiplantibacillus paraplantarum* | SRCM210917 | Food |
| GCF_025950315.1 | *Lactiplantibacillus paraplantarum* | SRCM210393 | Food |
| GCF_029025825.1 | *Lactiplantibacillus paraplantarum* | FL-8 | Food |
| GCF_029391915.1 | *Lactiplantibacillus paraplantarum* | RX-8 | Environment |
| GCF_030262435.1 | *Lactiplantibacillus paraplantarum* | BPF2 | Food |
| GCF_037283745.1 | *Lactiplantibacillus paraplantarum* | A1453 | Others |
| GCF_038592875.1 | *Lactiplantibacillus paraplantarum* | lb01 | Food |
| GCF_040705215.1 | *Lactiplantibacillus paraplantarum* | CIRM-BIA 1870 | Environment |
| GCF_041107695.1 | *Lactiplantibacillus paraplantarum* | BPF2 | Others |
| GCF_041147075.1 | *Lactiplantibacillus paraplantarum* | LA1049 | Food |
| GCF_048588285.1 | *Lactiplantibacillus paraplantarum* | SDN1.2 | Animal |
| GCF_051098045.1 | *Lactiplantibacillus paraplantarum* | IBB3436 | Animal |
| GCF_001188985.1 | *Lactiplantibacillus pentosus* | FL0421 | Environment |
| GCF_002211885.1 | *Lactiplantibacillus pentosus* | SLC13 | Food |
| GCF_002751855.1 | *Lactiplantibacillus pentosus* | RI-031 | Food |
| GCF_002850015.1 | *Lactiplantibacillus pentosus* | BGM48 | Food |
| GCF_002993385.1 | *Lactiplantibacillus pentosus* | IG4 | Food |
| GCF_002993395.1 | *Lactiplantibacillus pentosus* | IG7 | Food |
| GCF_002993425.1 | *Lactiplantibacillus pentosus* | IG3 | Food |
| GCF_002993435.1 | *Lactiplantibacillus pentosus* | IG5 | Food |
| GCF_002993465.1 | *Lactiplantibacillus pentosus* | IG2 | Food |
| GCF_002993485.1 | *Lactiplantibacillus pentosus* | IG6 | Food |
| GCF_003627295.1 | *Lactiplantibacillus pentosus* | ZFM222 | Food |
| GCF_003627375.1 | *Lactiplantibacillus pentosus* | ZFM94 | Human |
| GCF_003641185.1 | *Lactiplantibacillus pentosus* | DSM 20314 | Others |
| GCF_003702565.1 | *Lactiplantibacillus pentosus* | IG12 | Others |
| GCF_003702605.1 | *Lactiplantibacillus pentosus* | IG11 | Others |
| GCF_003702625.1 | *Lactiplantibacillus pentosus* | IG8 | Others |
| GCF_003702635.1 | *Lactiplantibacillus pentosus* | IG10 | Others |
| GCF_003702665.1 | *Lactiplantibacillus pentosus* | IG9 | Others |
| GCF_004354685.1 | *Lactiplantibacillus pentosus* | ATCC 8041 | Others |
| GCF_009295675.1 | *Lactiplantibacillus pentosus* | krglsrbmofpi2 | Others |
| GCF_009812415.1 | *Lactiplantibacillus pentosus* | DZ35 | Food |
| GCF_016804305.1 | *Lactiplantibacillus pentosus* | MS031 | Food |
| GCF_017742275.1 | *Lactiplantibacillus pentosus* | 06B0048 | Food |
| GCF_018069575.1 | *Lactiplantibacillus pentosus* | B8 | Environment |
| GCF_018403455.1 | *Lactiplantibacillus pentosus* | missing | Others |
| GCF_018982875.1 | *Lactiplantibacillus pentosus* | O17 | Food |
| GCF_018991285.1 | *Lactiplantibacillus pentosus* | 1.2.11 | Food |
| GCF_018991345.1 | *Lactiplantibacillus pentosus* | 1.2.13 | Food |
| GCF_018991355.1 | *Lactiplantibacillus pentosus* | 1.8.6 | Food |
| GCF_018991365.1 | *Lactiplantibacillus pentosus* | 1.2.7 | Food |
| GCF_018991425.1 | *Lactiplantibacillus pentosus* | 14.2.3 | Food |
| GCF_018991465.1 | *Lactiplantibacillus pentosus* | 7.8.46 | Food |
| GCF_018991535.1 | *Lactiplantibacillus pentosus* | 7.8.2 | Food |
| GCF_018991565.1 | *Lactiplantibacillus pentosus* | 3.8.45 | Food |
| GCF_018993275.1 | *Lactiplantibacillus pentosus* | 7.8.11 | Food |
| GCF_018993345.1 | *Lactiplantibacillus pentosus* | 3.8.24 | Food |
| GCF_018993445.1 | *Lactiplantibacillus pentosus* | 7.2.15 | Food |
| GCF_018993485.1 | *Lactiplantibacillus pentosus* | 7.2.23 | Food |
| GCF_018993495.1 | *Lactiplantibacillus pentosus* | 3.2.37 | Food |
| GCF_018993525.1 | *Lactiplantibacillus pentosus* | 14.8.42 | Food |
| GCF_018993585.2 | *Lactiplantibacillus pentosus* | MU0445 | Food |
| GCF_018993625.1 | *Lactiplantibacillus pentosus* | 14.2.16 | Food |
| GCF_018993635.1 | *Lactiplantibacillus pentosus* | 1.8.18 | Food |
| GCF_018993665.1 | *Lactiplantibacillus pentosus* | 7.2.20 | Food |
| GCF_018993725.1 | *Lactiplantibacillus pentosus* | 3.2.36 | Food |
| GCF_018993945.1 | *Lactiplantibacillus pentosus* | 1.8.9 | Food |
| GCF_018993965.2 | *Lactiplantibacillus pentosus* | LA0445 | Food |
| GCF_020532005.1 | *Lactiplantibacillus pentosus* | GPS1-6 | Food |
| GCF_022701335.1 | *Lactiplantibacillus pentosus* | KZ0310 | Food |
| GCF_022936785.1 | *Lactiplantibacillus pentosus* | O12 | Food |
| GCF_022936805.1 | *Lactiplantibacillus pentosus* | O19 | Food |
| GCF_022936825.1 | *Lactiplantibacillus pentosus* | O11 | Food |
| GCF_023823145.1 | *Lactiplantibacillus pentosus* | 9D3 | Others |
| GCF_023972895.1 | *Lactiplantibacillus pentosus* | KW1 | Food |
| GCF_025190375.1 | *Lactiplantibacillus pentosus* | CIRM-BIA 2177 | Food |
| GCF_025398935.1 | *Lactiplantibacillus pentosus* | 68-1 | Food |
| GCF_026222675.1 | *Lactiplantibacillus pentosus* | OHF 23 | Human |
| GCF_027675785.1 | *Lactiplantibacillus pentosus* | UN03-35 | Human |
| GCF_027675885.1 | *Lactiplantibacillus pentosus* | UN03-28 | Human |
| GCF_027676485.1 | *Lactiplantibacillus pentosus* | UN03-143 | Human |
| GCF_028464285.1 | *Lactiplantibacillus pentosus* | YQ001 | Food |
| GCF_029228745.1 | *Lactiplantibacillus pentosus* | P2000 | Food |
| GCF_029542285.1 | *Lactiplantibacillus pentosus* | EP3 | Others |
| GCF_029813085.1 | *Lactiplantibacillus pentosus* | K0802 | Food |
| GCF_030480485.1 | *Lactiplantibacillus pentosus* | LB-1 | Food |
| GCF_030489685.1 | *Lactiplantibacillus pentosus* | LB-2 | Food |
| GCF_030549395.1 | *Lactiplantibacillus pentosus* | B652 | Food |
| GCF_030578355.1 | *Lactiplantibacillus pentosus* | LNP1-39 | Food |
| GCF_032190575.1 | *Lactiplantibacillus pentosus* | 7.2.15 | Food |
| GCF_032190655.1 | *Lactiplantibacillus pentosus* | 14.8.42 | Food |
| GCF_032190675.1 | *Lactiplantibacillus pentosus* | 7.8.46 | Food |
| GCF_032190695.1 | *Lactiplantibacillus pentosus* | 1.8.9 | Food |
| GCF_032190735.1 | *Lactiplantibacillus pentosus* | 1.8.18 | Food |
| GCF_032195525.1 | *Lactiplantibacillus pentosus* | 7.8.2 | Food |
| GCF_038447725.1 | *Lactiplantibacillus pentosus* | S3T60C | Food |
| GCF_039612585.1 | *Lactiplantibacillus pentosus* | XB7 | Human |
| GCF_039612645.1 | *Lactiplantibacillus pentosus* | B7 | Human |
| GCF_040969965.1 | *Lactiplantibacillus pentosus* | PCZ4 | Food |
| GCF_043593105.1 | *Lactiplantibacillus pentosus* | 14.8.42 | Food |
| GCF_044142985.1 | *Lactiplantibacillus pentosus* | S2-VN | Environment |
| GCF_047779755.1 | *Lactiplantibacillus pentosus* | P7 | Food |
| GCF_049517695.1 | *Lactiplantibacillus pentosus* | NG3-L3 | Food |
| GCF_049517795.1 | *Lactiplantibacillus pentosus* | KY6-L10 | Food |
| GCF_049517815.1 | *Lactiplantibacillus pentosus* | KY6-L4 | Food |
| GCF_049517855.1 | *Lactiplantibacillus pentosus* | KY5-L10 | Food |
| GCF_049517915.1 | *Lactiplantibacillus pentosus* | KY5-L9 | Food |
| GCF_049517935.1 | *Lactiplantibacillus pentosus* | KY5-L8 | Food |
| GCF_049517975.1 | *Lactiplantibacillus pentosus* | KY5-L7 | Food |
| GCF_049518035.1 | *Lactiplantibacillus pentosus* | KY5-L6 | Food |
| GCF_049518055.1 | *Lactiplantibacillus pentosus* | KY5-L5 | Food |
| GCF_049518095.1 | *Lactiplantibacillus pentosus* | KY5-L4 | Food |
| GCF_049518115.1 | *Lactiplantibacillus pentosus* | KY5-L3 | Food |
| GCF_049518175.1 | *Lactiplantibacillus pentosus* | KY5-L2 | Food |
| GCF_049518215.1 | *Lactiplantibacillus pentosus* | KY5-L1 | Food |
| GCF_049518235.1 | *Lactiplantibacillus pentosus* | KY4-L10 | Food |
| GCF_049518295.1 | *Lactiplantibacillus pentosus* | KY4-L7 | Food |
| GCF_049518335.1 | *Lactiplantibacillus pentosus* | KY4-L5 | Food |
| GCF_049518355.1 | *Lactiplantibacillus pentosus* | KY4-L4 | Food |
| GCF_049523775.1 | *Lactiplantibacillus pentosus* | KY4-L1 | Food |
| GCF_049523795.1 | *Lactiplantibacillus pentosus* | HS3-L6 | Food |
| GCF_049523835.1 | *Lactiplantibacillus pentosus* | HS3-L5 | Food |
| GCF_049523895.1 | *Lactiplantibacillus pentosus* | HS3-L4 | Food |
| GCF_049523915.1 | *Lactiplantibacillus pentosus* | HS3-L3 | Food |
| GCF_049523935.1 | *Lactiplantibacillus pentosus* | HS3-L2 | Food |
| GCF_049524035.1 | *Lactiplantibacillus pentosus* | HS3-L1 | Food |
| GCF_049544935.1 | *Lactiplantibacillus pentosus* | YF1 | Food |
| GCF_050407965.1 | *Lactiplantibacillus pentosus* | TBRC 20328 | Food |
| GCF_050511075.1 | *Lactiplantibacillus pentosus* | S9C1 | Food |
| GCF_050858995.1 | *Lactiplantibacillus pentosus* | LTJ12 | Food |
| GCF_051388335.1 | *Lactiplantibacillus pentosus* | RS15-3 | Food |
| GCF_964063425.1 | *Lactiplantibacillus pentosus* | - | Others |
| GCF_001436855.1 | *Lactiplantibacillus plantarum* | AG30 | Others |
| GCF_009766165.1 | *Lactiplantibacillus plantarum* | Lp90 | Food |
| GCF_009766195.1 | *Lactiplantibacillus plantarum* | DmCS_001 | Food |
| GCF_001633255.1 | *Lactiplantibacillus plantarum* | FMNP01 | Animal |
| GCF_001633265.1 | *Lactiplantibacillus plantarum* | 90sk | Animal |
| GCF_001633285.1 | *Lactiplantibacillus plantarum* | B21 | Animal |
| GCF_001633775.1 | *Lactiplantibacillus plantarum* | CIP104448 | Animal |
| GCF_029874465.1 | *Lactiplantibacillus plantarum* | NL42 | Food |
| GCF_021559915.1 | *Lactiplantibacillus plantarum* | PS128 | Animal |
| GCF_026156765.1 | *Lactiplantibacillus plantarum* | 8 RA-3 | Food |
| GCF_026156785.1 | *Lactiplantibacillus plantarum* | L31-1 | Food |
| GCF_026156815.1 | *Lactiplantibacillus plantarum* | SNU.Lp177 | Food |
| GCF_018257075.1 | *Lactiplantibacillus plantarum* | 5-?.?. | Animal |
| GCF_018257115.1 | *Lactiplantibacillus plantarum* | ZS2058 | Animal |
| GCF_018257135.1 | *Lactiplantibacillus plantarum* | HFC8 | Animal |
| GCF_025122345.1 | *Lactiplantibacillus plantarum* | Nizo2877 | Animal |
| GCF_003325775.1 | *Lactiplantibacillus plantarum* | WLPL04 | Food |
| GCF_023702795.1 | *Lactiplantibacillus plantarum* | 80 | Food |
| GCF_023702905.1 | *Lactiplantibacillus plantarum* | DSM 13273 | Food |
| GCF_023702925.1 | *Lactiplantibacillus plantarum* | CRL 1506 | Food |
| GCF_023702945.1 | *Lactiplantibacillus plantarum* | SF2A35B | Food |
| GCF_023702995.1 | *Lactiplantibacillus plantarum* | LZ95 | Food |
| GCF_023703015.1 | *Lactiplantibacillus plantarum* | Lp1610 | Food |
| GCF_023703125.1 | *Lactiplantibacillus plantarum* | Lp1612 | Food |
| GCF_023703145.1 | *Lactiplantibacillus plantarum* | Zhang-LL | Food |
| GCF_023703155.1 | *Lactiplantibacillus plantarum* | 43-3 | Food |
| GCF_023703175.1 | *Lactiplantibacillus plantarum* | JBE245 | Food |
| GCF_023703235.1 | *Lactiplantibacillus plantarum* | E2C2 | Food |
| GCF_023703265.1 | *Lactiplantibacillus plantarum* | E2C5 | Food |
| GCF_023702885.1 | *Lactiplantibacillus plantarum* | CAUH2 | Food |
| GCF_023702965.1 | *Lactiplantibacillus plantarum* | FBR5 | Food |
| GCF_030646455.1 | *Lactiplantibacillus plantarum* | FBR4 | Food |
| GCF_030504815.1 | *Lactiplantibacillus plantarum* | FBR6 | Food |
| GCF_009914805.1 | *Lactiplantibacillus plantarum* | ER | Animal |
| GCF_001484005.1 | *Lactiplantibacillus plantarum* | NAB2 | Human |
| GCF_031876755.1 | *Lactiplantibacillus plantarum* | 19.1 | Food |
| GCF_031877725.1 | *Lactiplantibacillus plantarum* | CNW10 | Food |
| GCF_048890125.1 | *Lactiplantibacillus plantarum* | Nizo2484 | Food |
| GCF_031296365.1 | *Lactiplantibacillus plantarum* | Nizo2485 | Food |
| GCF_013808505.1 | *Lactiplantibacillus plantarum* | Nizo2494 | Food |
| GCF_045800705.1 | *Lactiplantibacillus plantarum* | Nizo2726 | Food |
| GCF_045800735.1 | *Lactiplantibacillus plantarum* | Nizo2535 | Food |
| GCF_017581045.1 | *Lactiplantibacillus plantarum* | Nizo2741 | Food |
| GCF_002532125.1 | *Lactiplantibacillus plantarum* | Nizo2753 | Food |
| GCF_046736505.1 | *Lactiplantibacillus plantarum* | Nizo2757 | Animal |
| GCF_026930305.1 | *Lactiplantibacillus plantarum* | Nizo2766 | Human |
| GCF_026930585.1 | *Lactiplantibacillus plantarum* | Nizo2830 | Human |
| GCF_049477935.1 | *Lactiplantibacillus plantarum* | Nizo2802 | Human |
| GCF_028201575.1 | *Lactiplantibacillus plantarum* | Nizo2801 | Human |
| GCF_040790815.1 | *Lactiplantibacillus plantarum* | Nizo2814 | Human |
| GCF_040790905.1 | *Lactiplantibacillus plantarum* | Nizo2831 | Human |
| GCF_001331925.2 | *Lactiplantibacillus plantarum* | Nizo2806 | Human |
| GCF_017580975.1 | *Lactiplantibacillus plantarum* | Nizo2855 | Environment |
| GCF_002165725.1 | *Lactiplantibacillus plantarum* | Nizo2891 | Environment |
| GCF_002943545.1 | *Lactiplantibacillus plantarum* | Nizo2889 | Food |
| GCF_034426955.1 | *Lactiplantibacillus plantarum* | Nizo3400 | Food |
| GCF_047204915.1 | *Lactiplantibacillus plantarum* | Nizo3894 | Animal |
| GCF_017580905.1 | *Lactiplantibacillus plantarum* | Nizo3892 | Food |
| GCF_017580925.1 | *Lactiplantibacillus plantarum* | NAB1 | Food |
| GCF_003346075.1 | *Lactiplantibacillus plantarum* | Nizo2256 | Food |
| GCF_003346085.1 | *Lactiplantibacillus plantarum* | Nizo1838 | Food |
| GCF_003346105.1 | *Lactiplantibacillus plantarum* | Nizo1839 | Food |
| GCF_049241955.1 | *Lactiplantibacillus plantarum* | Nizo1840 | Food |
| GCF_001649985.1 | *Lactiplantibacillus plantarum* | Nizo2029 | Animal |
| GCF_001633415.1 | *Lactiplantibacillus plantarum* | Nizo2257 | Food |
| GCF_001982115.1 | *Lactiplantibacillus plantarum* | Nizo2258 | Others |
| GCF_001982165.1 | *Lactiplantibacillus plantarum* | Nizo2259 | Others |
| GCF_001982195.1 | *Lactiplantibacillus plantarum* | Nizo2260 | Others |
| GCF_001982205.1 | *Lactiplantibacillus plantarum* | Nizo2262 | Others |
| GCF_001982265.1 | *Lactiplantibacillus plantarum* | Nizo2263 | Others |
| GCF_001982335.1 | *Lactiplantibacillus plantarum* | Nizo2264 | Others |
| GCF_001982345.1 | *Lactiplantibacillus plantarum* | Nizo2457 | Others |
| GCF_001982385.1 | *Lactiplantibacillus plantarum* | UC8491 | Others |
| GCF_001982405.1 | *Lactiplantibacillus plantarum* | NRCC1 | Others |
| GCF_017581065.1 | *Lactiplantibacillus plantarum* | Nizo1837 | Environment |
| GCF_036259715.1 | *Lactiplantibacillus plantarum* | LZ206 | Animal |
| GCF_010092485.1 | *Lactiplantibacillus plantarum* | LZ227 | Animal |
| GCF_018138185.1 | *Lactiplantibacillus plantarum* | MPL16 | Others |
| GCF_018138205.1 | *Lactiplantibacillus plantarum* | SRCM101060 | Others |
| GCF_018138245.1 | *Lactiplantibacillus plantarum* | NCU116 | Others |
| GCF_018138265.1 | *Lactiplantibacillus plantarum* | TL2766 | Others |
| GCF_964186755.1 | *Lactiplantibacillus plantarum* | KP | Others |
| GCF_964186795.1 | *Lactiplantibacillus plantarum* | DF | Others |
| GCF_964186815.1 | *Lactiplantibacillus plantarum* | PC520 | Others |
| GCF_964187105.1 | *Lactiplantibacillus plantarum* | XJ25 | Others |
| GCF_045348195.1 | *Lactiplantibacillus plantarum* | LY-78 | Others |
| GCF_039938975.1 | *Lactiplantibacillus plantarum* | JSA22 | Human |
| GCF_047224495.1 | *Lactiplantibacillus plantarum* | Kanjika2007 | Human |
| GCF_038024955.1 | *Lactiplantibacillus plantarum* | CGMCC 8198 | Food |
| GCF_001633545.1 | *Lactiplantibacillus plantarum* | LL441 | Food |
| GCF_002005385.2 | *Lactiplantibacillus plantarum* | C410L1 | Food |
| GCF_004368485.1 | *Lactiplantibacillus plantarum* | MF1298 | Food |
| GCF_012689225.1 | *Lactiplantibacillus plantarum* | I61 | Food |
| GCF_039871905.1 | *Lactiplantibacillus plantarum* | P22 | Food |
| GCF_040356725.1 | *Lactiplantibacillus plantarum* | K35 | Food |
| GCF_964200455.1 | *Lactiplantibacillus plantarum* | P31 | Food |
| GCF_005864275.1 | *Lactiplantibacillus plantarum* | P42 | Others |
| GCF_900695295.1 | *Lactiplantibacillus plantarum* | P26 | Food |
| GCF_900695365.1 | *Lactiplantibacillus plantarum* | P62 | Food |
| GCF_037966755.1 | *Lactiplantibacillus plantarum* | P67 | Environment |
| GCF_900695425.1 | *Lactiplantibacillus plantarum* | P86 | Food |
| GCF_900700275.1 | *Lactiplantibacillus plantarum* | P73 | Food |
| GCF_033802805.1 | *Lactiplantibacillus plantarum* | P76 | Food |
| GCF_036321505.1 | *Lactiplantibacillus plantarum* | MHO2.4 | Food |
| GCF_002576835.1 | *Lactiplantibacillus plantarum* | MHO2.5 | Food |
| GCF_016775685.1 | *Lactiplantibacillus plantarum* | MHO2.9 | Food |
| GCF_030464405.1 | *Lactiplantibacillus plantarum* | A3 | Food |
| GCF_030464575.1 | *Lactiplantibacillus plantarum* | I08 | Food |
| GCF_030464605.1 | *Lactiplantibacillus plantarum* | CIF17A2 | Food |
| GCF_030464625.1 | *Lactiplantibacillus plantarum* | CIF17A4 | Food |
| GCF_030464645.1 | *Lactiplantibacillus plantarum* | CIF17A5 | Food |
| GCF_030464665.1 | *Lactiplantibacillus plantarum* | CIF17AN2 | Food |
| GCF_030464685.1 | *Lactiplantibacillus plantarum* | DSM 2601 | Food |
| GCF_030464705.1 | *Lactiplantibacillus plantarum* | DSM 20246 | Food |
| GCF_002370985.1 | *Lactiplantibacillus plantarum* | CIF17AN8 | Food |
| GCF_009889835.1 | *Lactiplantibacillus plantarum* | TISTR875 | Food |
| GCF_009889885.1 | *Lactiplantibacillus plantarum* | 299v | Food |
| GCF_009889895.1 | *Lactiplantibacillus plantarum* | K36 | Food |
| GCF_009889925.1 | *Lactiplantibacillus plantarum* | P14 | Food |
| GCF_009889935.1 | *Lactiplantibacillus plantarum* | RI-162 | Food |
| GCF_009889965.1 | *Lactiplantibacillus plantarum* | RI-165 | Food |
| GCF_009889995.1 | *Lactiplantibacillus plantarum* | RI-048 | Food |
| GCF_001704645.1 | *Lactiplantibacillus plantarum* | RI-012 | Food |
| GCF_009890025.1 | *Lactiplantibacillus plantarum* | RI-011 | Food |
| GCF_009890045.1 | *Lactiplantibacillus plantarum* | RI-190 | Food |
| GCF_026689375.1 | *Lactiplantibacillus plantarum* | RI-189 | Food |
| GCF_047468855.1 | *Lactiplantibacillus plantarum* | RI-405 | Environment |
| GCF_016029535.1 | *Lactiplantibacillus plantarum* | RI-208 | Food |
| GCF_022844655.1 | *Lactiplantibacillus plantarum* | RI-422 | Food |
| GCF_001651845.1 | *Lactiplantibacillus plantarum* | RI-408 | Human |
| GCF_002286275.1 | *Lactiplantibacillus plantarum* | RI-086 | Human |
| GCF_003053025.1 | *Lactiplantibacillus plantarum* | RI-123 | Human |
| GCF_003053035.1 | *Lactiplantibacillus plantarum* | RI-140 | Human |
| GCF_003053045.1 | *Lactiplantibacillus plantarum* | RI-506 | Human |
| GCF_003053165.1 | *Lactiplantibacillus plantarum* | RI-266 | Human |
| GCF_003053185.1 | *Lactiplantibacillus plantarum* | RI-507 | Human |
| GCF_003061725.1 | *Lactiplantibacillus plantarum* | RI-510 | Human |
| GCF_003061765.1 | *Lactiplantibacillus plantarum* | RI-511 | Human |
| GCF_003061785.1 | *Lactiplantibacillus plantarum* | RI-515 | Human |
| GCF_003061805.1 | *Lactiplantibacillus plantarum* | RI-139 | Human |
| GCF_051153115.1 | *Lactiplantibacillus plantarum* | RI-146 | Others |
| GCF_051153235.1 | *Lactiplantibacillus plantarum* | RI-147 | Others |
| GCF_051154105.1 | *Lactiplantibacillus plantarum* | RI-509 | Others |
| GCF_051154175.1 | *Lactiplantibacillus plantarum* | RI-505 | Others |
| GCF_051154215.1 | *Lactiplantibacillus plantarum* | RI-513 | Others |
| GCF_051154295.1 | *Lactiplantibacillus plantarum* | RI-514 | Others |
| GCF_051154415.1 | *Lactiplantibacillus plantarum* | RI-113 | Others |
| GCF_051154465.1 | *Lactiplantibacillus plantarum* | 10CH | Others |
| GCF_051154805.1 | *Lactiplantibacillus plantarum* | CLP0611 | Others |
| GCF_051154825.1 | *Lactiplantibacillus plantarum* | JBE490 | Others |
| GCF_051155135.1 | *Lactiplantibacillus plantarum* | LP2 | Others |
| GCF_051155175.1 | *Lactiplantibacillus plantarum* | BLS41 | Others |
| GCF_051155195.1 | *Lactiplantibacillus plantarum* | TMW 1.25 | Others |
| GCF_051155255.1 | *Lactiplantibacillus plantarum* | TMW 1.277 | Others |
| GCF_051155295.1 | *Lactiplantibacillus plantarum* | TMW 1.708 | Others |
| GCF_051155475.1 | *Lactiplantibacillus plantarum* | TMW 1.1623 | Others |
| GCF_051155515.1 | *Lactiplantibacillus plantarum* | O2T60C | Others |
| GCF_051155535.1 | *Lactiplantibacillus plantarum* | S11T3E | Others |
| GCF_051155615.1 | *Lactiplantibacillus plantarum* | SRCM102022 | Others |
| GCF_051156635.1 | *Lactiplantibacillus plantarum* | LPL-1 | Others |
| GCF_051156655.1 | *Lactiplantibacillus plantarum* | dm | Others |
| GCF_051156675.1 | *Lactiplantibacillus plantarum* | GB-LP1 | Others |
| GCF_022631455.1 | *Lactiplantibacillus plantarum* | 8 PA 3 | Environment |
| GCF_018991855.1 | *Lactiplantibacillus plantarum* | A6 | Others |
| GCF_018991195.2 | *Lactiplantibacillus plantarum* | LP3 | Food |
| GCF_018993285.1 | *Lactiplantibacillus plantarum* | BDGP2 | Food |
| GCF_018993325.1 | *Lactiplantibacillus plantarum* | Lp820 | Food |
| GCF_032190715.1 | *Lactiplantibacillus plantarum* | ATCC 8014 | Food |
| GCF_041147375.1 | *Lactiplantibacillus plantarum* | Lp510 | Food |
| GCF_018395655.2 | *Lactiplantibacillus plantarum* | NI326 | Food |
| GCF_001469145.1 | *Lactiplantibacillus plantarum* | D13 | Others |
| GCF_046352955.1 | *Lactiplantibacillus plantarum* | SF15C | Others |
| GCF_027585195.1 | *Lactiplantibacillus plantarum* | PC520 | Others |
| GCF_027585215.1 | *Lactiplantibacillus plantarum* | ATCC 8014 | Others |
| GCF_027585225.1 | *Lactiplantibacillus plantarum* | DietG20.1.2 | Others |
| GCF_027585245.1 | *Lactiplantibacillus plantarum* | ATCC 8014 | Others |
| GCF_027585265.1 | *Lactiplantibacillus plantarum* | RI-393 | Others |
| GCF_027585285.1 | *Lactiplantibacillus plantarum* | RI-191 | Others |
| GCF_027585315.1 | *Lactiplantibacillus plantarum* | RI-203 | Others |
| GCF_027585395.1 | *Lactiplantibacillus plantarum* | RI-265 | Others |
| GCF_019469465.1 | *Lactiplantibacillus plantarum* | KC3 | Food |
| GCF_049190885.1 | *Lactiplantibacillus plantarum* | K259 | Food |
| GCF_009889975.1 | *Lactiplantibacillus plantarum* | LM1004 | Food |
| GCF_009890055.1 | *Lactiplantibacillus plantarum* | BP06 | Food |
| GCF_032602185.1 | *Lactiplantibacillus plantarum* | JMCC0013 | Food |
| GCF_012272935.1 | *Lactiplantibacillus plantarum* | X7021 | Food |
| GCF_051647865.1 | *Lactiplantibacillus plantarum* | KC28 | Others |
| GCF_025133105.1 | *Lactiplantibacillus plantarum* | C4 | Environment |
| GCF_002750575.1 | *Lactiplantibacillus plantarum* | K25 | Food |
| GCF_000966475.1 | *Lactiplantibacillus plantarum* | JDARSH | Food |
| GCF_039954855.1 | *Lactiplantibacillus plantarum* | AS-10 | Food |
| GCF_041725645.1 | *Lactiplantibacillus plantarum* | AS-8 | Food |
| GCF_023973045.1 | *Lactiplantibacillus plantarum* | AS-9 | Food |
| GCF_048817855.1 | *Lactiplantibacillus plantarum* | AS-6 | Food |
| GCF_002631775.1 | *Lactiplantibacillus plantarum* | PFC-311 | Others |
| GCF_009937825.1 | *Lactiplantibacillus plantarum* | DS8_9 | Food |
| GCF_052412495.1 | *Lactiplantibacillus plantarum* | DS18_9 | Food |
| GCF_025190125.1 | *Lactiplantibacillus plantarum* | DS23_9 | Food |
| GCF_034321885.1 | *Lactiplantibacillus plantarum* | DS3_9 | Food |
| GCF_004102885.1 | *Lactiplantibacillus plantarum* | DS13_9 | Food |
| GCF_002737935.1 | *Lactiplantibacillus plantarum* | DS6_9 | Others |
| GCF_044731905.1 | *Lactiplantibacillus plantarum* | DS14_9 | Food |
| GCF_043673385.1 | *Lactiplantibacillus plantarum* | DS9_9 | Others |
| GCF_043951925.1 | *Lactiplantibacillus plantarum* | DS11_9 | Others |
| GCF_038096155.1 | *Lactiplantibacillus plantarum* | plantarum | Environment |
| GCF_040705195.1 | *Lactiplantibacillus plantarum* | LQ80 | Environment |
| GCF_002024845.1 | *Lactiplantibacillus plantarum* | HAC01 | Environment |
| GCF_026127625.1 | *Lactiplantibacillus plantarum* | EBKLp545 | Others |
| GCF_001302645.1 | *Lactiplantibacillus plantarum* | SK151 | Human |
| GCF_023348465.1 | *Lactiplantibacillus plantarum* | DSR_M2 | Human |
| GCF_051371445.1 | *Lactiplantibacillus plantarum* | nF1 | Animal |
| GCF_030464385.1 | *Lactiplantibacillus plantarum* | CRL 681 | Human |
| GCF_030464445.1 | *Lactiplantibacillus plantarum* | CCFM605 | Human |
| GCF_030464465.1 | *Lactiplantibacillus plantarum* | CGMCC12436 | Human |
| GCF_030464485.1 | *Lactiplantibacillus plantarum* | TMW 1.1478 | Human |
| GCF_030464495.1 | *Lactiplantibacillus plantarum* | KMB_621 | Human |
| GCF_030464525.1 | *Lactiplantibacillus plantarum* | KMB_618 | Human |
| GCF_030464545.1 | *Lactiplantibacillus plantarum* | KMB_614 | Human |
| GCF_030464725.1 | *Lactiplantibacillus plantarum* | KMB_597 | Human |
| GCF_030464765.1 | *Lactiplantibacillus plantarum* | b-2 | Human |
| GCF_030466465.1 | *Lactiplantibacillus plantarum* | IDCC3501 | Human |
| GCF_050110895.1 | *Lactiplantibacillus plantarum* | AM25-25 | Human |
| GCF_050613195.1 | *Lactiplantibacillus plantarum* | AM25-20AC | Animal |
| GCF_033792285.1 | *Lactiplantibacillus plantarum* | TJA26B | Animal |
| GCF_033792305.1 | *Lactiplantibacillus plantarum* | C29 | Animal |
| GCF_033792335.1 | *Lactiplantibacillus plantarum* | DR7 | Animal |
| GCF_011009755.1 | *Lactiplantibacillus plantarum* | ZFM55 | Animal |
| GCF_026421325.1 | *Lactiplantibacillus plantarum* | ATG-K6 | Human |
| GCF_051225605.1 | *Lactiplantibacillus plantarum* | ATG-K8 | Animal |
| GCF_003999275.1 | *Lactiplantibacillus plantarum* | ATG-K2 | Animal |
| GCF_024969715.1 | *Lactiplantibacillus plantarum* | NCIMB 700965 | Animal |
| GCF_024970125.1 | *Lactiplantibacillus plantarum* | ZFM9 | Animal |
| GCF_024970145.1 | *Lactiplantibacillus plantarum* | ZFM4 | Animal |
| GCF_024970165.1 | *Lactiplantibacillus plantarum* | KACC 92189 | Animal |
| GCF_039871345.1 | *Lactiplantibacillus plantarum* | UBLP-40 | Animal |
| GCF_001595615.1 | *Lactiplantibacillus plantarum* | NF92 | Human |
| GCF_003469805.1 | *Lactiplantibacillus plantarum* | LMT1-48 | Human |
| GCF_003470765.1 | *Lactiplantibacillus plantarum* | SN35N | Human |
| GCF_004300925.1 | *Lactiplantibacillus plantarum* | Curd | Human |
| GCF_004300935.1 | *Lactiplantibacillus plantarum* | FBL-3a | Human |
| GCF_004300945.1 | *Lactiplantibacillus plantarum* | Q7 | Human |
| GCF_004300955.1 | *Lactiplantibacillus plantarum* | KB1253 | Human |
| GCF_004300965.1 | *Lactiplantibacillus plantarum* | SKT109 | Human |
| GCF_004301025.1 | *Lactiplantibacillus plantarum* | YW11 | Human |
| GCF_004301035.1 | *Lactiplantibacillus plantarum* | 13_3 | Human |
| GCF_004301045.1 | *Lactiplantibacillus plantarum* | 12_3 | Human |
| GCF_004301055.1 | *Lactiplantibacillus plantarum* | SRCM103287 | Human |
| GCF_004301075.1 | *Lactiplantibacillus plantarum* | SRCM 103305 | Human |
| GCF_004301125.1 | *Lactiplantibacillus plantarum* | SRCM 103292 | Human |
| GCF_004301135.1 | *Lactiplantibacillus plantarum* | SRCM103406 | Human |
| GCF_004301145.1 | *Lactiplantibacillus plantarum* | SRCM103411 | Human |
| GCF_004301155.1 | *Lactiplantibacillus plantarum* | SRCM103295 | Human |
| GCF_004301205.1 | *Lactiplantibacillus plantarum* | SRCM103311 | Human |
| GCF_004301225.1 | *Lactiplantibacillus plantarum* | SRCM103357 | Human |
| GCF_004301235.1 | *Lactiplantibacillus plantarum* | SRCM103361 | Human |
| GCF_004301245.1 | *Lactiplantibacillus plantarum* | SRCM103362 | Human |
| GCF_004301255.1 | *Lactiplantibacillus plantarum* | SRCM103418 | Human |
| GCF_004301305.1 | *Lactiplantibacillus plantarum* | SRCM103426 | Human |
| GCF_004301325.1 | *Lactiplantibacillus plantarum* | T9 | Human |
| GCF_004301335.1 | *Lactiplantibacillus plantarum* | D31 | Human |
| GCF_004301345.1 | *Lactiplantibacillus plantarum* | SRCM103472 | Human |
| GCF_004301355.1 | *Lactiplantibacillus plantarum* | SRCM103473 | Human |
| GCF_004301395.1 | *Lactiplantibacillus plantarum* | dkp1 | Human |
| GCF_004301425.1 | *Lactiplantibacillus plantarum* | B-1 | Human |
| GCF_004301435.1 | *Lactiplantibacillus plantarum* | YW32 | Human |
| GCF_004301445.1 | *Lactiplantibacillus plantarum* | XZ3303 | Human |
| GCF_004301455.1 | *Lactiplantibacillus plantarum* | SRCM103297 | Human |
| GCF_004301485.1 | *Lactiplantibacillus plantarum* | SRCM103300 | Human |
| GCF_004301545.1 | *Lactiplantibacillus plantarum* | SRCM103303 | Human |
| GCF_004301555.1 | *Lactiplantibacillus plantarum* | T9 | Human |
| GCF_004301565.1 | *Lactiplantibacillus plantarum* | E6-1 | Human |
| GCF_004301585.1 | *Lactiplantibacillus plantarum* | E6-2 | Human |
| GCF_004301595.1 | *Lactiplantibacillus plantarum* | E6-4 | Human |
| GCF_004301645.1 | *Lactiplantibacillus plantarum* | E6-5 | Human |
| GCF_004301655.1 | *Lactiplantibacillus plantarum* | E6-3 | Human |
| GCF_004301685.1 | *Lactiplantibacillus plantarum* | M4-2 | Human |
| GCF_004301695.1 | *Lactiplantibacillus plantarum* | E6-6 | Human |
| GCF_004301705.1 | *Lactiplantibacillus plantarum* | M4-1 | Human |
| GCF_004301725.1 | *Lactiplantibacillus plantarum* | M4-3 | Human |
| GCF_004301765.1 | *Lactiplantibacillus plantarum* | M4-4 | Human |
| GCF_004301775.1 | *Lactiplantibacillus plantarum* | Y2-2 | Human |
| GCF_004301795.1 | *Lactiplantibacillus plantarum* | Y2-1 | Human |
| GCF_004301805.1 | *Lactiplantibacillus plantarum* | Y2-3 | Human |
| GCF_004301815.1 | *Lactiplantibacillus plantarum* | Y2-4 | Human |
| GCF_004301865.1 | *Lactiplantibacillus plantarum* | Y2-5 | Human |
| GCF_011421665.1 | *Lactiplantibacillus plantarum* | A1-1 | Human |
| GCF_018784325.1 | *Lactiplantibacillus plantarum* | A2-1 | Human |
| GCF_021199095.1 | *Lactiplantibacillus plantarum* | A1-2 | Human |
| GCF_027557615.1 | *Lactiplantibacillus plantarum* | A2-2 | Human |
| GCF_033194695.1 | *Lactiplantibacillus plantarum* | A3-1 | Human |
| GCF_037414365.1 | *Lactiplantibacillus plantarum* | A3-2 | Human |
| GCF_037414405.1 | *Lactiplantibacillus plantarum* | A4-1 | Human |
| GCF_040914485.1 | *Lactiplantibacillus plantarum* | A4-2 | Human |
| GCF_040914525.1 | *Lactiplantibacillus plantarum* | A5-1 | Human |
| GCF_049330845.1 | *Lactiplantibacillus plantarum* | A5-2 | Human |
| GCF_004301875.1 | *Lactiplantibacillus plantarum* | A6-1 | Animal |
| GCF_004301885.1 | *Lactiplantibacillus plantarum* | A7-1 | Animal |
| GCF_004301905.1 | *Lactiplantibacillus plantarum* | A6-2 | Animal |
| GCF_004301915.1 | *Lactiplantibacillus plantarum* | A7-2 | Animal |
| GCF_004301955.1 | *Lactiplantibacillus plantarum* | A14-1 | Animal |
| GCF_004301975.1 | *Lactiplantibacillus plantarum* | B1-1 | Animal |
| GCF_004301995.1 | *Lactiplantibacillus plantarum* | A14-2 | Animal |
| GCF_004302005.1 | *Lactiplantibacillus plantarum* | B2-2 | Animal |
| GCF_004302035.1 | *Lactiplantibacillus plantarum* | B1-2 | Animal |
| GCF_004302055.1 | *Lactiplantibacillus plantarum* | B2-1 | Animal |
| GCF_004302065.1 | *Lactiplantibacillus plantarum* | B3-1 | Animal |
| GCF_004302105.1 | *Lactiplantibacillus plantarum* | B3-2 | Animal |
| GCF_004302115.1 | *Lactiplantibacillus plantarum* | B4-1 | Animal |
| GCF_004302125.1 | *Lactiplantibacillus plantarum* | B5-1 | Animal |
| GCF_004302135.1 | *Lactiplantibacillus plantarum* | B4-2 | Animal |
| GCF_004302185.1 | *Lactiplantibacillus plantarum* | B5-2 | Animal |
| GCF_004302195.1 | *Lactiplantibacillus plantarum* | B6-1 | Animal |
| GCF_004302205.1 | *Lactiplantibacillus plantarum* | B6-2 | Animal |
| GCF_004302215.1 | *Lactiplantibacillus plantarum* | B7-2 | Animal |
| GCF_004302255.1 | *Lactiplantibacillus plantarum* | B7-1 | Animal |
| GCF_004302285.1 | *Lactiplantibacillus plantarum* | B14-1 | Animal |
| GCF_004302295.1 | *Lactiplantibacillus plantarum* | B14-2 | Animal |
| GCF_004302305.1 | *Lactiplantibacillus plantarum* | R1-1-1 | Animal |
| GCF_004302315.1 | *Lactiplantibacillus plantarum* | R1-1-2 | Animal |
| GCF_004302345.1 | *Lactiplantibacillus plantarum* | R1-2-2 | Animal |
| GCF_004302385.1 | *Lactiplantibacillus plantarum* | R1-2-1 | Animal |
| GCF_004302395.1 | *Lactiplantibacillus plantarum* | R1-3-1 | Animal |
| GCF_004302405.1 | *Lactiplantibacillus plantarum* | R1-3-2 | Animal |
| GCF_004302415.1 | *Lactiplantibacillus plantarum* | R2-1-1 | Animal |
| GCF_004302445.1 | *Lactiplantibacillus plantarum* | R2-1-2 | Animal |
| GCF_004302485.1 | *Lactiplantibacillus plantarum* | R2-2-1 | Animal |
| GCF_004302495.1 | *Lactiplantibacillus plantarum* | R2-2-2 | Animal |
| GCF_004302505.1 | *Lactiplantibacillus plantarum* | R2-3-1 | Animal |
| GCF_004302515.1 | *Lactiplantibacillus plantarum* | R2-3-2 | Animal |
| GCF_004302535.1 | *Lactiplantibacillus plantarum* | R3-1-1 | Animal |
| GCF_004302585.1 | *Lactiplantibacillus plantarum* | R3-1-2 | Animal |
| GCF_004302615.1 | *Lactiplantibacillus plantarum* | R3-2-1 | Animal |
| GCF_004302625.1 | *Lactiplantibacillus plantarum* | R3-2-2 | Animal |
| GCF_004302645.1 | *Lactiplantibacillus plantarum* | R3-3-1 | Animal |
| GCF_004302685.1 | *Lactiplantibacillus plantarum* | R4-1-1 | Animal |
| GCF_004302695.1 | *Lactiplantibacillus plantarum* | R3-3-2 | Animal |
| GCF_004302715.1 | *Lactiplantibacillus plantarum* | R4-2-1 | Animal |
| GCF_004302725.1 | *Lactiplantibacillus plantarum* | R4-3-2 | Animal |
| GCF_004302745.1 | *Lactiplantibacillus plantarum* | R4-2-2 | Animal |
| GCF_024800605.1 | *Lactiplantibacillus plantarum* | R4-3-1 | Animal |
| GCF_024969905.1 | *Lactiplantibacillus plantarum* | R5-1-1 | Animal |
| GCF_008016845.1 | *Lactiplantibacillus plantarum* | R5-1-2 | Animal |
| GCF_008016855.1 | *Lactiplantibacillus plantarum* | R5-2-1 | Animal |
| GCF_008016925.1 | *Lactiplantibacillus plantarum* | R5-2-2 | Animal |
| GCF_025388655.1 | *Lactiplantibacillus plantarum* | R5-3-2 | Human |
| GCF_025388695.1 | *Lactiplantibacillus plantarum* | R5-3-1 | Human |
| GCF_025847695.1 | *Lactiplantibacillus plantarum* | R6-1-1 | Human |
| GCF_051390715.1 | *Lactiplantibacillus plantarum* | R6-2-1 | Human |
| GCF_051390735.1 | *Lactiplantibacillus plantarum* | R6-2-2 | Human |
| GCF_051390745.1 | *Lactiplantibacillus plantarum* | R6-1-2 | Human |
| GCF_051390775.1 | *Lactiplantibacillus plantarum* | R6-3-1 | Human |
| GCF_051390855.1 | *Lactiplantibacillus plantarum* | R6-3-2 | Human |
| GCF_051391375.1 | *Lactiplantibacillus plantarum* | R7-1-1 | Human |
| GCF_051391395.1 | *Lactiplantibacillus plantarum* | R7-2-1 | Human |
| GCF_051391415.1 | *Lactiplantibacillus plantarum* | R7-2-2 | Human |
| GCF_045271585.1 | *Lactiplantibacillus plantarum* | R14-1-1 | Human |
| GCF_030646105.1 | *Lactiplantibacillus plantarum* | R14-1-2 | Human |
| GCF_024732385.1 | *Lactiplantibacillus plantarum* | R14-2-1 | Human |
| GCF_029854335.1 | *Lactiplantibacillus plantarum* | R14-3-2 | Food |
| GCF_001633675.1 | *Lactiplantibacillus plantarum* | R14-2-2 | Food |
| GCF_036287535.1 | *Lactiplantibacillus plantarum* | R14-3-1 | Food |
| GCF_019641415.1 | *Lactiplantibacillus plantarum* | IRG1 | Food |
| GCF_001639455.1 | *Lactiplantibacillus plantarum* | NCIMB700965.EF.A | Food |
| GCF_029849115.1 | *Lactiplantibacillus plantarum* | EM | Food |
| GCF_030403475.1 | *Lactiplantibacillus plantarum* | ATCC 202195 | Food |
| GCF_030504705.1 | *Lactiplantibacillus plantarum* | INF 15D | Food |
| GCF_001715615.1 | *Lactiplantibacillus plantarum* | 8p-a3 | Food |
| GCF_021279005.2 | *Lactiplantibacillus plantarum* | 8p-a3-Clr | Food |
| GCF_002205775.2 | *Lactiplantibacillus plantarum* | FUA3590 | Food |
| GCF_003692725.1 | *Lactiplantibacillus plantarum* | UNQLp11 | Food |
| GCF_003990985.1 | *Lactiplantibacillus plantarum* | FAM 21789 | Food |
| GCF_014041895.1 | *Lactiplantibacillus plantarum* | NTTN08 | Food |
| GCF_019425695.1 | *Lactiplantibacillus plantarum* | YLBGNL-S7 | Food |
| GCF_023195495.1 | *Lactiplantibacillus plantarum* | pc-26 | Food |
| GCF_023278325.1 | *Lactiplantibacillus plantarum* | LLY-606 | Food |
| GCF_030549385.1 | *Lactiplantibacillus plantarum* | Y44 | Food |
| GCF_030549465.1 | *Lactiplantibacillus plantarum* | EML1 | Food |
| GCF_030575255.1 | *Lactiplantibacillus plantarum* | SA3 | Food |
| GCF_035336205.1 | *Lactiplantibacillus plantarum* | C1 | Food |
| GCF_035338015.1 | *Lactiplantibacillus plantarum* | BIO1096 | Food |
| GCF_037996605.1 | *Lactiplantibacillus plantarum* | TMW 1.1308 | Food |
| GCF_040583315.1 | *Lactiplantibacillus plantarum* | KCCP11226 | Food |
| GCF_039748295.1 | *Lactiplantibacillus plantarum* | BK-022 | Food |
| GCF_003545985.1 | *Lactiplantibacillus plantarum* | 83-18 | Food |
| GCF_003709415.1 | *Lactiplantibacillus plantarum* | 123-17 | Food |
| GCF_001754005.1 | *Lactiplantibacillus plantarum* | 8P-A3 | Food |
| GCF_030503695.1 | *Lactiplantibacillus plantarum* | IMAU20970 | Food |
| GCF_002948215.1 | *Lactiplantibacillus plantarum* | IMAU80873 | Food |
| GCF_030410355.1 | *Lactiplantibacillus plantarum* | LR46 | Food |
| GCF_019390115.1 | *Lactiplantibacillus plantarum* | LR39 | Food |
| GCF_019390145.1 | *Lactiplantibacillus plantarum* | LR14 | Food |
| GCF_048814805.1 | *Lactiplantibacillus plantarum* | FUA3584 | Food |
| GCF_045010365.1 | *Lactiplantibacillus plantarum* | TMW1.460 | Food |
| GCF_002920935.1 | *Lactiplantibacillus plantarum* | Lp835 | Food |
| GCF_022510025.2 | *Lactiplantibacillus plantarum* | LpRas | Food |
| GCF_023207995.1 | *Lactiplantibacillus plantarum* | Lp823 | Food |
| GCF_034258695.1 | *Lactiplantibacillus plantarum* | Lp546 | Food |
| GCF_039871925.1 | *Lactiplantibacillus plantarum* | Lp821 | Food |
| GCF_023195585.1 | *Lactiplantibacillus plantarum* | Lp543 | Food |
| GCF_039725565.1 | *Lactiplantibacillus plantarum* | Lp542 | Food |
| GCF_013694305.1 | *Lactiplantibacillus plantarum* | Lp533 | Food |
| GCF_013155145.1 | *Lactiplantibacillus plantarum* | Lp541 | Environment |
| GCF_017580935.1 | *Lactiplantibacillus plantarum* | Lp520 | Food |
| GCF_017581005.1 | *Lactiplantibacillus plantarum* | Lp309 | Food |
| GCF_013487805.1 | *Lactiplantibacillus plantarum* | Lp519 | Food |
| GCF_001633405.1 | *Lactiplantibacillus plantarum* | Lp218 | Food |
| GCF_014084065.1 | *Lactiplantibacillus plantarum* | Lp206 | Food |
| GCF_031085385.1 | *Lactiplantibacillus plantarum* | Lp305 | Food |
| GCF_046109905.1 | *Lactiplantibacillus plantarum* | SRCM100438 | Food |
| GCF_001990145.1 | *Lactiplantibacillus plantarum* | SRCM100440 | Food |
| GCF_023370155.1 | *Lactiplantibacillus plantarum* | SRCM100442 | Food |
| GCF_025133155.1 | *Lactiplantibacillus plantarum* | SRCM100995 | Food |
| GCF_041060785.1 | *Lactiplantibacillus plantarum* | SRCM101105 | Food |
| GCF_051123805.1 | *Lactiplantibacillus plantarum* | SRCM101187 | Food |
| GCF_001880185.2 | *Lactiplantibacillus plantarum* | SRCM101222 | Food |
| GCF_046843265.1 | *Lactiplantibacillus plantarum* | SRCM101518 | Food |
| GCF_049188355.1 | *Lactiplantibacillus plantarum* | SRCM102737 | Food |
| GCF_001633435.1 | *Lactiplantibacillus plantarum* | SRCM101167 | Food |
| GCF_001633485.1 | *Lactiplantibacillus plantarum* | SF9C | Food |
| GCF_001633495.1 | *Lactiplantibacillus plantarum* | EBKLp545 | Food |
| GCF_001278015.1 | *Lactiplantibacillus plantarum* | SRCM101511 | Food |
| GCF_001720285.1 | *Lactiplantibacillus plantarum* | CACC 558 | Food |
| GCF_051903995.1 | *Lactiplantibacillus plantarum* | 202195 | Food |
| GCF_017580955.1 | *Lactiplantibacillus plantarum* | P5 | Food |
| GCF_030758995.1 | *Lactiplantibacillus plantarum* | X7022 | Food |
| GCF_019038995.1 | *Lactiplantibacillus plantarum* | Tw226 | Food |
| GCF_023507555.1 | *Lactiplantibacillus plantarum* | LS/07 | Food |
| GCF_029764995.1 | *Lactiplantibacillus plantarum* | L12 | Food |
| GCF_029846785.1 | *Lactiplantibacillus plantarum* | L14 | Food |
| GCF_029906245.1 | *Lactiplantibacillus plantarum* | SPC-SNU 72-2 | Food |
| GCF_040717425.1 | *Lactiplantibacillus plantarum* | DMR 17 | Food |
| GCF_051391195.1 | *Lactiplantibacillus plantarum* | FH3 | Food |
| GCF_025190035.1 | *Lactiplantibacillus plantarum* | LP12418 | Food |
| GCF_039566255.1 | *Lactiplantibacillus plantarum* | AMT74419 | Food |
| GCF_039566515.1 | *Lactiplantibacillus plantarum* | CNEI-KCA4 | Food |
| GCF_039566665.1 | *Lactiplantibacillus plantarum* | TCI507 | Food |
| GCF_039566735.1 | *Lactiplantibacillus plantarum* | K03D08 | Food |
| GCF_039566835.1 | *Lactiplantibacillus plantarum* | Heal19 | Food |
| GCF_052217085.1 | *Lactiplantibacillus plantarum* | ZN-3 | Food |
| GCF_001643065.1 | *Lactiplantibacillus plantarum* | HC-2 | Food |
| GCF_002173655.1 | *Lactiplantibacillus plantarum* | BK-021 | Food |
| GCF_004054305.1 | *Lactiplantibacillus plantarum* | CNEI-KCA5 | Food |
| GCF_004055415.1 | *Lactiplantibacillus plantarum* | ATCC 5870 | Food |
| GCF_004055435.1 | *Lactiplantibacillus plantarum* | Lbio1 | Food |
| GCF_004078535.1 | *Lactiplantibacillus plantarum* | Lbio28 | Food |
| GCF_004078645.1 | *Lactiplantibacillus plantarum* | Lbio14 | Food |
| GCF_004087995.1 | *Lactiplantibacillus plantarum* | SK156 | Food |
| GCF_004101325.1 | *Lactiplantibacillus plantarum* | BCC9546 | Food |
| GCF_004101505.1 | *Lactiplantibacillus plantarum* | DSM 20174 | Food |
| GCF_004101545.1 | *Lactiplantibacillus plantarum* | Lsi | Food |
| GCF_004101605.1 | *Lactiplantibacillus plantarum* | Dan91 | Food |
| GCF_004101625.1 | *Lactiplantibacillus plantarum* | PMO08 | Food |
| GCF_004101645.1 | *Lactiplantibacillus plantarum* | DMR09 | Food |
| GCF_004103495.1 | *Lactiplantibacillus plantarum* | TK-P2A | Food |
| GCF_004103515.1 | *Lactiplantibacillus plantarum* | 1001095A_150126_D1 | Food |
| GCF_004141755.1 | *Lactiplantibacillus plantarum* | 1001254B_151014_C2 | Food |
| GCF_004141875.1 | *Lactiplantibacillus plantarum* | 1001254B_151014_A7 | Food |
| GCF_004141895.1 | *Lactiplantibacillus plantarum* | 1001287B_170213_A4 | Food |
| GCF_022568855.1 | *Lactiplantibacillus plantarum* | 1001216B_150713_H5 | Food |
| GCF_022568865.1 | *Lactiplantibacillus plantarum* | 1001254B_151014_B11 | Food |
| GCF_022568895.1 | *Lactiplantibacillus plantarum* | MK55 | Food |
| GCF_028462545.1 | *Lactiplantibacillus plantarum* | ZDY2013 | Food |
| GCF_028462565.1 | *Lactiplantibacillus plantarum* | g22 | Food |
| GCF_028462625.1 | *Lactiplantibacillus plantarum* | LPBF 35 | Food |
| GCF_038592865.1 | *Lactiplantibacillus plantarum* | PC518 | Food |
| GCF_040448705.1 | *Lactiplantibacillus plantarum* | SHY 21-2 | Food |
| GCF_044095195.1 | *Lactiplantibacillus plantarum* | PC518 | Food |
| GCF_044758965.1 | *Lactiplantibacillus plantarum* | S58 | Food |
| GCF_051712885.1 | *Lactiplantibacillus plantarum* | CXG9 | Food |
| GCF_049919675.1 | *Lactiplantibacillus plantarum* | KM2 | Food |
| GCF_037094665.1 | *Lactiplantibacillus plantarum* | Lp900 | Food |
| GCF_003045645.1 | *Lactiplantibacillus plantarum* | 12 | Food |
| GCF_003045665.1 | *Lactiplantibacillus plantarum* | AR195 | Food |
| GCF_003045705.1 | *Lactiplantibacillus plantarum* | KLDS1.0386 | Food |
| GCF_003045725.1 | *Lactiplantibacillus plantarum* | B1.3 | Food |
| GCF_001005805.1 | *Lactiplantibacillus plantarum* | B1.1 | Food |
| GCF_032248515.1 | *Lactiplantibacillus plantarum* | EL11 | Food |
| GCF_033802745.1 | *Lactiplantibacillus plantarum* | WS1.1 | Food |
| GCF_052416535.1 | *Lactiplantibacillus plantarum* | BGM37 | Others |
| GCF_040719475.1 | *Lactiplantibacillus plantarum* | AJ11 | Human |
| GCF_013753885.1 | *Lactiplantibacillus plantarum* | K4 | Human |
| GCF_001444495.1 | *Lactiplantibacillus plantarum* | 8.1 | Animal |
| GCF_025189815.1 | *Lactiplantibacillus plantarum* | 1B1 | Food |
| GCF_025189845.1 | *Lactiplantibacillus plantarum* | Lp-G18 | Food |
| GCF_025189865.1 | *Lactiplantibacillus plantarum* | LRCC5314 | Food |
| GCF_025189885.1 | *Lactiplantibacillus plantarum* | NCIMB8826 | Food |
| GCF_025189925.1 | *Lactiplantibacillus plantarum* | XSBN-3 | Food |
| GCF_025190145.1 | *Lactiplantibacillus plantarum* | XSBN-10 | Food |
| GCF_025190165.1 | *Lactiplantibacillus plantarum* | XSBN-13 | Food |
| GCF_025190185.1 | *Lactiplantibacillus plantarum* | P8L400 | Food |
| GCF_025190205.1 | *Lactiplantibacillus plantarum* | P8L200 | Food |
| GCF_025190225.1 | *Lactiplantibacillus plantarum* | P8N600 | Food |
| GCF_025212205.1 | *Lactiplantibacillus plantarum* | P8N200 | Food |
| GCF_021536745.2 | *Lactiplantibacillus plantarum* | P8N800 | Environment |
| GCF_001633595.1 | *Lactiplantibacillus plantarum* | P8N400 | Environment |
| GCF_001982285.1 | *Lactiplantibacillus plantarum* | P8L600 | Environment |
| GCF_047041085.1 | *Lactiplantibacillus plantarum* | P8L800 | Food |
| GCF_000764285.1 | *Lactiplantibacillus plantarum* | Dm-2019-33 | Food |
| GCF_014324175.1 | *Lactiplantibacillus plantarum* | Dm-2019-28 | Animal |
| GCF_013377705.1 | *Lactiplantibacillus plantarum* | Dm-2019-3 | Animal |
| GCF_030463605.1 | *Lactiplantibacillus plantarum* | Dm-2019-48 | Animal |
| GCF_051217015.1 | *Lactiplantibacillus plantarum* | ATCC 202195 | Human |
| GCF_051860185.1 | *Lactiplantibacillus plantarum* | GD00040 | Animal |
| GCF_027920405.1 | *Lactiplantibacillus plantarum* | COY2906 | Human |
| GCF_000830535.1 | *Lactiplantibacillus plantarum* | MCC636 | Human |
| GCF_006770485.1 | *Lactiplantibacillus plantarum* | 3.2.8 | Human |
| GCF_006770505.1 | *Lactiplantibacillus plantarum* | 3.2.8 | Human |
| GCF_001675425.1 | *Lactiplantibacillus plantarum* | 7.8.4 | Human |
| GCF_003589725.1 | *Lactiplantibacillus plantarum* | T1R2B | Human |
| GCF_003627335.1 | *Lactiplantibacillus plantarum* | QS7 | Human |
| GCF_003627355.1 | *Lactiplantibacillus plantarum* | XJ25 | Human |
| GCF_018351295.1 | *Lactiplantibacillus plantarum* | wikim 18 | Human |
| GCF_014878225.1 | *Lactiplantibacillus plantarum* | YT041 | Food |
| GCF_036416335.1 | *Lactiplantibacillus plantarum* | 8p-a3-Clr-Amx | Food |
| GCF_036353265.1 | *Lactiplantibacillus plantarum* | L75a | Food |
| GCF_003345375.1 | *Lactiplantibacillus plantarum* | 13A | Food |
| GCF_001308305.1 | *Lactiplantibacillus plantarum* | 41G | Food |
| GCF_020844685.1 | *Lactiplantibacillus plantarum* | 38I | Food |
| GCF_026127685.1 | *Lactiplantibacillus plantarum* | 41P | Food |
| GCF_009759845.1 | *Lactiplantibacillus plantarum* | DW12 | Human |
| GCF_009759825.1 | *Lactiplantibacillus plantarum* | MSD1 | Human |
| GCF_028895425.1 | *Lactiplantibacillus plantarum* | JZ6 | Human |
| GCF_028895435.1 | *Lactiplantibacillus plantarum* | LB244R | Human |
| GCF_028895445.1 | *Lactiplantibacillus plantarum* | LRCC5310 | Human |
| GCF_028895485.1 | *Lactiplantibacillus plantarum* | 1107 | Human |
| GCF_028895505.1 | *Lactiplantibacillus plantarum* | DSM 8862 | Human |
| GCF_028895525.1 | *Lactiplantibacillus plantarum* | LPKH | Human |
| GCF_028895545.1 | *Lactiplantibacillus plantarum* | NMZ-1139 | Human |
| GCF_028895555.1 | *Lactiplantibacillus plantarum* | R95 | Human |
| GCF_028895585.1 | *Lactiplantibacillus plantarum* | R75 | Human |
| GCF_028895595.1 | *Lactiplantibacillus plantarum* | R98 | Human |
| GCF_028895625.1 | *Lactiplantibacillus plantarum* | R77 | Human |
| GCF_028895645.1 | *Lactiplantibacillus plantarum* | R62 | Human |
| GCF_028895665.1 | *Lactiplantibacillus plantarum* | R58 | Human |
| GCF_028895685.1 | *Lactiplantibacillus plantarum* | R49 | Human |
| GCF_028895695.1 | *Lactiplantibacillus plantarum* | R46 | Human |
| GCF_028895705.1 | *Lactiplantibacillus plantarum* | R39 | Human |
| GCF_028895745.1 | *Lactiplantibacillus plantarum* | R35 | Human |
| GCF_028895765.1 | *Lactiplantibacillus plantarum* | R106 | Human |
| GCF_028895785.1 | *Lactiplantibacillus plantarum* | R105 | Human |
| GCF_028895795.1 | *Lactiplantibacillus plantarum* | R102 | Human |
| GCF_028895825.1 | *Lactiplantibacillus plantarum* | C7-7 | Human |
| GCF_028895845.1 | *Lactiplantibacillus plantarum* | C7-35 | Human |
| GCF_028895855.1 | *Lactiplantibacillus plantarum* | C7-39 | Human |
| GCF_028895885.1 | *Lactiplantibacillus plantarum* | C7-40 | Human |
| GCF_028895905.1 | *Lactiplantibacillus plantarum* | C7-52 | Human |
| GCF_028895925.1 | *Lactiplantibacillus plantarum* | FUA3428 | Human |
| GCF_028895945.1 | *Lactiplantibacillus plantarum* | FUA3309 | Human |
| GCF_028895965.1 | *Lactiplantibacillus plantarum* | FUA3302 | Human |
| GCF_013367715.1 | *Lactiplantibacillus plantarum* | ZW5 | Human |
| GCF_048817875.1 | *Lactiplantibacillus plantarum* | L125 | Human |
| GCF_050017215.1 | *Lactiplantibacillus plantarum* | GUANKE | Human |
| GCF_902386645.1 | *Lactiplantibacillus plantarum* | 022AE | Human |
| GCF_038743365.1 | *Lactiplantibacillus plantarum* | DY46 | Human |
| GCF_901830435.1 | *Lactiplantibacillus plantarum* | IMI 507027 | Human |
| GCF_052438955.1 | *Lactiplantibacillus plantarum* | P9 | Human |
| GCF_001596195.1 | *Lactiplantibacillus plantarum* | A8 | Human |
| GCF_000956195.1 | *Lactiplantibacillus plantarum* | W2 | Human |
| GCF_002370925.1 | *Lactiplantibacillus plantarum* | IMI 507028 | Food |
| GCF_009889735.1 | *Lactiplantibacillus plantarum* | ST | Food |
| GCF_009889825.1 | *Lactiplantibacillus plantarum* | T30PCM17 | Food |
| GCF_009889865.1 | *Lactiplantibacillus plantarum* | T30PCM31 | Food |
| GCF_009913615.1 | *Lactiplantibacillus plantarum* | T30PCM2 | Human |
| GCF_009913635.1 | *Lactiplantibacillus plantarum* | IMI 507026 | Human |
| GCF_009913655.1 | *Lactiplantibacillus plantarum* | HY41 | Human |
| GCF_040822455.1 | *Lactiplantibacillus plantarum* | SCB0151 | Human |
| GCF_029590535.1 | *Lactiplantibacillus plantarum* | SCB223 | Human |
| GCF_041222805.1 | *Lactiplantibacillus plantarum* | Lb2 | Animal |
| GCF_029997055.1 | *Lactiplantibacillus plantarum* | FCa3L | Animal |
| GCF_049990875.1 | *Lactiplantibacillus plantarum* | LOC1 | Animal |
| GCF_041531555.1 | *Lactiplantibacillus plantarum* | D444 | Animal |
| GCF_001639565.1 | *Lactiplantibacillus plantarum* | LM14-2 | Human |
| GCF_041941405.1 | *Lactiplantibacillus plantarum* | LP-F1 | Human |
| GCF_025189685.1 | *Lactiplantibacillus plantarum* | MNCW_1 | Human |
| GCF_001981655.1 | *Lactiplantibacillus plantarum* | VHProbi V38 | Human |
| GCF_049524395.1 | *Lactiplantibacillus plantarum* | LPC904 | Food |
| GCF_049524635.1 | *Lactiplantibacillus plantarum* | LPIMC513 | Food |
| GCF_049524735.1 | *Lactiplantibacillus plantarum* | LPT52 | Food |
| GCF_049524755.1 | *Lactiplantibacillus plantarum* | KUGBRC | Food |
| GCF_049524795.1 | *Lactiplantibacillus plantarum* | JB-1 | Food |
| GCF_049524855.1 | *Lactiplantibacillus plantarum* | PC1-1 | Food |
| GCF_049524875.1 | *Lactiplantibacillus plantarum* | DRD-15 | Food |
| GCF_049524915.1 | *Lactiplantibacillus plantarum* | DRD-10 | Food |
| GCF_049524975.1 | *Lactiplantibacillus plantarum* | DRD-44 | Food |
| GCF_049524995.1 | *Lactiplantibacillus plantarum* | DRD-38 | Food |
| GCF_049525035.1 | *Lactiplantibacillus plantarum* | DRD-41 | Food |
| GCF_049525095.1 | *Lactiplantibacillus plantarum* | DRD-46 | Food |
| GCF_049525115.1 | *Lactiplantibacillus plantarum* | DRD-76 | Food |
| GCF_049525155.1 | *Lactiplantibacillus plantarum* | DRD-63 | Food |
| GCF_049525215.1 | *Lactiplantibacillus plantarum* | DRD-67 | Food |
| GCF_049525235.1 | *Lactiplantibacillus plantarum* | DRD-32 | Food |
| GCF_049525335.1 | *Lactiplantibacillus plantarum* | DRD-31 | Food |
| GCF_049525355.1 | *Lactiplantibacillus plantarum* | DRD-36 | Food |
| GCF_049525375.1 | *Lactiplantibacillus plantarum* | DRD-34 | Food |
| GCF_036353295.2 | *Lactiplantibacillus plantarum* | DRD-124 | Food |
| GCF_002994725.1 | *Lactiplantibacillus plantarum* | DRD-16 | Food |
| GCF_013307305.1 | *Lactiplantibacillus plantarum* | DRD-65 | Food |
| GCF_024181705.1 | *Lactiplantibacillus plantarum* | Z.6-1 | Food |
| GCF_022832515.1 | *Lactiplantibacillus plantarum* | DM083 | Food |
| GCF_029623555.1 | *Lactiplantibacillus plantarum* | 3-?.?. | Food |
| GCF_041080595.1 | *Lactiplantibacillus plantarum* | P9 | Food |
| GCF_019308385.1 | *Lactiplantibacillus plantarum* | Lp-6 | Food |
| GCF_002116955.1 | *Lactiplantibacillus plantarum* | YD1 | Food |
| GCF_002868755.1 | *Lactiplantibacillus plantarum* | YD2 | Food |
| GCF_002868775.1 | *Lactiplantibacillus plantarum* | HOM3204 | Food |
| GCF_003269405.1 | *Lactiplantibacillus plantarum* | LpYC41 | Food |
| GCF_003286955.1 | *Lactiplantibacillus plantarum* | Q180 | Food |
| GCF_003428355.1 | *Lactiplantibacillus plantarum* | VHProbi O04 | Food |
| GCF_003577505.1 | *Lactiplantibacillus plantarum* | VHProbi O10 | Food |
| GCF_003597595.1 | *Lactiplantibacillus plantarum* | SRCM210459 | Food |
| GCF_003597615.1 | *Lactiplantibacillus plantarum* | SRCM210576 | Food |
| GCF_003597635.1 | *Lactiplantibacillus plantarum* | SRCM210465 | Food |
| GCF_003813125.1 | *Lactiplantibacillus plantarum* | SRCM210579 | Food |
| GCF_004337615.1 | *Lactiplantibacillus plantarum* | SRCM210580 | Food |
| GCF_009913695.1 | *Lactiplantibacillus plantarum* | SRCM210797 | Food |
| GCF_009935675.1 | *Lactiplantibacillus plantarum* | BIA JP8 | Food |
| GCF_012109355.1 | *Lactiplantibacillus plantarum* | 1525 | Food |
| GCF_012974545.1 | *Lactiplantibacillus plantarum* | 1526 | Food |
| GCF_014840995.1 | *Lactiplantibacillus plantarum* | 1529 | Food |
| GCF_017742875.1 | *Lactiplantibacillus plantarum* | 1527 | Food |
| GCF_019879165.1 | *Lactiplantibacillus plantarum* | HMX2 | Food |
| GCF_025245865.1 | *Lactiplantibacillus plantarum* | CIRM-BIA 2117 | Food |
| GCF_026153115.1 | *Lactiplantibacillus plantarum* | CIRM-BIA 2107 | Food |
| GCF_026976315.1 | *Lactiplantibacillus plantarum* | CIRM-BIA 2115 | Food |
| GCF_028768485.1 | *Lactiplantibacillus plantarum* | CIRM-BIA 2106 | Food |
| GCF_029543005.1 | *Lactiplantibacillus plantarum* | CIRM-BIA 2113 | Food |
| GCF_029637825.1 | *Lactiplantibacillus plantarum* | CIRM-BIA 1524 | Food |
| GCF_029910155.1 | *Lactiplantibacillus plantarum* | CIRM-BIA 2114 | Food |
| GCF_031348585.1 | *Lactiplantibacillus plantarum* | CIRM-BIA 465 | Food |
| GCF_031432955.1 | *Lactiplantibacillus plantarum* | CIRM-BIA 653 | Food |
| GCF_031461015.1 | *Lactiplantibacillus plantarum* | CIRM-BIA 466 | Food |
| GCF_031461055.1 | *Lactiplantibacillus plantarum* | CIRM-BIA 1111 | Food |
| GCF_031596315.1 | *Lactiplantibacillus plantarum* | CIRM-BIA 1108 | Food |
| GCF_031597175.1 | *Lactiplantibacillus plantarum* | CIRM-BIA 1110 | Food |
| GCF_041344935.1 | *Lactiplantibacillus plantarum* | CIRM-BIA 2109 | Food |
| GCF_049942725.1 | *Lactiplantibacillus plantarum* | CIRM-BIA 2110 | Food |
| GCF_049942745.1 | *Lactiplantibacillus plantarum* | CIRM-BIA 2184 | Food |
| GCF_052044035.1 | *Lactiplantibacillus plantarum* | CIRM-BIA 2180 | Food |
| GCF_052044045.1 | *Lactiplantibacillus plantarum* | CIRM-BIA 2182 | Food |
| GCF_052044075.1 | *Lactiplantibacillus plantarum* | CIRM-BIA 2181 | Food |
| GCF_052044095.1 | *Lactiplantibacillus plantarum* | CIRM-BIA 2185 | Food |
| GCF_052063245.1 | *Lactiplantibacillus plantarum* | CIRM-BIA 1525 | Food |
| GCF_052373675.1 | *Lactiplantibacillus plantarum* | CIRM-BIA 2224 | Food |
| GCF_052373895.1 | *Lactiplantibacillus plantarum* | CIRM-BIA 2183 | Food |
| GCF_009720585.1 | *Lactiplantibacillus plantarum* | SRCM210354 | Food |
| GCF_003325395.1 | *Lactiplantibacillus plantarum* | Y2-5 | Food |
| GCF_050920835.1 | *Lactiplantibacillus plantarum* | Y2-1 | Food |
| GCF_028994535.1 | *Lactiplantibacillus plantarum* | MGEL20154 | Environment |
| GCF_009913795.1 | *Lactiplantibacillus plantarum* | L1 | Food |
| GCF_004118615.1 | *Lactiplantibacillus plantarum* | BF_15 | Food |
| GCF_018257155.1 | *Lactiplantibacillus plantarum* | MH-301 | Animal |
| GCF_013808535.1 | *Lactiplantibacillus plantarum* | E2 | Food |
| GCF_004319665.1 | *Lactiplantibacillus plantarum* | VHProbi P06 | Human |
| GCF_046097075.1 | *Lactiplantibacillus plantarum* | LP24 | Food |
| GCF_026156855.1 | *Lactiplantibacillus plantarum* | LP23 | Food |
| GCF_026156905.1 | *Lactiplantibacillus plantarum* | FUA3073 | Others |
| GCF_004683785.1 | *Lactiplantibacillus plantarum* | FUA3247 | Food |
| GCF_009863935.1 | *Lactiplantibacillus plantarum* | FUA3309 | Food |
| GCF_001633385.1 | *Lactiplantibacillus plantarum* | LP03 | Food |
| GCF_001982035.1 | *Lactiplantibacillus plantarum* | FUA3302 | Environment |
| GCF_001982305.1 | *Lactiplantibacillus plantarum* | LP51 | Environment |
| GCF_001982325.1 | *Lactiplantibacillus plantarum* | FUA3112 | Environment |
| GCF_026275505.1 | *Lactiplantibacillus plantarum* | FUA3038 | Animal |
| GCF_001981875.1 | *Lactiplantibacillus plantarum* | FUA3428 | Food |
| GCF_001981955.1 | *Lactiplantibacillus plantarum* | LP48 | Food |
| GCF_002749875.1 | *Lactiplantibacillus plantarum* | FUA3183 | Food |
| GCF_019390095.1 | *Lactiplantibacillus plantarum* | L55 | Food |
| GCF_019399915.1 | *Lactiplantibacillus plantarum* | APC2 | Food |
| GCF_026156725.1 | *Lactiplantibacillus plantarum* | FB1 | Food |
| GCF_026156745.1 | *Lactiplantibacillus plantarum* | JS13 | Food |
| GCF_026156925.1 | *Lactiplantibacillus plantarum* | GY3 | Food |
| GCF_020916405.1 | *Lactiplantibacillus plantarum* | JY | Food |
| GCF_050685975.1 | *Lactiplantibacillus plantarum* | LC.H2.1 | Animal |
| GCF_001596095.1 | *Lactiplantibacillus plantarum* | MC2 | Food |
| GCF_047613345.1 | *Lactiplantibacillus plantarum* | PG1 | Food |
| GCF_047613425.1 | *Lactiplantibacillus plantarum* | PG2 | Food |
| GCF_050613265.1 | *Lactiplantibacillus plantarum* | QZSL | Animal |
| GCF_041297935.1 | *Lactiplantibacillus plantarum* | PG3 | Animal |
| GCF_003586485.1 | *Lactiplantibacillus plantarum* | S2.9 | Animal |
| GCF_019890755.1 | *Lactiplantibacillus plantarum* | S2.6 | Animal |
| GCF_001633685.1 | *Lactiplantibacillus plantarum* | S2.13 | Food |
| GCF_026156885.1 | *Lactiplantibacillus plantarum* | MNN1 | Food |
| GCF_047495545.1 | *Lactiplantibacillus plantarum* | XJSC | Food |
| GCF_047649195.1 | *Lactiplantibacillus plantarum* | YC1.2 | Food |
| GCF_036880975.1 | *Lactiplantibacillus plantarum* | YC41 | Food |
| GCF_030237865.1 | *Lactiplantibacillus plantarum* | XXS | Animal |
| GCF_030237845.1 | *Lactiplantibacillus plantarum* | BRD_L15 | Animal |
| GCF_001981585.1 | *Lactiplantibacillus plantarum* | ISO1 | Food |
| GCF_001981985.1 | *Lactiplantibacillus plantarum* | Y42 | Food |
| GCF_025122595.1 | *Lactiplantibacillus plantarum* | V1 | Food |
| GCF_025131215.1 | *Lactiplantibacillus plantarum* | SNP-2 | Food |
| GCF_003023825.1 | *Lactiplantibacillus plantarum* | HY41 | Food |
| GCF_004102845.1 | *Lactiplantibacillus plantarum* | B958 | Food |
| GCF_004212195.1 | *Lactiplantibacillus plantarum* | BC015 | Food |
| GCF_046353015.1 | *Lactiplantibacillus plantarum* | MC19 | Animal |
| GCF_046353025.1 | *Lactiplantibacillus plantarum* | KR19 | Animal |
| GCF_046353105.1 | *Lactiplantibacillus plantarum* | SRCM101587 | Animal |
| GCF_026156645.1 | *Lactiplantibacillus plantarum* | LL441 | Human |
| GCF_052064905.1 | *Lactiplantibacillus plantarum* | FLPL05 | Human |
| GCF_047277305.1 | *Lactiplantibacillus plantarum* | VHProbi E15 | Others |
| GCF_011040375.1 | *Lactiplantibacillus plantarum* | P8N200 | Animal |
| GCF_001010175.1 | *Lactiplantibacillus plantarum* | P8N400 | Others |
| GCF_001619265.1 | *Lactiplantibacillus plantarum* | P8N600 | Others |
| GCF_001619275.1 | *Lactiplantibacillus plantarum* | P8L200 | Others |
| GCF_001619295.1 | *Lactiplantibacillus plantarum* | P8N800 | Others |
| GCF_001981565.1 | *Lactiplantibacillus plantarum* | P8L400 | Others |
| GCF_001981575.1 | *Lactiplantibacillus plantarum* | P8L600 | Others |
| GCF_001981595.1 | *Lactiplantibacillus plantarum* | P8L800 | Others |
| GCF_001981645.1 | *Lactiplantibacillus plantarum* | AM100_PB1O_1D_2A | Others |
| GCF_001981665.1 | *Lactiplantibacillus plantarum* | AM100_PB1_LT_1D_14A | Others |
| GCF_001981785.1 | *Lactiplantibacillus plantarum* | AF91-04IFC-1A | Others |
| GCF_001981865.1 | *Lactiplantibacillus plantarum* | AM25-29 | Others |
| GCF_001982125.1 | *Lactiplantibacillus plantarum* | OM09-1A | Others |
| GCF_002370965.1 | *Lactiplantibacillus plantarum* | OF28-12b13 | Others |
| GCF_002750675.1 | *Lactiplantibacillus plantarum* | AM94-03pH10A | Others |
| GCF_002750695.1 | *Lactiplantibacillus plantarum* | AM93-05pH10A | Others |
| GCF_003692595.1 | *Lactiplantibacillus plantarum* | AM89-09b3TA | Others |
| GCF_004328745.1 | *Lactiplantibacillus plantarum* | HH-LP56 | Others |
| GCF_023347215.1 | *Lactiplantibacillus plantarum* | ZW59 | Others |
| GCF_026127545.1 | *Lactiplantibacillus plantarum* | P6 | Others |
| GCF_026127565.1 | *Lactiplantibacillus plantarum* | A7 | Others |
| GCF_026127665.1 | *Lactiplantibacillus plantarum* | H10 | Others |
| GCF_026127705.1 | *Lactiplantibacillus plantarum* | A1 | Others |
| GCF_026127805.1 | *Lactiplantibacillus plantarum* | A4 | Others |
| GCF_037996145.1 | *Lactiplantibacillus plantarum* | B5 | Others |
| GCF_042691645.1 | *Lactiplantibacillus plantarum* | G7 | Others |
| GCF_043951895.1 | *Lactiplantibacillus plantarum* | B5 | Others |
| GCF_043951965.1 | *Lactiplantibacillus plantarum* | YZH81 | Others |
| GCF_040271445.1 | *Lactiplantibacillus plantarum* | 92117_i1 | Animal |
| GCF_046353005.1 | *Lactiplantibacillus plantarum* | 92117_i3 | Others |
| GCF_046353035.1 | *Lactiplantibacillus plantarum* | 92122 | Others |
| GCF_046353045.1 | *Lactiplantibacillus plantarum* | MWLp-12 | Others |
| GCF_001672035.1 | *Lactiplantibacillus plantarum* | 24-?.?. | Food |
| GCF_002895245.1 | *Lactiplantibacillus plantarum* | SMB758 | Food |
| GCF_017592585.1 | *Lactiplantibacillus plantarum* | VHProbi V22 | Food |
| GCF_044473385.1 | *Lactiplantibacillus plantarum* | T01_HHH8 | Food |
| GCF_003611015.1 | *Lactiplantibacillus plantarum* | T01_DDD7 | Food |
| GCF_018138165.1 | *Lactiplantibacillus plantarum* | T01_DDD6 | Others |
| GCF_018138175.1 | *Lactiplantibacillus plantarum* | T01_HHH2 | Others |
| GCF_018138275.1 | *Lactiplantibacillus plantarum* | T01_HHH6 | Others |
| GCF_018138285.1 | *Lactiplantibacillus plantarum* | T01_FFF6 | Others |
| GCF_025144505.1 | *Lactiplantibacillus plantarum* | T01_EEE9 | Food |
| GCF_001633505.1 | *Lactiplantibacillus plantarum* | T01_GG4 | Others |
| GCF_048568895.1 | *Lactiplantibacillus plantarum* | T01_HH2 | Others |
| GCF_040883545.1 | *Lactiplantibacillus plantarum* | T01_CCC2 | Others |
| GCF_002109405.1 | *Lactiplantibacillus plantarum* | T01_BB11 | Food |
| GCF_016894405.1 | *Lactiplantibacillus plantarum* | T01_DD3 | Food |
| GCF_002407395.1 | *Lactiplantibacillus plantarum* | T01_EE9 | Food |
| GCF_013305265.1 | *Lactiplantibacillus plantarum* | T01_F6 | Food |
| GCF_003346175.1 | *Lactiplantibacillus plantarum* | T01_FF8 | Food |
| GCF_002117265.1 | *Lactiplantibacillus plantarum* | T01_CCC10 | Food |
| GCF_001704595.1 | *Lactiplantibacillus plantarum* | T01_FF7 | Food |
| GCF_048657025.1 | *Lactiplantibacillus plantarum* | T01_H6 | Food |
| GCF_048657045.1 | *Lactiplantibacillus plantarum* | T01_CC5 | Food |
| GCF_031082465.1 | *Lactiplantibacillus plantarum* | T01_NZ | Others |
| GCF_031082485.1 | *Lactiplantibacillus plantarum* | T01_JI1 | Others |
| GCF_031082605.1 | *Lactiplantibacillus plantarum* | T01_DD5 | Others |
| GCF_031082615.1 | *Lactiplantibacillus plantarum* | T01_CCC4 | Others |
| GCF_031082645.1 | *Lactiplantibacillus plantarum* | T01_F11 | Others |
| GCF_031082735.1 | *Lactiplantibacillus plantarum* | T01_DD1 | Others |
| GCF_031082555.1 | *Lactiplantibacillus plantarum* | T01_D6 | Others |
| GCF_031082575.1 | *Lactiplantibacillus plantarum* | T01_D8 | Others |
| GCF_031082635.1 | *Lactiplantibacillus plantarum* | T01_C1 | Others |
| GCF_031082525.1 | *Lactiplantibacillus plantarum* | LR-31 | Others |
| GCF_031082545.1 | *Lactiplantibacillus plantarum* | EN6 | Others |
| GCF_031082685.1 | *Lactiplantibacillus plantarum* | KF511 | Others |
| GCF_032818175.1 | *Lactiplantibacillus plantarum* | ZFM518 | Animal |
| GCF_001662895.1 | *Lactiplantibacillus plantarum* | CCHR2 | Food |
| GCF_964186915.1 | *Lactiplantibacillus plantarum* | SPC-SNU-72-1 | Others |
| GCF_964187035.1 | *Lactiplantibacillus plantarum* | ELAB25 | Others |
| GCF_029542245.1 | *Lactiplantibacillus plantarum* | AG10 | Animal |
| GCF_003352125.1 | *Lactiplantibacillus plantarum* | WLPL21 | Food |
| GCF_022713005.1 | *Lactiplantibacillus plantarum* | ZDY04 | Food |
| GCF_024442115.1 | *Lactiplantibacillus plantarum* | ELAB51Y | Food |
| GCF_026156615.1 | *Lactiplantibacillus plantarum* | 33C | Food |
| GCF_026156665.1 | *Lactiplantibacillus plantarum* | MA2 | Food |
| GCF_026156675.1 | *Lactiplantibacillus plantarum* | WLPL01 | Food |
| GCF_026156705.1 | *Lactiplantibacillus plantarum* | MY04 | Food |
| GCF_026156845.1 | *Lactiplantibacillus plantarum* | DMC-S1 | Food |
| GCF_026156915.1 | *Lactiplantibacillus plantarum* | FME91 | Food |
| GCF_026156965.1 | *Lactiplantibacillus plantarum* | ELAB66 | Food |
| GCF_026156985.1 | *Lactiplantibacillus plantarum* | HOM3201 | Food |
| GCF_034086125.1 | *Lactiplantibacillus plantarum* | GK502 | Food |
| GCF_048565615.1 | *Lactiplantibacillus plantarum* | Tw226 | Food |
| GCF_048565635.1 | *Lactiplantibacillus plantarum* | LP1812 | Food |
| GCF_049177195.1 | *Lactiplantibacillus plantarum* | C36 | Food |
| GCF_049177205.1 | *Lactiplantibacillus plantarum* | C34 | Food |
| GCF_051388055.1 | *Lactiplantibacillus plantarum* | YY-112 | Food |
| GCF_051388075.1 | *Lactiplantibacillus plantarum* | ILSF15 | Food |
| GCF_051388115.1 | *Lactiplantibacillus plantarum* | IYO2065 | Food |
| GCF_051388135.1 | *Lactiplantibacillus plantarum* | AWA2013 | Food |
| GCF_051388155.1 | *Lactiplantibacillus plantarum* | AWA2045 | Food |
| GCF_051388315.1 | *Lactiplantibacillus plantarum* | ELAB 66W | Food |
| GCF_051390795.1 | *Lactiplantibacillus plantarum* | ELAB 48C | Food |
| GCF_051390835.1 | *Lactiplantibacillus plantarum* | LM 0705 | Food |
| GCF_051390875.1 | *Lactiplantibacillus plantarum* | HC-2 | Food |
| GCF_051390915.1 | *Lactiplantibacillus plantarum* | FCQNA35M1 | Food |
| GCF_051390935.1 | *Lactiplantibacillus plantarum* | PCQZX1M2 | Food |
| GCF_051390955.1 | *Lactiplantibacillus plantarum* | VJLHD18L1 | Food |
| GCF_051390975.1 | *Lactiplantibacillus plantarum* | FCQNA34M6 | Food |
| GCF_051390995.1 | *Lactiplantibacillus plantarum* | FCQNA31M2 | Food |
| GCF_051391015.1 | *Lactiplantibacillus plantarum* | FCQNA32M4 | Food |
| GCF_051391035.1 | *Lactiplantibacillus plantarum* | FCQNA28M4 | Food |
| GCF_051391055.1 | *Lactiplantibacillus plantarum* | FCQNA27M4 | Food |
| GCF_051391095.1 | *Lactiplantibacillus plantarum* | FCQNA23M1 | Food |
| GCF_051391115.1 | *Lactiplantibacillus plantarum* | VJLHD12L4 | Food |
| GCF_051391135.1 | *Lactiplantibacillus plantarum* | PCQYC1M1 | Food |
| GCF_051391155.1 | *Lactiplantibacillus plantarum* | PCQYB1M3 | Food |
| GCF_051391175.1 | *Lactiplantibacillus plantarum* | PCQWS1M2 | Food |
| GCF_051391215.1 | *Lactiplantibacillus plantarum* | PCQLP6M2 | Food |
| GCF_051391235.1 | *Lactiplantibacillus plantarum* | PCQKX1M2 | Food |
| GCF_051391255.1 | *Lactiplantibacillus plantarum* | PCQDJ1M5 | Food |
| GCF_051391275.1 | *Lactiplantibacillus plantarum* | PCQDDK5M2 | Food |
| GCF_051391315.1 | *Lactiplantibacillus plantarum* | FSCPS6L4 | Food |
| GCF_051391335.1 | *Lactiplantibacillus plantarum* | FSCPS8L3 | Food |
| GCF_051391435.1 | *Lactiplantibacillus plantarum* | FSCPS35L5 | Food |
| GCF_051391455.1 | *Lactiplantibacillus plantarum* | CM3 | Food |
| GCF_051391475.1 | *Lactiplantibacillus plantarum* | CM4 | Food |
| GCF_051391495.1 | *Lactiplantibacillus plantarum* | CM1 | Food |
| GCF_051391535.1 | *Lactiplantibacillus plantarum* | BC299 | Food |
| GCF_051391555.1 | *Lactiplantibacillus plantarum* | 40C | Food |
| GCF_051391575.1 | *Lactiplantibacillus plantarum* | BGPKM22 | Food |
| GCF_051391595.1 | *Lactiplantibacillus plantarum* | C232 | Food |
| GCF_031583165.1 | *Lactiplantibacillus plantarum* | D444 | Food |
| GCF_025189985.1 | *Lactiplantibacillus plantarum* | E932 | Food |
| GCF_025190105.1 | *Lactiplantibacillus plantarum* | NAB1 | Food |
| GCF_026459915.1 | *Lactiplantibacillus plantarum* | HA3 | Food |
| GCF_051918085.1 | *Lactiplantibacillus plantarum* | HA9 | Food |
| GCF_032920365.1 | *Lactiplantibacillus plantarum* | ULE1599 | Food |
| GCF_009913675.1 | *Lactiplantibacillus plantarum* | ULE949 | Food |
| GCF_020552045.1 | *Lactiplantibacillus plantarum* | ULE639 | Food |
| GCF_020552065.1 | *Lactiplantibacillus plantarum* | ULE1841 | Food |
| GCF_020552075.1 | *Lactiplantibacillus plantarum* | ULE949 | Food |
| GCF_020552095.1 | *Lactiplantibacillus plantarum* | ULE1599 | Food |
| GCF_020552185.1 | *Lactiplantibacillus plantarum* | ULE639 | Food |
| GCF_020552215.1 | *Lactiplantibacillus plantarum* | PA21 | Food |
| GCF_020552235.1 | *Lactiplantibacillus plantarum* | RS20D | Food |
| GCF_020552285.1 | *Lactiplantibacillus plantarum* | BS25 | Food |
| GCF_020552325.1 | *Lactiplantibacillus plantarum* | P-8-A100-3 | Food |
| GCF_020552405.1 | *Lactiplantibacillus plantarum* | P-8-A100-2 | Food |
| GCF_020552425.1 | *Lactiplantibacillus plantarum* | P-8-A75-3 | Food |
| GCF_020552435.1 | *Lactiplantibacillus plantarum* | P-8-A75-2 | Food |
| GCF_020552445.1 | *Lactiplantibacillus plantarum* | P-8-A50-3 | Food |
| GCF_020552525.1 | *Lactiplantibacillus plantarum* | P-8-A50-2 | Food |
| GCF_020552585.1 | *Lactiplantibacillus plantarum* | P-8-A100-1 | Food |
| GCF_020552595.1 | *Lactiplantibacillus plantarum* | P-8-A25-2 | Food |
| GCF_020552625.1 | *Lactiplantibacillus plantarum* | P-8-A50-1 | Food |
| GCF_020552635.1 | *Lactiplantibacillus plantarum* | P-8-A25-3 | Food |
| GCF_003097595.1 | *Lactiplantibacillus plantarum* | P-8-A75-1 | Others |
| GCF_003258615.1 | *Lactiplantibacillus plantarum* | P-8-A0-3 | Animal |
| GCF_004730965.1 | *Lactiplantibacillus plantarum* | P-8-A0-2 | Food |
| GCF_001874125.1 | *Lactiplantibacillus plantarum* | P-8-A25-1 | Environment |
| GCF_030736815.1 | *Lactiplantibacillus plantarum* | P-8-A0-1 | Environment |
| GCF_021559675.1 | *Lactiplantibacillus plantarum* | BRD3A | Environment |
| GCF_048008025.1 | *Lactiplantibacillus plantarum* | KKP 3573 | Environment |
| GCF_048009075.1 | *Lactiplantibacillus plantarum* | KCKM 0597 | Environment |
| GCF_045348015.1 | *Lactiplantibacillus plantarum* | KCKM 0112 | Others |
| GCF_001273585.1 | *Lactiplantibacillus plantarum* | KCKM 0594 | Animal |
| GCF_001660645.1 | *Lactiplantibacillus plantarum* | KCKM 0106 | Animal |
| GCF_001633325.1 | *Lactiplantibacillus plantarum* | JGS49 | Food |
| GCF_001633335.1 | *Lactiplantibacillus plantarum* | VHProbi SY | Food |
| GCF_001633355.1 | *Lactiplantibacillus plantarum* | VHProbi QSH01 | Food |
| GCF_001633645.1 | *Lactiplantibacillus plantarum* | SYBC-SM2 | Food |
| GCF_001639645.1 | *Lactiplantibacillus plantarum* | SYBC-SM3 | Food |
| GCF_009762745.1 | *Lactiplantibacillus plantarum* | 7.8.4 | Others |
| GCF_050896535.2 | *Lactiplantibacillus plantarum* | BBM002 | Others |
| GCF_041679785.1 | *Lactiplantibacillus plantarum* | P46 | Human |
| GCF_015377525.1 | *Lactiplantibacillus plantarum* | P47 | Others |
| GCF_000743895.1 | *Lactiplantibacillus plantarum* | IS-10506 | Animal |
| GCF_028656165.1 | *Lactiplantibacillus plantarum* | Ep-M17 | Food |
| GCF_902825385.1 | *Lactiplantibacillus plantarum* | r-JN-1 | Animal |
| GCF_001633665.1 | *Lactiplantibacillus plantarum* | MSJK0048 | Food |
| GCF_040268325.1 | *Lactiplantibacillus plantarum* | MSJK0068 | Food |
| GCF_009889775.1 | *Lactiplantibacillus plantarum* | MDBL 269 | Food |
| GCF_001639485.1 | *Lactiplantibacillus plantarum* | WH137 | Food |
| GCF_001659745.1 | *Lactiplantibacillus plantarum* | SRP140 | Animal |
| GCF_001660025.1 | *Lactiplantibacillus plantarum* | AJOP098 | Animal |
| GCF_026184455.1 | *Lactiplantibacillus plantarum* | TBP126 | Animal |
| GCF_052232885.1 | *Lactiplantibacillus plantarum* | FWG097 | Animal |
| GCF_002117245.1 | *Lactiplantibacillus plantarum* | PRS217-49 | Food |
| GCF_002117285.1 | *Lactiplantibacillus plantarum* | beLP1 | Food |
| GCF_023702985.1 | *Lactiplantibacillus plantarum* | Z3 | Animal |
| GCF_023703225.1 | *Lactiplantibacillus plantarum* | D6 | Animal |
| GCF_023348525.1 | *Lactiplantibacillus plantarum* | Z1 | Human |
| GCF_013808525.1 | *Lactiplantibacillus plantarum* | GA_C_14 | Food |
| GCF_028446625.1 | *Lactiplantibacillus plantarum* | SN21-2 | Animal |
| GCF_017351995.1 | *Lactiplantibacillus plantarum* | YTU99 | Food |
| GCF_045528025.1 | *Lactiplantibacillus plantarum* | LFN10 | Food |
| GCF_016838645.1 | *Lactiplantibacillus plantarum* | D27 | Food |
| GCF_034555115.1 | *Lactiplantibacillus plantarum* | cqf-43 | Others |
| GCF_034555095.1 | *Lactiplantibacillus plantarum* | J50 | Animal |
| GCF_001296095.1 | *Lactiplantibacillus plantarum* | WT12 | Food |
| GCF_001633605.1 | *Lactiplantibacillus plantarum* | BF12 | Food |
| GCF_023145715.1 | *Lactiplantibacillus plantarum* | R22 | Food |
| GCF_034929385.1 | *Lactiplantibacillus plantarum* | CYLB101 | Food |
| GCF_039414055.1 | *Lactiplantibacillus plantarum* | CYLB561 | Food |
| GCF_025189945.1 | *Lactiplantibacillus plantarum* | NCHBL-004 | Food |
| GCF_026127585.1 | *Lactiplantibacillus plantarum* | ZX028 | Food |
| GCF_036431865.1 | *Lactiplantibacillus plantarum* | VHProbi M25 | Environment |
| GCF_038039495.1 | *Lactiplantibacillus plantarum* | CUL66B | Environment |
| GCF_038321065.1 | *Lactiplantibacillus plantarum* | CUL66A | Food |
| GCF_049919585.1 | *Lactiplantibacillus plantarum* | Lap | Food |
| GCF_019311695.1 | *Lactiplantibacillus plantarum* | CGMCC 14177 | Food |
| GCF_001617525.2 | *Lactiplantibacillus plantarum* | CGMCC 15358 | Food |
| GCF_016415605.1 | *Lactiplantibacillus plantarum* | F53 | Food |
| GCF_001639585.1 | *Lactiplantibacillus plantarum* | L3 | Environment |
| GCF_001639595.1 | *Lactiplantibacillus plantarum* | IMAUJBP3 | Environment |
| GCF_001639625.1 | *Lactiplantibacillus plantarum* | LAMZ-Sda | Environment |
| GCF_008868495.1 | *Lactiplantibacillus plantarum* | Lp20 | Environment |
| GCF_025189905.1 | *Lactiplantibacillus plantarum* | 2359 | Environment |
| GCF_025194145.1 | *Lactiplantibacillus plantarum* | Lrld-22 | Environment |
| GCF_026156805.1 | *Lactiplantibacillus plantarum* | DS1989 | Environment |
| GCF_046036855.1 | *Lactiplantibacillus plantarum* | DS1902 | Environment |
| GCF_050563485.1 | *Lactiplantibacillus plantarum* | IMB19 | Environment |
| GCF_040822595.1 | *Lactiplantibacillus plantarum* | A1440 | Environment |
| GCF_045159745.1 | *Lactiplantibacillus plantarum* | CUDS1902 | Environment |
| GCF_017963505.1 | *Lactiplantibacillus plantarum* | CUDS1073 | Food |
| GCF_017963525.1 | *Lactiplantibacillus plantarum* | DS1073 | Food |
| GCF_017963535.1 | *Lactiplantibacillus plantarum* | TMW 1.617 | Food |
| GCF_001639445.1 | *Lactiplantibacillus plantarum* | TMW 1.64 | Food |
| GCF_024181685.1 | *Lactiplantibacillus plantarum* | D1 | Food |
| GCF_003046075.1 | *Lactiplantibacillus plantarum* | ATA-LTC-Lp230198 | Food |
| GCF_001982005.1 | *Lactiplantibacillus plantarum* | SBS-CC-2127 | Food |
| GCF_019599425.1 | *Lactiplantibacillus plantarum* | IF | Food |
| GCF_020844665.1 | *Lactiplantibacillus plantarum* | L3 | Food |
| GCF_026127765.1 | *Lactiplantibacillus plantarum* | GBCC_F0227 | Food |
| GCF_026127775.1 | *Lactiplantibacillus plantarum* | LP140 | Food |
| GCF_037546205.1 | *Lactiplantibacillus plantarum* | plantII | Food |
| GCF_037546345.1 | *Lactiplantibacillus plantarum* | L75 | Food |
| GCF_020400695.1 | *Lactiplantibacillus plantarum* | TRA56 | Food |
| GCF_025189965.1 | *Lactiplantibacillus plantarum* | CS3 | Food |
| GCF_017581025.1 | *Lactiplantibacillus plantarum* | HOM2217 | Others |
| GCF_026127745.1 | *Lactiplantibacillus plantarum* | KA9MA | Food |
| GCF_019321805.1 | *Lactiplantibacillus plantarum* | Z33 | Animal |
| GCF_009913975.1 | *Lactiplantibacillus plantarum* | P05 | Food |
| GCF_001633765.1 | *Lactiplantibacillus plantarum* | A188 | Human |
| GCF_009864015.1 | *Lactiplantibacillus plantarum* | Z22 | Food |
| GCF_025190025.1 | *Lactiplantibacillus plantarum* | Z55 | Others |
| GCF_025212155.1 | *Lactiplantibacillus plantarum* | Z24 | Others |
| GCF_016812075.1 | *Lactiplantibacillus plantarum* | DMDL 9010 | Food |
| GCF_050847075.1 | *Lactiplantibacillus plantarum* | MKMB01 | Animal |
| GCF_026183415.1 | *Lactiplantibacillus plantarum* | C3 | Animal |
| GCF_001633805.1 | *Lactiplantibacillus plantarum* | CF-7 | Human |
| GCF_001639425.1 | *Lactiplantibacillus plantarum* | C6 | Human |
| GCF_001639505.1 | *Lactiplantibacillus plantarum* | MKMB02 | Human |
| GCF_015547405.1 | *Lactiplantibacillus plantarum* | LS 4-4 | Human |
| GCF_015549145.1 | *Lactiplantibacillus plantarum* | AHQ-14 | Human |
| GCF_015556355.1 | *Lactiplantibacillus plantarum* | BGI-N6 | Human |
| GCF_015558185.1 | *Lactiplantibacillus plantarum* | O24 | Human |
| GCF_015559875.1 | *Lactiplantibacillus plantarum* | APC2688 | Human |
| GCF_015561225.1 | *Lactiplantibacillus plantarum* | TCI837 | Human |
| GCF_030263185.2 | *Lactiplantibacillus plantarum* | SCZH-7 | Human |
| GCF_001597605.1 | *Lactiplantibacillus plantarum* | FNZ042 | Human |
| GCF_031082705.1 | *Lactiplantibacillus plantarum* | GX17 | Others |
| GCF_031082715.1 | *Lactiplantibacillus plantarum* | Z45 | Others |
| GCF_031082765.1 | *Lactiplantibacillus plantarum* | ZY-1 | Others |
| GCF_025190005.1 | *Lactiplantibacillus plantarum* | SPS109 | Others |
| GCF_039566535.1 | *Lactiplantibacillus plantarum* | LMG 13556 | Food |
| GCF_045866315.1 | *Lactiplantibacillus plantarum* | C0502 | Food |
| GCF_002165715.1 | *Lactiplantibacillus plantarum* | GMNL-661 | Environment |
| GCF_001633245.1 | *Lactiplantibacillus plantarum* | CMTB-TF1 | Animal |
| GCF_023348385.1 | *Lactiplantibacillus plantarum* | gbc_d | Human |
| GCF_022558425.1 | *Lactiplantibacillus plantarum* | gbc_C | Food |
| GCF_001540925.1 | *Lactiplantibacillus plantarum* | AUSA002 | Animal |
| GCF_001540965.1 | *Lactiplantibacillus plantarum* | AUSA004 | Animal |
| GCF_046244405.1 | *Lactiplantibacillus plantarum* | BR2-12 | Food |
| GCF_025723165.1 | *Lactiplantibacillus plantarum* | ATA-LPC98052 | Human |
| GCF_052409245.1 | *Lactiplantibacillus plantarum* | AM-LP-81 | Food |
| GCF_029854235.1 | *Lactiplantibacillus plantarum* | E8 | Others |
| GCF_004025165.1 | *Lactiplantibacillus plantarum* | E1 | Food |
| GCF_004028295.1 | *Lactiplantibacillus plantarum* | SL47 | Food |
| GCF_004028315.1 | *Lactiplantibacillus plantarum* | NS2301M | Food |
| GCF_004028335.1 | *Lactiplantibacillus plantarum* | 7.2.2 | Food |
| GCF_004122965.1 | *Lactiplantibacillus plantarum* | BD-LP | Food |
| GCF_004123035.1 | *Lactiplantibacillus plantarum* | PM8 | Food |
| GCF_004123095.1 | *Lactiplantibacillus plantarum* | HD02 | Food |
| GCF_020844675.1 | *Lactiplantibacillus plantarum* | MD159 | Food |
| GCF_026127635.1 | *Lactiplantibacillus plantarum* | G2Lp | Food |
| GCF_024137845.1 | *Lactiplantibacillus plantarum* | Do4_IM | Human |
| GCF_001639545.1 | *Lactiplantibacillus plantarum* | JY067 | Human |
| GCF_001754025.1 | *Lactiplantibacillus plantarum* | S10 | Food |
| GCF_006494465.1 | *Lactiplantibacillus plantarum* | GBW-LP001 | Food |
| GCF_027474465.1 | *Lactiplantibacillus plantarum* | VHProbi P32 | Food |
| GCF_015693925.1 | *Lactiplantibacillus plantarum* | LP01 | Food |
| GCF_047223445.1 | *Lactiplantibacillus plantarum* | LP1-6 | Food |
| GCF_017576965.1 | *Lactiplantibacillus plantarum* | LP3-9 | Food |
| GCF_040536935.1 | *Lactiplantibacillus plantarum* | LP3-4 | Food |
| GCF_001742965.1 | *Lactiplantibacillus plantarum* | LP3-5 | Food |
| GCF_002532115.1 | *Lactiplantibacillus plantarum* | AFY-10 | Food |
| GCF_007833595.1 | *Lactiplantibacillus plantarum* | LVS14 | Animal |
| GCF_021462365.1 | *Lactiplantibacillus plantarum* | SJTUF 61119 | Food |
| GCF_001633565.1 | *Lactiplantibacillus plantarum* | NMGL2 | Food |
| GCF_002117305.1 | *Lactiplantibacillus plantarum* | DSM 12028 | Others |
| GCF_009619495.1 | *Lactiplantibacillus plantarum* | HY705 | Others |
| GCF_002749655.1 | *Lactiplantibacillus plantarum* | BR-12 | Others |
| GCF_001639525.1 | *Lactiplantibacillus plantarum* | WHH1701 | Human |
| GCF_023369895.1 | *Lactiplantibacillus plantarum* | MRD5401 | Animal |
| GCF_964186765.1 | *Lactiplantibacillus plantarum* | NML21 | Human |
| GCF_964186805.1 | *Lactiplantibacillus plantarum* | TCI999 | Human |
| GCF_964186825.1 | *Lactiplantibacillus plantarum* | FAM 25263 | Human |
| GCF_964186835.1 | *Lactiplantibacillus plantarum* | B2 | Human |
| GCF_964186855.1 | *Lactiplantibacillus plantarum* | R10 | Human |
| GCF_964186875.1 | *Lactiplantibacillus plantarum* | SNK12 | Human |
| GCF_964186885.1 | *Lactiplantibacillus plantarum* | BALOs9 | Human |
| GCF_964186975.1 | *Lactiplantibacillus plantarum* | LP104 | Human |
| GCF_964187015.1 | *Lactiplantibacillus plantarum* | TCI227 | Human |
| GCF_964187025.1 | *Lactiplantibacillus plantarum* | LP8 | Human |
| GCF_024391065.1 | *Lactiplantibacillus plantarum* | NBC99 | Human |
| GCF_024391105.1 | *Lactiplantibacillus plantarum* | LpR3P51-ShortReadAssembly | Human |
| GCF_033024555.1 | *Lactiplantibacillus plantarum* | LpWF-ShortReadAssembly | Human |
| GCF_026622935.1 | *Lactiplantibacillus plantarum* | LpR3P51-LongReadAssembly | Food |
| GCF_030464425.1 | *Lactiplantibacillus plantarum* | LpDm48 | Food |
| GCF_030464565.1 | *Lactiplantibacillus plantarum* | LpDm13 | Food |
| GCF_001633725.1 | *Lactiplantibacillus plantarum* | LpWF-F9a | Food |
| GCF_000931425.2 | *Lactiplantibacillus plantarum* | LpWF-D11 | Food |
| GCF_002249825.1 | *Lactiplantibacillus plantarum* | LpWF-LongReadAssembly | Food |
| GCF_006364975.1 | *Lactiplantibacillus plantarum* | CVASU2 | Human |
| GCF_026127845.1 | *Lactiplantibacillus plantarum* | KM119 | Others |
| GCF_048541615.1 | *Lactiplantibacillus plantarum* | G2 | Food |
| GCF_020881935.1 | *Lactiplantibacillus plantarum* | MB685 | Food |
| GCF_009914095.1 | *Lactiplantibacillus plantarum* | LP45 | Food |
| GCF_009756965.1 | *Lactiplantibacillus plantarum* | LP06 | Food |
| GCF_030253605.1 | *Lactiplantibacillus plantarum* | ZJ316PV | Food |
| GCF_003076435.1 | *Lactiplantibacillus plantarum* | LAB39 | Animal |
| GCF_003143915.1 | *Lactiplantibacillus plantarum* | M95 | Food |
| GCF_001704315.1 | *Lactiplantibacillus plantarum* | OBM7 | Animal |
| GCF_001704335.1 | *Lactiplantibacillus plantarum* | BM10 | Animal |
| GCF_046352965.1 | *Lactiplantibacillus plantarum* | BM04 | Animal |
| GCF_000731855.1 | *Lactiplantibacillus plantarum* | V2 | Food |
| GCF_019076805.1 | *Lactiplantibacillus plantarum* | LP4 | Food |
| GCF_001633575.1 | *Lactiplantibacillus plantarum* | C2 | Food |
| GCF_032921125.1 | *Lactiplantibacillus plantarum* | ZG308 | Food |
| GCF_003999605.1 | *Lactiplantibacillus plantarum* | Y52 | Food |
| GCF_025631195.1 | *Lactiplantibacillus plantarum* | M31 | Food |
| GCF_037992995.1 | *Lactiplantibacillus plantarum* | 6Y-15 | Food |
| GCF_048654445.1 | *Lactiplantibacillus plantarum* | A13 | Food |
| GCF_051557165.1 | *Lactiplantibacillus plantarum* | RAL-4 | Food |
| GCF_027854125.1 | *Lactiplantibacillus plantarum* | CAL-6 | Food |
| GCF_003344825.1 | *Lactiplantibacillus plantarum* | N3 | Food |
| GCF_003344845.1 | *Lactiplantibacillus plantarum* | B3 | Food |
| GCF_009913835.1 | *Lactiplantibacillus plantarum* | LA3 | Food |
| GCF_009913855.1 | *Lactiplantibacillus plantarum* | BL25 | Food |
| GCF_052037465.1 | *Lactiplantibacillus plantarum* | W43 | Food |
| GCF_051943145.1 | *Lactiplantibacillus plantarum* | W32 | Animal |
| GCF_035588615.1 | *Lactiplantibacillus plantarum* | CQ01-107 | Animal |
| GCF_013458335.1 | *Lactiplantibacillus plantarum* | LPJBC5 | Animal |
| GCF_040435965.1 | *Lactiplantibacillus plantarum* | BLP3 | Animal |
| GCF_036321605.1 | *Lactiplantibacillus plantarum* | kfsvi1 | Food |
| GCF_030644445.1 | *Lactiplantibacillus plantarum* | DH24 | Animal |
| GCF_014132175.1 | *Lactiplantibacillus plantarum* | YG1-L1 | Animal |
| GCF_015694325.1 | *Lactiplantibacillus plantarum* | NG3-L4 | Animal |
| GCF_029855105.1 | *Lactiplantibacillus plantarum* | NG3-L1 | Animal |
| GCF_002290185.1 | *Lactiplantibacillus plantarum* | NG2-L3 | Animal |
| GCF_050707955.1 | *Lactiplantibacillus plantarum* | NG2-L2 | Food |
| GCF_001888525.1 | *Lactiplantibacillus plantarum* | NG2-L1 | Animal |
| GCF_026240755.1 | *Lactiplantibacillus plantarum* | KY6-L9 | Animal |
| GCF_044590115.1 | *Lactiplantibacillus plantarum* | KY6-L8 | Food |
| GCF_036898925.1 | *Lactiplantibacillus plantarum* | KY6-L7 | Others |
| GCF_037572815.1 | *Lactiplantibacillus plantarum* | KY6-L6 | Animal |
| GCF_050609385.1 | *Lactiplantibacillus plantarum* | KY6-L5 | Food |
| GCF_029906425.1 | *Lactiplantibacillus plantarum* | KY6-L3 | Food |
| GCF_024396815.1 | *Lactiplantibacillus plantarum* | KY6-L2 | Food |
| GCF_001888245.1 | *Lactiplantibacillus plantarum* | KY6-L1 | Human |
| GCF_001888565.1 | *Lactiplantibacillus plantarum* | KY4-L9 | Human |
| GCF_001888575.1 | *Lactiplantibacillus plantarum* | KY4-L8 | Human |
| GCF_001888585.1 | *Lactiplantibacillus plantarum* | KY4-L3 | Human |
| GCF_001888595.1 | *Lactiplantibacillus plantarum* | HS1-L2 | Human |
| GCF_001888645.1 | *Lactiplantibacillus plantarum* | HS1-L1 | Human |
| GCF_001888675.1 | *Lactiplantibacillus plantarum* | B5.2v | Human |
| GCF_002234395.1 | *Lactiplantibacillus plantarum* | K4 | Human |
| GCF_004354995.1 | *Lactiplantibacillus plantarum* | KU210152 | Human |
| GCF_011304595.2 | *Lactiplantibacillus plantarum* | KU15149 | Human |
| GCF_012070635.1 | *Lactiplantibacillus plantarum* | R26 | Human |
| GCF_012932405.1 | *Lactiplantibacillus plantarum* | GD2 | Human |
| GCF_015689055.1 | *Lactiplantibacillus plantarum* | ATG-V8 | Human |
| GCF_024758665.1 | *Lactiplantibacillus plantarum* | ATG-V2 | Human |
| GCF_024758745.1 | *Lactiplantibacillus plantarum* | ATG-K20 | Human |
| GCF_026016545.1 | *Lactiplantibacillus plantarum* | GZ | Human |
| GCF_027558615.1 | *Lactiplantibacillus plantarum* | 68zh | Human |
| GCF_027661015.1 | *Lactiplantibacillus plantarum* | BIM B-492D | Human |
| GCF_027661665.1 | *Lactiplantibacillus plantarum* | 25-?.?. | Human |
| GCF_027664985.1 | *Lactiplantibacillus plantarum* | ELPL27 | Human |
| GCF_027671205.1 | *Lactiplantibacillus plantarum* | A45 | Human |
| GCF_027691765.1 | *Lactiplantibacillus plantarum* | EI6 | Human |
| GCF_027693035.1 | *Lactiplantibacillus plantarum* | VHProbi O19 | Human |
| GCF_027698045.1 | *Lactiplantibacillus plantarum* | CNTA 628 | Human |
| GCF_027698165.1 | *Lactiplantibacillus plantarum* | TDM 0103 | Human |
| GCF_027698905.1 | *Lactiplantibacillus plantarum* | 90-TC-4 | Human |
| GCF_028321145.1 | *Lactiplantibacillus plantarum* | NCU0011190 | Human |
| GCF_028321905.1 | *Lactiplantibacillus plantarum* | Z43 | Human |
| GCF_028321915.1 | *Lactiplantibacillus plantarum* | BE1107-5-2 | Human |
| GCF_028322885.1 | *Lactiplantibacillus plantarum* | BE1103-6-20 | Human |
| GCF_028323095.1 | *Lactiplantibacillus plantarum* | BE1101-5-7 | Human |
| GCF_028323145.1 | *Lactiplantibacillus plantarum* | BE1101-5-41 | Human |
| GCF_028323205.1 | *Lactiplantibacillus plantarum* | BE1101-5-22 | Human |
| GCF_028463965.1 | *Lactiplantibacillus plantarum* | BE1101-5-21 | Human |
| GCF_028869445.1 | *Lactiplantibacillus plantarum* | BE1010-6-34 | Human |
| GCF_030063125.1 | *Lactiplantibacillus plantarum* | BE1010-6-24 | Human |
| GCF_036255815.1 | *Lactiplantibacillus plantarum* | BE1010-5-28 | Human |
| GCF_037099845.1 | *Lactiplantibacillus plantarum* | BE1010-5-29 | Human |
| GCF_037100195.1 | *Lactiplantibacillus plantarum* | BE1004-5-37 | Human |
| GCF_037414485.1 | *Lactiplantibacillus plantarum* | BE1004-5-36 | Human |
| GCF_039838505.1 | *Lactiplantibacillus plantarum* | BE1004-5-12 | Human |
| GCF_040084715.1 | *Lactiplantibacillus plantarum* | BE1003-8-8 | Human |
| GCF_040183155.1 | *Lactiplantibacillus plantarum* | BE1003-8-46 | Human |
| GCF_042465735.1 | *Lactiplantibacillus plantarum* | BE1003-8-17 | Human |
| GCF_046267855.1 | *Lactiplantibacillus plantarum* | BE1003-6-37 | Human |
| GCF_022810685.1 | *Lactiplantibacillus plantarum* | BE1003-6-14 | Food |
| GCF_050036925.1 | *Lactiplantibacillus plantarum* | BE1001-6-25 | Food |
| GCF_050037425.1 | *Lactiplantibacillus plantarum* | BE1001-5-20 | Food |
| GCF_026013765.1 | *Lactiplantibacillus plantarum* | BE1001-5-12 | Animal |
| GCF_018403705.1 | *Lactiplantibacillus plantarum* | BE091028-5-46 | Others |
| GCF_029854315.1 | *Lactiplantibacillus plantarum* | PL-02 | Others |
| GCF_033055375.1 | *Lactiplantibacillus plantarum* | CLP51 | Animal |
| GCF_033055385.1 | *Lactiplantibacillus plantarum* | HC64 | Animal |
| GCF_033055415.1 | *Lactiplantibacillus plantarum* | DXJSLMS1M11 | Animal |
| GCF_033055425.1 | *Lactiplantibacillus plantarum* | QS61 | Animal |
| GCF_033055465.1 | *Lactiplantibacillus plantarum* | QSCPS1L3 | Animal |
| GCF_016598735.1 | *Lactiplantibacillus plantarum* | QS6-12 | Others |
| GCF_001267905.1 | *Lactiplantibacillus plantarum* | RS4 | Others |
| GCF_001581895.1 | *Lactiplantibacillus plantarum* | s8-3 | Others |
| GCF_002220815.1 | *Lactiplantibacillus plantarum* | V-CQBB3-125-L8 | Others |
| GCF_017798305.1 | *Lactiplantibacillus plantarum* | QS612 | Others |
| GCF_900618215.1 | *Lactiplantibacillus plantarum* | X8 | Others |
| GCF_021560135.1 | *Lactiplantibacillus plantarum* | SCMSRS7 | Animal |
| GCF_020131335.1 | *Lactiplantibacillus plantarum* | rui | Environment |
| GCF_024137985.1 | *Lactiplantibacillus plantarum* | X1 | Food |
| GCF_014131735.1 | *Lactiplantibacillus plantarum* | FHNMY6M5 | Food |
| GCF_002109425.1 | *Lactiplantibacillus plantarum* | FGSZY333 | Food |
| GCF_032911485.1 | *Lactiplantibacillus plantarum* | FGSYC672 | Environment |
| GCF_011022295.1 | *Lactiplantibacillus plantarum* | FHNMY13M5 | Environment |
| GCF_025402835.1 | *Lactiplantibacillus plantarum* | DQHXNS9L9 | Environment |
| GCF_036281295.1 | *Lactiplantibacillus plantarum* | DL21 | Environment |
| GCF_036281355.1 | *Lactiplantibacillus plantarum* | FGSYC225L2 | Environment |
| GCF_050037215.1 | *Lactiplantibacillus plantarum* | DL38 | Environment |
| GCF_017301935.1 | *Lactiplantibacillus plantarum* | DJXSRYG2L1 | Animal |
| GCF_001888485.1 | *Lactiplantibacillus plantarum* | QHLJZD20L2 | Animal |
| GCF_001888495.1 | *Lactiplantibacillus plantarum* | QHLJZD19L5 | Animal |
| GCF_001888505.1 | *Lactiplantibacillus plantarum* | QHLJZD25L1 | Animal |
| GCF_016066915.1 | *Lactiplantibacillus plantarum* | QHLJZD23L2 | Food |
| GCF_004000705.1 | *Lactiplantibacillus plantarum* | QHLJZD16L2 | Environment |
| GCF_034333585.1 | *Lactiplantibacillus plantarum* | QHLJZD26L1 | Animal |
| GCF_050106605.1 | *Lactiplantibacillus plantarum* | QHLJZD4L1 | Animal |
| GCF_040745055.1 | *Lactiplantibacillus plantarum* | NMT1M1 | Animal |
| GCF_000687495.1 | *Lactiplantibacillus plantarum* | QHLJZD29L2 | Others |
| GCF_001368775.1 | *Lactiplantibacillus plantarum* | QHLJZD24L1 | Others |
| GCF_001888255.1 | *Lactiplantibacillus plantarum* | DHuNHHMY2L1 | Others |
| GCF_001888265.1 | *Lactiplantibacillus plantarum* | DHuNHHMY3L2 | Others |
| GCF_001888325.1 | *Lactiplantibacillus plantarum* | DJXSRYG1L1 | Others |
| GCF_001888335.1 | *Lactiplantibacillus plantarum* | CCFM8610 | Others |
| GCF_001888345.1 | *Lactiplantibacillus plantarum* | DHuNHHMY10L1 | Others |
| GCF_001888355.1 | *Lactiplantibacillus plantarum* | DHuNHHMY13L2 | Others |
| GCF_001888405.1 | *Lactiplantibacillus plantarum* | DHuNHHMY12L1 | Others |
| GCF_001888415.1 | *Lactiplantibacillus plantarum* | PCQYD1M3 | Others |
| GCF_001888425.1 | *Lactiplantibacillus plantarum* | PCQDJ1M5 | Others |
| GCF_001888465.1 | *Lactiplantibacillus plantarum* | PCQDDK5M2 | Others |
| GCF_001888655.1 | *Lactiplantibacillus plantarum* | JS-NJ-PK-4-G-1 | Others |
| GCF_001888665.1 | *Lactiplantibacillus plantarum* | FHNMY24M8 | Others |
| GCF_001888725.1 | *Lactiplantibacillus plantarum* | FZJHZD262 | Others |
| GCF_001888735.1 | *Lactiplantibacillus plantarum* | QHLJZD21L1 | Others |
| GCF_001888745.1 | *Lactiplantibacillus plantarum* | QHLJZD18L5 | Others |
| GCF_001888775.1 | *Lactiplantibacillus plantarum* | PCQYB1M3 | Others |
| GCF_002220175.1 | *Lactiplantibacillus plantarum* | PCQWS1M2 | Others |
| GCF_002914965.1 | *Lactiplantibacillus plantarum* | PCQLP6M2 | Others |
| GCF_003020005.1 | *Lactiplantibacillus plantarum* | PCQKX1M2 | Others |
| GCF_003966855.1 | *Lactiplantibacillus plantarum* | PCQYC1M1 | Others |
| GCF_004403045.2 | *Lactiplantibacillus plantarum* | PCQZX1M2 | Others |
| GCF_004404125.1 | *Lactiplantibacillus plantarum* | NX87 | Others |
| GCF_009807195.1 | *Lactiplantibacillus plantarum* | L47-2 | Others |
| GCF_009807205.1 | *Lactiplantibacillus plantarum* | JCM 1149 | Others |
| GCF_009807215.1 | *Lactiplantibacillus plantarum* | 111 | Others |
| GCF_010586945.1 | *Lactiplantibacillus plantarum* | Hi188 | Others |
| GCF_019311725.2 | *Lactiplantibacillus plantarum* | HL-PL05 | Others |
| GCF_020522885.1 | *Lactiplantibacillus plantarum* | IGMA4EH | Others |
| GCF_023168025.1 | *Lactiplantibacillus plantarum* | ZR79 | Others |
| GCF_029767725.1 | *Lactiplantibacillus plantarum* | DRC2402 | Others |
| GCF_029814785.1 | *Lactiplantibacillus plantarum* | DRC2401 | Others |
| GCF_029834415.1 | *Lactiplantibacillus plantarum* | DSR330 | Others |
| GCF_030297695.1 | *Lactiplantibacillus plantarum* | DRC2312 | Others |
| GCF_030297715.1 | *Lactiplantibacillus plantarum* | MY-2 | Others |
| GCF_030297735.1 | *Lactiplantibacillus plantarum* | Lp-115 | Others |
| GCF_030403425.1 | *Lactiplantibacillus plantarum* | Lp M2 | Others |
| GCF_030480505.1 | *Lactiplantibacillus plantarum* | HP-B1280 | Others |
| GCF_030480525.1 | *Lactiplantibacillus plantarum* | HQ04 | Others |
| GCF_030480565.1 | *Lactiplantibacillus plantarum* | M2-3 | Others |
| GCF_030709915.1 | *Lactiplantibacillus plantarum* | TPM 14.2 | Others |
| GCF_030709995.1 | *Lactiplantibacillus plantarum* | NFICC19 | Others |
| GCF_030710035.1 | *Lactiplantibacillus plantarum* | IM3 | Others |
| GCF_030710045.1 | *Lactiplantibacillus plantarum* | HJ4 | Others |
| GCF_030710055.1 | *Lactiplantibacillus plantarum* | BFE 5092 | Others |
| GCF_030710095.1 | *Lactiplantibacillus plantarum* | - | Others |
| GCF_030710155.1 | *Lactiplantibacillus plantarum* | Lp998 | Others |
| GCF_031032805.1 | *Lactiplantibacillus plantarum* | A6 | Others |
| GCF_032463565.1 | *Lactiplantibacillus plantarum* | CECT 8962 | Others |
| GCF_032463585.1 | *Lactiplantibacillus plantarum* | CECT 8966 | Others |
| GCF_033546835.1 | *Lactiplantibacillus plantarum* | CECT 8965 | Others |
| GCF_037113575.1 | *Lactiplantibacillus plantarum* | CECT 8963 | Others |
| GCF_037283765.1 | *Lactiplantibacillus plantarum* | NCTC1407 | Others |
| GCF_037997095.1 | *Lactiplantibacillus plantarum* | CECT 9492 | Others |
| GCF_040112745.1 | *Lactiplantibacillus plantarum* | CECT 9571 | Others |
| GCF_040785175.1 | *Lactiplantibacillus plantarum* | CECT 9434 | Others |
| GCF_040785225.1 | *Lactiplantibacillus plantarum* | CECT 9435 | Others |
| GCF_041344945.1 | *Lactiplantibacillus plantarum* | AMBR9 | Others |
| GCF_041928905.1 | *Lactiplantibacillus plantarum* | MGYG-HGUT-02386 | Others |
| GCF_044129425.1 | *Lactiplantibacillus plantarum* | CECT 9491 | Others |
| GCF_047445195.1 | *Lactiplantibacillus plantarum* | IM1393 | Others |
| GCF_050768895.1 | *Lactiplantibacillus plantarum* | IM930 | Others |
| GCF_051388175.1 | *Lactiplantibacillus plantarum* | IM1155 | Others |
| GCF_051388275.1 | *Lactiplantibacillus plantarum* | IM1130 | Others |
| GCF_051388345.1 | *Lactiplantibacillus plantarum* | IM562 | Others |
| GCF_051388375.1 | *Lactiplantibacillus plantarum* | IM1131 | Others |
| GCF_051388515.1 | *Lactiplantibacillus plantarum* | - | Others |
| GCF_051388595.1 | *Lactiplantibacillus plantarum* | - | Others |
| GCF_051391075.1 | *Lactiplantibacillus plantarum* | - | Others |
| GCF_900078525.1 | *Lactiplantibacillus plantarum* | AMBF-0252 | Others |
| GCF_900080205.1 | *Lactiplantibacillus plantarum* | AMBV-1343 | Others |
| GCF_900095055.1 | *Lactiplantibacillus plantarum* | AMBF-0262 | Others |
| GCF_900176235.1 | *Lactiplantibacillus plantarum* | AMBV-0661 | Others |
| GCF_900289155.1 | *Lactiplantibacillus plantarum* | AMBF-0234 | Others |
| GCF_900290085.1 | *Lactiplantibacillus plantarum* | AMBV-0030 | Others |
| GCF_900290125.1 | *Lactiplantibacillus plantarum* | AMBV-0637 | Others |
| GCF_900290135.1 | *Lactiplantibacillus plantarum* | AMBV-0098 | Others |
| GCF_925281465.1 | *Lactiplantibacillus plantarum* | AMBV-1010 | Others |
| GCF_925286555.1 | *Lactiplantibacillus plantarum* | AMBV-1009 | Others |
| GCF_925291875.1 | *Lactiplantibacillus plantarum* | AMBP-0424 | Others |
| GCF_925301115.1 | *Lactiplantibacillus plantarum* | AMBV-1314 | Others |
| GCF_925301295.1 | *Lactiplantibacillus plantarum* | AMBV-1176 | Others |
| GCF_925320525.1 | *Lactiplantibacillus plantarum* | AMBV-1344 | Others |
| GCF_964065245.1 | *Lactiplantibacillus plantarum* | AMBP-0214 | Others |
| GCF_964065445.1 | *Lactiplantibacillus plantarum* | AMBF-0270 | Others |
| GCF_964084915.1 | *Lactiplantibacillus plantarum* | CIRM-BIA777 | Others |
| GCF_965213325.1 | *Lactiplantibacillus plantarum* | VK-MK | Others |
| GCF_965213335.1 | *Lactiplantibacillus plantarum* | VK-6 | Others |
| GCF_000934625.1 | *Lactobacillus acidophilus* | FSI4 | Food |
| GCF_001639165.1 | *Lactobacillus acidophilus* | WG-LB-IV | Food |
| GCF_001868765.1 | *Lactobacillus acidophilus* | KLDS 1.0901 | Food |
| GCF_002224305.1 | *Lactobacillus acidophilus* | ATCC 53544 | Human |
| GCF_002286215.1 | *Lactobacillus acidophilus* | LA1 | Human |
| GCF_002406675.1 | *Lactobacillus acidophilus* | P2 | Food |
| GCF_002914945.1 | *Lactobacillus acidophilus* | BA05 | Others |
| GCF_003047065.1 | *Lactobacillus acidophilus* | DSM 20079 | Human |
| GCF_003053135.1 | *Lactobacillus acidophilus* | DS24_1 | Human |
| GCF_003053245.1 | *Lactobacillus acidophilus* | DS10_1A | Human |
| GCF_003061885.1 | *Lactobacillus acidophilus* | DS20_1 | Human |
| GCF_003061905.1 | *Lactobacillus acidophilus* | DS13_1B | Human |
| GCF_003061925.1 | *Lactobacillus acidophilus* | DS9_1A | Human |
| GCF_003061945.1 | *Lactobacillus acidophilus* | DS8_1A | Human |
| GCF_003061965.1 | *Lactobacillus acidophilus* | DS13_1A | Human |
| GCF_003061985.1 | *Lactobacillus acidophilus* | DS5_1A | Human |
| GCF_003062005.1 | *Lactobacillus acidophilus* | DS2_1A | Human |
| GCF_003062025.1 | *Lactobacillus acidophilus* | DS11_1A | Human |
| GCF_003062045.1 | *Lactobacillus acidophilus* | DS1_1A | Human |
| GCF_003641085.1 | *Lactobacillus acidophilus* | UBLA-34 | Food |
| GCF_008868625.1 | *Lactobacillus acidophilus* | BIO6307 | Human |
| GCF_013342945.1 | *Lactobacillus acidophilus* | LA-G80-111 | Human |
| GCF_013867555.1 | *Lactobacillus acidophilus* | s-4 | Others |
| GCF_013867605.1 | *Lactobacillus acidophilus* | s-13 | Others |
| GCF_017009485.1 | *Lactobacillus acidophilus* | BCRC 12255 | Food |
| GCF_017009515.1 | *Lactobacillus acidophilus* | BCRC 14065 | Others |
| GCF_017009595.1 | *Lactobacillus acidophilus* | BCRC 17008 | Human |
| GCF_017009605.1 | *Lactobacillus acidophilus* | BCRC 16099 | Food |
| GCF_017009655.1 | *Lactobacillus acidophilus* | BCRC 17481 | Human |
| GCF_017009695.1 | *Lactobacillus acidophilus* | BCRC 80064 | Human |
| GCF_017009715.1 | *Lactobacillus acidophilus* | La-5 | Others |
| GCF_017695935.1 | *Lactobacillus acidophilus* | APC2845 | Human |
| GCF_018252545.1 | *Lactobacillus acidophilus* | LA-G80 | Human |
| GCF_021229035.1 | *Lactobacillus acidophilus* | NBIMCC 8242 (180) | Food |
| GCF_022509485.1 | *Lactobacillus acidophilus* | QAULAN51 | Food |
| GCF_023093425.1 | *Lactobacillus acidophilus* | PB2021-BA04 | Animal |
| GCF_024397395.1 | *Lactobacillus acidophilus* | HN017 | Food |
| GCF_024665555.1 | *Lactobacillus acidophilus* | LA-5 | Human |
| GCF_025194685.1 | *Lactobacillus acidophilus* | CIRM-BIA 1674 | Human |
| GCF_025194705.1 | *Lactobacillus acidophilus* | CIRM-BIA 903 | Food |
| GCF_025194745.1 | *Lactobacillus acidophilus* | CIRM-BIA 902 | Food |
| GCF_025194765.1 | *Lactobacillus acidophilus* | CIRM-BIA 441 | Human |
| GCF_025194805.1 | *Lactobacillus acidophilus* | CIRM-BIA 443 | Others |
| GCF_025194825.1 | *Lactobacillus acidophilus* | CIRM-BIA 442 | Food |
| GCF_025194875.1 | *Lactobacillus acidophilus* | CIRM-BIA 446 | Food |
| GCF_025194905.1 | *Lactobacillus acidophilus* | CIRM-BIA 448 | Food |
| GCF_025495965.1 | *Lactobacillus acidophilus* | CUL21B | Human |
| GCF_027659185.1 | *Lactobacillus acidophilus* | AM104-58 | Human |
| GCF_027659195.1 | *Lactobacillus acidophilus* | AM104-61 | Human |
| GCF_027659735.1 | *Lactobacillus acidophilus* | AM102-83 | Human |
| GCF_027660125.1 | *Lactobacillus acidophilus* | AM102-112 | Human |
| GCF_027660145.1 | *Lactobacillus acidophilus* | AM102-110 | Human |
| GCF_027660185.1 | *Lactobacillus acidophilus* | AM102-109 | Human |
| GCF_027660295.1 | *Lactobacillus acidophilus* | AM102-77 | Human |
| GCF_027662945.1 | *Lactobacillus acidophilus* | AM04S-70 | Human |
| GCF_027672145.1 | *Lactobacillus acidophilus* | AM18-13 | Human |
| GCF_027672405.1 | *Lactobacillus acidophilus* | AM13-1 | Human |
| GCF_027680805.1 | *Lactobacillus acidophilus* | AF75-02b14 | Human |
| GCF_027684755.1 | *Lactobacillus acidophilus* | AF16-5LB | Human |
| GCF_027687975.1 | *Lactobacillus acidophilus* | AF05-37B | Human |
| GCF_027689875.1 | *Lactobacillus acidophilus* | TM02-12 | Human |
| GCF_027690645.1 | *Lactobacillus acidophilus* | TF07-26 | Human |
| GCF_027690935.1 | *Lactobacillus acidophilus* | TF04-11-1 | Human |
| GCF_027692675.1 | *Lactobacillus acidophilus* | OF38-15pH5 | Human |
| GCF_029334915.1 | *Lactobacillus acidophilus* | MehediL2 | Food |
| GCF_030369715.1 | *Lactobacillus acidophilus* | W626 | Food |
| GCF_030520045.1 | *Lactobacillus acidophilus* | ATCC 53544 | Animal |
| GCF_030520065.1 | *Lactobacillus acidophilus* | ATCC 9224 | Human |
| GCF_032463485.1 | *Lactobacillus acidophilus* | CICC6074 | Food |
| GCF_033569435.1 | *Lactobacillus acidophilus* | P42 | Animal |
| GCF_033598375.1 | *Lactobacillus acidophilus* | NSL | Animal |
| GCF_034298135.1 | *Lactobacillus acidophilus* | ATCC 4356 | Human |
| GCF_036347815.1 | *Lactobacillus acidophilus* | MCC 0241 | Food |
| GCF_036350535.1 | *Lactobacillus acidophilus* | MCC 0257 | Food |
| GCF_037996485.1 | *Lactobacillus acidophilus* | ATA-LTC-La060301 | Human |
| GCF_037996565.1 | *Lactobacillus acidophilus* | ATA-LAP1201 | Human |
| GCF_039880475.1 | *Lactobacillus acidophilus* | NCFM | Human |
| GCF_040785195.1 | *Lactobacillus acidophilus* | gbc_A | Others |
| GCF_040883405.1 | *Lactobacillus acidophilus* | AM-LA-19 | Others |
| GCF_040925255.1 | *Lactobacillus acidophilus* | G1 | Human |
| GCF_040925285.1 | *Lactobacillus acidophilus* | B3 | Human |
| GCF_040954475.1 | *Lactobacillus acidophilus* | NCFM_HOWARU | Human |
| GCF_045269835.1 | *Lactobacillus acidophilus* | MRD044 | Animal |
| GCF_046529775.1 | *Lactobacillus acidophilus* | C4 | Human |
| GCF_049070585.1 | *Lactobacillus acidophilus* | DS0557 | Human |
| GCF_050573395.1 | *Lactobacillus acidophilus* | WGS-8 | Animal |
| GCF_051388395.1 | *Lactobacillus acidophilus* | FFJND6L5 | Human |
| GCF_051388435.1 | *Lactobacillus acidophilus* | FFJND7L5 | Human |
| GCF_051388655.1 | *Lactobacillus acidophilus* | FCQHC4LH1 | Human |
| GCF_051388755.1 | *Lactobacillus acidophilus* | FAHWH11L56 | Human |
| GCF_051388795.1 | *Lactobacillus acidophilus* | FNMGHHHT12L40 | Human |
| GCF_051397015.1 | *Lactobacillus acidophilus* | SLAM_LAA02 | Human |
| GCF_965136345.1 | *Lactobacillus acidophilus* | CIP103601 | Others |
| GCF_001546015.1 | *Lactobacillus crispatus* | VMC1 | Human |
| GCF_001563615.1 | *Lactobacillus crispatus* | PSS7772C | Human |
| GCF_001567095.1 | *Lactobacillus crispatus* | JCM 5810 | Animal |
| GCF_001700475.1 | *Lactobacillus crispatus* | C037 | Human |
| GCF_002088015.1 | *Lactobacillus crispatus* | ATCC 33820 | Others |
| GCF_002218565.1 | *Lactobacillus crispatus* | UMNLC2 | Animal |
| GCF_002218615.1 | *Lactobacillus crispatus* | UMNLC1 | Animal |
| GCF_002218645.1 | *Lactobacillus crispatus* | UMNLC3 | Animal |
| GCF_002218655.1 | *Lactobacillus crispatus* | UMNLC4 | Animal |
| GCF_002218695.1 | *Lactobacillus crispatus* | UMNLC6 | Animal |
| GCF_002218765.1 | *Lactobacillus crispatus* | UMNLC5 | Animal |
| GCF_002218775.1 | *Lactobacillus crispatus* | UMNLC7 | Animal |
| GCF_002218805.1 | *Lactobacillus crispatus* | UMNLC10 | Animal |
| GCF_002218885.1 | *Lactobacillus crispatus* | UMNLC14 | Animal |
| GCF_002218895.1 | *Lactobacillus crispatus* | UMNLC15 | Animal |
| GCF_002218925.1 | *Lactobacillus crispatus* | UMNLC16 | Animal |
| GCF_002218965.1 | *Lactobacillus crispatus* | UMNLC19 | Animal |
| GCF_002218975.1 | *Lactobacillus crispatus* | UMNLC20 | Animal |
| GCF_002219005.1 | *Lactobacillus crispatus* | UMNLC21 | Animal |
| GCF_002219015.1 | *Lactobacillus crispatus* | UMNLC24 | Animal |
| GCF_002219045.1 | *Lactobacillus crispatus* | UMNLC22 | Animal |
| GCF_002219055.1 | *Lactobacillus crispatus* | UMNLC25 | Animal |
| GCF_002219085.1 | *Lactobacillus crispatus* | UMNLC23 | Animal |
| GCF_002861765.1 | *Lactobacillus crispatus* | UMB0803 | Human |
| GCF_002861775.1 | *Lactobacillus crispatus* | UMB0044 | Human |
| GCF_002861815.1 | *Lactobacillus crispatus* | UMB0085 | Human |
| GCF_002863485.1 | *Lactobacillus crispatus* | UMB0054 | Human |
| GCF_002863505.1 | *Lactobacillus crispatus* | UMB1398 | Human |
| GCF_003795065.1 | *Lactobacillus crispatus* | CO3MRSI1 | Human |
| GCF_003971565.1 | *Lactobacillus crispatus* | AB70 | Human |
| GCF_004334905.1 | *Lactobacillus crispatus* | CG-12 | Animal |
| GCF_004361125.1 | *Lactobacillus crispatus* | RL28 | Human |
| GCF_004361175.1 | *Lactobacillus crispatus* | RL26 | Human |
| GCF_004361185.1 | *Lactobacillus crispatus* | RL24 | Human |
| GCF_004361195.1 | *Lactobacillus crispatus* | RL21 | Human |
| GCF_004361205.1 | *Lactobacillus crispatus* | RL19 | Human |
| GCF_004361245.1 | *Lactobacillus crispatus* | RL15 | Human |
| GCF_004361295.1 | *Lactobacillus crispatus* | RL11 | Human |
| GCF_004361375.1 | *Lactobacillus crispatus* | RL02 | Human |
| GCF_004361445.1 | *Lactobacillus crispatus* | RL27 | Human |
| GCF_004361465.1 | *Lactobacillus crispatus* | RL25 | Human |
| GCF_004361515.1 | *Lactobacillus crispatus* | RL20 | Human |
| GCF_004361555.1 | *Lactobacillus crispatus* | RL14 | Human |
| GCF_004361565.1 | *Lactobacillus crispatus* | RL10 | Human |
| GCF_004361575.1 | *Lactobacillus crispatus* | RL09 | Human |
| GCF_007713895.1 | *Lactobacillus crispatus* | NCK1350 | Human |
| GCF_008694205.1 | *Lactobacillus crispatus* | NCK971 | Animal |
| GCF_008694765.1 | *Lactobacillus crispatus* | NCK978 | Animal |
| GCF_008694785.1 | *Lactobacillus crispatus* | NCK988 | Animal |
| GCF_008694845.1 | *Lactobacillus crispatus* | NCK974 | Animal |
| GCF_008694865.1 | *Lactobacillus crispatus* | NCK973 | Animal |
| GCF_008694885.1 | *Lactobacillus crispatus* | NCK972 | Animal |
| GCF_008694935.1 | *Lactobacillus crispatus* | NCK967 | Animal |
| GCF_009730275.1 | *Lactobacillus crispatus* | FDAARGOS_743 | Human |
| GCF_009769205.1 | *Lactobacillus crispatus* | DC21.1 | Animal |
| GCF_009933525.1 | *Lactobacillus crispatus* | C25 | Animal |
| GCF_012843585.1 | *Lactobacillus crispatus* | WCA-383-APC-5E | Animal |
| GCF_013456995.1 | *Lactobacillus crispatus* | B4 | Human |
| GCF_013487905.1 | *Lactobacillus crispatus* | 1D | Animal |
| GCF_013778545.1 | *Lactobacillus crispatus* | DSM 29598 | Food |
| GCF_014654865.1 | *Lactobacillus crispatus* | BC5 | Human |
| GCF_014982905.1 | *Lactobacillus crispatus* | DSM 108970 | Animal |
| GCF_015708055.1 | *Lactobacillus crispatus* | CRI17 | Human |
| GCF_015708065.1 | *Lactobacillus crispatus* | CRI10 | Human |
| GCF_015708075.1 | *Lactobacillus crispatus* | CRI8 | Human |
| GCF_016162005.1 | *Lactobacillus crispatus* | LB67 | Animal |
| GCF_016162045.1 | *Lactobacillus crispatus* | LB66 | Animal |
| GCF_016162055.1 | *Lactobacillus crispatus* | LB64 | Animal |
| GCF_016162105.1 | *Lactobacillus crispatus* | LB65 | Animal |
| GCF_016767795.1 | *Lactobacillus crispatus* | PRL2021 | Human |
| GCF_016901535.1 | *Lactobacillus crispatus* | An726 | Animal |
| GCF_018987235.1 | *Lactobacillus crispatus* | ATCC 33820 | Human |
| GCF_019537355.1 | *Lactobacillus crispatus* | UBLCp-01 | Human |
| GCF_020042125.1 | *Lactobacillus crispatus* | Lc1226 | Human |
| GCF_020042225.1 | *Lactobacillus crispatus* | Lc116 | Human |
| GCF_021278925.1 | *Lactobacillus crispatus* | lc83 | Human |
| GCF_021278945.1 | *Lactobacillus crispatus* | lc31 | Human |
| GCF_025194045.1 | *Lactobacillus crispatus* | CIRM-BIA 2233 | Others |
| GCF_025194115.1 | *Lactobacillus crispatus* | CIRM-BIA 523 | Animal |
| GCF_026740115.1 | *Lactobacillus crispatus* | M247 | Human |
| GCF_027271175.1 | *Lactobacillus crispatus* | Lcr-MH175 | Human |
| GCF_027682305.1 | *Lactobacillus crispatus* | AF54-1pH4A | Human |
| GCF_029011595.1 | *Lactobacillus crispatus* | VSI17 | Human |
| GCF_029011765.1 | *Lactobacillus crispatus* | VSI08 | Human |
| GCF_032376685.1 | *Lactobacillus crispatus* | UMB4356 | Human |
| GCF_033803605.1 | *Lactobacillus crispatus* | UMB5393 | Human |
| GCF_033803625.1 | *Lactobacillus crispatus* | UMB5409 | Human |
| GCF_033803925.1 | *Lactobacillus crispatus* | UMB6018 | Human |
| GCF_033803945.1 | *Lactobacillus crispatus* | UMB6035 | Human |
| GCF_033803985.1 | *Lactobacillus crispatus* | UMB6021 | Human |
| GCF_038001365.1 | *Lactobacillus crispatus* | LMG 11415 | Human |
| GCF_038001385.1 | *Lactobacillus crispatus* | LMG 18189 | Human |
| GCF_040428475.1 | *Lactobacillus crispatus* | JV-V01 | Human |
| GCF_040428515.1 | *Lactobacillus crispatus* | 0795_578_1_1_MRS1 | Human |
| GCF_040428525.1 | *Lactobacillus crispatus* | 0421_295_1_1_MRS1 | Human |
| GCF_040428535.1 | *Lactobacillus crispatus* | 0055_051_1_1_MRS12 | Human |
| GCF_040428575.1 | *Lactobacillus crispatus* | 0422_293_1_1_MRS10 | Human |
| GCF_040428585.1 | *Lactobacillus crispatus* | 233 | Human |
| GCF_040428635.1 | *Lactobacillus crispatus* | 241_F3_3 | Human |
| GCF_040428645.1 | *Lactobacillus crispatus* | 415_F3_2 | Human |
| GCF_040428695.1 | *Lactobacillus crispatus* | 241_F1_2 | Human |
| GCF_040428725.1 | *Lactobacillus crispatus* | 197_Q2_b2 | Human |
| GCF_040428785.1 | *Lactobacillus crispatus* | 194_Q2_3 | Human |
| GCF_040428805.1 | *Lactobacillus crispatus* | 194_F1_1 | Human |
| GCF_040428825.1 | *Lactobacillus crispatus* | 194_Q3_2 | Human |
| GCF_040701525.1 | *Lactobacillus crispatus* | 39_01 | Human |
| GCF_045271605.1 | *Lactobacillus crispatus* | MRD6003 | Human |
| GCF_047782765.1 | *Lactobacillus crispatus* | T31e | Animal |
| GCF_048541565.1 | *Lactobacillus crispatus* | M247_Siena | Human |
| GCF_050705815.1 | *Lactobacillus crispatus* | VHProbi E04 | Human |
| GCF_964656045.1 | *Lactobacillus crispatus* | S10-5-C2-2 | Others |
| GCF_965135635.1 | *Lactobacillus crispatus* | CIP103602 | Others |
| GCF_002847905.1 | *Lactobacillus delbrueckii* | UMB0003 | Human |
| GCF_009362855.1 | *Lactobacillus delbrueckii* | 328M | Food |
| GCF_009734125.1 | *Lactobacillus delbrueckii* | TS1-06 | Food |
| GCF_018967015.1 | *Lactobacillus delbrueckii* | Lb100 | Others |
| GCF_021600185.1 | *Lactobacillus delbrueckii* | ME-803 | Others |
| GCF_021600205.1 | *Lactobacillus delbrueckii* | ME-806 | Others |
| GCF_021600225.1 | *Lactobacillus delbrueckii* | ME-807 | Others |
| GCF_021600265.1 | *Lactobacillus delbrueckii* | ME-802 | Others |
| GCF_021600345.1 | *Lactobacillus delbrueckii* | ME-801 | Others |
| GCF_021600365.1 | *Lactobacillus delbrueckii* | ME-800 | Others |
| GCF_021600405.1 | *Lactobacillus delbrueckii* | ME-798 | Others |
| GCF_021600425.1 | *Lactobacillus delbrueckii* | ME-797 | Others |
| GCF_021600445.1 | *Lactobacillus delbrueckii* | ME-796 | Others |
| GCF_021600465.1 | *Lactobacillus delbrueckii* | ME-795 | Others |
| GCF_021600485.1 | *Lactobacillus delbrueckii* | ME-793 | Others |
| GCF_021600505.1 | *Lactobacillus delbrueckii* | ME-792 | Others |
| GCF_021600525.1 | *Lactobacillus delbrueckii* | ME-791 | Others |
| GCF_021600545.1 | *Lactobacillus delbrueckii* | ME-790 | Others |
| GCF_021600565.1 | *Lactobacillus delbrueckii* | ME-789 | Others |
| GCF_021600585.1 | *Lactobacillus delbrueckii* | ME-788 | Others |
| GCF_021600645.1 | *Lactobacillus delbrueckii* | ME-785 | Others |
| GCF_021600785.1 | *Lactobacillus delbrueckii* | ME-783 | Others |
| GCF_021600905.1 | *Lactobacillus delbrueckii* | ME-782 | Others |
| GCF_021601085.1 | *Lactobacillus delbrueckii* | ME-804 | Others |
| GCF_021601185.1 | *Lactobacillus delbrueckii* | ME-805 | Others |
| GCF_022509495.1 | *Lactobacillus delbrueckii* | QAULDN14 | Food |
| GCF_022509545.1 | *Lactobacillus delbrueckii* | QAULDN61 | Food |
| GCF_022642945.1 | *Lactobacillus delbrueckii* | L10 | Animal |
| GCF_025186005.1 | *Lactobacillus delbrueckii* | 862 | Food |
| GCF_025193525.1 | *Lactobacillus delbrueckii* | CIRM-BIA 865 | Environment |
| GCF_025946805.1 | *Lactobacillus delbrueckii* | sunkii | Human |
| GCF_026891255.1 | *Lactobacillus delbrueckii* | LDEL-06 | Human |
| GCF_027668465.1 | *Lactobacillus delbrueckii* | AM60-1pH4A | Human |
| GCF_027675585.1 | *Lactobacillus delbrueckii* | UN03-69 | Human |
| GCF_027681325.1 | *Lactobacillus delbrueckii* | AF61-16pH5TA | Human |
| GCF_027681365.1 | *Lactobacillus delbrueckii* | AF61-11pH5A | Human |
| GCF_027857975.1 | *Lactobacillus delbrueckii* | lbd113 | Food |
| GCF_027858015.1 | *Lactobacillus delbrueckii* | lbd72 | Food |
| GCF_027858045.1 | *Lactobacillus delbrueckii* | lbd111 | Food |
| GCF_030228765.1 | *Lactobacillus delbrueckii* | UMB10309 | Human |
| GCF_030237825.1 | *Lactobacillus delbrueckii* | C37 | Animal |
| GCF_030360925.1 | *Lactobacillus delbrueckii* | TOM.179 | Food |
| GCF_030581895.1 | *Lactobacillus delbrueckii* | DPUL-F36 | Food |
| GCF_038024995.1 | *Lactobacillus delbrueckii* | ATA-LTC-Ld090812 | Human |
| GCF_047462615.1 | *Lactobacillus delbrueckii* | WS4 | Food |
| GCF_963920275.1 | *Lactobacillus delbrueckii* | L-DE_H19bR1 | Others |
| GCF_963920285.1 | *Lactobacillus delbrueckii* | L-DE_46bR1 | Others |
| GCF_963920295.1 | *Lactobacillus delbrueckii* | L-DE_H29bR1 | Others |
| GCF_963920315.1 | *Lactobacillus delbrueckii* | L-DE_155bR2 | Others |
| GCF_963920335.1 | *Lactobacillus delbrueckii* | L-DE_H14bR1 | Others |
| GCF_963920345.1 | *Lactobacillus delbrueckii* | L-DE_Y450R1 | Others |
| GCF_963920355.1 | *Lactobacillus delbrueckii* | L-DE_JB13R1 | Others |
| GCF_963920365.1 | *Lactobacillus delbrueckii* | L-DE_H24bR3 | Others |
| GCF_963920375.1 | *Lactobacillus delbrueckii* | L-DE_149bR5 | Others |
| GCF_963920385.1 | *Lactobacillus delbrueckii* | L-DE_110bR2 | Others |
| GCF_963920395.1 | *Lactobacillus delbrueckii* | L-DE_73bR5 | Others |
| GCF_963920405.1 | *Lactobacillus delbrueckii* | L-DE_H15bR1 | Others |
| GCF_963920425.1 | *Lactobacillus delbrueckii* | L-DE_H23bR3 | Others |
| GCF_963920435.1 | *Lactobacillus delbrueckii* | L-DE_144bR1 | Others |
| GCF_963920445.1 | *Lactobacillus delbrueckii* | L-DE_H28bR3 | Others |
| GCF_001063045.1 | *Lactobacillus gasseri* | 240_LCRI | Human |
| GCF_001063065.1 | *Lactobacillus gasseri* | 249_LKEF | Human |
| GCF_001063505.1 | *Lactobacillus gasseri* | 497_LGAS | Human |
| GCF_001066235.1 | *Lactobacillus gasseri* | 770_LJOH | Human |
| GCF_001068345.1 | *Lactobacillus gasseri* | 987_LJOH | Human |
| GCF_001546525.1 | *Lactobacillus gasseri* | PSS7772D | Human |
| GCF_002003555.1 | *Lactobacillus gasseri* | AL5 | Human |
| GCF_002007185.1 | *Lactobacillus gasseri* | AL3 | Human |
| GCF_002158885.1 | *Lactobacillus gasseri* | 4M13 | Human |
| GCF_002863445.1 | *Lactobacillus gasseri* | UMB0670 | Human |
| GCF_002863455.1 | *Lactobacillus gasseri* | UMB0099 | Human |
| GCF_002884735.1 | *Lactobacillus gasseri* | UMB0056 | Human |
| GCF_002940965.1 | *Lactobacillus gasseri* | UMB0045 | Human |
| GCF_003307315.1 | *Lactobacillus gasseri* | JCM 1025 | Others |
| GCF_003315575.1 | *Lactobacillus gasseri* | JG141 | Human |
| GCF_003437055.1 | *Lactobacillus gasseri* | TF08-1 | Human |
| GCF_005844525.1 | *Lactobacillus gasseri* | D8 | Human |
| GCF_006982025.1 | *Lactobacillus gasseri* | 7135 | Others |
| GCF_007785965.2 | *Lactobacillus gasseri* | UMB1196 | Human |
| GCF_007785995.1 | *Lactobacillus gasseri* | UMB0607 | Human |
| GCF_007786195.1 | *Lactobacillus gasseri* | UMB3077 | Human |
| GCF_007826985.1 | *Lactobacillus gasseri* | UMB1399 | Human |
| GCF_008868535.1 | *Lactobacillus gasseri* | BIO6369 | Human |
| GCF_015546835.1 | *Lactobacillus gasseri* | C10 | Human |
| GCF_015550315.1 | *Lactobacillus gasseri* | D8 | Human |
| GCF_017565825.1 | *Lactobacillus gasseri* | S1 5006-2 | Human |
| GCF_017638885.1 | *Lactobacillus gasseri* | HL20 | Human |
| GCF_017840575.1 | *Lactobacillus gasseri* | HL70 | Human |
| GCF_018728605.1 | *Lactobacillus gasseri* | LM19 | Human |
| GCF_020991185.1 | *Lactobacillus gasseri* | Lg1266 | Human |
| GCF_020991205.1 | *Lactobacillus gasseri* | Lg637 | Human |
| GCF_022456925.1 | *Lactobacillus gasseri* | CM2267 | Human |
| GCF_022642475.1 | *Lactobacillus gasseri* | L5 | Animal |
| GCF_027152465.1 | *Lactobacillus gasseri* | C0054B2 | Human |
| GCF_027152605.1 | *Lactobacillus gasseri* | C0054A2 | Human |
| GCF_027152625.1 | *Lactobacillus gasseri* | C0045A4 | Human |
| GCF_027152645.1 | *Lactobacillus gasseri* | C0045A5 | Human |
| GCF_027152655.1 | *Lactobacillus gasseri* | C0054A1 | Human |
| GCF_027152685.1 | *Lactobacillus gasseri* | C0045B1 | Human |
| GCF_027152725.1 | *Lactobacillus gasseri* | C0045A3 | Human |
| GCF_027152765.1 | *Lactobacillus gasseri* | C0045A2 | Human |
| GCF_027152785.1 | *Lactobacillus gasseri* | C0045A1 | Human |
| GCF_027152865.1 | *Lactobacillus gasseri* | C0016A2 | Human |
| GCF_027152905.1 | *Lactobacillus gasseri* | C0016A1 | Human |
| GCF_027152925.1 | *Lactobacillus gasseri* | C0015B5 | Human |
| GCF_027152945.1 | *Lactobacillus gasseri* | C0015B4 | Human |
| GCF_027152965.1 | *Lactobacillus gasseri* | C0015B1 | Human |
| GCF_027152985.1 | *Lactobacillus gasseri* | C0015B2 | Human |
| GCF_027153005.1 | *Lactobacillus gasseri* | C0015A4 | Human |
| GCF_027153025.1 | *Lactobacillus gasseri* | C0015A3 | Human |
| GCF_027153045.1 | *Lactobacillus gasseri* | C0015A2 | Human |
| GCF_027153065.1 | *Lactobacillus gasseri* | C0015A1 | Human |
| GCF_027153085.1 | *Lactobacillus gasseri* | C0014A1 | Human |
| GCF_027153105.1 | *Lactobacillus gasseri* | C0012A1 | Human |
| GCF_027153125.1 | *Lactobacillus gasseri* | C0010A1 | Human |
| GCF_027153145.1 | *Lactobacillus gasseri* | C0144A5 | Human |
| GCF_027153165.1 | *Lactobacillus gasseri* | C0144A4 | Human |
| GCF_027153185.1 | *Lactobacillus gasseri* | C0144A2 | Human |
| GCF_027153195.1 | *Lactobacillus gasseri* | C0144A3 | Human |
| GCF_027153225.1 | *Lactobacillus gasseri* | C0144A1 | Human |
| GCF_027153245.1 | *Lactobacillus gasseri* | C0010A2 | Human |
| GCF_027153345.1 | *Lactobacillus gasseri* | C0072A5 | Human |
| GCF_027153405.1 | *Lactobacillus gasseri* | C0072A4 | Human |
| GCF_027154325.1 | *Lactobacillus gasseri* | C0164A5 | Human |
| GCF_027154425.1 | *Lactobacillus gasseri* | C0164A4 | Human |
| GCF_027154465.1 | *Lactobacillus gasseri* | C0164A1 | Human |
| GCF_027154475.1 | *Lactobacillus gasseri* | C0164A3 | Human |
| GCF_027154585.1 | *Lactobacillus gasseri* | C0163A1 | Human |
| GCF_027154595.1 | *Lactobacillus gasseri* | C0163A2 | Human |
| GCF_027154625.1 | *Lactobacillus gasseri* | C0046B5 | Human |
| GCF_027154665.1 | *Lactobacillus gasseri* | C0046B2 | Human |
| GCF_027155985.1 | *Lactobacillus gasseri* | C0150A4 | Human |
| GCF_027156025.1 | *Lactobacillus gasseri* | C0150A3 | Human |
| GCF_027156225.1 | *Lactobacillus gasseri* | C0141A5 | Human |
| GCF_027156345.1 | *Lactobacillus gasseri* | C0140B3 | Human |
| GCF_027156385.1 | *Lactobacillus gasseri* | C0140B2 | Human |
| GCF_027156485.1 | *Lactobacillus gasseri* | C0140A2 | Human |
| GCF_027156745.1 | *Lactobacillus gasseri* | C0131A4 | Human |
| GCF_027156785.1 | *Lactobacillus gasseri* | C0131A3 | Human |
| GCF_027156805.1 | *Lactobacillus gasseri* | C0131A2 | Human |
| GCF_027156825.1 | *Lactobacillus gasseri* | C0131A1 | Human |
| GCF_027156845.1 | *Lactobacillus gasseri* | C0130A5 | Human |
| GCF_027156905.1 | *Lactobacillus gasseri* | C0130A3 | Human |
| GCF_027156915.1 | *Lactobacillus gasseri* | C0130B1 | Human |
| GCF_027156945.1 | *Lactobacillus gasseri* | C0130A4 | Human |
| GCF_027156995.1 | *Lactobacillus gasseri* | C0130A2 | Human |
| GCF_027157085.1 | *Lactobacillus gasseri* | C0130A1 | Human |
| GCF_027157105.1 | *Lactobacillus gasseri* | C0129A3 | Human |
| GCF_027157115.1 | *Lactobacillus gasseri* | C0129A4 | Human |
| GCF_027157125.1 | *Lactobacillus gasseri* | C0129A5 | Human |
| GCF_027157165.1 | *Lactobacillus gasseri* | C0128B4 | Human |
| GCF_027157245.1 | *Lactobacillus gasseri* | C0128B1 | Human |
| GCF_027157255.1 | *Lactobacillus gasseri* | C0128B3 | Human |
| GCF_027157275.1 | *Lactobacillus gasseri* | C0128B2 | Human |
| GCF_027157295.1 | *Lactobacillus gasseri* | C0128A5 | Human |
| GCF_027157385.1 | *Lactobacillus gasseri* | C0128A4 | Human |
| GCF_027157455.1 | *Lactobacillus gasseri* | C0128A2 | Human |
| GCF_027157485.1 | *Lactobacillus gasseri* | C0128A3 | Human |
| GCF_027157605.1 | *Lactobacillus gasseri* | C0121A2 | Human |
| GCF_027158505.1 | *Lactobacillus gasseri* | C0023A5 | Human |
| GCF_027158515.1 | *Lactobacillus gasseri* | C0128A1 | Human |
| GCF_027158545.1 | *Lactobacillus gasseri* | C0023A4 | Human |
| GCF_027158585.1 | *Lactobacillus gasseri* | C0023A3 | Human |
| GCF_027158605.1 | *Lactobacillus gasseri* | C0023A2 | Human |
| GCF_027158615.1 | *Lactobacillus gasseri* | C0023A1 | Human |
| GCF_027158645.1 | *Lactobacillus gasseri* | C0127C1 | Human |
| GCF_027159105.1 | *Lactobacillus gasseri* | C0122B3 | Human |
| GCF_027159135.1 | *Lactobacillus gasseri* | C0122B2 | Human |
| GCF_027159165.1 | *Lactobacillus gasseri* | C0122B4 | Human |
| GCF_027159185.1 | *Lactobacillus gasseri* | C0122B1 | Human |
| GCF_027159195.1 | *Lactobacillus gasseri* | C0122A3 | Human |
| GCF_027159215.1 | *Lactobacillus gasseri* | C0122A5 | Human |
| GCF_027159245.1 | *Lactobacillus gasseri* | C0122A4 | Human |
| GCF_027159265.1 | *Lactobacillus gasseri* | C0122A2 | Human |
| GCF_027159285.1 | *Lactobacillus gasseri* | C0122A1 | Human |
| GCF_027159305.1 | *Lactobacillus gasseri* | C0121A3 | Human |
| GCF_027159325.1 | *Lactobacillus gasseri* | C0120A4 | Human |
| GCF_027159335.1 | *Lactobacillus gasseri* | C0121A5 | Human |
| GCF_027159365.1 | *Lactobacillus gasseri* | C0120A2 | Human |
| GCF_027159375.1 | *Lactobacillus gasseri* | C0120A3 | Human |
| GCF_027159405.1 | *Lactobacillus gasseri* | C0120A1 | Human |
| GCF_027159435.1 | *Lactobacillus gasseri* | C0018B4 | Human |
| GCF_027159545.1 | *Lactobacillus gasseri* | C0018B5 | Human |
| GCF_027159555.1 | *Lactobacillus gasseri* | C0018B2 | Human |
| GCF_027159585.1 | *Lactobacillus gasseri* | C0018B3 | Human |
| GCF_027159625.1 | *Lactobacillus gasseri* | C0018B1 | Human |
| GCF_027159665.1 | *Lactobacillus gasseri* | C0018A5 | Human |
| GCF_027159675.1 | *Lactobacillus gasseri* | C0018A4 | Human |
| GCF_027159705.1 | *Lactobacillus gasseri* | C0018A2 | Human |
| GCF_027159765.1 | *Lactobacillus gasseri* | C0018A3 | Human |
| GCF_027159825.1 | *Lactobacillus gasseri* | C0018A1 | Human |
| GCF_027159845.1 | *Lactobacillus gasseri* | C0020B5 | Human |
| GCF_027159885.1 | *Lactobacillus gasseri* | C0020B4 | Human |
| GCF_027159925.1 | *Lactobacillus gasseri* | C0020B2 | Human |
| GCF_027159945.1 | *Lactobacillus gasseri* | C0020B3 | Human |
| GCF_027160025.1 | *Lactobacillus gasseri* | C0020B1 | Human |
| GCF_027160385.1 | *Lactobacillus gasseri* | C0115B3 | Human |
| GCF_027160425.1 | *Lactobacillus gasseri* | C0115C5 | Human |
| GCF_027160455.1 | *Lactobacillus gasseri* | C0115B1 | Human |
| GCF_027160505.1 | *Lactobacillus gasseri* | C0115B2 | Human |
| GCF_027160625.1 | *Lactobacillus gasseri* | C0114C5 | Human |
| GCF_027160665.1 | *Lactobacillus gasseri* | C0114C4 | Human |
| GCF_027160685.1 | *Lactobacillus gasseri* | C0114C1 | Human |
| GCF_027160705.1 | *Lactobacillus gasseri* | C0114B5 | Human |
| GCF_027160765.1 | *Lactobacillus gasseri* | C0114B2 | Human |
| GCF_027160785.1 | *Lactobacillus gasseri* | C0114B1 | Human |
| GCF_027160905.1 | *Lactobacillus gasseri* | C0113A4 | Human |
| GCF_027160925.1 | *Lactobacillus gasseri* | C0113A3 | Human |
| GCF_027160945.1 | *Lactobacillus gasseri* | C0113A2 | Human |
| GCF_027160965.1 | *Lactobacillus gasseri* | C0112B4 | Human |
| GCF_027160985.1 | *Lactobacillus gasseri* | C0112C2 | Human |
| GCF_027160995.1 | *Lactobacillus gasseri* | C0113A1 | Human |
| GCF_027161025.1 | *Lactobacillus gasseri* | C0112B3 | Human |
| GCF_027161045.1 | *Lactobacillus gasseri* | C0112B1 | Human |
| GCF_027161065.1 | *Lactobacillus gasseri* | C0112B2 | Human |
| GCF_027584195.1 | *Lactobacillus gasseri* | C0087A1 | Human |
| GCF_027677045.1 | *Lactobacillus gasseri* | TM13-16 | Human |
| GCF_027681405.1 | *Lactobacillus gasseri* | AF60-5pH5 | Human |
| GCF_027681485.1 | *Lactobacillus gasseri* | AF59-17pH5T | Human |
| GCF_027685305.1 | *Lactobacillus gasseri* | AF13-8H | Human |
| GCF_028864285.1 | *Lactobacillus gasseri* | 100-2021-13 | Human |
| GCF_028864305.1 | *Lactobacillus gasseri* | A15-1-18 | Human |
| GCF_028872155.1 | *Lactobacillus gasseri* | LM1065 | Human |
| GCF_029010235.1 | *Lactobacillus gasseri* | VSI47 | Human |
| GCF_029011455.1 | *Lactobacillus gasseri* | VSI26 | Human |
| GCF_029011785.1 | *Lactobacillus gasseri* | VSI07 | Human |
| GCF_029011805.1 | *Lactobacillus gasseri* | VSI06 | Human |
| GCF_030215285.1 | *Lactobacillus gasseri* | UMB7762 | Human |
| GCF_030216925.1 | *Lactobacillus gasseri* | UMB5940 | Human |
| GCF_030217385.1 | *Lactobacillus gasseri* | UMB1399B | Human |
| GCF_030217485.1 | *Lactobacillus gasseri* | UMB1303B | Human |
| GCF_030217755.1 | *Lactobacillus gasseri* | UMB1196B | Human |
| GCF_030218505.1 | *Lactobacillus gasseri* | UMB0613 | Human |
| GCF_030224655.1 | *Lactobacillus gasseri* | UMB2499 | Human |
| GCF_030226605.1 | *Lactobacillus gasseri* | UMB1196C | Human |
| GCF_030704365.1 | *Lactobacillus gasseri* | IM13 | Human |
| GCF_030876865.1 | *Lactobacillus gasseri* | L7 | Human |
| GCF_032377135.1 | *Lactobacillus gasseri* | UMB1549 | Human |
| GCF_032377295.1 | *Lactobacillus gasseri* | UMB3348 | Human |
| GCF_032432735.1 | *Lactobacillus gasseri* | UMB1644 | Human |
| GCF_032432755.1 | *Lactobacillus gasseri* | UMB5890 | Human |
| GCF_032432775.1 | *Lactobacillus gasseri* | UMB1579 | Human |
| GCF_033803545.1 | *Lactobacillus gasseri* | UMB2385 | Human |
| GCF_033804425.1 | *Lactobacillus gasseri* | UMB3277 | Human |
| GCF_033804445.1 | *Lactobacillus gasseri* | UMB3275 | Human |
| GCF_033804545.1 | *Lactobacillus gasseri* | UMB3290 | Human |
| GCF_035621655.1 | *Lactobacillus gasseri* | SYP-B4209 | Others |
| GCF_037100225.1 | *Lactobacillus gasseri* | DS2831 | Human |
| GCF_037414355.1 | *Lactobacillus gasseri* | CUDS2831 | Human |
| GCF_037996545.1 | *Lactobacillus gasseri* | ATA-LTC-Lg030199 | Human |
| GCF_038149705.1 | *Lactobacillus gasseri* | 1673 | Human |
| GCF_039757835.1 | *Lactobacillus gasseri* | C10 | Human |
| GCF_039757855.1 | *Lactobacillus gasseri* | G2 | Human |
| GCF_039757915.1 | *Lactobacillus gasseri* | A2 | Human |
| GCF_040267755.1 | *Lactobacillus gasseri* | TCI943 | Human |
| GCF_040428225.1 | *Lactobacillus gasseri* | 0795_578_1_1_MRSI4 | Human |
| GCF_040428245.1 | *Lactobacillus gasseri* | 194_F1_d | Human |
| GCF_040700245.1 | *Lactobacillus gasseri* | 41_07 | Human |
| GCF_040819595.1 | *Lactobacillus gasseri* | MG4644 | Human |
| GCF_040883535.1 | *Lactobacillus gasseri* | AM-LG-29 | Others |
| GCF_043002145.1 | *Lactobacillus gasseri* | CM2267_MRS2 | Human |
| GCF_044007195.1 | *Lactobacillus gasseri* | UMB9087 | Human |
| GCF_045159885.1 | *Lactobacillus gasseri* | LGZ1029 | Human |
| GCF_045270855.1 | *Lactobacillus gasseri* | MRD5460 | Human |
| GCF_046837765.1 | *Lactobacillus gasseri* | LG-145 | Human |
| GCF_047944955.1 | *Lactobacillus gasseri* | P0993 | Human |
| GCF_047975875.1 | *Lactobacillus gasseri* | P0528 | Human |
| GCF_048889985.1 | *Lactobacillus gasseri* | K9 | Human |
| GCF_048961775.1 | *Lactobacillus gasseri* | P5359 | Human |
| GCF_049060445.1 | *Lactobacillus gasseri* | Q9001 | Human |
| GCF_049204875.1 | *Lactobacillus gasseri* | IATA122 | Human |
| GCF_049205295.1 | *Lactobacillus gasseri* | IATA081 | Human |
| GCF_050706785.1 | *Lactobacillus gasseri* | VHProbi E09 | Human |
| GCF_051107075.1 | *Lactobacillus gasseri* | MCC 0232 | Human |
| GCF_052221265.1 | *Lactobacillus gasseri* | A1_770/7 | Human |
| GCF_052221285.1 | *Lactobacillus gasseri* | S1_ID 2028 | Human |
| GCF_052221305.1 | *Lactobacillus gasseri* | B1_772/3 | Human |
| GCF_900452355.1 | *Lactobacillus gasseri* | NCTC13722 | Human |
| GCF_902167745.1 | *Lactobacillus gasseri* | SV_Bg7063 | Others |
| GCF_902386655.1 | *Lactobacillus gasseri* | MGYG-HGUT-02387 | Human |
| GCF_902399865.1 | *Lactobacillus gasseri* | MGYG-HGUT-03690 | Human |
| GCF_925297925.1 | *Lactobacillus gasseri* | IM1390 | Others |
| GCF_964656015.1 | *Lactobacillus gasseri* | S1-F-C2 | Others |
| GCF_000961015.1 | *Lactobacillus helveticus* | KLDS1.8701 | Others |
| GCF_001006025.1 | *Lactobacillus helveticus* | MB2-1 | Others |
| GCF_001308285.1 | *Lactobacillus helveticus* | CAUH18 | Food |
| GCF_001702095.1 | *Lactobacillus helveticus* | D76 | Human |
| GCF_001746265.1 | *Lactobacillus helveticus* | D75 | Human |
| GCF_001895375.1 | *Lactobacillus helveticus* | AJT | Others |
| GCF_002532085.1 | *Lactobacillus helveticus* | M92 | Food |
| GCF_002849915.1 | *Lactobacillus helveticus* | FAM8627 | Food |
| GCF_002849935.1 | *Lactobacillus helveticus* | FAM8105 | Food |
| GCF_002849955.1 | *Lactobacillus helveticus* | FAM22155 | Food |
| GCF_003203795.1 | *Lactobacillus helveticus* | FAM22076 | Food |
| GCF_003203915.1 | *Lactobacillus helveticus* | FAM23285 | Food |
| GCF_003203955.1 | *Lactobacillus helveticus* | FAM19188 | Food |
| GCF_003203985.1 | *Lactobacillus helveticus* | FAM1450 | Others |
| GCF_003204055.1 | *Lactobacillus helveticus* | FAM8106 | Food |
| GCF_003233715.1 | *Lactobacillus helveticus* | FAM21493 | Food |
| GCF_003610975.1 | *Lactobacillus helveticus* | LH99 | Food |
| GCF_003955865.1 | *Lactobacillus helveticus* | LH5 | Human |
| GCF_004114755.1 | *Lactobacillus helveticus* | IDCC3801 | Human |
| GCF_009881115.1 | *Lactobacillus helveticus* | DLBSA202 | Human |
| GCF_009901645.1 | *Lactobacillus helveticus* | DLBSA201 | Human |
| GCF_011392385.1 | *Lactobacillus helveticus* | UC1035 | Others |
| GCF_013280625.1 | *Lactobacillus helveticus* | IMAU80668 | Others |
| GCF_013280655.1 | *Lactobacillus helveticus* | IMAU80756 | Others |
| GCF_013280665.1 | *Lactobacillus helveticus* | IMAU80627 | Others |
| GCF_013280675.1 | *Lactobacillus helveticus* | IMAU80622 | Others |
| GCF_013280715.1 | *Lactobacillus helveticus* | IMAU60227 | Others |
| GCF_013280735.1 | *Lactobacillus helveticus* | IMAU60212 | Others |
| GCF_013280755.1 | *Lactobacillus helveticus* | IMAU60210 | Others |
| GCF_013280765.1 | *Lactobacillus helveticus* | IMAU60206 | Others |
| GCF_013280775.1 | *Lactobacillus helveticus* | IMAU60211 | Others |
| GCF_013280815.1 | *Lactobacillus helveticus* | XZNQ | Others |
| GCF_013280825.1 | *Lactobacillus helveticus* | IMAU60204 | Others |
| GCF_013280855.1 | *Lactobacillus helveticus* | IMAU60117 | Others |
| GCF_013280915.1 | *Lactobacillus helveticus* | IMAU50151 | Others |
| GCF_013280925.1 | *Lactobacillus helveticus* | IMAU50091 | Others |
| GCF_013280955.1 | *Lactobacillus helveticus* | IMAU50079 | Others |
| GCF_013280975.1 | *Lactobacillus helveticus* | IMAU50064 | Others |
| GCF_013280985.1 | *Lactobacillus helveticus* | IMAU50024 | Others |
| GCF_013280995.1 | *Lactobacillus helveticus* | IMAU50019 | Others |
| GCF_013281005.1 | *Lactobacillus helveticus* | IMAU50022 | Others |
| GCF_013281055.1 | *Lactobacillus helveticus* | IMAU40093 | Others |
| GCF_013281075.1 | *Lactobacillus helveticus* | IMAU50011 | Others |
| GCF_013281085.1 | *Lactobacillus helveticus* | IMAU40088 | Others |
| GCF_013281095.1 | *Lactobacillus helveticus* | IMAU50013 | Others |
| GCF_013281155.1 | *Lactobacillus helveticus* | IMAU30132 | Others |
| GCF_013281175.1 | *Lactobacillus helveticus* | IMAU30120 | Others |
| GCF_013281185.1 | *Lactobacillus helveticus* | IMAU30115 | Others |
| GCF_013281205.1 | *Lactobacillus helveticus* | IMAU30109 | Others |
| GCF_013281285.1 | *Lactobacillus helveticus* | IMAU30077 | Others |
| GCF_013281295.1 | *Lactobacillus helveticus* | IMAU30094 | Others |
| GCF_013281315.1 | *Lactobacillus helveticus* | IMAU30049 | Others |
| GCF_013281355.1 | *Lactobacillus helveticus* | IMAU30046 | Others |
| GCF_013281365.1 | *Lactobacillus helveticus* | IMAU30040 | Others |
| GCF_013281395.1 | *Lactobacillus helveticus* | IMAU30035 | Others |
| GCF_013281605.1 | *Lactobacillus helveticus* | IMAU20067 | Others |
| GCF_013281635.1 | *Lactobacillus helveticus* | IMAU20078 | Others |
| GCF_013281645.1 | *Lactobacillus helveticus* | IMAU20062 | Others |
| GCF_013281675.1 | *Lactobacillus helveticus* | IMAU10149 | Others |
| GCF_013281685.1 | *Lactobacillus helveticus* | IMAU10142 | Others |
| GCF_013281695.1 | *Lactobacillus helveticus* | IMAU20035 | Others |
| GCF_013281715.1 | *Lactobacillus helveticus* | IMAU10049 | Others |
| GCF_013281755.1 | *Lactobacillus helveticus* | IMAU10033 | Others |
| GCF_013281795.1 | *Lactobacillus helveticus* | IMAU10029 | Others |
| GCF_015709185.1 | *Lactobacillus helveticus* | TK-J7A | Food |
| GCF_018408455.1 | *Lactobacillus helveticus* | JCM1004 | Others |
| GCF_019443925.1 | *Lactobacillus helveticus* | LH2020 | Others |
| GCF_021229065.1 | *Lactobacillus helveticus* | NBIMCC 8269 (179) | Food |
| GCF_022811585.1 | *Lactobacillus helveticus* | SCB0641 | Food |
| GCF_022832545.1 | *Lactobacillus helveticus* | SCB643 | Food |
| GCF_022833865.1 | *Lactobacillus helveticus* | AHU1049 | Others |
| GCF_023614485.1 | *Lactobacillus helveticus* | BIM B-461 G | Human |
| GCF_024927945.1 | *Lactobacillus helveticus* | VHProbi Y21 | Human |
| GCF_034044615.1 | *Lactobacillus helveticus* | KF7 | Food |
| GCF_034045205.1 | *Lactobacillus helveticus* | KF6 | Food |
| GCF_034045235.1 | *Lactobacillus helveticus* | KF4 | Food |
| GCF_035284085.1 | *Lactobacillus helveticus* | KF5 | Food |
| GCF_039623715.1 | *Lactobacillus helveticus* | IMAUJBH1 | Food |
| GCF_040239425.1 | *Lactobacillus helveticus* | LTC 224 | Human |
| GCF_040239445.1 | *Lactobacillus helveticus* | LTC 226 | Environment |
| GCF_040822695.1 | *Lactobacillus helveticus* | ATA-LTC-Lh | Human |
| GCF_043842675.1 | *Lactobacillus helveticus* | Lh21462 | Food |
| GCF_043842715.1 | *Lactobacillus helveticus* | Lh11961 | Food |
| GCF_043842745.1 | *Lactobacillus helveticus* | Lh21456 | Food |
| GCF_044990645.1 | *Lactobacillus helveticus* | FNCPS 9452 | Food |
| GCF_045159805.1 | *Lactobacillus helveticus* | WHH2580 | Food |
| GCF_045270815.1 | *Lactobacillus helveticus* | MRD070 | Human |
| GCF_045348125.1 | *Lactobacillus helveticus* | 191404 | Food |
| GCF_046109915.1 | *Lactobacillus helveticus* | TCI357 | Food |
| GCF_051059265.1 | *Lactobacillus helveticus* | KM7 | Animal |
| GCF_051590725.1 | *Lactobacillus helveticus* | NDM 13 | Food |
| GCF_051995165.1 | *Lactobacillus helveticus* | AD031 | Human |
| GCF_902386585.1 | *Lactobacillus helveticus* | MGYG-HGUT-02384 | Human |
| GCF_000227195.1 | *Lactobacillus iners* | 7_1_47FAA | Human |
| GCF_002871595.1 | *Lactobacillus iners* | UMB0033 | Human |
| GCF_002884695.1 | *Lactobacillus iners* | UMB1051 | Human |
| GCF_002884705.1 | *Lactobacillus iners* | UMB0030A | Human |
| GCF_002892385.1 | *Lactobacillus iners* | KA00186 | Human |
| GCF_009556455.1 | *Lactobacillus iners* | LI335 | Human |
| GCF_009857205.1 | *Lactobacillus iners* | Indica1 | Human |
| GCF_011058695.1 | *Lactobacillus iners* | C0094A1 | Human |
| GCF_011058715.1 | *Lactobacillus iners* | C0059G1 | Human |
| GCF_011058735.1 | *Lactobacillus iners* | C0011D1 | Human |
| GCF_011058755.1 | *Lactobacillus iners* | C0210C1 | Human |
| GCF_011058775.1 | *Lactobacillus iners* | C0322A1 | Human |
| GCF_011058795.1 | *Lactobacillus iners* | C0254C1 | Human |
| GCF_019459565.1 | *Lactobacillus iners* | UBLI-01 | Human |
| GCF_022455765.1 | *Lactobacillus iners* | S859 | Human |
| GCF_022455795.1 | *Lactobacillus iners* | S761 | Human |
| GCF_022455815.1 | *Lactobacillus iners* | S847 | Human |
| GCF_022456055.1 | *Lactobacillus iners* | S716 | Human |
| GCF_022456075.1 | *Lactobacillus iners* | S862 | Human |
| GCF_022456095.1 | *Lactobacillus iners* | S850 | Human |
| GCF_022456115.1 | *Lactobacillus iners* | S861 | Human |
| GCF_022456135.1 | *Lactobacillus iners* | S855 | Human |
| GCF_022456155.1 | *Lactobacillus iners* | S197 | Human |
| GCF_022456165.1 | *Lactobacillus iners* | S849 | Human |
| GCF_022456255.1 | *Lactobacillus iners* | S286 | Human |
| GCF_022456275.1 | *Lactobacillus iners* | S287 | Human |
| GCF_022456315.1 | *Lactobacillus iners* | S183 | Human |
| GCF_022456325.1 | *Lactobacillus iners* | S801 | Human |
| GCF_022456355.1 | *Lactobacillus iners* | S803 | Human |
| GCF_022456375.1 | *Lactobacillus iners* | S802 | Human |
| GCF_022456395.1 | *Lactobacillus iners* | S800 | Human |
| GCF_022456425.1 | *Lactobacillus iners* | S798 | Human |
| GCF_027584045.1 | *Lactobacillus iners* | C0089G7 | Human |
| GCF_030211705.1 | *Lactobacillus iners* | UMB10188 | Human |
| GCF_030217015.1 | *Lactobacillus iners* | UMB5616 | Human |
| GCF_030217305.1 | *Lactobacillus iners* | UMB2676 | Human |
| GCF_030218725.1 | *Lactobacillus iners* | UMB0058 | Human |
| GCF_030224705.1 | *Lactobacillus iners* | UMB1643 | Human |
| GCF_030226355.1 | *Lactobacillus iners* | UMB6964 | Human |
| GCF_030226495.1 | *Lactobacillus iners* | UMB0734 | Human |
| GCF_033803785.1 | *Lactobacillus iners* | UMB4068 | Human |
| GCF_033803805.1 | *Lactobacillus iners* | UMB4066 | Human |
| GCF_033803825.1 | *Lactobacillus iners* | UMB4096 | Human |
| GCF_033803865.1 | *Lactobacillus iners* | UMB5013 | Human |
| GCF_033803905.1 | *Lactobacillus iners* | UMB5018 | Human |
| GCF_040428215.1 | *Lactobacillus iners* | E4_1683.1263.1_CBA2 | Human |
| GCF_040428235.1 | *Lactobacillus iners* | E5_1732.1307.1_BDMRSCQ4 | Human |
| GCF_040428265.1 | *Lactobacillus iners* | E3_1733.1310.1_CBA5 | Human |
| GCF_040428305.1 | *Lactobacillus iners* | E7_1841.1391.1_CBA1 | Human |
| GCF_040428325.1 | *Lactobacillus iners* | MRSI1 | Human |
| GCF_040428355.1 | *Lactobacillus iners* | CBA7_S775 | Human |
| GCF_040428385.1 | *Lactobacillus iners* | E5_BDMRSCQ12_S286 | Human |
| GCF_040428415.1 | *Lactobacillus iners* | JKR_P1_89_A12 | Human |
| GCF_040428425.1 | *Lactobacillus iners* | 0991_727_w8_CBAc | Human |
| GCF_040428455.1 | *Lactobacillus iners* | LEAF_3008A | Human |
| GCF_040428485.1 | *Lactobacillus iners* | LEAF_2053A-b | Human |
| GCF_040717755.1 | *Lactobacillus iners* | UMB5900 | Human |
| GCF_040717805.1 | *Lactobacillus iners* | UMB5939 | Human |
| GCF_043002695.1 | *Lactobacillus iners* | E3_1733.1310.1_CBA1 | Human |
| GCF_043003265.1 | *Lactobacillus iners* | UP11_143_D | Human |
| GCF_043003935.1 | *Lactobacillus iners* | Liners_2503_V10-0 | Human |
| GCF_043004745.1 | *Lactobacillus iners* | 0998_735_1_CBA_90B_S800 | Human |
| GCF_043005555.1 | *Lactobacillus iners* | 0809_588_1_1_MRS1 | Human |
| GCF_047273005.1 | *Lactobacillus iners* | IN55195 | Human |
| GCF_047273015.1 | *Lactobacillus iners* | IN366 | Human |
| GCF_047273055.1 | *Lactobacillus iners* | I012T4 | Human |
| GCF_051144505.1 | *Lactobacillus iners* | NVM210S01 | Human |
| GCF_051144515.1 | *Lactobacillus iners* | NVM020S14 | Human |
| GCF_051144685.1 | *Lactobacillus iners* | NVM196S05 | Human |
| GCF_051144745.1 | *Lactobacillus iners* | NVM076S08 | Human |
| GCF_051144755.1 | *Lactobacillus iners* | NVM041S27 | Human |
| GCF_051201725.1 | *Lactobacillus iners* | NVM025S01 | Human |
| GCF_902374445.1 | *Lactobacillus iners* | MGYG-HGUT-01383 | Human |
| GCF_000364185.2 | *Lactobacillus intestinalis* | ASF360 | Animal |
| GCF_004793775.1 | *Lactobacillus intestinalis* | NM61_E11 | Animal |
| GCF_024397795.1 | *Lactobacillus intestinalis* | DSM 6629 | Animal |
| GCF_041225805.1 | *Lactobacillus intestinalis* | 12-1131 | Animal |
| GCF_910574025.1 | *Lactobacillus intestinalis* | MGBC000063 | Animal |
| GCF_910574115.1 | *Lactobacillus intestinalis* | MGBC000013 | Animal |
| GCF_910574335.1 | *Lactobacillus intestinalis* | MGBC000045 | Animal |
| GCF_910575535.1 | *Lactobacillus intestinalis* | MGBC000150 | Animal |
| GCF_910576825.1 | *Lactobacillus intestinalis* | MGBC000276 | Animal |
| GCF_932750815.1 | *Lactobacillus intestinalis* | DSM107393 | Other |
| GCF_001742045.1 | *Lactobacillus jensenii* | TL2937 | Human |
| GCF_001936235.1 | *Lactobacillus jensenii* | SNUV360 | Human |
| GCF_002848045.1 | *Lactobacillus jensenii* | UMB0077 | Human |
| GCF_002863405.1 | *Lactobacillus jensenii* | UMB0007 | Human |
| GCF_007785935.1 | *Lactobacillus jensenii* | UMB8489 | Human |
| GCF_007786085.1 | *Lactobacillus jensenii* | UMB0034 | Human |
| GCF_008726325.1 | *Lactobacillus jensenii* | UMB8651 | Human |
| GCF_008726555.1 | *Lactobacillus jensenii* | UMB4685 | Human |
| GCF_008728065.1 | *Lactobacillus jensenii* | UMB4707 | Human |
| GCF_009730255.1 | *Lactobacillus jensenii* | FDAARGOS_749 | Human |
| GCF_011029225.1 | *Lactobacillus jensenii* | VA04-2AN | Human |
| GCF_011714625.1 | *Lactobacillus jensenii* | M166 | Human |
| GCF_012029675.1 | *Lactobacillus jensenii* | UMB0847 | Human |
| GCF_012029775.1 | *Lactobacillus jensenii* | UMB0836 | Human |
| GCF_012030285.1 | *Lactobacillus jensenii* | UMB7766 | Human |
| GCF_018094625.1 | *Lactobacillus jensenii* | ATCC 25258 | Others |
| GCF_019459545.1 | *Lactobacillus jensenii* | UBLJe-01 | Human |
| GCF_021494985.1 | *Lactobacillus jensenii* | UMB6889 | Human |
| GCF_021495105.1 | *Lactobacillus jensenii* | UMB6690 | Human |
| GCF_021495305.1 | *Lactobacillus jensenii* | UMB7846 | Human |
| GCF_022455835.1 | *Lactobacillus jensenii* | H11_S760_001 | Human |
| GCF_022456225.1 | *Lactobacillus jensenii* | CBA2_S235 | Human |
| GCF_022456535.1 | *Lactobacillus jensenii* | MRSIHK11_S855 | Human |
| GCF_022456675.1 | *Lactobacillus jensenii* | MRS1_S459 | Human |
| GCF_026184075.1 | *Lactobacillus jensenii* | UMB9248 | Human |
| GCF_027160725.1 | *Lactobacillus jensenii* | C0114B4 | Human |
| GCF_027160745.1 | *Lactobacillus jensenii* | C0114B3 | Human |
| GCF_027583935.1 | *Lactobacillus jensenii* | C0102D5 | Human |
| GCF_027584135.1 | *Lactobacillus jensenii* | C0089E5 | Human |
| GCF_030211725.1 | *Lactobacillus jensenii* | UMB10187 | Human |
| GCF_030217465.1 | *Lactobacillus jensenii* | UMB1307B | Human |
| GCF_030218205.1 | *Lactobacillus jensenii* | UMB1020 | Human |
| GCF_030226935.1 | *Lactobacillus jensenii* | UMB10320 | Human |
| GCF_030229985.1 | *Lactobacillus jensenii* | UMB7766-LJ435B | Human |
| GCF_032376755.1 | *Lactobacillus jensenii* | UMB5777 | Human |
| GCF_032377035.1 | *Lactobacillus jensenii* | UMB1545 | Human |
| GCF_032377315.1 | *Lactobacillus jensenii* | UMB0908 | Human |
| GCF_033803585.1 | *Lactobacillus jensenii* | UMB5669 | Human |
| GCF_033804025.1 | *Lactobacillus jensenii* | UMB7393 | Human |
| GCF_033804065.1 | *Lactobacillus jensenii* | UMB6506 | Human |
| GCF_033804085.1 | *Lactobacillus jensenii* | UMB3478 | Human |
| GCF_033804105.1 | *Lactobacillus jensenii* | UMB6491 | Human |
| GCF_033804125.1 | *Lactobacillus jensenii* | UMB3466 | Human |
| GCF_033804145.1 | *Lactobacillus jensenii* | UMB3451 | Human |
| GCF_038149725.1 | *Lactobacillus jensenii* | 1855 | Human |
| GCF_038149745.1 | *Lactobacillus jensenii* | 5069 | Human |
| GCF_040428125.1 | *Lactobacillus jensenii* | 188_Q3c | Human |
| GCF_040428165.1 | *Lactobacillus jensenii* | 188_Q2_1 | Human |
| GCF_040718035.1 | *Lactobacillus jensenii* | UMB6502B | Human |
| GCF_042997765.1 | *Lactobacillus jensenii* | 0422_293_1_1_MRS1 | Human |
| GCF_042999335.1 | *Lactobacillus jensenii* | 188_Q2_2 | Human |
| GCF_043000305.1 | *Lactobacillus jensenii* | 188_Q1_1 | Human |
| GCF_043000965.1 | *Lactobacillus jensenii* | 188_F1_4 | Human |
| GCF_043001555.1 | *Lactobacillus jensenii* | 0795_578_1_1_MRS2 | Human |
| GCF_045273875.1 | *Lactobacillus jensenii* | MRD-NHRL | Human |
| GCF_050113995.1 | *Lactobacillus jensenii* | BIO6428 | Human |
| GCF_951856875.1 | *Lactobacillus jensenii* | LJ7 | Human |
| GCF_951856975.1 | *Lactobacillus jensenii* | LJ4 | Human |
| GCF_951857025.1 | *Lactobacillus jensenii* | LJ9 | Human |
| GCF_951857035.1 | *Lactobacillus jensenii* | LJ8 | Human |
| GCF_951863445.1 | *Lactobacillus jensenii* | LJ2 | Human |
| GCF_951863455.1 | *Lactobacillus jensenii* | LJ3 | Human |
| GCF_951863495.1 | *Lactobacillus jensenii* | LJ6 | Human |
| GCF_001572665.1 | *Lactobacillus johnsonii* | W1 | Environment |
| GCF_002156645.1 | *Lactobacillus johnsonii* | LJ0 | Animal |
| GCF_003428395.1 | *Lactobacillus johnsonii* | IDCC9203 | Human |
| GCF_004011315.1 | *Lactobacillus johnsonii* | ZLJ010 | Animal |
| GCF_004569425.1 | *Lactobacillus johnsonii* | CC3 | Animal |
| GCF_004684975.1 | *Lactobacillus johnsonii* | Q1-7 | Animal |
| GCF_007876425.1 | *Lactobacillus johnsonii* | LBJ456 | Animal |
| GCF_008868555.1 | *Lactobacillus johnsonii* | BIO5467 | Food |
| GCF_009708135.1 | *Lactobacillus johnsonii* | LL8 | Animal |
| GCF_014058685.1 | *Lactobacillus johnsonii* | NCK2677 | Animal |
| GCF_014841035.1 | *Lactobacillus johnsonii* | GHZ10a | Animal |
| GCF_015235065.1 | *Lactobacillus johnsonii* | 19428wE2_CC3 | Animal |
| GCF_015560715.1 | *Lactobacillus johnsonii* | B4 | Human |
| GCF_021442345.1 | *Lactobacillus johnsonii* | CNCM I-4884 | Human |
| GCF_021464365.1 | *Lactobacillus johnsonii* | MT4 | Animal |
| GCF_022643205.1 | *Lactobacillus johnsonii* | L2 | Animal |
| GCF_023483905.1 | *Lactobacillus johnsonii* | PH-2 | Animal |
| GCF_024622485.1 | *Lactobacillus johnsonii* | DSM 100219 | Animal |
| GCF_025190845.1 | *Lactobacillus johnsonii* | CIRM-BIA 878 | Others |
| GCF_025190885.1 | *Lactobacillus johnsonii* | CIRM-BIA 650 | Others |
| GCF_025190905.1 | *Lactobacillus johnsonii* | CIRM-BIA 651 | Animal |
| GCF_025190925.1 | *Lactobacillus johnsonii* | CIRM-BIA 868 | Others |
| GCF_025190945.1 | *Lactobacillus johnsonii* | CIRM-BIA 869 | Others |
| GCF_025191025.1 | *Lactobacillus johnsonii* | CIRM-BIA 900 | Food |
| GCF_025191045.1 | *Lactobacillus johnsonii* | CIRM-BIA 871 | Food |
| GCF_025191055.1 | *Lactobacillus johnsonii* | CIRM-BIA 674 | Human |
| GCF_030237765.1 | *Lactobacillus johnsonii* | C38 | Animal |
| GCF_032376765.1 | *Lactobacillus johnsonii* | UMB3423 | Human |
| GCF_032463545.1 | *Lactobacillus johnsonii* | N7 | Animal |
| GCF_032463685.1 | *Lactobacillus johnsonii* | N5 | Animal |
| GCF_033704075.1 | *Lactobacillus johnsonii* | KD1 | Others |
| GCF_046581365.1 | *Lactobacillus johnsonii* | GJ231 | Animal |
| GCF_050847055.1 | *Lactobacillus johnsonii* | TRM 1303 | Animal |
| GCF_051063995.1 | *Lactobacillus johnsonii* | 374F | Human |
| GCF_051351065.1 | *Lactobacillus johnsonii* | HL-RH01 | Human |
| GCF_007095465.1 | *Lactobacillus mulieris* | c10Ua161M | Human |
| GCF_007786095.1 | *Lactobacillus mulieris* | UMB1355 | Human |
| GCF_008726405.1 | *Lactobacillus mulieris* | UMB8440 | Human |
| GCF_012102955.1 | *Lactobacillus mulieris* | UMB9245 | Human |
| GCF_021495045.1 | *Lactobacillus mulieris* | UMB7800 | Human |
| GCF_022454455.1 | *Lactobacillus mulieris* | DZD_CM_20_S788 | Human |
| GCF_022454635.1 | *Lactobacillus mulieris* | DZD_CM_06_S774 | Human |
| GCF_022455095.1 | *Lactobacillus mulieris* | CM_F05_S806 | Human |
| GCF_022455405.1 | *Lactobacillus mulieris* | CM_D01_S772 | Human |
| GCF_026184095.1 | *Lactobacillus mulieris* | UMB8026 | Human |
| GCF_027153865.1 | *Lactobacillus mulieris* | C0172B3 | Human |
| GCF_027153885.1 | *Lactobacillus mulieris* | C0172B4 | Human |
| GCF_027153895.1 | *Lactobacillus mulieris* | C0172B2 | Human |
| GCF_027154705.1 | *Lactobacillus mulieris* | C0161C1 | Human |
| GCF_027154965.1 | *Lactobacillus mulieris* | C0161C2 | Human |
| GCF_027155165.1 | *Lactobacillus mulieris* | C0160B4 | Human |
| GCF_027155225.1 | *Lactobacillus mulieris* | C0160B3 | Human |
| GCF_027155265.1 | *Lactobacillus mulieris* | C0159B2 | Human |
| GCF_027155305.1 | *Lactobacillus mulieris* | C0160B5 | Human |
| GCF_027155335.1 | *Lactobacillus mulieris* | C0159B1 | Human |
| GCF_027155555.1 | *Lactobacillus mulieris* | C0036B4 | Human |
| GCF_027155625.1 | *Lactobacillus mulieris* | C0036B1 | Human |
| GCF_027156005.1 | *Lactobacillus mulieris* | C0150A1 | Human |
| GCF_027156035.1 | *Lactobacillus mulieris* | C0149A5 | Human |
| GCF_027156065.1 | *Lactobacillus mulieris* | C0149A4 | Human |
| GCF_027156085.1 | *Lactobacillus mulieris* | C0149A3 | Human |
| GCF_027157445.1 | *Lactobacillus mulieris* | C0127B5 | Human |
| GCF_027158665.1 | *Lactobacillus mulieris* | C0127B3 | Human |
| GCF_027158955.1 | *Lactobacillus mulieris* | C0022B5 | Human |
| GCF_027583865.1 | *Lactobacillus mulieris* | C0109C3 | Human |
| GCF_027584015.1 | *Lactobacillus mulieris* | C0093F8 | Human |
| GCF_027584075.1 | *Lactobacillus mulieris* | C0090C3 | Human |
| GCF_027584235.1 | *Lactobacillus mulieris* | C0081E5 | Human |
| GCF_030212205.1 | *Lactobacillus mulieris* | UMB9984 | Human |
| GCF_030215185.1 | *Lactobacillus mulieris* | UMB7784 | Human |
| GCF_030218625.1 | *Lactobacillus mulieris* | UMB0047 | Human |
| GCF_032376585.1 | *Lactobacillus mulieris* | UMB3420 | Human |
| GCF_032376715.1 | *Lactobacillus mulieris* | UMB0446 | Human |
| GCF_040428115.1 | *Lactobacillus mulieris* | 241_S1_1 | Human |
| GCF_042995895.1 | *Lactobacillus mulieris* | 294_F1_4-2 | Human |
| GCF_042996645.1 | *Lactobacillus mulieris* | 294_F1_4-1 | Human |
| GCF_042997415.1 | *Lactobacillus mulieris* | CM2267_MRS1 | Human |
| GCF_902385715.1 | *Lactobacillus mulieris* | MGYG-HGUT-02313 | Human |
| GCF_003307255.1 | *Lactobacillus paragasseri* | JCM 1130 | Others |
| GCF_003307275.1 | *Lactobacillus paragasseri* | JCM 5343 | Others |
| GCF_003307295.1 | *Lactobacillus paragasseri* | JCM 5344 | Others |
| GCF_003584685.1 | *Lactobacillus paragasseri* | JCM 5343 | Others |
| GCF_007785845.1 | *Lactobacillus paragasseri* | UMB6975 | Human |
| GCF_009734365.1 | *Lactobacillus paragasseri* | NCK1347 | Human |
| GCF_010092655.1 | *Lactobacillus paragasseri* | Indica | Human |
| GCF_014269295.2 | *Lactobacillus paragasseri* | UBLG-36 | Human |
| GCF_015550525.1 | *Lactobacillus paragasseri* | 1001095IJ_161003_B4 | Human |
| GCF_015552765.1 | *Lactobacillus paragasseri* | J1101004_170508_E2 | Human |
| GCF_015560405.1 | *Lactobacillus paragasseri* | D6t1_180914_C1 | Human |
| GCF_018588245.1 | *Lactobacillus paragasseri* | ADH | Human |
| GCF_019459585.1 | *Lactobacillus paragasseri* | UBLG-36 | Human |
| GCF_019972195.1 | *Lactobacillus paragasseri* | missing | Others |
| GCF_022509305.1 | *Lactobacillus paragasseri* | QAULPN3 | Food |
| GCF_024972875.1 | *Lactobacillus paragasseri* | CRI18 | Human |
| GCF_024972895.1 | *Lactobacillus paragasseri* | CRI16 | Human |
| GCF_024972915.1 | *Lactobacillus paragasseri* | CRI22 | Human |
| GCF_025491465.1 | *Lactobacillus paragasseri* | BRTN | Animal |
| GCF_027666495.1 | *Lactobacillus paragasseri* | AM75-02pH5 | Human |
| GCF_027681345.1 | *Lactobacillus paragasseri* | AF61-17pH5T | Human |
| GCF_028882125.1 | *Lactobacillus paragasseri* | A8-1-17-13 | Human |
| GCF_028882155.1 | *Lactobacillus paragasseri* | A4-2-12 | Human |
| GCF_028882165.1 | *Lactobacillus paragasseri* | A8-1-14-17 | Human |
| GCF_029823055.1 | *Lactobacillus paragasseri* | LMG 11478 | Others |
| GCF_030215385.1 | *Lactobacillus paragasseri* | UMB6975B | Human |
| GCF_030216525.1 | *Lactobacillus paragasseri* | UMB6880 | Human |
| GCF_030216895.1 | *Lactobacillus paragasseri* | UMB6357 | Human |
| GCF_030216965.1 | *Lactobacillus paragasseri* | UMB5640 | Human |
| GCF_030217995.1 | *Lactobacillus paragasseri* | UMB1065 | Human |
| GCF_030218525.1 | *Lactobacillus paragasseri* | UMB0725 | Human |
| GCF_030225275.1 | *Lactobacillus paragasseri* | UMB0010A | Human |
| GCF_030225955.1 | *Lactobacillus paragasseri* | UMB9935 | Human |
| GCF_030226085.1 | *Lactobacillus paragasseri* | UMB9291B | Human |
| GCF_030546315.1 | *Lactobacillus paragasseri* | GP0003 | Human |
| GCF_032248535.1 | *Lactobacillus paragasseri* | BBM171 | Human |
| GCF_032376475.1 | *Lactobacillus paragasseri* | UMB3776 | Human |
| GCF_032376705.1 | *Lactobacillus paragasseri* | UMB4347 | Human |
| GCF_032377175.1 | *Lactobacillus paragasseri* | UMB1634 | Human |
| GCF_033803645.1 | *Lactobacillus paragasseri* | UMB4951 | Human |
| GCF_033803665.1 | *Lactobacillus paragasseri* | UMB4933 | Human |
| GCF_033803685.1 | *Lactobacillus paragasseri* | UMB7023 | Human |
| GCF_033803705.1 | *Lactobacillus paragasseri* | UMB6098 | Human |
| GCF_033803725.1 | *Lactobacillus paragasseri* | UMB7013 | Human |
| GCF_033803745.1 | *Lactobacillus paragasseri* | UMB6101 | Human |
| GCF_033803765.1 | *Lactobacillus paragasseri* | UMB6107 | Human |
| GCF_033804165.1 | *Lactobacillus paragasseri* | UMB4898 | Human |
| GCF_033804185.1 | *Lactobacillus paragasseri* | UMB4892 | Human |
| GCF_033804205.1 | *Lactobacillus paragasseri* | UMB4908 | Human |
| GCF_033804225.1 | *Lactobacillus paragasseri* | UMB3564 | Human |
| GCF_033804245.1 | *Lactobacillus paragasseri* | UMB3579 | Human |
| GCF_033804325.1 | *Lactobacillus paragasseri* | UMB2060 | Human |
| GCF_033804345.1 | *Lactobacillus paragasseri* | UMB1891 | Human |
| GCF_033804365.1 | *Lactobacillus paragasseri* | UMB2049 | Human |
| GCF_033804385.1 | *Lactobacillus paragasseri* | UMB2014 | Human |
| GCF_033804405.1 | *Lactobacillus paragasseri* | UMB2003 | Human |
| GCF_035621675.1 | *Lactobacillus paragasseri* | SYP-B4209 | Food |
| GCF_039751075.1 | *Lactobacillus paragasseri* | H1 | Human |
| GCF_039751115.1 | *Lactobacillus paragasseri* | E7 | Human |
| GCF_039751135.1 | *Lactobacillus paragasseri* | A10 | Human |
| GCF_039751195.1 | *Lactobacillus paragasseri* | E10 | Human |
| GCF_049191415.1 | *Lactobacillus paragasseri* | IATA108 | Human |
| GCF_049191805.1 | *Lactobacillus paragasseri* | IATA141 | Human |
| GCF_049204835.1 | *Lactobacillus paragasseri* | IATA126 | Human |
| GCF_052221085.1 | *Lactobacillus paragasseri* | R3_770/9 | Human |
| GCF_052221105.1 | *Lactobacillus paragasseri* | J1_ID 2029 | Human |
| GCF_900636895.1 | *Lactobacillus paragasseri* | NCTC13720 | Human |
| GCF_925299355.1 | *Lactobacillus paragasseri* | IM914 | Others |
| GCF_964656005.1 | *Lactobacillus paragasseri* | S3-11-C17 | Others |
| GCF_964656055.1 | *Lactobacillus paragasseri* | S3-6-C10 | Others |
| GCF_965137695.1 | *Lactobacillus paragasseri* | CIP62.18 | Others |
| GCF_002253025.1 | *Lactobacillus taiwanensis* | 609u | Animal |
| GCF_002253045.1 | *Lactobacillus taiwanensis* | 609r | Animal |
| GCF_002253065.1 | *Lactobacillus taiwanensis* | 609q | Animal |
| GCF_002253075.1 | *Lactobacillus taiwanensis* | 609h | Animal |
| GCF_002253095.1 | *Lactobacillus taiwanensis* | 601y | Animal |
| GCF_002253105.1 | *Lactobacillus taiwanensis* | 601c | Animal |
| GCF_002253145.1 | *Lactobacillus taiwanensis* | 601b | Animal |
| GCF_002253195.1 | *Lactobacillus taiwanensis* | 601a | Animal |
| GCF_002253325.1 | *Lactobacillus taiwanensis* | 114f | Animal |
| GCF_002253345.1 | *Lactobacillus taiwanensis* | 111w | Animal |
| GCF_002253365.1 | *Lactobacillus taiwanensis* | 114e | Animal |
| GCF_002253375.1 | *Lactobacillus taiwanensis* | 111u | Animal |
| GCF_002253385.1 | *Lactobacillus taiwanensis* | 111z | Animal |
| GCF_002253395.1 | *Lactobacillus taiwanensis* | 111o | Animal |
| GCF_002253445.1 | *Lactobacillus taiwanensis* | 111m | Animal |
| GCF_002253455.1 | *Lactobacillus taiwanensis* | 111k | Animal |
| GCF_002253465.1 | *Lactobacillus taiwanensis* | 107v | Animal |
| GCF_002253475.1 | *Lactobacillus taiwanensis* | 107u | Animal |
| GCF_002253525.1 | *Lactobacillus taiwanensis* | 107q | Animal |
| GCF_002253535.1 | *Lactobacillus taiwanensis* | 107d | Animal |
| GCF_002253565.1 | *Lactobacillus taiwanensis* | 103n | Animal |
| GCF_002253575.1 | *Lactobacillus taiwanensis* | 103q | Animal |
| GCF_002253605.1 | *Lactobacillus taiwanensis* | 103a | Animal |
| GCF_002253615.1 | *Lactobacillus taiwanensis* | 103j | Animal |
| GCF_009663675.1 | *Lactobacillus taiwanensis* | 321-5 | Animal |
| GCF_017894345.1 | *Lactobacillus taiwanensis* | CLG01 | Animal |
| GCF_024622435.1 | *Lactobacillus taiwanensis* | DSM 28674 | Animal |
| GCF_024622445.1 | *Lactobacillus taiwanensis* | DSM 100220 | Animal |
| GCF_041225845.1 | *Lactobacillus taiwanensis* | 12-1144 | Animal |
| GCF_001663835.1 | *Latilactobacillus curvatus* | FBA2 | Food |
| GCF_001698165.1 | *Latilactobacillus curvatus* | WiKim52 | Food |
| GCF_001723545.1 | *Latilactobacillus curvatus* | WiKim38 | Food |
| GCF_001981905.1 | *Latilactobacillus curvatus* | RI-406 | Others |
| GCF_001981925.1 | *Latilactobacillus curvatus* | RI-198 | Others |
| GCF_001982045.1 | *Latilactobacillus curvatus* | RI-193 | Others |
| GCF_002224425.1 | *Latilactobacillus curvatus* | MRS6 | Food |
| GCF_003254785.1 | *Latilactobacillus curvatus* | ZJUNIT8 | Food |
| GCF_003410375.1 | *Latilactobacillus curvatus* | TMW 1.1928 | Food |
| GCF_003957415.1 | *Latilactobacillus curvatus* | IRG2 | Human |
| GCF_004088235.1 | *Latilactobacillus curvatus* | SRCM103465 | Food |
| GCF_005049195.1 | *Latilactobacillus curvatus* | S46 | Animal |
| GCF_006540285.1 | *Latilactobacillus curvatus* | NBRC 15884 | Others |
| GCF_007954645.1 | *Latilactobacillus curvatus* | CBA3617 | Food |
| GCF_009683015.1 | *Latilactobacillus curvatus* | VRA_2sq_f | Animal |
| GCF_009683145.1 | *Latilactobacillus curvatus* | VRA_2sq_n | Animal |
| GCF_018732225.1 | *Latilactobacillus curvatus* | SPC-SNU 70-3 | Food |
| GCF_019704515.1 | *Latilactobacillus curvatus* | WDN19 | Others |
| GCF_023656945.1 | *Latilactobacillus curvatus* | HFS9 | Human |
| GCF_023743295.1 | *Latilactobacillus curvatus* | DRD-164 | Food |
| GCF_023744385.1 | *Latilactobacillus curvatus* | DRD-170 | Food |
| GCF_023744395.1 | *Latilactobacillus curvatus* | DRD-171 | Food |
| GCF_024172305.1 | *Latilactobacillus curvatus* | TMW 1.1381 | Others |
| GCF_024172325.1 | *Latilactobacillus curvatus* | TMW 1.1390 | Others |
| GCF_024172625.1 | *Latilactobacillus curvatus* | TMW 1.421 | Food |
| GCF_024172665.1 | *Latilactobacillus curvatus* | TMW 1.595 | Others |
| GCF_024272745.1 | *Latilactobacillus curvatus* | ELA204093 | Animal |
| GCF_024272755.1 | *Latilactobacillus curvatus* | ELA204098 | Animal |
| GCF_024272805.1 | *Latilactobacillus curvatus* | ELA214388 | Animal |
| GCF_024272815.1 | *Latilactobacillus curvatus* | ELA204100 | Animal |
| GCF_024272845.1 | *Latilactobacillus curvatus* | ELA204096 | Animal |
| GCF_024272865.1 | *Latilactobacillus curvatus* | ELA214062 | Animal |
| GCF_024272885.1 | *Latilactobacillus curvatus* | ELA214061 | Animal |
| GCF_024272895.1 | *Latilactobacillus curvatus* | ELA204092 | Animal |
| GCF_024272925.1 | *Latilactobacillus curvatus* | ELA214060 | Animal |
| GCF_024272945.1 | *Latilactobacillus curvatus* | ELA204033 | Animal |
| GCF_024272965.1 | *Latilactobacillus curvatus* | ELA214059 | Animal |
| GCF_024272985.1 | *Latilactobacillus curvatus* | ELA204029 | Animal |
| GCF_024273005.1 | *Latilactobacillus curvatus* | ELA204023 | Animal |
| GCF_024273015.1 | *Latilactobacillus curvatus* | ELA214002 | Animal |
| GCF_024273055.1 | *Latilactobacillus curvatus* | ELA214117 | Animal |
| GCF_024999845.1 | *Latilactobacillus curvatus* | CIRM-BIA 1443 | Environment |
| GCF_025190625.1 | *Latilactobacillus curvatus* | CIRM-BIA 1458 | Food |
| GCF_025193945.1 | *Latilactobacillus curvatus* | CIRM-BIA 1635 | Food |
| GCF_025194065.1 | *Latilactobacillus curvatus* | CIRM-BIA 468 | Food |
| GCF_027942455.1 | *Latilactobacillus curvatus* | SRCM217410 | Food |
| GCF_028622855.1 | *Latilactobacillus curvatus* | curvatus | Animal |
| GCF_029581635.1 | *Latilactobacillus curvatus* | TMW 1.2272 | Others |
| GCF_029581675.1 | *Latilactobacillus curvatus* | TMW 1.1365 | Others |
| GCF_029581685.1 | *Latilactobacillus curvatus* | TMW 1.2270 | Food |
| GCF_029581695.1 | *Latilactobacillus curvatus* | TMW 1.1408 | Food |
| GCF_029581735.1 | *Latilactobacillus curvatus* | TMW 1.706 | Others |
| GCF_029581755.1 | *Latilactobacillus curvatus* | TMW 1.591 | Others |
| GCF_030062375.1 | *Latilactobacillus curvatus* | ZHA1 | Animal |
| GCF_030144365.1 | *Latilactobacillus curvatus* | KFRI-K200347 | Food |
| GCF_032190815.1 | *Latilactobacillus curvatus* | 3.8.43 | Food |
| GCF_035621435.1 | *Latilactobacillus curvatus* | K285 | Food |
| GCF_045527965.1 | *Latilactobacillus curvatus* | FAM 25314 | Food |
| GCF_045528005.1 | *Latilactobacillus curvatus* | FAM 24637 | Food |
| GCF_049544915.1 | *Latilactobacillus curvatus* | DRC2305 | Food |
| GCF_049943105.1 | *Latilactobacillus curvatus* | KU15003 | Food |
| GCF_900178545.1 | *Latilactobacillus curvatus* | MFP1 | Food |
| GCF_902362325.1 | *Latilactobacillus curvatus* | MGYG-HGUT-00020 | Human |
| GCF_964063355.1 | *Latilactobacillus curvatus* | - | Others |
| GCF_964063385.1 | *Latilactobacillus curvatus* | - | Others |
| GCF_965118335.1 | *Latilactobacillus curvatus* | SF1574 | Food |
| GCF_965118885.1 | *Latilactobacillus curvatus* | SF766 | Food |
| GCF_001981735.1 | *Latilactobacillus sakei* | RI-403 | Others |
| GCF_001981825.1 | *Latilactobacillus sakei* | RI-404 | Others |
| GCF_002953655.1 | *Latilactobacillus sakei* | DS4 | Food |
| GCF_003288235.1 | *Latilactobacillus sakei* | WiKim0074 | Food |
| GCF_021405435.1 | *Latilactobacillus sakei* | JD10 | Animal |
| GCF_022559185.1 | *Latilactobacillus sakei* | C21B | Food |
| GCF_022559205.1 | *Latilactobacillus sakei* | E23B | Food |
| GCF_030144745.1 | *Latilactobacillus sakei* | HEM 224 | Food |
| GCF_037283805.1 | *Latilactobacillus sakei* | A1426 | Others |
| GCF_040909445.1 | *Latilactobacillus sakei* | H1 | Human |
| GCF_051387975.1 | *Latilactobacillus sakei* | FZJHZ2M8 | Human |
| GCF_051388535.1 | *Latilactobacillus sakei* | QAHLA3L8 | Food |
| GCF_900215905.1 | *Latilactobacillus sakei* | J18 | Food |
| GCF_900234355.1 | *Latilactobacillus sakei* | J64 | Food |
| GCF_901830485.1 | *Latilactobacillus sakei* | AMBR8 | Human |
| GCF_004354795.1 | *Lentilactobacillus hilgardii* | ATCC 8290 | Others |
| GCF_008694025.1 | *Lentilactobacillus hilgardii* | LH500 | Food |
| GCF_009832765.1 | *Lentilactobacillus hilgardii* | FLUB | Food |
| GCF_011765585.1 | *Lentilactobacillus hilgardii* | LMG 07934 | Food |
| GCF_019955265.1 | *Lentilactobacillus hilgardii* | TMW12196 | Food |
| GCF_019955285.1 | *Lentilactobacillus hilgardii* | TMW 1828 | Food |
| GCF_025191085.1 | *Lentilactobacillus hilgardii* | CIRM-BIA 2119 | Food |
| GCF_052222745.1 | *Lentilactobacillus hilgardii* | Q19 | Food |
| GCF_052589095.1 | *Lentilactobacillus hilgardii* | S5 | Food |
| GCF_902374015.1 | *Lentilactobacillus hilgardii* | MGYG-HGUT-01333 | Human |
| GCF_001437335.1 | *Lentilactobacillus parabuchneri* | DSM 15352 | Others |
| GCF_001677035.1 | *Lentilactobacillus parabuchneri* | IPLA 11122 | Food |
| GCF_001687145.1 | *Lentilactobacillus parabuchneri* | IPLA 11150 | Food |
| GCF_001687155.1 | *Lentilactobacillus parabuchneri* | IPLA 11117 | Food |
| GCF_001922025.1 | *Lentilactobacillus parabuchneri* | FAM21731 | Food |
| GCF_002095615.1 | *Lentilactobacillus parabuchneri* | FAM21823 | Others |
| GCF_002095645.1 | *Lentilactobacillus parabuchneri* | FAM21829 | Food |
| GCF_002095655.1 | *Lentilactobacillus parabuchneri* | FAM21834 | Food |
| GCF_002095695.1 | *Lentilactobacillus parabuchneri* | FAM23165 | Food |
| GCF_002095715.1 | *Lentilactobacillus parabuchneri* | FAM23168 | Food |
| GCF_002095725.1 | *Lentilactobacillus parabuchneri* | FAM23166 | Food |
| GCF_002095795.1 | *Lentilactobacillus parabuchneri* | FAM21809 | Food |
| GCF_002095825.1 | *Lentilactobacillus parabuchneri* | FAM23169 | Food |
| GCF_002095835.1 | *Lentilactobacillus parabuchneri* | FAM23163 | Food |
| GCF_002095845.1 | *Lentilactobacillus parabuchneri* | FAM23164 | Food |
| GCF_002095895.1 | *Lentilactobacillus parabuchneri* | FAM23279 | Food |
| GCF_002095905.1 | *Lentilactobacillus parabuchneri* | FAM23280 | Food |
| GCF_002095915.1 | *Lentilactobacillus parabuchneri* | FAM23281 | Food |
| GCF_005864155.1 | *Lentilactobacillus parabuchneri* | FAM 23169 | Food |
| GCF_014879295.1 | *Lentilactobacillus parabuchneri* | KEM | Others |
| GCF_019265985.1 | *Lentilactobacillus parabuchneri* | IPLA11151 | Food |
| GCF_019266005.1 | *Lentilactobacillus parabuchneri* | IPLA11129 | Food |
| GCF_019266025.1 | *Lentilactobacillus parabuchneri* | IPLA11125 | Food |
| GCF_028200805.1 | *Lentilactobacillus parabuchneri* | YQ007 | Food |
| GCF_029823065.1 | *Lentilactobacillus parabuchneri* | LMG 11457 | Others |
| GCF_040270305.1 | *Lentilactobacillus parabuchneri* | GOS-6 | Animal |
| GCF_045166205.1 | *Lentilactobacillus parabuchneri* | DSM 5987 | Food |
| GCF_000784455.1 | *Levilactobacillus brevis* | WK12 | Others |
| GCF_000875905.1 | *Levilactobacillus brevis* | 15f | Human |
| GCF_001540905.1 | *Levilactobacillus brevis* | Lb1595 | Animal |
| GCF_001676805.1 | *Levilactobacillus brevis* | NPS-QW-145 | Food |
| GCF_001722065.1 | *Levilactobacillus brevis* | DPC 6108 | Human |
| GCF_002093065.1 | *Levilactobacillus brevis* | CRL 2013 | Food |
| GCF_002138395.1 | *Levilactobacillus brevis* | 100D8 | Environment |
| GCF_002173555.1 | *Levilactobacillus brevis* | SRCM101174 | Food |
| GCF_002179515.1 | *Levilactobacillus brevis* | 3M004 | Others |
| GCF_002532185.1 | *Levilactobacillus brevis* | ZG1 | Food |
| GCF_002532245.1 | *Levilactobacillus brevis* | SF9B | Food |
| GCF_002933755.1 | *Levilactobacillus brevis* | NBRC 3345 | Others |
| GCF_003053125.1 | *Levilactobacillus brevis* | DS1_5 | Human |
| GCF_003289085.1 | *Levilactobacillus brevis* | TUCO-5E | Animal |
| GCF_003345725.1 | *Levilactobacillus brevis* | WIKIM12 | Food |
| GCF_003346245.1 | *Levilactobacillus brevis* | KMB_620 | Food |
| GCF_003813165.1 | *Levilactobacillus brevis* | LMT1-73 | Food |
| GCF_004055405.1 | *Levilactobacillus brevis* | SRCM 103306 | Food |
| GCF_006228205.1 | *Levilactobacillus brevis* | UCCLBBS124 | Food |
| GCF_006228225.1 | *Levilactobacillus brevis* | SA-C12 | Others |
| GCF_006228245.1 | *Levilactobacillus brevis* | UCCLB556 | Others |
| GCF_006228305.1 | *Levilactobacillus brevis* | UCCLB521 | Others |
| GCF_006381875.1 | *Levilactobacillus brevis* | TR169 | Food |
| GCF_006381935.1 | *Levilactobacillus brevis* | TR052 | Food |
| GCF_006382065.1 | *Levilactobacillus brevis* | TR055 | Food |
| GCF_006538845.1 | *Levilactobacillus brevis* | NBRC 3960 | Others |
| GCF_006538905.1 | *Levilactobacillus brevis* | NBRC 12005 | Others |
| GCF_006539265.1 | *Levilactobacillus brevis* | NBRC 13110 | Others |
| GCF_013249075.1 | *Levilactobacillus brevis* | BIO5542 | Others |
| GCF_014905055.1 | *Levilactobacillus brevis* | NSMJ23 | Food |
| GCF_015238595.1 | *Levilactobacillus brevis* | HQ1-1 | Food |
| GCF_018588725.1 | *Levilactobacillus brevis* | LBH1073 | Environment |
| GCF_018784425.1 | *Levilactobacillus brevis* | MCC633 | Human |
| GCF_018798865.1 | *Levilactobacillus brevis* | SPC-SNU 70-2 | Food |
| GCF_018916925.1 | *Levilactobacillus brevis* | N38 | Animal |
| GCF_019693335.1 | *Levilactobacillus brevis* | Lbr-35 | Food |
| GCF_020532035.1 | *Levilactobacillus brevis* | ASK-1 | Food |
| GCF_022350025.1 | *Levilactobacillus brevis* | YSJ3 | Food |
| GCF_023744035.1 | *Levilactobacillus brevis* | DRD-195 | Food |
| GCF_023744455.1 | *Levilactobacillus brevis* | DRD-136 | Animal |
| GCF_024330045.1 | *Levilactobacillus brevis* | ?.?.-34 | Food |
| GCF_024800685.1 | *Levilactobacillus brevis* | PL102 | Food |
| GCF_025000005.1 | *Levilactobacillus brevis* | CIRM-BIA 1442 | Food |
| GCF_025122425.1 | *Levilactobacillus brevis* | LBAE A15 | Food |
| GCF_025186345.1 | *Levilactobacillus brevis* | 994 | Food |
| GCF_025194285.1 | *Levilactobacillus brevis* | CIRM-BIA 2232 | Food |
| GCF_025194305.1 | *Levilactobacillus brevis* | CIRM-BIA 2225 | Others |
| GCF_025194485.1 | *Levilactobacillus brevis* | CIRM-BIA 907 | Food |
| GCF_025194505.1 | *Levilactobacillus brevis* | CIRM-BIA 895 | Food |
| GCF_025194515.1 | *Levilactobacillus brevis* | CIRM-BIA 899 | Food |
| GCF_025447255.1 | *Levilactobacillus brevis* | SRCM210362 | Food |
| GCF_026639055.1 | *Levilactobacillus brevis* | SC013 | Food |
| GCF_026930365.1 | *Levilactobacillus brevis* | MB20 | Human |
| GCF_026930425.1 | *Levilactobacillus brevis* | MB1 | Human |
| GCF_026930445.1 | *Levilactobacillus brevis* | MB2 | Human |
| GCF_027108875.1 | *Levilactobacillus brevis* | MB13 | Human |
| GCF_027591835.1 | *Levilactobacillus brevis* | LSF9-1 | Food |
| GCF_030361165.1 | *Levilactobacillus brevis* | TOM.290 | Others |
| GCF_030361325.1 | *Levilactobacillus brevis* | TOM.66 | Others |
| GCF_030378305.1 | *Levilactobacillus brevis* | SRCM217016 | Food |
| GCF_037201785.1 | *Levilactobacillus brevis* | LMT1-73 | Food |
| GCF_039839695.1 | *Levilactobacillus brevis* | MKMB05 | Food |
| GCF_039839865.1 | *Levilactobacillus brevis* | MKMB04 | Food |
| GCF_040425635.1 | *Levilactobacillus brevis* | KL251 | Food |
| GCF_040556855.1 | *Levilactobacillus brevis* | TCI988 | Food |
| GCF_041228385.1 | *Levilactobacillus brevis* | BD-LB | Animal |
| GCF_041870135.1 | *Levilactobacillus brevis* | CHEE98 | Food |
| GCF_043854055.1 | *Levilactobacillus brevis* | YT108 | Animal |
| GCF_045689685.1 | *Levilactobacillus brevis* | A156 | Food |
| GCF_047041105.1 | *Levilactobacillus brevis* | H8 | Food |
| GCF_048565585.1 | *Levilactobacillus brevis* | IBB3735 | Food |
| GCF_050410815.1 | *Levilactobacillus brevis* | CRAI | Food |
| GCF_050846755.1 | *Levilactobacillus brevis* | G2UC | Human |
| GCF_051027615.1 | *Levilactobacillus brevis* | SWP-TKLB01 | Food |
| GCF_051048205.1 | *Levilactobacillus brevis* | KU15176 | Others |
| GCF_051152975.1 | *Levilactobacillus brevis* | BE1107-5-7 | Others |
| GCF_051153015.1 | *Levilactobacillus brevis* | BE1107-5-40 | Others |
| GCF_051153035.1 | *Levilactobacillus brevis* | BE1107-5-35 | Others |
| GCF_051153155.1 | *Levilactobacillus brevis* | BE1103-6-27 | Others |
| GCF_051153215.1 | *Levilactobacillus brevis* | BE1103-6-17 | Others |
| GCF_051153275.1 | *Levilactobacillus brevis* | BE1103-6-14 | Others |
| GCF_051153295.1 | *Levilactobacillus brevis* | BE1103-5-9 | Others |
| GCF_051153375.1 | *Levilactobacillus brevis* | BE1103-5-7 | Others |
| GCF_051153395.1 | *Levilactobacillus brevis* | BE1103-5-8 | Others |
| GCF_051153415.1 | *Levilactobacillus brevis* | BE1103-5-27 | Others |
| GCF_051154345.1 | *Levilactobacillus brevis* | BE1101-5-1 | Others |
| GCF_051154745.1 | *Levilactobacillus brevis* | BE1010-5-31 | Others |
| GCF_051155115.1 | *Levilactobacillus brevis* | BE1004-5-43 | Others |
| GCF_051155555.1 | *Levilactobacillus brevis* | BE1003-5-42 | Others |
| GCF_051155575.1 | *Levilactobacillus brevis* | BE1003-5-37 | Others |
| GCF_051155595.1 | *Levilactobacillus brevis* | BE1003-5-30 | Others |
| GCF_051155635.1 | *Levilactobacillus brevis* | BE1003-5-2 | Others |
| GCF_051157175.1 | *Levilactobacillus brevis* | BE090929-6-37 | Others |
| GCF_051157825.1 | *Levilactobacillus brevis* | BE090929-5-9 | Others |
| GCF_051157895.1 | *Levilactobacillus brevis* | BE090929-5-8 | Others |
| GCF_051157915.1 | *Levilactobacillus brevis* | BE090929-5-7 | Others |
| GCF_051158175.1 | *Levilactobacillus brevis* | BE090929-5-35 | Others |
| GCF_051158275.1 | *Levilactobacillus brevis* | BE090929-5-34 | Others |
| GCF_051158375.1 | *Levilactobacillus brevis* | BE090929-5-31 | Others |
| GCF_051158515.1 | *Levilactobacillus brevis* | BE090929-5-26 | Others |
| GCF_051158535.1 | *Levilactobacillus brevis* | BE090929-5-3 | Others |
| GCF_051158555.1 | *Levilactobacillus brevis* | BE090929-5-22 | Others |
| GCF_051158635.1 | *Levilactobacillus brevis* | BE090929-5-2 | Others |
| GCF_051158655.1 | *Levilactobacillus brevis* | BE090929-5-20 | Others |
| GCF_051158675.1 | *Levilactobacillus brevis* | BE090929-5-19 | Others |
| GCF_051158715.1 | *Levilactobacillus brevis* | BE090929-5-19-MN | Others |
| GCF_051158775.1 | *Levilactobacillus brevis* | BE090929-5-18 | Others |
| GCF_051158795.1 | *Levilactobacillus brevis* | BE090929-5-14 | Others |
| GCF_051158875.1 | *Levilactobacillus brevis* | BE090929-5-10 | Others |
| GCF_051257785.1 | *Levilactobacillus brevis* | L010 | Food |
| GCF_051294845.1 | *Levilactobacillus brevis* | DPL5 | Human |
| GCF_051514545.1 | *Levilactobacillus brevis* | CGMCC NO.3414 | Food |
| GCF_900452615.1 | *Levilactobacillus brevis* | NCTC13386 | Others |
| GCF_925279105.1 | *Levilactobacillus brevis* | IM1470 | Others |
| GCF_925281805.1 | *Levilactobacillus brevis* | IM1457 | Others |
| GCF_925285845.1 | *Levilactobacillus brevis* | IM1297 | Others |
| GCF_964065115.1 | *Levilactobacillus brevis* | - | Others |
| GCF_964065285.1 | *Levilactobacillus brevis* | - | Others |
| GCF_965136155.1 | *Levilactobacillus brevis* | CIP102806T | Others |
| GCF_965213345.1 | *Levilactobacillus brevis* | Lactobacillus brevis VK-A | Others |
| GCF_001243975.1 | *Ligilactobacillus agilis* | Marseille | Others |
| GCF_002218575.1 | *Ligilactobacillus agilis* | UMNLA7 | Animal |
| GCF_002218605.1 | *Ligilactobacillus agilis* | UMNLA6 | Animal |
| GCF_002218725.1 | *Ligilactobacillus agilis* | UMNLA8 | Animal |
| GCF_002237855.1 | *Ligilactobacillus agilis* | UMNLA1 | Animal |
| GCF_002237865.1 | *Ligilactobacillus agilis* | UMNLA4 | Animal |
| GCF_002237875.1 | *Ligilactobacillus agilis* | UMNLA3 | Animal |
| GCF_002237885.1 | *Ligilactobacillus agilis* | UMNLA2 | Animal |
| GCF_002237935.1 | *Ligilactobacillus agilis* | UMNLA5 | Animal |
| GCF_002240375.2 | *Ligilactobacillus agilis* | La3 | Animal |
| GCF_002848175.1 | *Ligilactobacillus agilis* | 268A | Animal |
| GCF_002848195.1 | *Ligilactobacillus agilis* | 273 | Animal |
| GCF_012027835.1 | *Ligilactobacillus agilis* | SW282 | Animal |
| GCF_012489675.1 | *Ligilactobacillus agilis* | SY111 | Others |
| GCF_012489825.1 | *Ligilactobacillus agilis* | SN10121 | Others |
| GCF_012489865.1 | *Ligilactobacillus agilis* | SN811 | Others |
| GCF_012490005.1 | *Ligilactobacillus agilis* | SN4111 | Others |
| GCF_012490125.1 | *Ligilactobacillus agilis* | NB11 | Others |
| GCF_012490225.1 | *Ligilactobacillus agilis* | PTL465 | Others |
| GCF_014893475.1 | *Ligilactobacillus agilis* | YZ054 | Animal |
| GCF_016742775.1 | *Ligilactobacillus agilis* | T8a | Animal |
| GCF_016900275.1 | *Ligilactobacillus agilis* | An795 | Animal |
| GCF_016900285.1 | *Ligilactobacillus agilis* | An796 | Animal |
| GCF_022642225.1 | *Ligilactobacillus agilis* | L6 | Animal |
| GCF_022643045.1 | *Ligilactobacillus agilis* | L3 | Animal |
| GCF_025311455.1 | *Ligilactobacillus agilis* | AM_LB6 | Animal |
| GCF_025311475.1 | *Ligilactobacillus agilis* | AM_LB8 | Animal |
| GCF_027674925.1 | *Ligilactobacillus agilis* | AM110-61 | Human |
| GCF_027674985.1 | *Ligilactobacillus agilis* | AM110-53 | Human |
| GCF_027675035.1 | *Ligilactobacillus agilis* | AM110-45 | Human |
| GCF_027675105.1 | *Ligilactobacillus agilis* | AM110-43 | Human |
| GCF_027723985.1 | *Ligilactobacillus agilis* | AM110-129 | Human |
| GCF_027724045.1 | *Ligilactobacillus agilis* | AM110-13 | Human |
| GCF_030373825.1 | *Ligilactobacillus agilis* | 147_Schadl_VitK | Animal |
| GCF_041081355.1 | *Ligilactobacillus agilis* | W70 | Animal |
| GCF_050916625.1 | *Ligilactobacillus agilis* | LDTM 47 | Animal |
| GCF_902386685.1 | *Ligilactobacillus agilis* | MGYG-HGUT-02390 | Human |
| GCF_001652035.1 | *Ligilactobacillus aviarius* | UMNLAv9 | Animal |
| GCF_001652055.1 | *Ligilactobacillus aviarius* | UMNLAv1 | Animal |
| GCF_001652125.1 | *Ligilactobacillus aviarius* | UMNLAv7 | Animal |
| GCF_001652145.1 | *Ligilactobacillus aviarius* | UMNLAv8 | Animal |
| GCF_001654565.1 | *Ligilactobacillus aviarius* | UMNLAv10 | Animal |
| GCF_001654615.1 | *Ligilactobacillus aviarius* | UMNLAv11 | Animal |
| GCF_001654655.1 | *Ligilactobacillus aviarius* | UMNLAv13 | Animal |
| GCF_001654675.1 | *Ligilactobacillus aviarius* | UMNLAv14 | Animal |
| GCF_002553635.1 | *Ligilactobacillus aviarius* | UMNLAv98 | Animal |
| GCF_002553645.1 | *Ligilactobacillus aviarius* | UMNLAv97 | Animal |
| GCF_016902635.1 | *Ligilactobacillus aviarius* | An347 | Animal |
| GCF_029967555.1 | *Ligilactobacillus aviarius* | CML180 | Animal |
| GCF_030373615.1 | *Ligilactobacillus aviarius* | 151_Feed | Animal |
| GCF_039521485.1 | *Ligilactobacillus aviarius* | JCM 5666 | Animal |
| GCF_947381475.1 | *Ligilactobacillus aviarius* | B009 | Others |
| GCF_947381545.1 | *Ligilactobacillus aviarius* | B056 | Others |
| GCF_947381705.1 | *Ligilactobacillus aviarius* | B118 | Others |
| GCF_947381715.1 | *Ligilactobacillus aviarius* | J35 | Others |
| GCF_947381745.1 | *Ligilactobacillus aviarius* | J25 | Others |
| GCF_947381765.1 | *Ligilactobacillus aviarius* | I11 | Others |
| GCF_947381835.1 | *Ligilactobacillus aviarius* | J01 | Others |
| GCF_947381845.1 | *Ligilactobacillus aviarius* | J03 | Others |
| GCF_947381855.1 | *Ligilactobacillus aviarius* | J28 | Others |
| GCF_010586905.1 | *Ligilactobacillus murinus* | V10 | Others |
| GCF_015235625.1 | *Ligilactobacillus murinus* | 2_2BalbC1BO2_S2 | Animal |
| GCF_026805335.1 | *Ligilactobacillus murinus* | Mic08 | Human |
| GCF_026805365.1 | *Ligilactobacillus murinus* | Mic07 | Human |
| GCF_029369785.1 | *Ligilactobacillus murinus* | PG1-1-10 | Animal |
| GCF_033405435.1 | *Ligilactobacillus murinus* | KD6 | Animal |
| GCF_035904565.1 | *Ligilactobacillus murinus* | PC39 | Animal |
| GCF_039534855.1 | *Ligilactobacillus murinus* | JCM 1717 | Animal |
| GCF_041226725.1 | *Ligilactobacillus murinus* | 1 | Animal |
| GCF_045161885.1 | *Ligilactobacillus murinus* | XYW20210915001 | Animal |
| GCF_045164165.1 | *Ligilactobacillus murinus* | XYW20210915003 | Animal |
| GCF_902362225.1 | *Ligilactobacillus murinus* | MGYG-HGUT-00009 | Human |
| GCF_910573925.1 | *Ligilactobacillus murinus* | MGBC000007 | Animal |
| GCF_910574405.1 | *Ligilactobacillus murinus* | MGBC000047 | Animal |
| GCF_910574705.1 | *Ligilactobacillus murinus* | MGBC000065 | Animal |
| GCF_910574975.1 | *Ligilactobacillus murinus* | MGBC000062 | Animal |
| GCF_910575635.1 | *Ligilactobacillus murinus* | MGBC000185 | Animal |
| GCF_964339165.1 | *Ligilactobacillus murinus* | consensus | Others |
| GCF_000758365.1 | *Ligilactobacillus salivarius* | JCM1046 | Animal |
| GCF_001063855.1 | *Ligilactobacillus salivarius* | 609_LSAL | Human |
| GCF_001067265.1 | *Ligilactobacillus salivarius* | 778_LSAL | Human |
| GCF_001723525.1 | *Ligilactobacillus salivarius* | CICC 23174 | Animal |
| GCF_002079335.1 | *Ligilactobacillus salivarius* | NCIMB8818 | Others |
| GCF_002079365.1 | *Ligilactobacillus salivarius* | NCIMB8816 | Others |
| GCF_002079425.1 | *Ligilactobacillus salivarius* | JCM 1047 | Others |
| GCF_002079435.1 | *Ligilactobacillus salivarius* | L21 | Others |
| GCF_002079465.1 | *Ligilactobacillus salivarius* | JCM 1045 | Others |
| GCF_002079485.1 | *Ligilactobacillus salivarius* | JCM 1044 | Others |
| GCF_002079505.1 | *Ligilactobacillus salivarius* | JCM 1042 | Others |
| GCF_002079525.1 | *Ligilactobacillus salivarius* | JCM 1040 | Others |
| GCF_002079545.1 | *Ligilactobacillus salivarius* | gul2 | Others |
| GCF_002079565.1 | *Ligilactobacillus salivarius* | gul1 | Others |
| GCF_002079585.1 | *Ligilactobacillus salivarius* | DSM 20554 | Others |
| GCF_002079595.1 | *Ligilactobacillus salivarius* | DSM 20492 | Others |
| GCF_002079625.1 | *Ligilactobacillus salivarius* | CCuG47826 | Others |
| GCF_002079645.1 | *Ligilactobacillus salivarius* | CCuG47171 | Others |
| GCF_002079685.1 | *Ligilactobacillus salivarius* | CCuG45735 | Others |
| GCF_002079705.1 | *Ligilactobacillus salivarius* | CCuG44481 | Others |
| GCF_002079715.1 | *Ligilactobacillus salivarius* | CCuG38008 | Others |
| GCF_002079745.1 | *Ligilactobacillus salivarius* | CCuG2753OB | Others |
| GCF_002079765.1 | *Ligilactobacillus salivarius* | AH4331 | Others |
| GCF_002079785.1 | *Ligilactobacillus salivarius* | AH43348 | Others |
| GCF_002079805.1 | *Ligilactobacillus salivarius* | AH43324 | Others |
| GCF_002079845.1 | *Ligilactobacillus salivarius* | AH4231 | Others |
| GCF_002079905.1 | *Ligilactobacillus salivarius* | 01M14315 | Others |
| GCF_002079925.1 | *Ligilactobacillus salivarius* | NCIMB8817 | Others |
| GCF_002159345.1 | *Ligilactobacillus salivarius* | An63 | Animal |
| GCF_002160855.1 | *Ligilactobacillus salivarius* | An128 | Animal |
| GCF_002161265.1 | *Ligilactobacillus salivarius* | An84 | Animal |
| GCF_002162055.1 | *Ligilactobacillus salivarius* | ZLS006 | Animal |
| GCF_002250405.1 | *Ligilactobacillus salivarius* | L28 | Animal |
| GCF_002289925.1 | *Ligilactobacillus salivarius* | KLW009 | Animal |
| GCF_002735985.1 | *Ligilactobacillus salivarius* | BCRC 12574 | Human |
| GCF_002736025.1 | *Ligilactobacillus salivarius* | BCRC 14759 | Human |
| GCF_002738245.1 | *Ligilactobacillus salivarius* | SGL 03 | Human |
| GCF_003052845.1 | *Ligilactobacillus salivarius* | DS3_12 | Human |
| GCF_003052875.1 | *Ligilactobacillus salivarius* | DS13_12 | Human |
| GCF_003061545.1 | *Ligilactobacillus salivarius* | DS11_12 | Human |
| GCF_003061585.1 | *Ligilactobacillus salivarius* | DS2_12 | Human |
| GCF_003438595.1 | *Ligilactobacillus salivarius* | OM08-20 | Human |
| GCF_003470145.1 | *Ligilactobacillus salivarius* | AM25-8 | Human |
| GCF_003472615.1 | *Ligilactobacillus salivarius* | AM09-40 | Human |
| GCF_008016885.1 | *Ligilactobacillus salivarius* | C12 | Animal |
| GCF_008016915.1 | *Ligilactobacillus salivarius* | C2 | Animal |
| GCF_009863295.1 | *Ligilactobacillus salivarius* | FWXBH2_2 | Human |
| GCF_009863365.1 | *Ligilactobacillus salivarius* | FNXYC6M7 | Human |
| GCF_009863375.1 | *Ligilactobacillus salivarius* | FWXBH18_5 | Human |
| GCF_009863405.1 | *Ligilactobacillus salivarius* | FNMGHLBE8_L1 | Human |
| GCF_009863415.1 | *Ligilactobacillus salivarius* | FNMGHLBE13_L1 | Human |
| GCF_009863435.1 | *Ligilactobacillus salivarius* | FNMGHLBE2_L7 | Human |
| GCF_009863465.1 | *Ligilactobacillus salivarius* | FNMGHLBE11_L1_2 | Human |
| GCF_009863485.1 | *Ligilactobacillus salivarius* | FJSWX10_2 | Human |
| GCF_009863495.1 | *Ligilactobacillus salivarius* | FJLHD7M2 | Human |
| GCF_009863515.1 | *Ligilactobacillus salivarius* | FJLHD9M1 | Human |
| GCF_009863535.1 | *Ligilactobacillus salivarius* | FJLHD32M4 | Human |
| GCF_009863555.1 | *Ligilactobacillus salivarius* | FJLHD2M8 | Human |
| GCF_009863585.1 | *Ligilactobacillus salivarius* | FJLHD18M1 | Human |
| GCF_009863595.1 | *Ligilactobacillus salivarius* | FJLHD27M4 | Human |
| GCF_009863605.1 | *Ligilactobacillus salivarius* | FJLHD25M7 | Human |
| GCF_009863625.1 | *Ligilactobacillus salivarius* | FJLHD16M3 | Human |
| GCF_009863635.1 | *Ligilactobacillus salivarius* | FJLHD24M1 | Human |
| GCF_009863685.1 | *Ligilactobacillus salivarius* | FJLHD10M2 | Human |
| GCF_009863695.1 | *Ligilactobacillus salivarius* | FJLHD14M6 | Human |
| GCF_009863705.1 | *Ligilactobacillus salivarius* | FHNXY27_L3 | Human |
| GCF_009863715.1 | *Ligilactobacillus salivarius* | FGSYC47M10 | Human |
| GCF_009863735.1 | *Ligilactobacillus salivarius* | FHNXY73M9 | Human |
| GCF_009863785.1 | *Ligilactobacillus salivarius* | FGSYC2M4_2 | Human |
| GCF_009863805.1 | *Ligilactobacillus salivarius* | FGDLZ35_2.scaf | Human |
| GCF_009863815.1 | *Ligilactobacillus salivarius* | FCQNA25M6 | Human |
| GCF_009863825.1 | *Ligilactobacillus salivarius* | FCQHC3_L6_2 | Human |
| GCF_009863835.1 | *Ligilactobacillus salivarius* | FCQHC8_L1 | Human |
| GCF_009863885.1 | *Ligilactobacillus salivarius* | FBJSY20_2 | Human |
| GCF_009863905.1 | *Ligilactobacillus salivarius* | FAHBZ_8M2 | Human |
| GCF_009863915.1 | *Ligilactobacillus salivarius* | E6_1 | Human |
| GCF_009863925.1 | *Ligilactobacillus salivarius* | 6_2 | Human |
| GCF_009865705.1 | *Ligilactobacillus salivarius* | NT64_1 | Human |
| GCF_009865735.1 | *Ligilactobacillus salivarius* | NT4_8 | Human |
| GCF_009865755.1 | *Ligilactobacillus salivarius* | NT33_2 | Human |
| GCF_009865785.1 | *Ligilactobacillus salivarius* | NT15_1 | Human |
| GCF_009865795.1 | *Ligilactobacillus salivarius* | HN24_2 | Human |
| GCF_009865805.1 | *Ligilactobacillus salivarius* | HN26_4 | Human |
| GCF_009865815.1 | *Ligilactobacillus salivarius* | JSWX5_1 | Human |
| GCF_009865865.1 | *Ligilactobacillus salivarius* | FZJTZ9M6 | Human |
| GCF_009865895.1 | *Ligilactobacillus salivarius* | FZJTZ64M3 | Human |
| GCF_009865925.1 | *Ligilactobacillus salivarius* | FZJTZ27M6_2 | Human |
| GCF_009865965.1 | *Ligilactobacillus salivarius* | FZJTZ13M4 | Human |
| GCF_009865985.1 | *Ligilactobacillus salivarius* | FYNLJ23_2 | Human |
| GCF_009865995.1 | *Ligilactobacillus salivarius* | FZJTZ10M2 | Human |
| GCF_009866015.1 | *Ligilactobacillus salivarius* | FYNDL2_4 | Human |
| GCF_009866025.1 | *Ligilactobacillus salivarius* | FYNDL5_1 | Human |
| GCF_009866035.1 | *Ligilactobacillus salivarius* | FYNDL6_3 | Human |
| GCF_009866085.1 | *Ligilactobacillus salivarius* | FXJWS6M4_2 | Human |
| GCF_009866105.1 | *Ligilactobacillus salivarius* | FXJWS41_2 | Human |
| GCF_009866115.1 | *Ligilactobacillus salivarius* | FXJKS25M8 | Human |
| GCF_009866125.1 | *Ligilactobacillus salivarius* | FXJCJ9_2.scaf | Human |
| GCF_009866135.1 | *Ligilactobacillus salivarius* | FXJSW2_3.scaf | Human |
| GCF_009866185.1 | *Ligilactobacillus salivarius* | FXJCJ7_2 | Human |
| GCF_009866205.1 | *Ligilactobacillus salivarius* | FWXBH4_1 | Human |
| GCF_009866215.1 | *Ligilactobacillus salivarius* | FWXBH9_2 | Human |
| GCF_009866265.1 | *Ligilactobacillus salivarius* | FWXBH25_3 | Human |
| GCF_009866275.1 | *Ligilactobacillus salivarius* | FSDLZ18M11 | Human |
| GCF_009869955.1 | *Ligilactobacillus salivarius* | HENA17_3 | Human |
| GCF_009869975.1 | *Ligilactobacillus salivarius* | FWXBH3_1 | Human |
| GCF_009869985.1 | *Ligilactobacillus salivarius* | FZJTZ28M4 | Human |
| GCF_009870175.1 | *Ligilactobacillus salivarius* | NT62_5 | Human |
| GCF_009870215.1 | *Ligilactobacillus salivarius* | FXJSW20M4_2 | Human |
| GCF_009870225.1 | *Ligilactobacillus salivarius* | FXJKS17M7 | Human |
| GCF_009870285.1 | *Ligilactobacillus salivarius* | FSDLZ17M12 | Human |
| GCF_009870295.1 | *Ligilactobacillus salivarius* | FSDLZ20M1_2 | Human |
| GCF_009870335.1 | *Ligilactobacillus salivarius* | F_SD_HZ_D3_L_5 | Human |
| GCF_009870355.1 | *Ligilactobacillus salivarius* | FJLHD4M1 | Human |
| GCF_009870365.1 | *Ligilactobacillus salivarius* | FJSWX_34J_L5 | Human |
| GCF_011029235.1 | *Ligilactobacillus salivarius* | VA40-10 | Human |
| GCF_011045395.1 | *Ligilactobacillus salivarius* | IBB3154 | Animal |
| GCF_013249205.1 | *Ligilactobacillus salivarius* | BIO6313 | Others |
| GCF_013391745.1 | *Ligilactobacillus salivarius* | KZ-NCB | Animal |
| GCF_013487885.1 | *Ligilactobacillus salivarius* | 2D | Animal |
| GCF_014841055.1 | *Ligilactobacillus salivarius* | ZLp4b | Animal |
| GCF_014982915.1 | *Ligilactobacillus salivarius* | DSM 108969 | Animal |
| GCF_015070845.1 | *Ligilactobacillus salivarius* | 144 | Animal |
| GCF_015159735.1 | *Ligilactobacillus salivarius* | UBLS-22 | Human |
| GCF_015548605.1 | *Ligilactobacillus salivarius* | 1001095A_150126_H2 | Human |
| GCF_015552175.1 | *Ligilactobacillus salivarius* | D6t1_180914_C6 | Human |
| GCF_015557145.1 | *Ligilactobacillus salivarius* | 1001295B_180824_C9 | Human |
| GCF_015560055.1 | *Ligilactobacillus salivarius* | 1001287B_170213_E2 | Human |
| GCF_015560415.1 | *Ligilactobacillus salivarius* | 1001270B_150601_C6 | Human |
| GCF_016742795.1 | *Ligilactobacillus salivarius* | T3 | Animal |
| GCF_016742875.1 | *Ligilactobacillus salivarius* | A19 | Animal |
| GCF_016900035.1 | *Ligilactobacillus salivarius* | An813 | Animal |
| GCF_016900465.1 | *Ligilactobacillus salivarius* | An787 | Animal |
| GCF_019593595.1 | *Ligilactobacillus salivarius* | C3 | Animal |
| GCF_020026485.1 | *Ligilactobacillus salivarius* | AER35 | Animal |
| GCF_020026515.1 | *Ligilactobacillus salivarius* | AER04 | Animal |
| GCF_020026555.1 | *Ligilactobacillus salivarius* | AER36 | Animal |
| GCF_020535185.1 | *Ligilactobacillus salivarius* | AR809 | Human |
| GCF_021266585.1 | *Ligilactobacillus salivarius* | BNS11 | Animal |
| GCF_021432185.1 | *Ligilactobacillus salivarius* | 2102-15 | Human |
| GCF_023573545.1 | *Ligilactobacillus salivarius* | S01 | Animal |
| GCF_024125465.1 | *Ligilactobacillus salivarius* | B27-2 | Human |
| GCF_024397675.1 | *Ligilactobacillus salivarius* | SNK-6 | Animal |
| GCF_024466835.1 | *Ligilactobacillus salivarius* | L.S.05 | Animal |
| GCF_024637975.1 | *Ligilactobacillus salivarius* | VHProbi A17 | Human |
| GCF_024665615.1 | *Ligilactobacillus salivarius* | SS-258 | Human |
| GCF_027665935.1 | *Ligilactobacillus salivarius* | AM79-08pH10TA | Human |
| GCF_027668985.1 | *Ligilactobacillus salivarius* | AM60-15pH4 | Human |
| GCF_027674525.1 | *Ligilactobacillus salivarius* | AM110-98 | Human |
| GCF_027674565.1 | *Ligilactobacillus salivarius* | AM110-97 | Human |
| GCF_027680645.1 | *Ligilactobacillus salivarius* | AF71-03pH5A | Human |
| GCF_027681385.1 | *Ligilactobacillus salivarius* | AF61-10pH5 | Human |
| GCF_027681505.1 | *Ligilactobacillus salivarius* | AF59-11pH5 | Human |
| GCF_027685795.1 | *Ligilactobacillus salivarius* | AF11-8H | Human |
| GCF_027685885.1 | *Ligilactobacillus salivarius* | AF11-29H | Human |
| GCF_027685965.1 | *Ligilactobacillus salivarius* | AF11-20 | Human |
| GCF_027686005.1 | *Ligilactobacillus salivarius* | AF11-17H | Human |
| GCF_027691665.1 | *Ligilactobacillus salivarius* | OM13-15MHA | Human |
| GCF_027692205.1 | *Ligilactobacillus salivarius* | OF46-5pH5 | Human |
| GCF_027692505.1 | *Ligilactobacillus salivarius* | OF42-10pH5A | Human |
| GCF_027695365.1 | *Ligilactobacillus salivarius* | LG55-22 | Human |
| GCF_027695745.1 | *Ligilactobacillus salivarius* | LG100-36 | Human |
| GCF_027698205.1 | *Ligilactobacillus salivarius* | AM93-02F | Human |
| GCF_028656255.1 | *Ligilactobacillus salivarius* | SRCM217603 | Animal |
| GCF_028864315.1 | *Ligilactobacillus salivarius* | 098-2021-6 | Human |
| GCF_028864335.1 | *Ligilactobacillus salivarius* | A7-2-5 | Human |
| GCF_028864355.1 | *Ligilactobacillus salivarius* | A7-1-15 | Human |
| GCF_028864385.1 | *Ligilactobacillus salivarius* | 144-2021-8 | Human |
| GCF_028864405.1 | *Ligilactobacillus salivarius* | A5-1-10 | Human |
| GCF_028864415.1 | *Ligilactobacillus salivarius* | A5-2-10 | Human |
| GCF_028864445.1 | *Ligilactobacillus salivarius* | 198vzk-4 | Human |
| GCF_029070905.1 | *Ligilactobacillus salivarius* | C57 | Human |
| GCF_029350895.1 | *Ligilactobacillus salivarius* | Salm-9 | Animal |
| GCF_029743055.1 | *Ligilactobacillus salivarius* | P1CEA3 | Animal |
| GCF_029823545.1 | *Ligilactobacillus salivarius* | LMG 9477 | Others |
| GCF_029872075.1 | *Ligilactobacillus salivarius* | W57D | Others |
| GCF_029917105.1 | *Ligilactobacillus salivarius* | AR612 | Human |
| GCF_030062785.1 | *Ligilactobacillus salivarius* | S92 | Animal |
| GCF_030062805.1 | *Ligilactobacillus salivarius* | S99 | Animal |
| GCF_030062825.1 | *Ligilactobacillus salivarius* | S32 | Animal |
| GCF_030062845.1 | *Ligilactobacillus salivarius* | S35 | Animal |
| GCF_030062875.1 | *Ligilactobacillus salivarius* | S39 | Animal |
| GCF_030062895.1 | *Ligilactobacillus salivarius* | S40 | Animal |
| GCF_030062915.1 | *Ligilactobacillus salivarius* | S91 | Animal |
| GCF_030062935.1 | *Ligilactobacillus salivarius* | S96 | Animal |
| GCF_030169145.1 | *Ligilactobacillus salivarius* | ZSA5 | Animal |
| GCF_030237775.1 | *Ligilactobacillus salivarius* | C39 | Animal |
| GCF_030263175.1 | *Ligilactobacillus salivarius* | F14 | Human |
| GCF_030371315.1 | *Ligilactobacillus salivarius* | 92_BHI | Animal |
| GCF_030371905.1 | *Ligilactobacillus salivarius* | 30_SSukc10 | Animal |
| GCF_030372485.1 | *Ligilactobacillus salivarius* | ET141 | Animal |
| GCF_030373745.1 | *Ligilactobacillus salivarius* | 150_Feed | Animal |
| GCF_030434015.1 | *Ligilactobacillus salivarius* | SRCM217594 | Animal |
| GCF_030434115.1 | *Ligilactobacillus salivarius* | SRCM217619 | Animal |
| GCF_030518315.1 | *Ligilactobacillus salivarius* | ZK-88 | Others |
| GCF_030876765.1 | *Ligilactobacillus salivarius* | B10 | Human |
| GCF_031557155.1 | *Ligilactobacillus salivarius* | S103 | Animal |
| GCF_033194755.1 | *Ligilactobacillus salivarius* | CavFT-hAR125 | Human |
| GCF_033344995.1 | *Ligilactobacillus salivarius* | BF12 | Animal |
| GCF_035231985.1 | *Ligilactobacillus salivarius* | B4311 | Animal |
| GCF_038020345.1 | *Ligilactobacillus salivarius* | UO.C249 | Animal |
| GCF_039612535.1 | *Ligilactobacillus salivarius* | B60 | Human |
| GCF_039612545.1 | *Ligilactobacillus salivarius* | B37 | Human |
| GCF_039751015.1 | *Ligilactobacillus salivarius* | A4 | Human |
| GCF_039757375.1 | *Ligilactobacillus salivarius* | D10 | Human |
| GCF_039757415.1 | *Ligilactobacillus salivarius* | H2 | Human |
| GCF_039757455.1 | *Ligilactobacillus salivarius* | G12 | Human |
| GCF_039757465.1 | *Ligilactobacillus salivarius* | C9 | Human |
| GCF_039757495.1 | *Ligilactobacillus salivarius* | B1 | Human |
| GCF_039757555.1 | *Ligilactobacillus salivarius* | A1 | Human |
| GCF_040285095.1 | *Ligilactobacillus salivarius* | NCK1352 | Human |
| GCF_040285135.1 | *Ligilactobacillus salivarius* | NCK1355 | Human |
| GCF_040566435.1 | *Ligilactobacillus salivarius* | C1-4 | Animal |
| GCF_040720955.1 | *Ligilactobacillus salivarius* | C2-3 | Animal |
| GCF_040924755.1 | *Ligilactobacillus salivarius* | G10 | Human |
| GCF_040924775.1 | *Ligilactobacillus salivarius* | D6 | Human |
| GCF_040924825.1 | *Ligilactobacillus salivarius* | D10 | Human |
| GCF_040924845.1 | *Ligilactobacillus salivarius* | A9 | Human |
| GCF_040924865.1 | *Ligilactobacillus salivarius* | A10 | Human |
| GCF_040924885.1 | *Ligilactobacillus salivarius* | A6 | Human |
| GCF_041021485.1 | *Ligilactobacillus salivarius* | Lac45 | Human |
| GCF_041021505.1 | *Ligilactobacillus salivarius* | NL5 | Human |
| GCF_041021525.1 | *Ligilactobacillus salivarius* | Lac40 | Human |
| GCF_042714325.1 | *Ligilactobacillus salivarius* | P1CEA3 | Animal |
| GCF_042715415.1 | *Ligilactobacillus salivarius* | PG21 | Animal |
| GCF_046531985.1 | *Ligilactobacillus salivarius* | LZZAY01 | Animal |
| GCF_046938615.1 | *Ligilactobacillus salivarius* | M698A | Animal |
| GCF_046938655.1 | *Ligilactobacillus salivarius* | M688A | Animal |
| GCF_047946765.1 | *Ligilactobacillus salivarius* | XIF24 | Human |
| GCF_048381415.1 | *Ligilactobacillus salivarius* | SuAm7 | Human |
| GCF_048452405.1 | *Ligilactobacillus salivarius* | BH7-4s | Human |
| GCF_048452685.1 | *Ligilactobacillus salivarius* | BH1-7 | Human |
| GCF_049056805.1 | *Ligilactobacillus salivarius* | GX118 | Environment |
| GCF_049532915.1 | *Ligilactobacillus salivarius* | Y20 | Animal |
| GCF_050847015.1 | *Ligilactobacillus salivarius* | TGM 0103 | Animal |
| GCF_051371065.1 | *Ligilactobacillus salivarius* | HC859 | Animal |
| GCF_052064895.1 | *Ligilactobacillus salivarius* | W58 | Human |
| GCF_900094615.1 | *Ligilactobacillus salivarius* | LPM01 | Others |
| GCF_902385835.1 | *Ligilactobacillus salivarius* | MGYG-HGUT-02324 | Human |
| GCF_925291425.1 | *Ligilactobacillus salivarius* | IM849 | Others |
| GCF_965136025.1 | *Ligilactobacillus salivarius* | CIP103140T | Others |
| GCF_002242615.1 | *Limosilactobacillus fermentum* | SK152 | Food |
| GCF_003255875.1 | *Limosilactobacillus fermentum* | CBA7106 | Human |
| GCF_003346795.1 | *Limosilactobacillus fermentum* | LDTM 7301 | Food |
| GCF_004063515.1 | *Limosilactobacillus fermentum* | SRCM103285 | Food |
| GCF_005341425.1 | *Limosilactobacillus fermentum* | B1 28 | Food |
| GCF_011290755.1 | *Limosilactobacillus fermentum* | CVM-347 | Human |
| GCF_012273035.1 | *Limosilactobacillus fermentum* | HFD1 | Human |
| GCF_013394085.1 | *Limosilactobacillus fermentum* | DSM 20052 | Food |
| GCF_016617695.1 | *Limosilactobacillus fermentum* | B44 | Animal |
| GCF_018884205.1 | *Limosilactobacillus fermentum* | YLF016 | Food |
| GCF_022509425.1 | *Limosilactobacillus fermentum* | QAULFN21 | Food |
| GCF_024204625.1 | *Limosilactobacillus fermentum* | DM075 | Human |
| GCF_024385625.1 | *Limosilactobacillus fermentum* | KHUD_007 | Human |
| GCF_026930525.1 | *Limosilactobacillus fermentum* | MC1 | Human |
| GCF_027696965.1 | *Limosilactobacillus fermentum* | CM02-01b2 | Human |
| GCF_028463945.1 | *Limosilactobacillus fermentum* | MWLf-4 | Human |
| GCF_028743095.1 | *Limosilactobacillus fermentum* | B-1840 | Human |
| GCF_029961225.1 | *Limosilactobacillus fermentum* | EFEL6800 | Human |
| GCF_030665445.1 | *Limosilactobacillus fermentum* | FUA033 | Human |
| GCF_030770375.1 | *Limosilactobacillus fermentum* | B-1840 | Human |
| GCF_030845215.1 | *Limosilactobacillus fermentum* | A51 | Food |
| GCF_031460995.1 | *Limosilactobacillus fermentum* | KCKM 0998 | Food |
| GCF_037986765.1 | *Limosilactobacillus fermentum* | XW411 | Human |
| GCF_037986805.1 | *Limosilactobacillus fermentum* | XW331 | Human |
| GCF_037986825.1 | *Limosilactobacillus fermentum* | XW341 | Human |
| GCF_037986955.1 | *Limosilactobacillus fermentum* | WX61 | Human |
| GCF_037986975.1 | *Limosilactobacillus fermentum* | XC416 | Human |
| GCF_037986985.1 | *Limosilactobacillus fermentum* | XC263 | Human |
| GCF_037987015.1 | *Limosilactobacillus fermentum* | WX91 | Human |
| GCF_037987065.1 | *Limosilactobacillus fermentum* | WX213 | Human |
| GCF_037987085.1 | *Limosilactobacillus fermentum* | WX183 | Human |
| GCF_037987375.1 | *Limosilactobacillus fermentum* | SL271 | Human |
| GCF_037987405.1 | *Limosilactobacillus fermentum* | SL211 | Human |
| GCF_037987425.1 | *Limosilactobacillus fermentum* | SL185 | Human |
| GCF_037987525.1 | *Limosilactobacillus fermentum* | SH251 | Human |
| GCF_037987545.1 | *Limosilactobacillus fermentum* | SH171 | Human |
| GCF_037987575.1 | *Limosilactobacillus fermentum* | SH131 | Human |
| GCF_037987625.1 | *Limosilactobacillus fermentum* | SH281 | Human |
| GCF_037987635.1 | *Limosilactobacillus fermentum* | SH371 | Human |
| GCF_037987765.1 | *Limosilactobacillus fermentum* | QH181 | Human |
| GCF_037987785.1 | *Limosilactobacillus fermentum* | NX681 | Human |
| GCF_037987865.1 | *Limosilactobacillus fermentum* | NX642 | Human |
| GCF_037988005.1 | *Limosilactobacillus fermentum* | JX192 | Human |
| GCF_037988035.1 | *Limosilactobacillus fermentum* | JX111 | Human |
| GCF_037990595.1 | *Limosilactobacillus fermentum* | GD752 | Human |
| GCF_037990675.1 | *Limosilactobacillus fermentum* | GD673 | Human |
| GCF_037990685.1 | *Limosilactobacillus fermentum* | GD661 | Human |
| GCF_037990725.1 | *Limosilactobacillus fermentum* | GD363 | Human |
| GCF_037990775.1 | *Limosilactobacillus fermentum* | GD351 | Human |
| GCF_037990835.1 | *Limosilactobacillus fermentum* | GD131 | Human |
| GCF_037990855.1 | *Limosilactobacillus fermentum* | GD181 | Human |
| GCF_037990875.1 | *Limosilactobacillus fermentum* | GD121 | Human |
| GCF_037990915.1 | *Limosilactobacillus fermentum* | GD111 | Human |
| GCF_037990925.1 | *Limosilactobacillus fermentum* | FJ181 | Human |
| GCF_037990955.1 | *Limosilactobacillus fermentum* | FJ16 | Human |
| GCF_037991015.1 | *Limosilactobacillus fermentum* | FJ112 | Human |
| GCF_037991055.1 | *Limosilactobacillus fermentum* | CQ61 | Human |
| GCF_037991115.1 | *Limosilactobacillus fermentum* | CQ272 | Human |
| GCF_041380155.1 | *Limosilactobacillus fermentum* | KA16MA | Others |
| GCF_041413225.1 | *Limosilactobacillus fermentum* | SN4-1 | Food |
| GCF_045272275.1 | *Limosilactobacillus fermentum* | MRD072 | Human |
| GCF_046603855.1 | *Limosilactobacillus fermentum* | SFAD 38 | Human |
| GCF_049070625.1 | *Limosilactobacillus fermentum* | DS2875 | Human |
| GCF_050032855.1 | *Limosilactobacillus fermentum* | A10 | Food |
| GCF_051045955.1 | *Limosilactobacillus fermentum* | HF06 | Food |
| GCF_051094845.1 | *Limosilactobacillus fermentum* | PV22 | Human |
| GCF_051153515.1 | *Limosilactobacillus fermentum* | BE1101-8-9 | Others |
| GCF_051153535.1 | *Limosilactobacillus fermentum* | BE1101-8-8 | Others |
| GCF_051153715.1 | *Limosilactobacillus fermentum* | BE1101-8-2 | Others |
| GCF_051154975.1 | *Limosilactobacillus fermentum* | BE1008-6-4b | Others |
| GCF_051155415.1 | *Limosilactobacillus fermentum* | BE1003-8-36 | Others |
| GCF_051156615.1 | *Limosilactobacillus fermentum* | BE1001-5-32 | Others |
| GCF_051628875.1 | *Limosilactobacillus fermentum* | P91 | Others |
| GCF_052029635.1 | *Limosilactobacillus fermentum* | JAC 231 | Human |
| GCF_027662565.1 | *Limosilactobacillus mucosae* | AM08-5 | Human |
| GCF_027693345.1 | *Limosilactobacillus mucosae* | OF26-1b12A | Human |
| GCF_027693385.1 | *Limosilactobacillus mucosae* | OF26-11pH9A | Human |
| GCF_027693545.1 | *Limosilactobacillus mucosae* | OF25-1pH5A | Human |
| GCF_027693785.1 | *Limosilactobacillus mucosae* | OF23-1pH5A | Human |
| GCF_027693805.1 | *Limosilactobacillus mucosae* | OF21-12A | Human |
| GCF_027697925.1 | *Limosilactobacillus mucosae* | AM96-01DM3TA-r | Human |
| GCF_028401825.1 | *Limosilactobacillus mucosae* | F88 | Animal |
| GCF_028401985.1 | *Limosilactobacillus mucosae* | F108 | Animal |
| GCF_028402065.1 | *Limosilactobacillus mucosae* | F146 | Animal |
| GCF_029076635.1 | *Limosilactobacillus mucosae* | DFI.5.78 | Human |
| GCF_046939535.1 | *Limosilactobacillus mucosae* | M212A | Animal |
| GCF_046939625.1 | *Limosilactobacillus mucosae* | M184A | Animal |
| GCF_049562395.1 | *Limosilactobacillus mucosae* | Q2 | Animal |
| GCF_050313655.1 | *Limosilactobacillus mucosae* | SGI.163 | Animal |
| GCF_052492655.1 | *Limosilactobacillus mucosae* | SLAM-JH01 | Animal |
| GCF_001046835.1 | *Limosilactobacillus reuteri* | IRT | Human |
| GCF_001618905.1 | *Limosilactobacillus reuteri* | ZLR003 | Animal |
| GCF_001657495.1 | *Limosilactobacillus reuteri* | CRL 1098 | Food |
| GCF_001688685.2 | *Limosilactobacillus reuteri* | I49 | Animal |
| GCF_001703855.1 | *Limosilactobacillus reuteri* | 480_44 | Animal |
| GCF_001703865.1 | *Limosilactobacillus reuteri* | 482_46 | Animal |
| GCF_001703885.1 | *Limosilactobacillus reuteri* | 482_54 | Animal |
| GCF_001703935.1 | *Limosilactobacillus reuteri* | 484_39 | Animal |
| GCF_001705505.1 | *Limosilactobacillus reuteri* | P43 | Animal |
| GCF_001889975.1 | *Limosilactobacillus reuteri* | 121 | Animal |
| GCF_002007085.2 | *Limosilactobacillus reuteri* | MD IIE-43 | Human |
| GCF_002112185.1 | *Limosilactobacillus reuteri* | 1366 | Animal |
| GCF_002112195.1 | *Limosilactobacillus reuteri* | M27U15 | Human |
| GCF_002112225.1 | *Limosilactobacillus reuteri* | JCM 1081 | Animal |
| GCF_002112245.1 | *Limosilactobacillus reuteri* | CSF8 | Animal |
| GCF_002112805.1 | *Limosilactobacillus reuteri* | MM34-4A | Human |
| GCF_002156605.1 | *Limosilactobacillus reuteri* | LR0 | Animal |
| GCF_002221655.1 | *Limosilactobacillus reuteri* | I49 | Animal |
| GCF_002253625.1 | *Limosilactobacillus reuteri* | 609i | Animal |
| GCF_002253665.1 | *Limosilactobacillus reuteri* | 609l | Animal |
| GCF_002253685.1 | *Limosilactobacillus reuteri* | 609e | Animal |
| GCF_002253705.1 | *Limosilactobacillus reuteri* | 609d | Animal |
| GCF_002253725.1 | *Limosilactobacillus reuteri* | 601g | Animal |
| GCF_002253745.1 | *Limosilactobacillus reuteri* | 601l | Animal |
| GCF_002253765.1 | *Limosilactobacillus reuteri* | 117w | Animal |
| GCF_002253785.1 | *Limosilactobacillus reuteri* | 117r | Animal |
| GCF_002253825.1 | *Limosilactobacillus reuteri* | 114h | Animal |
| GCF_002253835.1 | *Limosilactobacillus reuteri* | 114g | Animal |
| GCF_002253865.1 | *Limosilactobacillus reuteri* | 114q | Animal |
| GCF_002253875.1 | *Limosilactobacillus reuteri* | 117n | Animal |
| GCF_002253905.1 | *Limosilactobacillus reuteri* | 111v | Animal |
| GCF_002253925.1 | *Limosilactobacillus reuteri* | 107k | Animal |
| GCF_002253945.1 | *Limosilactobacillus reuteri* | 111l | Animal |
| GCF_002253955.1 | *Limosilactobacillus reuteri* | 107j | Animal |
| GCF_002253965.1 | *Limosilactobacillus reuteri* | 111b | Animal |
| GCF_002253975.1 | *Limosilactobacillus reuteri* | 111f | Animal |
| GCF_002254025.1 | *Limosilactobacillus reuteri* | 105p | Animal |
| GCF_002254035.1 | *Limosilactobacillus reuteri* | 107e | Animal |
| GCF_002254045.1 | *Limosilactobacillus reuteri* | 105o | Animal |
| GCF_002254085.1 | *Limosilactobacillus reuteri* | 105k | Animal |
| GCF_002254095.1 | *Limosilactobacillus reuteri* | 105w | Animal |
| GCF_002254105.1 | *Limosilactobacillus reuteri* | 105n | Animal |
| GCF_002254115.1 | *Limosilactobacillus reuteri* | 105i | Animal |
| GCF_002254165.1 | *Limosilactobacillus reuteri* | 105c | Animal |
| GCF_002254175.1 | *Limosilactobacillus reuteri* | 105d | Animal |
| GCF_002254185.1 | *Limosilactobacillus reuteri* | 103v | Animal |
| GCF_002254195.1 | *Limosilactobacillus reuteri* | 103p | Animal |
| GCF_002254245.1 | *Limosilactobacillus reuteri* | 103o | Animal |
| GCF_002254255.1 | *Limosilactobacillus reuteri* | 103b | Animal |
| GCF_002762415.1 | *Limosilactobacillus reuteri* | RC-14 | Food |
| GCF_002888655.1 | *Limosilactobacillus reuteri* | 20 | Animal |
| GCF_003046055.1 | *Limosilactobacillus reuteri* | LR5A | Food |
| GCF_003046135.1 | *Limosilactobacillus reuteri* | E81 | Food |
| GCF_003053005.1 | *Limosilactobacillus reuteri* | DS12_10 | Food |
| GCF_003061685.1 | *Limosilactobacillus reuteri* | DS14_10 | Food |
| GCF_003061745.1 | *Limosilactobacillus reuteri* | DS13_10 | Food |
| GCF_003064585.1 | *Limosilactobacillus reuteri* | DS22_10 | Food |
| GCF_003065345.1 | *Limosilactobacillus reuteri* | DS17_10 | Food |
| GCF_003072625.1 | *Limosilactobacillus reuteri* | WHH1689 | Food |
| GCF_003174815.1 | *Limosilactobacillus reuteri* | LR18 | Animal |
| GCF_003174855.1 | *Limosilactobacillus reuteri* | LR4 | Animal |
| GCF_003174865.1 | *Limosilactobacillus reuteri* | LR9 | Animal |
| GCF_003174875.1 | *Limosilactobacillus reuteri* | LR8 | Animal |
| GCF_003174915.1 | *Limosilactobacillus reuteri* | LR17 | Animal |
| GCF_003174935.1 | *Limosilactobacillus reuteri* | LR3 | Animal |
| GCF_003174945.1 | *Limosilactobacillus reuteri* | LR13 | Animal |
| GCF_003174955.1 | *Limosilactobacillus reuteri* | LR14 | Animal |
| GCF_003174995.1 | *Limosilactobacillus reuteri* | LR11 | Animal |
| GCF_003175015.1 | *Limosilactobacillus reuteri* | LR2 | Animal |
| GCF_003175025.1 | *Limosilactobacillus reuteri* | LR19 | Animal |
| GCF_003175075.1 | *Limosilactobacillus reuteri* | LR10 | Animal |
| GCF_003175125.1 | *Limosilactobacillus reuteri* | LR1 | Animal |
| GCF_003316895.1 | *Limosilactobacillus reuteri* | Byun-re-01 | Animal |
| GCF_003316935.1 | *Limosilactobacillus reuteri* | SKKU-OGDONS-01 | Animal |
| GCF_003703875.1 | *Limosilactobacillus reuteri* | R2lc | Animal |
| GCF_003703885.1 | *Limosilactobacillus reuteri* | 2010 | Animal |
| GCF_003719715.1 | *Limosilactobacillus reuteri* | UBLRU-87 | Food |
| GCF_004208615.1 | *Limosilactobacillus reuteri* | ATG-F4 | Human |
| GCF_004349655.1 | *Limosilactobacillus reuteri* | C93 | Human |
| GCF_004349685.1 | *Limosilactobacillus reuteri* | C88 | Human |
| GCF_004684995.1 | *Limosilactobacillus reuteri* | I8-5 | Animal |
| GCF_004794115.1 | *Limosilactobacillus reuteri* | NM12_1-47 | Animal |
| GCF_006874665.1 | *Limosilactobacillus reuteri* | YSJL-12 | Animal |
| GCF_007280535.1 | *Limosilactobacillus reuteri* | BIO5454 | Food |
| GCF_007633215.1 | *Limosilactobacillus reuteri* | LL7 | Animal |
| GCF_009184725.1 | *Limosilactobacillus reuteri* | reuteri | Human |
| GCF_009389015.1 | *Limosilactobacillus reuteri* | PTA6_F1 | Animal |
| GCF_009389035.1 | *Limosilactobacillus reuteri* | PTA6_F2 | Animal |
| GCF_009389105.1 | *Limosilactobacillus reuteri* | PTA6_C7 | Animal |
| GCF_009389155.1 | *Limosilactobacillus reuteri* | PTA5_C5 | Animal |
| GCF_009389165.1 | *Limosilactobacillus reuteri* | PTA5_C13 | Animal |
| GCF_009389205.1 | *Limosilactobacillus reuteri* | PTA5_C4 | Animal |
| GCF_009389215.1 | *Limosilactobacillus reuteri* | PTA5_F13 | Animal |
| GCF_009389245.1 | *Limosilactobacillus reuteri* | PTA5_F1 | Animal |
| GCF_009389255.1 | *Limosilactobacillus reuteri* | PTA5_F11 | Animal |
| GCF_009389285.1 | *Limosilactobacillus reuteri* | PTA5_F4 | Animal |
| GCF_009389305.1 | *Limosilactobacillus reuteri* | PTA4_C4 | Animal |
| GCF_009389325.1 | *Limosilactobacillus reuteri* | PTA4_C2 | Animal |
| GCF_009389335.1 | *Limosilactobacillus reuteri* | PTA4_C1 | Animal |
| GCF_009389365.1 | *Limosilactobacillus reuteri* | PTA1_F3 | Animal |
| GCF_009389375.1 | *Limosilactobacillus reuteri* | PTA2_C2 | Animal |
| GCF_009389385.1 | *Limosilactobacillus reuteri* | PTA1_C4 | Animal |
| GCF_009389425.1 | *Limosilactobacillus reuteri* | PTA1_C1 | Animal |
| GCF_009389435.1 | *Limosilactobacillus reuteri* | PTA1_C3 | Animal |
| GCF_009389465.1 | *Limosilactobacillus reuteri* | PTA8_1 | Animal |
| GCF_009389475.1 | *Limosilactobacillus reuteri* | PTA5_11 | Animal |
| GCF_009649095.1 | *Limosilactobacillus reuteri* | Rat19 | Animal |
| GCF_009649445.1 | *Limosilactobacillus reuteri* | L1600-1 | Animal |
| GCF_009649475.1 | *Limosilactobacillus reuteri* | N2J | Animal |
| GCF_009649505.1 | *Limosilactobacillus reuteri* | CR | Animal |
| GCF_009649535.1 | *Limosilactobacillus reuteri* | One-one | Animal |
| GCF_009649545.1 | *Limosilactobacillus reuteri* | L1604-1 | Animal |
| GCF_009649565.1 | *Limosilactobacillus reuteri* | Lr4020 | Animal |
| GCF_009649605.1 | *Limosilactobacillus reuteri* | N4I | Animal |
| GCF_009733615.2 | *Limosilactobacillus reuteri* | G01_USFQ | Animal |
| GCF_009733645.1 | *Limosilactobacillus reuteri* | G03_USFQ | Animal |
| GCF_009733655.1 | *Limosilactobacillus reuteri* | G02_USFQ | Animal |
| GCF_010206285.1 | *Limosilactobacillus reuteri* | MD207 | Animal |
| GCF_012275185.1 | *Limosilactobacillus reuteri* | CNI-KCA2 | Animal |
| GCF_012971015.1 | *Limosilactobacillus reuteri* | Z6 | Food |
| GCF_013348825.1 | *Limosilactobacillus reuteri* | AN417 | Animal |
| GCF_013694365.1 | *Limosilactobacillus reuteri* | CNEI-KCA3 | Animal |
| GCF_014145445.1 | *Limosilactobacillus reuteri* | AP3 | Animal |
| GCF_015377805.1 | *Limosilactobacillus reuteri* | TK-F8A | Food |
| GCF_015552675.1 | *Limosilactobacillus reuteri* | BSD2780061688 | Human |
| GCF_016697045.1 | *Limosilactobacillus reuteri* | I49 | Animal |
| GCF_018884225.1 | *Limosilactobacillus reuteri* | YLR001 | Food |
| GCF_019336465.1 | *Limosilactobacillus reuteri* | Fn041 | Human |
| GCF_020412465.1 | *Limosilactobacillus reuteri* | 19-E-6 | Animal |
| GCF_020412485.1 | *Limosilactobacillus reuteri* | 19-E-3 | Animal |
| GCF_020784195.1 | *Limosilactobacillus reuteri* | BHM_Thaddeus_3_1 | Animal |
| GCF_020784205.1 | *Limosilactobacillus reuteri* | AG_2_7_1 | Animal |
| GCF_020784255.1 | *Limosilactobacillus reuteri* | 2A | Animal |
| GCF_020784275.1 | *Limosilactobacillus reuteri* | M_81 | Animal |
| GCF_020784295.1 | *Limosilactobacillus reuteri* | M_2 | Animal |
| GCF_020784305.1 | *Limosilactobacillus reuteri* | M_20 | Animal |
| GCF_020784315.1 | *Limosilactobacillus reuteri* | O_Tuki_6_1 | Animal |
| GCF_020784355.1 | *Limosilactobacillus reuteri* | S_1_1 | Animal |
| GCF_020784375.1 | *Limosilactobacillus reuteri* | R.n.2-A | Animal |
| GCF_020784395.1 | *Limosilactobacillus reuteri* | S_9_1 | Animal |
| GCF_020784415.1 | *Limosilactobacillus reuteri* | S_5_1 | Animal |
| GCF_020784435.1 | *Limosilactobacillus reuteri* | R.n.1-B | Animal |
| GCF_020784455.1 | *Limosilactobacillus reuteri* | FUA 3048 | Animal |
| GCF_020784465.1 | *Limosilactobacillus reuteri* | R.n.3-A | Animal |
| GCF_020784495.1 | *Limosilactobacillus reuteri* | FUA 3043 | Animal |
| GCF_020784515.1 | *Limosilactobacillus reuteri* | FUA 3041 | Animal |
| GCF_020784525.1 | *Limosilactobacillus reuteri* | 3B | Animal |
| GCF_020784555.1 | *Limosilactobacillus reuteri* | D.l.1-B | Animal |
| GCF_020784575.1 | *Limosilactobacillus reuteri* | C.p.1-C | Animal |
| GCF_020784595.1 | *Limosilactobacillus reuteri* | C4 | Animal |
| GCF_020784605.1 | *Limosilactobacillus reuteri* | C3 | Animal |
| GCF_020784635.1 | *Limosilactobacillus reuteri* | D.l.3-A | Animal |
| GCF_020784655.1 | *Limosilactobacillus reuteri* | CC-AA2-1 | Animal |
| GCF_020784665.1 | *Limosilactobacillus reuteri* | CC-AA2-2 | Animal |
| GCF_020784695.1 | *Limosilactobacillus reuteri* | WF-AA1-A | Animal |
| GCF_020784715.1 | *Limosilactobacillus reuteri* | O_Tuki_7_1 | Animal |
| GCF_020784725.1 | *Limosilactobacillus reuteri* | WF-AA1-C | Animal |
| GCF_020784755.1 | *Limosilactobacillus reuteri* | O_Ruby_9_1 | Animal |
| GCF_020784775.1 | *Limosilactobacillus reuteri* | O_Ruby_8_1 | Animal |
| GCF_020784795.1 | *Limosilactobacillus reuteri* | apa71 | Animal |
| GCF_020784805.1 | *Limosilactobacillus reuteri* | PM_Patrick_6_1 | Animal |
| GCF_020784875.1 | *Limosilactobacillus reuteri* | PM_Patrick_1_1 | Animal |
| GCF_020784895.1 | *Limosilactobacillus reuteri* | PM_Patrick_2_1 | Animal |
| GCF_020784915.1 | *Limosilactobacillus reuteri* | M_Angie_4_1 | Animal |
| GCF_020784935.1 | *Limosilactobacillus reuteri* | M_Angie_1_1 | Animal |
| GCF_020784945.1 | *Limosilactobacillus reuteri* | 6C | Animal |
| GCF_020784975.1 | *Limosilactobacillus reuteri* | 6B | Animal |
| GCF_020784995.1 | *Limosilactobacillus reuteri* | 6A | Animal |
| GCF_020785015.1 | *Limosilactobacillus reuteri* | LTM_Sauna_1_1 | Animal |
| GCF_020785025.1 | *Limosilactobacillus reuteri* | SM_1_4_1 | Animal |
| GCF_020785035.1 | *Limosilactobacillus reuteri* | LTM_Jesus_5_1 | Animal |
| GCF_020785075.1 | *Limosilactobacillus reuteri* | G_6_1 | Animal |
| GCF_020785095.1 | *Limosilactobacillus reuteri* | LTM_Jesus_1_1 | Animal |
| GCF_020785115.1 | *Limosilactobacillus reuteri* | BHM_Lincecum_1_1 | Animal |
| GCF_020785135.1 | *Limosilactobacillus reuteri* | BHM_Lincecum_2_1 | Animal |
| GCF_020785145.1 | *Limosilactobacillus reuteri* | BHM_Thaddeus_2_1 | Animal |
| GCF_020785175.1 | *Limosilactobacillus reuteri* | AG_2_6_1 | Animal |
| GCF_020785185.1 | *Limosilactobacillus reuteri* | AG_3_1_1 | Animal |
| GCF_020785225.1 | *Limosilactobacillus reuteri* | 21B | Animal |
| GCF_020785255.1 | *Limosilactobacillus reuteri* | PNG008C_M | Human |
| GCF_020785275.1 | *Limosilactobacillus reuteri* | 21A | Animal |
| GCF_020785295.1 | *Limosilactobacillus reuteri* | PB-W2 | Human |
| GCF_020785305.1 | *Limosilactobacillus reuteri* | AG_1_1_1 | Animal |
| GCF_020785325.1 | *Limosilactobacillus reuteri* | PNG008A_M | Human |
| GCF_020785355.1 | *Limosilactobacillus reuteri* | PB-W1 | Human |
| GCF_020785395.1 | *Limosilactobacillus reuteri* | MV4-1a | Human |
| GCF_020785415.1 | *Limosilactobacillus reuteri* | PNG008_48h | Human |
| GCF_020785435.1 | *Limosilactobacillus reuteri* | M81-R43 | Human |
| GCF_020785455.1 | *Limosilactobacillus reuteri* | MM36-1a | Human |
| GCF_020785475.1 | *Limosilactobacillus reuteri* | PNG008_24h | Human |
| GCF_020785495.1 | *Limosilactobacillus reuteri* | FJ3 | Human |
| GCF_020785505.1 | *Limosilactobacillus reuteri* | ME-261 | Human |
| GCF_020785535.1 | *Limosilactobacillus reuteri* | ME-262 | Human |
| GCF_020785545.1 | *Limosilactobacillus reuteri* | SR14 | Human |
| GCF_020785575.1 | *Limosilactobacillus reuteri* | LMS11-1 | Human |
| GCF_020785585.1 | *Limosilactobacillus reuteri* | LMS11-3 | Human |
| GCF_020785595.1 | *Limosilactobacillus reuteri* | SR11 | Human |
| GCF_020785635.1 | *Limosilactobacillus reuteri* | Cor137_1_1 | Animal |
| GCF_020785655.1 | *Limosilactobacillus reuteri* | Cor124_1_1 | Animal |
| GCF_020785665.1 | *Limosilactobacillus reuteri* | T3 | Animal |
| GCF_020785675.1 | *Limosilactobacillus reuteri* | T1 | Animal |
| GCF_020785715.1 | *Limosilactobacillus reuteri* | tu160 | Animal |
| GCF_020785735.1 | *Limosilactobacillus reuteri* | Cor137_3_1 | Animal |
| GCF_020785765.1 | *Limosilactobacillus reuteri* | GQ_1_7_1 | Animal |
| GCF_020785795.1 | *Limosilactobacillus reuteri* | GQ_1_3_1 | Animal |
| GCF_020785815.1 | *Limosilactobacillus reuteri* | L3B | Animal |
| GCF_020785835.1 | *Limosilactobacillus reuteri* | GP6 | Animal |
| GCF_020785855.1 | *Limosilactobacillus reuteri* | GP1 | Animal |
| GCF_020785865.1 | *Limosilactobacillus reuteri* | GP3 | Animal |
| GCF_020785895.1 | *Limosilactobacillus reuteri* | EF3_1 | Animal |
| GCF_020785935.1 | *Limosilactobacillus reuteri* | 11B | Animal |
| GCF_020785945.1 | *Limosilactobacillus reuteri* | 11A | Animal |
| GCF_020785975.1 | *Limosilactobacillus reuteri* | AP5 | Animal |
| GCF_020785995.1 | *Limosilactobacillus reuteri* | EF2_1 | Animal |
| GCF_020786015.1 | *Limosilactobacillus reuteri* | AP3 | Animal |
| GCF_020786045.1 | *Limosilactobacillus reuteri* | 4A | Animal |
| GCF_020786095.1 | *Limosilactobacillus reuteri* | AP1 | Animal |
| GCF_020978225.1 | *Limosilactobacillus reuteri* | - | Animal |
| GCF_021165875.1 | *Limosilactobacillus reuteri* | VHProbi E18 | Human |
| GCF_021228055.1 | *Limosilactobacillus reuteri* | VHProbi M07 | Human |
| GCF_021229095.1 | *Limosilactobacillus reuteri* | LLR-K67 | Human |
| GCF_021383585.1 | *Limosilactobacillus reuteri* | RTR | Food |
| GCF_021398615.1 | *Limosilactobacillus reuteri* | DS0384 | Human |
| GCF_021459965.1 | *Limosilactobacillus reuteri* | M2021619 | Human |
| GCF_022509245.1 | *Limosilactobacillus reuteri* | QAULRN18 | Food |
| GCF_022511545.1 | *Limosilactobacillus reuteri* | BR13-C2 | Animal |
| GCF_022642805.1 | *Limosilactobacillus reuteri* | L8 | Animal |
| GCF_023078415.1 | *Limosilactobacillus reuteri* | EFEL6901 | Human |
| GCF_023896335.1 | *Limosilactobacillus reuteri* | PH-3 | Animal |
| GCF_024622285.1 | *Limosilactobacillus reuteri* | DSM 28673 | Animal |
| GCF_024622305.1 | *Limosilactobacillus reuteri* | DSM 100192 | Animal |
| GCF_024622315.1 | *Limosilactobacillus reuteri* | DSM 100191 | Animal |
| GCF_024652885.1 | *Limosilactobacillus reuteri* | FN041 | Human |
| GCF_025189665.1 | *Limosilactobacillus reuteri* | CIRM-BIA 696 | Others |
| GCF_025189695.1 | *Limosilactobacillus reuteri* | CIRM-BIA 522 | Human |
| GCF_025189785.1 | *Limosilactobacillus reuteri* | CIRM-BIA 912 | Food |
| GCF_025189805.1 | *Limosilactobacillus reuteri* | CIRM-BIA 2121 | Food |
| GCF_025369755.1 | *Limosilactobacillus reuteri* | AM_LB1 | Animal |
| GCF_025515425.1 | *Limosilactobacillus reuteri* | SRCM210547 | Human |
| GCF_026183435.1 | *Limosilactobacillus reuteri* | BRD_L17 | Animal |
| GCF_028462705.1 | *Limosilactobacillus reuteri* | 92128 | Food |
| GCF_028656195.1 | *Limosilactobacillus reuteri* | SRCM217616 | Animal |
| GCF_028656225.1 | *Limosilactobacillus reuteri* | SRCM217617 | Animal |
| GCF_028656265.1 | *Limosilactobacillus reuteri* | SRCM217606 | Animal |
| GCF_028656315.1 | *Limosilactobacillus reuteri* | SRCM217608 | Animal |
| GCF_028656325.1 | *Limosilactobacillus reuteri* | SRCM217607 | Animal |
| GCF_028656395.1 | *Limosilactobacillus reuteri* | SRCM217611 | Animal |
| GCF_030262475.1 | *Limosilactobacillus reuteri* | BIO7251 | Food |
| GCF_030345055.1 | *Limosilactobacillus reuteri* | M1 | Animal |
| GCF_030418275.1 | *Limosilactobacillus reuteri* | ATCC PTA 4659 | Human |
| GCF_030517815.1 | *Limosilactobacillus reuteri* | 3 | Human |
| GCF_030585585.1 | *Limosilactobacillus reuteri* | NL02 | Human |
| GCF_033570435.1 | *Limosilactobacillus reuteri* | P43 | Animal |
| GCF_034424825.1 | *Limosilactobacillus reuteri* | C4 | Animal |
| GCF_034744995.1 | *Limosilactobacillus reuteri* | KCJ2K2682 | Animal |
| GCF_034745005.1 | *Limosilactobacillus reuteri* | KCJ2K2679 | Animal |
| GCF_034745135.1 | *Limosilactobacillus reuteri* | KCJ2K2653 | Animal |
| GCF_034745195.1 | *Limosilactobacillus reuteri* | KCJ2K2641 | Animal |
| GCF_034745275.1 | *Limosilactobacillus reuteri* | KCJ2K2638 | Animal |
| GCF_034745295.1 | *Limosilactobacillus reuteri* | KCJ2K2628 | Animal |
| GCF_034745375.1 | *Limosilactobacillus reuteri* | KCJ2K2620 | Animal |
| GCF_034745415.1 | *Limosilactobacillus reuteri* | KCJ2K2614 | Animal |
| GCF_034745435.1 | *Limosilactobacillus reuteri* | KCJ2K2606 | Animal |
| GCF_035200785.1 | *Limosilactobacillus reuteri* | C501 | Human |
| GCF_035928175.1 | *Limosilactobacillus reuteri* | VHProbi V43 | Food |
| GCF_036432135.1 | *Limosilactobacillus reuteri* | LTAD-12f | Food |
| GCF_036621875.1 | *Limosilactobacillus reuteri* | lp167-67 | Animal |
| GCF_036621915.1 | *Limosilactobacillus reuteri* | pg-3b | Animal |
| GCF_036903235.1 | *Limosilactobacillus reuteri* | TPC32 | Animal |
| GCF_037414455.1 | *Limosilactobacillus reuteri* | CUDS0384 | Human |
| GCF_037966725.1 | *Limosilactobacillus reuteri* | ATA-LTC-Lr050600 | Human |
| GCF_040142685.1 | *Limosilactobacillus reuteri* | LU150 | Human |
| GCF_040785065.1 | *Limosilactobacillus reuteri* | gbc_i | Others |
| GCF_040959015.1 | *Limosilactobacillus reuteri* | MG4722 | Human |
| GCF_040961095.1 | *Limosilactobacillus reuteri* | BG-R33 (DSM 33633) | Human |
| GCF_040962025.1 | *Limosilactobacillus reuteri* | ATCC PTA 5289 | Human |
| GCF_041226345.1 | *Limosilactobacillus reuteri* | 62 | Animal |
| GCF_042464795.1 | *Limosilactobacillus reuteri* | LDHa | Animal |
| GCF_042464815.1 | *Limosilactobacillus reuteri* | LSHe | Animal |
| GCF_045037805.1 | *Limosilactobacillus reuteri* | ZY15 | Animal |
| GCF_045269915.1 | *Limosilactobacillus reuteri* | MRD060 | Human |
| GCF_046763525.1 | *Limosilactobacillus reuteri* | NCDC958 | Human |
| GCF_047445745.1 | *Limosilactobacillus reuteri* | JLR.JH054 | Animal |
| GCF_047456375.1 | *Limosilactobacillus reuteri* | ZJ617 | Animal |
| GCF_049808515.1 | *Limosilactobacillus reuteri* | 6132 | Human |
| GCF_050313125.1 | *Limosilactobacillus reuteri* | SGI.205 | Animal |
| GCF_050320225.1 | *Limosilactobacillus reuteri* | LM1063 | Others |
| GCF_050845985.1 | *Limosilactobacillus reuteri* | HP-B1251 | Food |
| GCF_050846995.1 | *Limosilactobacillus reuteri* | TDM 0502 | Animal |
| GCF_050967595.1 | *Limosilactobacillus reuteri* | CHF7-2 | Animal |
| GCF_051372025.1 | *Limosilactobacillus reuteri* | HC187 | Animal |
| GCF_051372505.1 | *Limosilactobacillus reuteri* | HC1146 | Animal |
| GCF_051670475.1 | *Limosilactobacillus reuteri* | CACC607 | Animal |
| GCF_051911155.1 | *Limosilactobacillus reuteri* | A | Animal |
| GCF_052115945.1 | *Limosilactobacillus reuteri* | Sow4_H10 | Animal |
| GCF_052146115.1 | *Limosilactobacillus reuteri* | S8 | Animal |
| GCF_052280555.1 | *Limosilactobacillus reuteri* | iVE-9 | Human |
| GCF_900093565.1 | *Limosilactobacillus reuteri* | L6798 | Others |
| GCF_901600665.1 | *Limosilactobacillus reuteri* | Marseille-P4870 | Food |
| GCF_901600675.1 | *Limosilactobacillus reuteri* | Marseille-P5461 | Human |
| GCF_901600695.1 | *Limosilactobacillus reuteri* | Marseille-P5460 | Human |
| GCF_901600705.1 | *Limosilactobacillus reuteri* | Marseille-P4904 | Human |
| GCF_910574215.1 | *Limosilactobacillus reuteri* | MGBC000067 | Animal |
| GCF_910574445.1 | *Limosilactobacillus reuteri* | MGBC000027 | Animal |
| GCF_910574725.1 | *Limosilactobacillus reuteri* | MGBC000088 | Animal |
| GCF_910574795.1 | *Limosilactobacillus reuteri* | MGBC000117 | Animal |
| GCF_910576155.1 | *Limosilactobacillus reuteri* | MGBC000225 | Animal |
| GCF_925280345.1 | *Limosilactobacillus reuteri* | IM842 | Others |
| GCF_925285275.1 | *Limosilactobacillus reuteri* | IM566 | Others |
| GCF_940926095.1 | *Limosilactobacillus reuteri* | AMBV339 | Human |
| GCF_009362935.1 | *Limosilactobacillus vaginalis* | LV515 | Human |
| GCF_022456495.1 | *Limosilactobacillus vaginalis* | CBA8_S776 | Human |
| GCF_022456515.1 | *Limosilactobacillus vaginalis* | BHK11_S832 | Human |
| GCF_027156105.1 | *Limosilactobacillus vaginalis* | C0148A2 | Human |
| GCF_027156125.1 | *Limosilactobacillus vaginalis* | C0148A1 | Human |
| GCF_027156145.1 | *Limosilactobacillus vaginalis* | C0148A5 | Human |
| GCF_027156165.1 | *Limosilactobacillus vaginalis* | C0141B4 | Human |
| GCF_027156185.1 | *Limosilactobacillus vaginalis* | C0141B3 | Human |
| GCF_027156205.1 | *Limosilactobacillus vaginalis* | C0141B1 | Human |
| GCF_027156215.1 | *Limosilactobacillus vaginalis* | C0141B2 | Human |
| GCF_027156305.1 | *Limosilactobacillus vaginalis* | C0140C5 | Human |
| GCF_027156545.1 | *Limosilactobacillus vaginalis* | C0030B4 | Human |
| GCF_027156605.1 | *Limosilactobacillus vaginalis* | C0030A5 | Human |
| GCF_027156625.1 | *Limosilactobacillus vaginalis* | C0030A3 | Human |
| GCF_027156665.1 | *Limosilactobacillus vaginalis* | C0030A2 | Human |
| GCF_027156685.1 | *Limosilactobacillus vaginalis* | C0030A1 | Human |
| GCF_027156755.1 | *Limosilactobacillus vaginalis* | C0030A4 | Human |
| GCF_027682185.1 | *Limosilactobacillus vaginalis* | AF54-2pH5A | Human |
| GCF_051371785.1 | *Limosilactobacillus vaginalis* | HC49 | Animal |
| GCF_947381785.1 | *Limosilactobacillus vaginalis* | J29 | Others |
