# Supplementary figures and images for: From Fermentation to Function: Genomic Diversity and Probiotic Potential in the Reclassified Lactobacillus Lineage
Source: Comput Struct Biotechnol J. 2026 Mar 16;35(1):0004. doi: 10.34133/csbj.0004 (PMC13067958; doi:10.34133/csbj.0004)

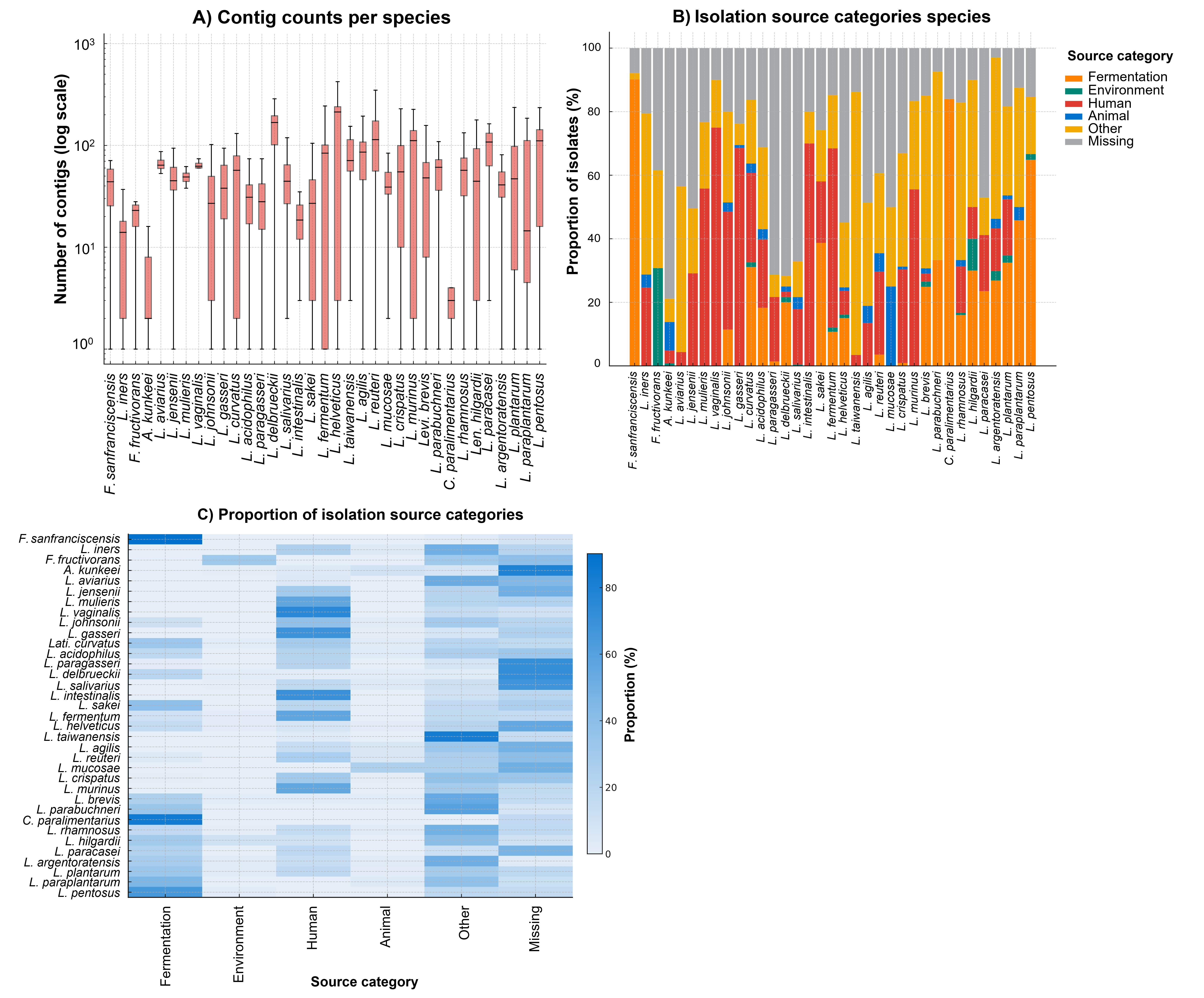

Supplement: Supplementary 1 — Figs. S1 to S4 Tables S1 to S6 [file csbj.0004.f0004.zip › FigureS1.png]

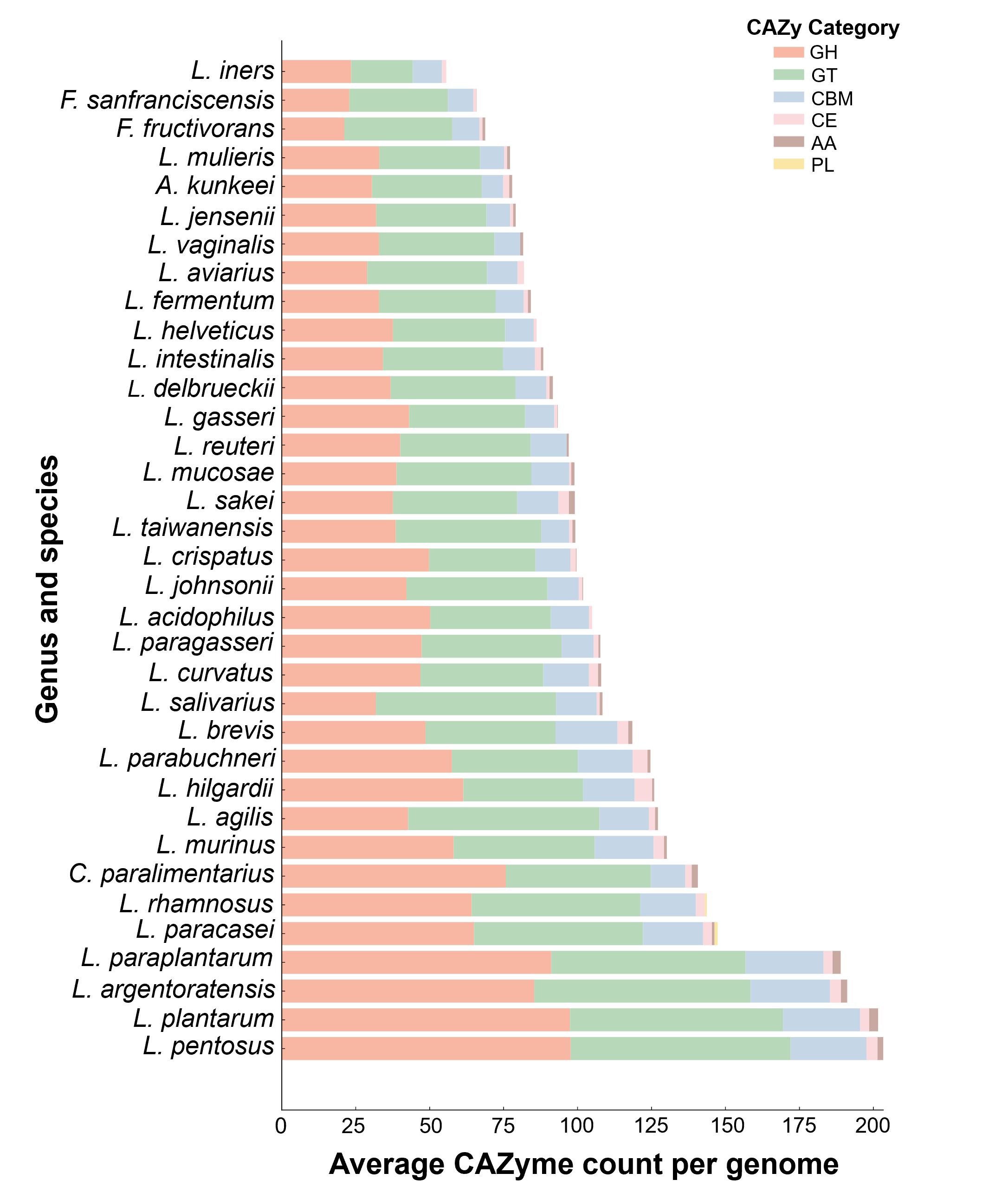

Supplement: Supplementary 1 — Figs. S1 to S4 Tables S1 to S6 [file csbj.0004.f0004.zip › FigureS2.png]

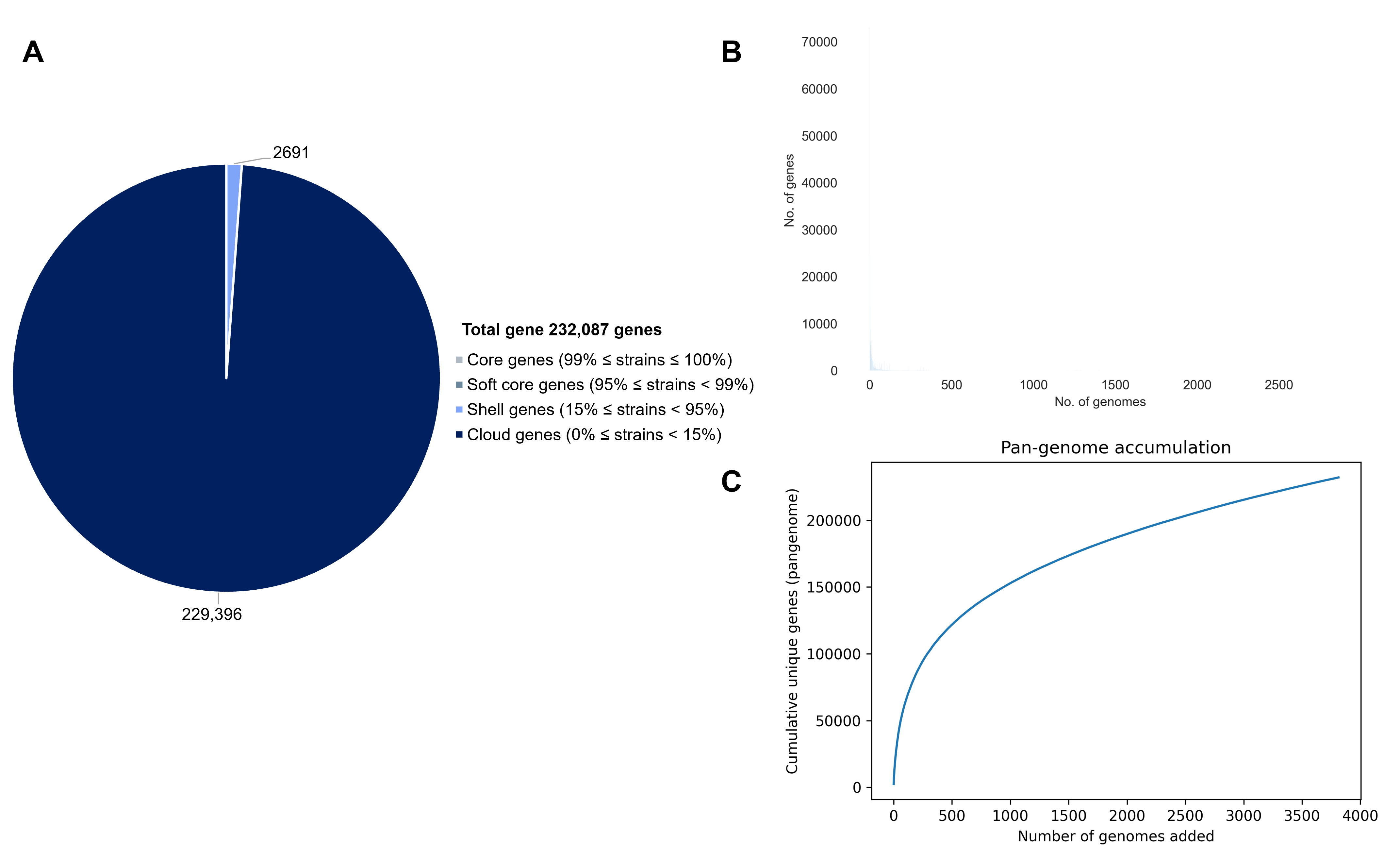

Supplement: Supplementary 1 — Figs. S1 to S4 Tables S1 to S6 [file csbj.0004.f0004.zip › FigureS3.png]
